# Supplementary figures and images for: Evaluating deep learning-based melanoma classification using immunohistochemistry and routine histology: A three center study (part 1 of 7)
Source: PLoS One. 2024 Jan 19;19(1):e0297146. doi: 10.1371/journal.pone.0297146 (PMC10798511; doi:10.1371/journal.pone.0297146)

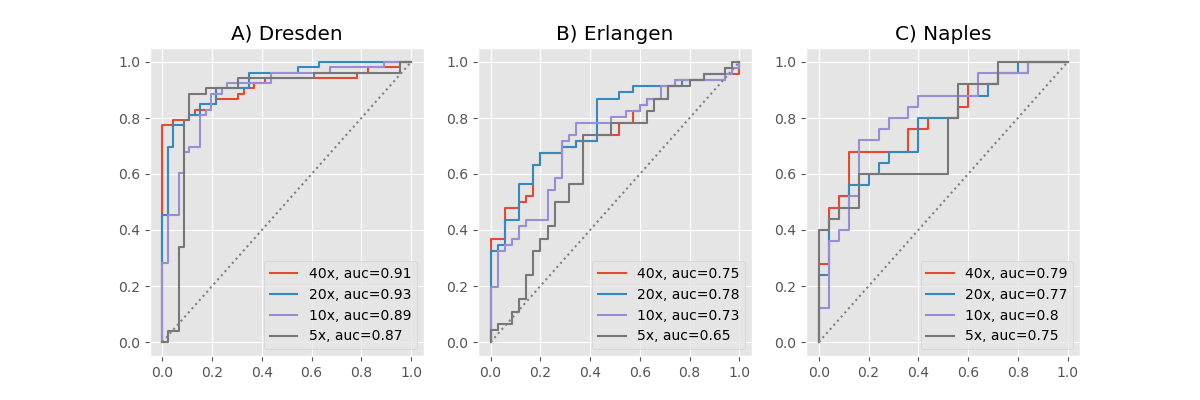

Supplement: S2 Fig — A: Results from Dresden B: Results from Erlangen C: Results from Naples. Red: 40x magnification Blue: 20x magnification Purple: 10x magnification Gray: 5x magnification. (TIF) [file pone.0297146.s002.tif]

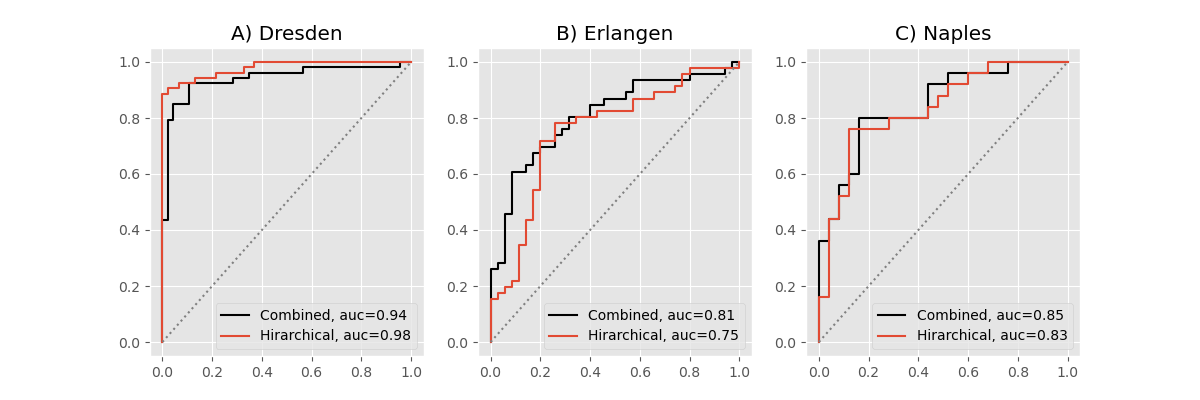

Supplement: S3 Fig — A: Results from Dresden B: Results from Erlangen C: Results from Naples. Black: Results of the combined approach using H&E and MElanA for all lesions Red: Hierarchical approach using MelanA-stained tissue only for H&E-based uncertain lesions. (TIF) [file pone.0297146.s003.tif]

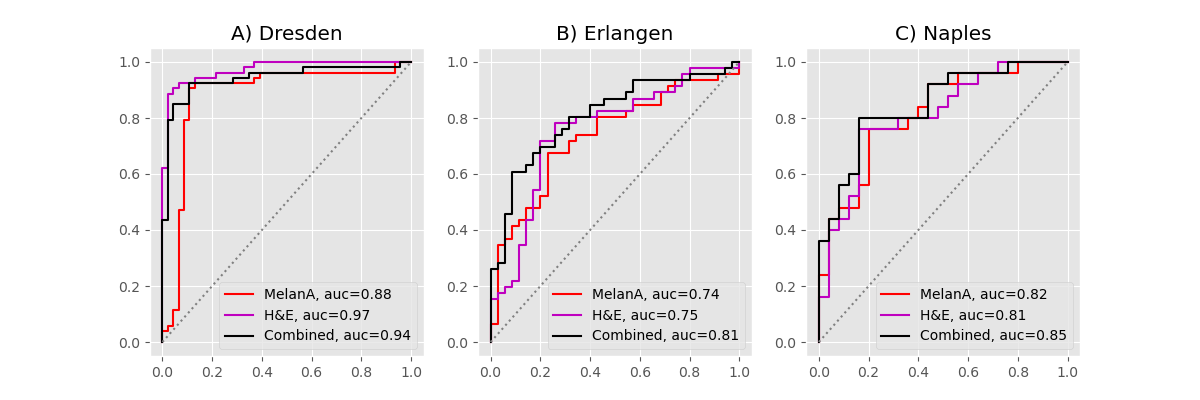

Supplement: S4 Fig — A: Results from Dresden B: Results from Erlangen C: Results from Naples. Red: MelanA-based performance taking all magnifications into account Purple: H&E-based performance Black: combined model using H&E as well as MelanA by aggregating the individual scores. (TIF) [file pone.0297146.s004.tif]

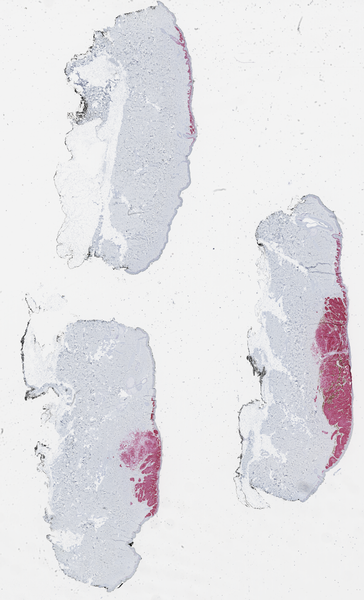

Supplement: S1 Dataset — (ZIP) [file pone.0297146.s007.zip › MelanA/522500_MelanA.png]

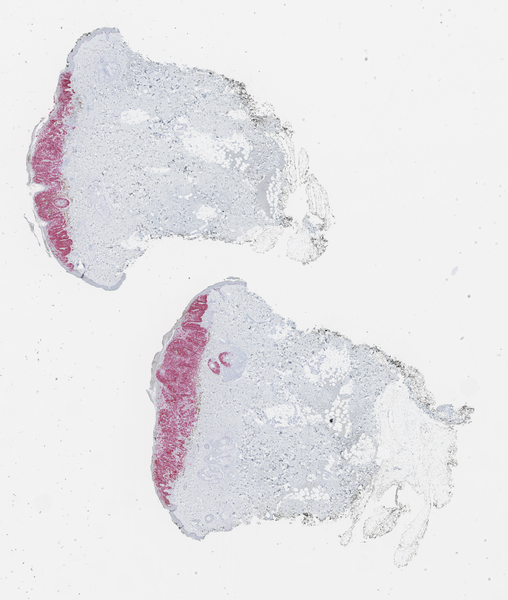

Supplement: S1 Dataset — (ZIP) [file pone.0297146.s007.zip › MelanA/341033_MelanA.png]

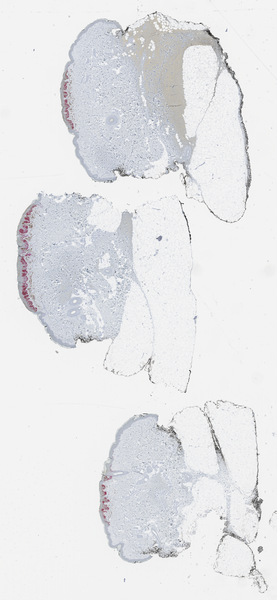

Supplement: S1 Dataset — (ZIP) [file pone.0297146.s007.zip › MelanA/516621_MelanA.png]

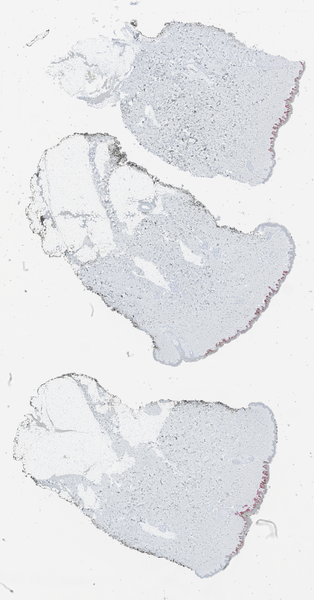

Supplement: S1 Dataset — (ZIP) [file pone.0297146.s007.zip › MelanA/725823_MelanA.png]

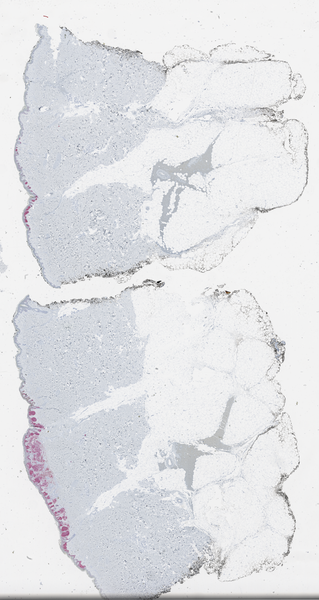

Supplement: S1 Dataset — (ZIP) [file pone.0297146.s007.zip › MelanA/261250_MelanA.png]

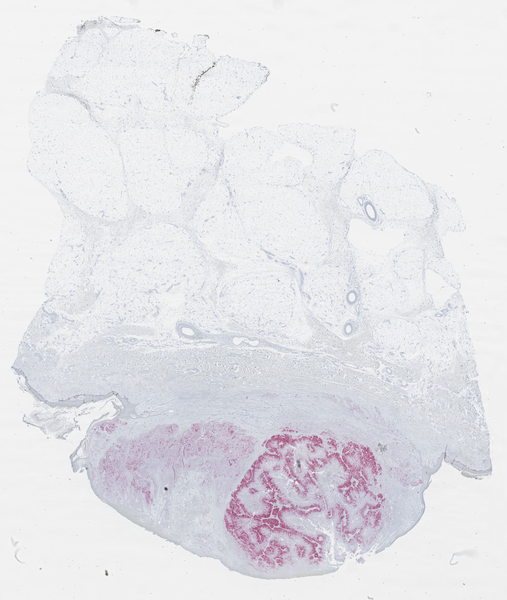

Supplement: S1 Dataset — (ZIP) [file pone.0297146.s007.zip › MelanA/797778_MelanA.png]

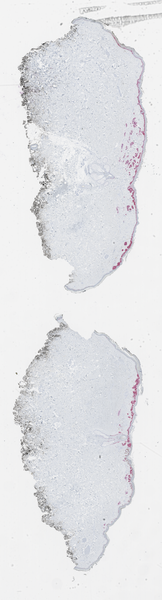

Supplement: S1 Dataset — (ZIP) [file pone.0297146.s007.zip › MelanA/203200_MelanA.png]

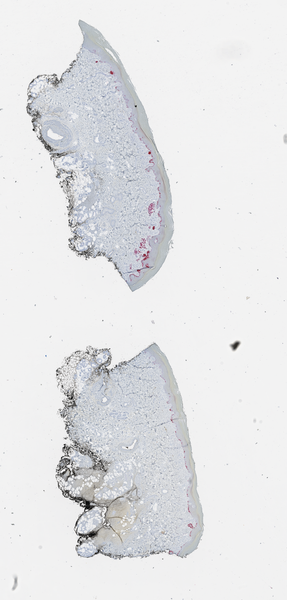

Supplement: S1 Dataset — (ZIP) [file pone.0297146.s007.zip › MelanA/774309-2_MelanA.png]

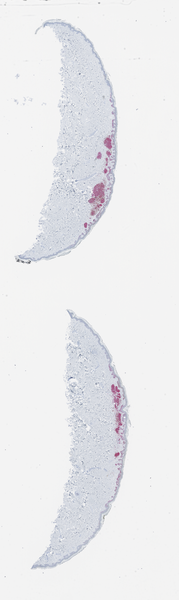

Supplement: S1 Dataset — (ZIP) [file pone.0297146.s007.zip › MelanA/428772_MelanA.png]

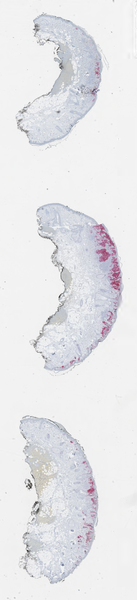

Supplement: S1 Dataset — (ZIP) [file pone.0297146.s007.zip › MelanA/799469_MelanA.png]

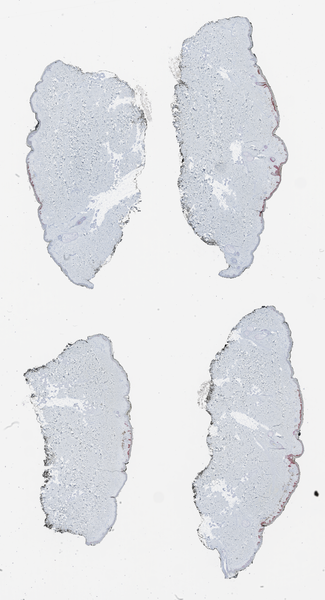

Supplement: S1 Dataset — (ZIP) [file pone.0297146.s007.zip › MelanA/796338_MelanA.png]

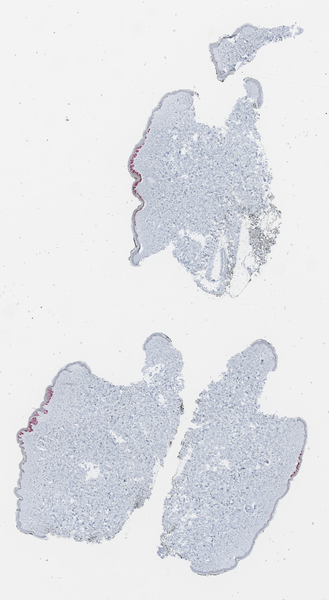

Supplement: S1 Dataset — (ZIP) [file pone.0297146.s007.zip › MelanA/688070-1_MelanA.png]

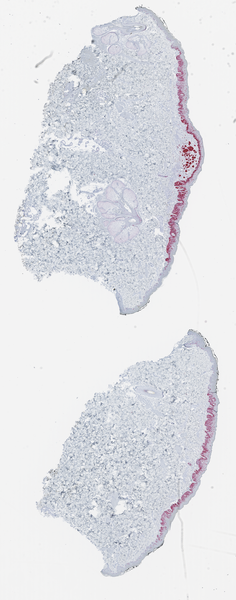

Supplement: S1 Dataset — (ZIP) [file pone.0297146.s007.zip › MelanA/692561_MelanA.png]

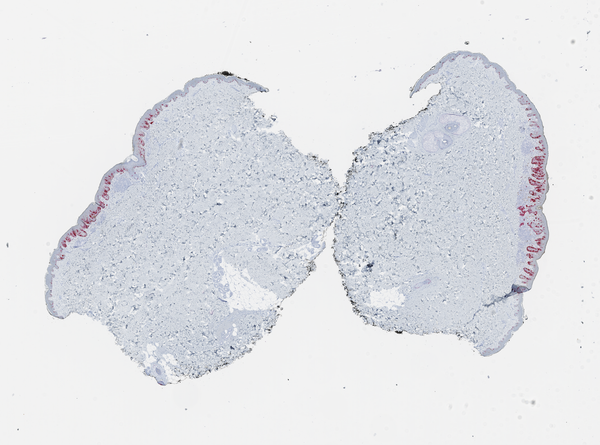

Supplement: S1 Dataset — (ZIP) [file pone.0297146.s007.zip › MelanA/333072_MelanA.png]

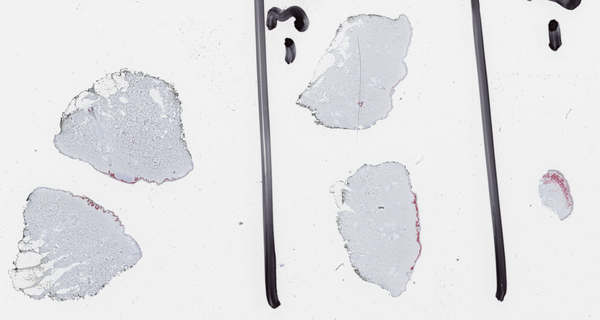

Supplement: S1 Dataset — (ZIP) [file pone.0297146.s007.zip › MelanA/773004-2_MelanA.png]

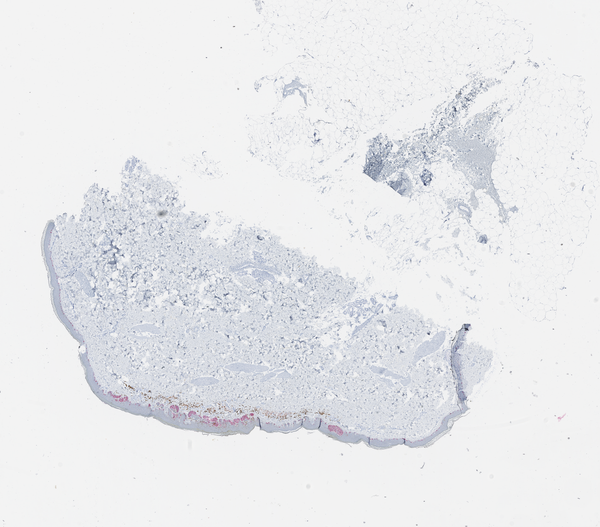

Supplement: S1 Dataset — (ZIP) [file pone.0297146.s007.zip › MelanA/420270_MelanA.png]

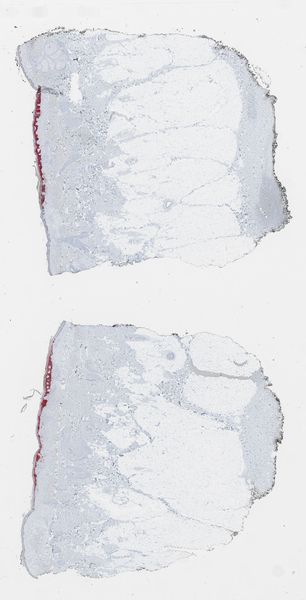

Supplement: S1 Dataset — (ZIP) [file pone.0297146.s007.zip › MelanA/773387_MelanA.png]

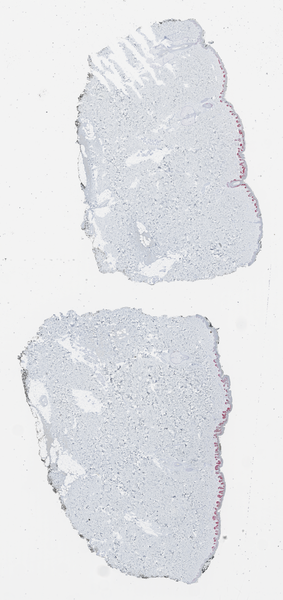

Supplement: S1 Dataset — (ZIP) [file pone.0297146.s007.zip › MelanA/732460_MelanA.png]

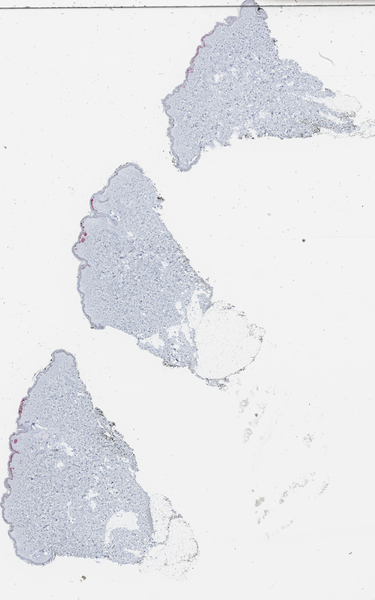

Supplement: S1 Dataset — (ZIP) [file pone.0297146.s007.zip › MelanA/688070-2_MelanA.png]

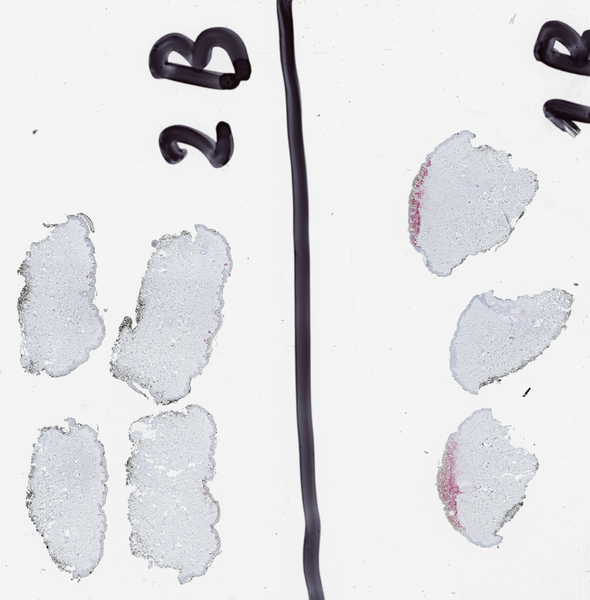

Supplement: S1 Dataset — (ZIP) [file pone.0297146.s007.zip › MelanA/550664-1_MelanA.png]

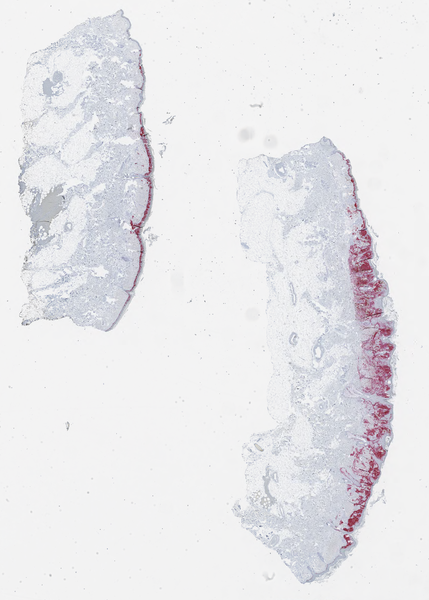

Supplement: S1 Dataset — (ZIP) [file pone.0297146.s007.zip › MelanA/856413_MelanA.png]

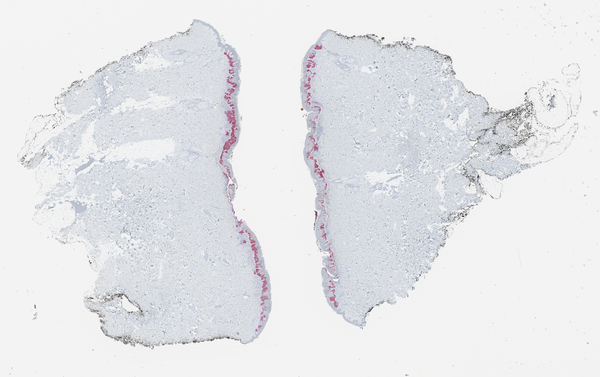

Supplement: S1 Dataset — (ZIP) [file pone.0297146.s007.zip › MelanA/303511_MelanA.png]

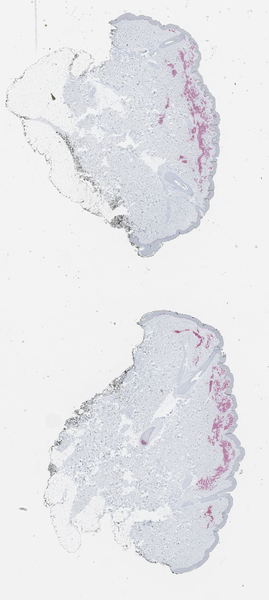

Supplement: S1 Dataset — (ZIP) [file pone.0297146.s007.zip › MelanA/115528_MelanA.png]

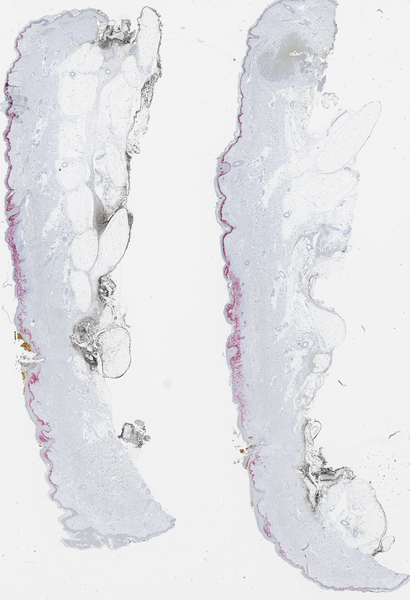

Supplement: S1 Dataset — (ZIP) [file pone.0297146.s007.zip › MelanA/545094_MelanA.png]

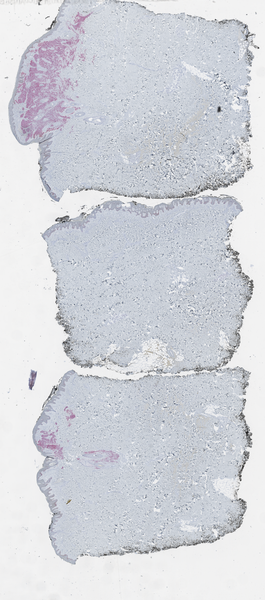

Supplement: S1 Dataset — (ZIP) [file pone.0297146.s007.zip › MelanA/546670_MelanA.png]

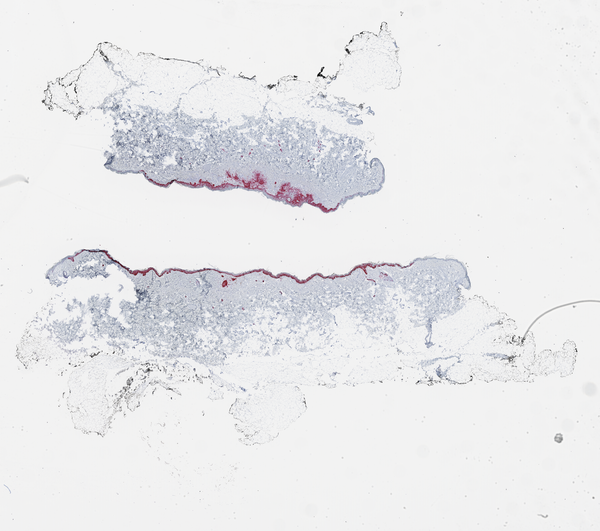

Supplement: S1 Dataset — (ZIP) [file pone.0297146.s007.zip › MelanA/693169_MelanA.png]

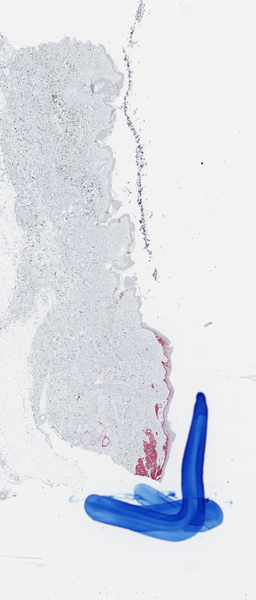

Supplement: S1 Dataset — (ZIP) [file pone.0297146.s007.zip › MelanA/619807-2_MelanA.png]

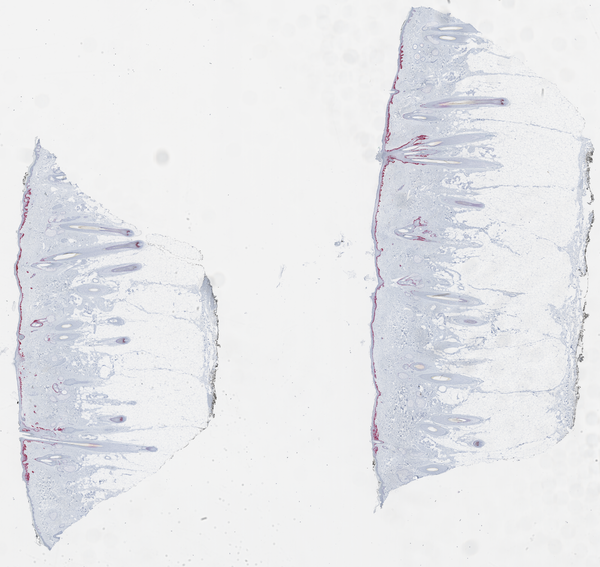

Supplement: S1 Dataset — (ZIP) [file pone.0297146.s007.zip › MelanA/451085_MelanA.png]

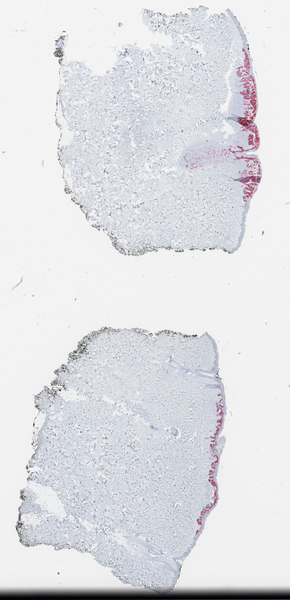

Supplement: S1 Dataset — (ZIP) [file pone.0297146.s007.zip › MelanA/108136_MelanA.png]

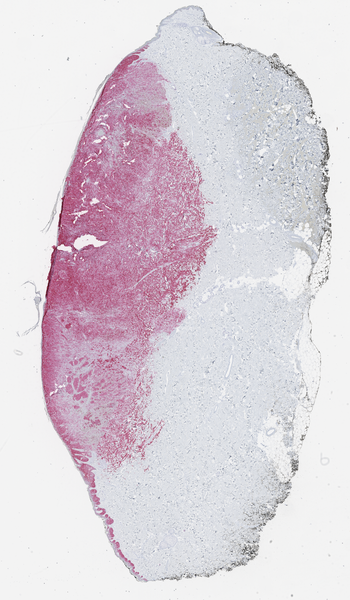

Supplement: S1 Dataset — (ZIP) [file pone.0297146.s007.zip › MelanA/219350_MelanA.png]

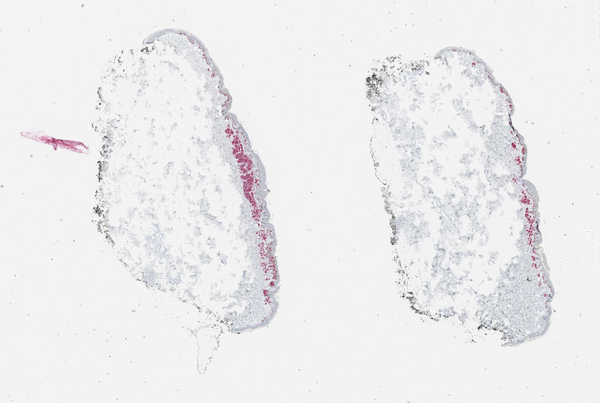

Supplement: S1 Dataset — (ZIP) [file pone.0297146.s007.zip › MelanA/115980_MelanA.png]

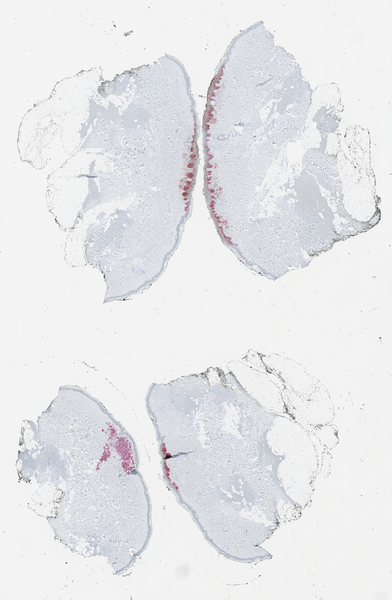

Supplement: S1 Dataset — (ZIP) [file pone.0297146.s007.zip › MelanA/262918_MelanA.png]

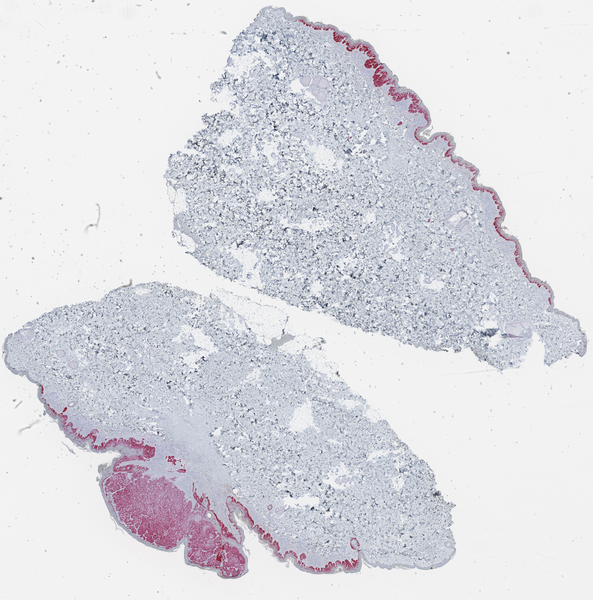

Supplement: S1 Dataset — (ZIP) [file pone.0297146.s007.zip › MelanA/661753_MelanA.png]

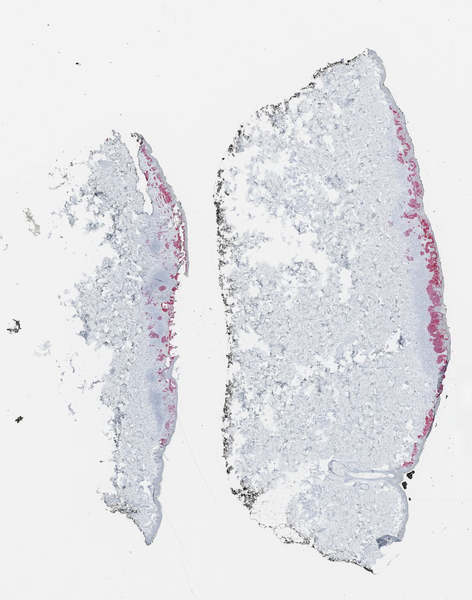

Supplement: S1 Dataset — (ZIP) [file pone.0297146.s007.zip › MelanA/115670_MelanA.png]

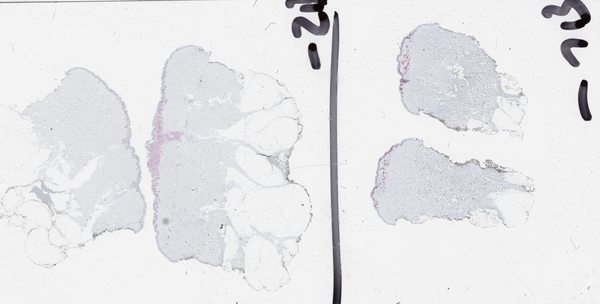

Supplement: S1 Dataset — (ZIP) [file pone.0297146.s007.zip › MelanA/282956-1_MelanA.png]

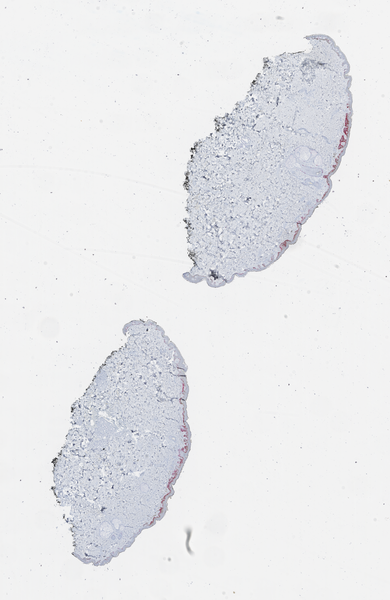

Supplement: S1 Dataset — (ZIP) [file pone.0297146.s007.zip › MelanA/792537_MelanA.png]

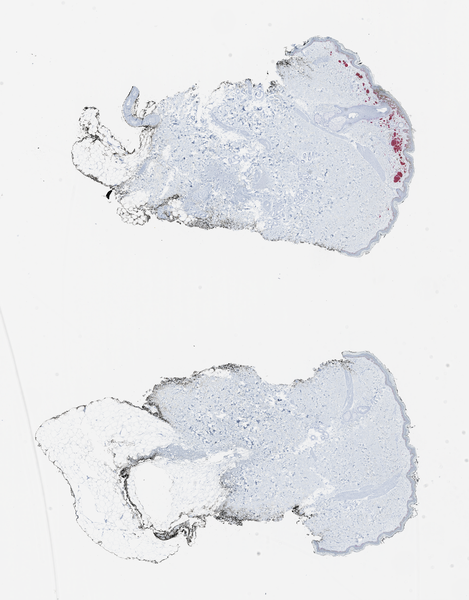

Supplement: S1 Dataset — (ZIP) [file pone.0297146.s007.zip › MelanA/425593-2_MelanA.png]

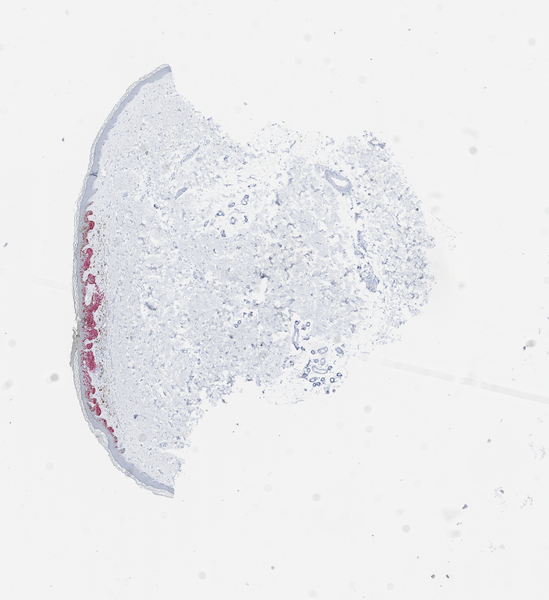

Supplement: S1 Dataset — (ZIP) [file pone.0297146.s007.zip › MelanA/495834_MelanA.png]

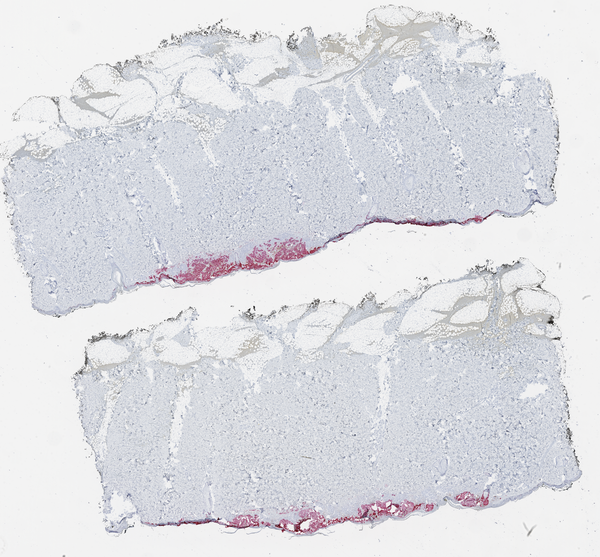

Supplement: S1 Dataset — (ZIP) [file pone.0297146.s007.zip › MelanA/220100_MelanA.png]

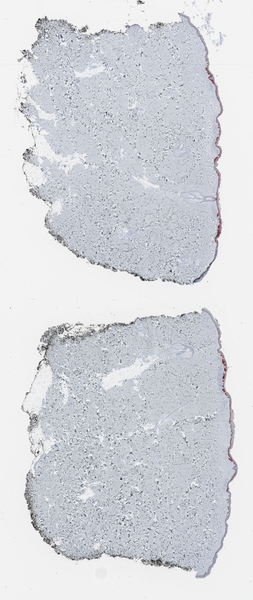

Supplement: S1 Dataset — (ZIP) [file pone.0297146.s007.zip › MelanA/749176_MelanA.png]

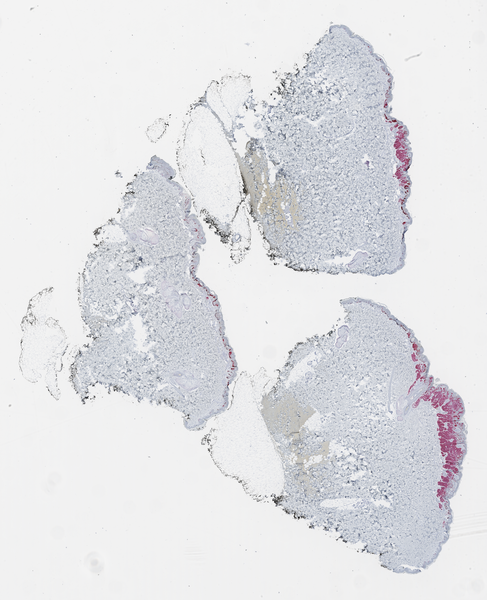

Supplement: S1 Dataset — (ZIP) [file pone.0297146.s007.zip › MelanA/556823_MelanA.png]

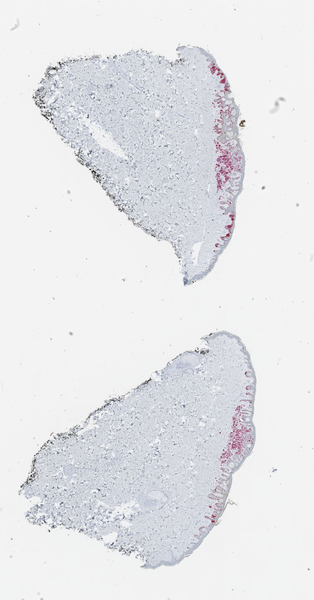

Supplement: S1 Dataset — (ZIP) [file pone.0297146.s007.zip › MelanA/118092_MelanA.png]

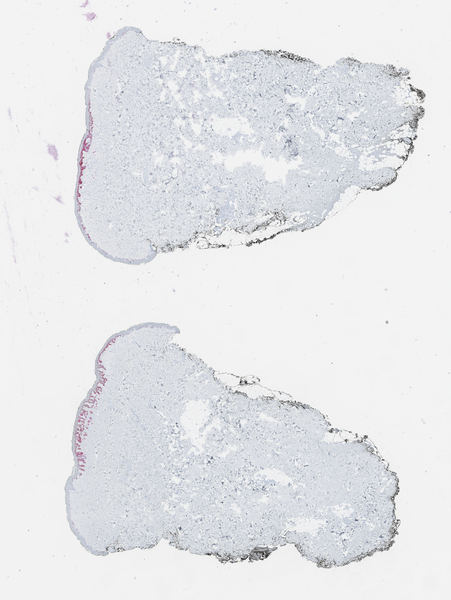

Supplement: S1 Dataset — (ZIP) [file pone.0297146.s007.zip › MelanA/230812_MelanA.png]

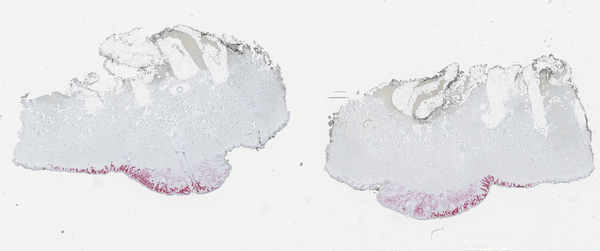

Supplement: S1 Dataset — (ZIP) [file pone.0297146.s007.zip › MelanA/809379_MelanA.png]

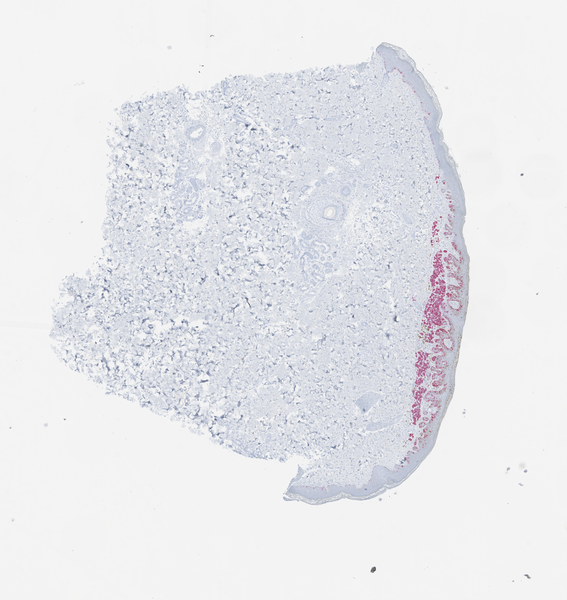

Supplement: S1 Dataset — (ZIP) [file pone.0297146.s007.zip › MelanA/329337_MelanA.png]

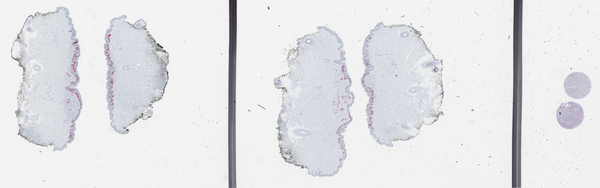

Supplement: S1 Dataset — (ZIP) [file pone.0297146.s007.zip › MelanA/437272_MelanA.png]

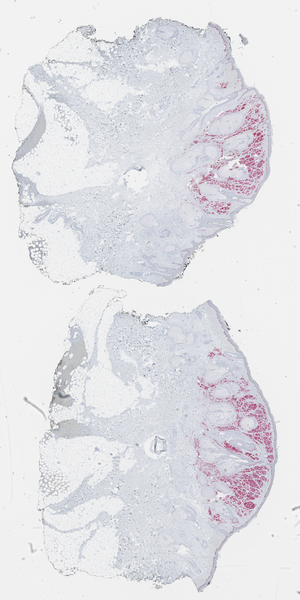

Supplement: S1 Dataset — (ZIP) [file pone.0297146.s007.zip › MelanA/680443_MelanA.png]

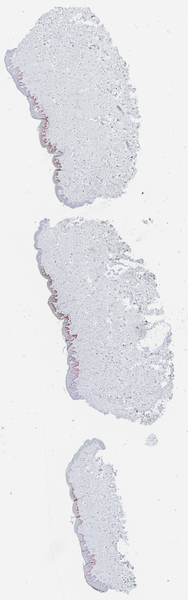

Supplement: S1 Dataset — (ZIP) [file pone.0297146.s007.zip › MelanA/542754-2_MelanA.png]

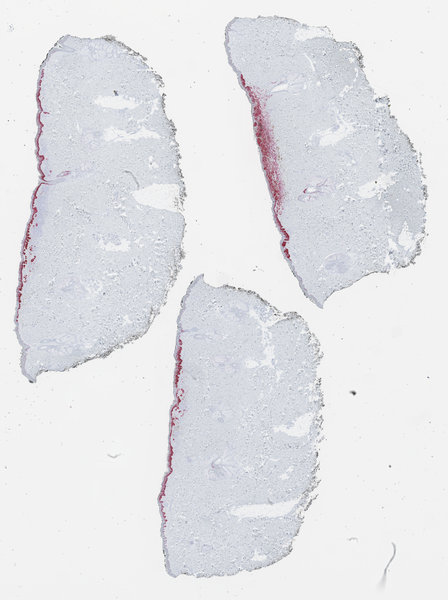

Supplement: S1 Dataset — (ZIP) [file pone.0297146.s007.zip › MelanA/761124_MelanA.png]

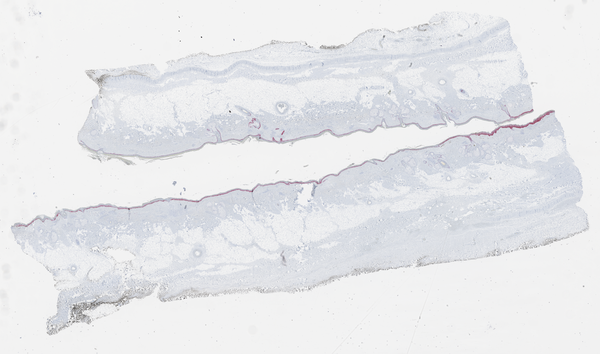

Supplement: S1 Dataset — (ZIP) [file pone.0297146.s007.zip › MelanA/777041_MelanA.png]

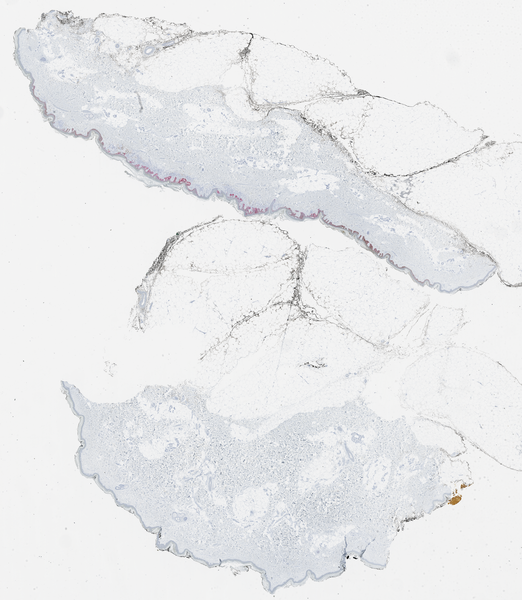

Supplement: S1 Dataset — (ZIP) [file pone.0297146.s007.zip › MelanA/284284_MelanA.png]

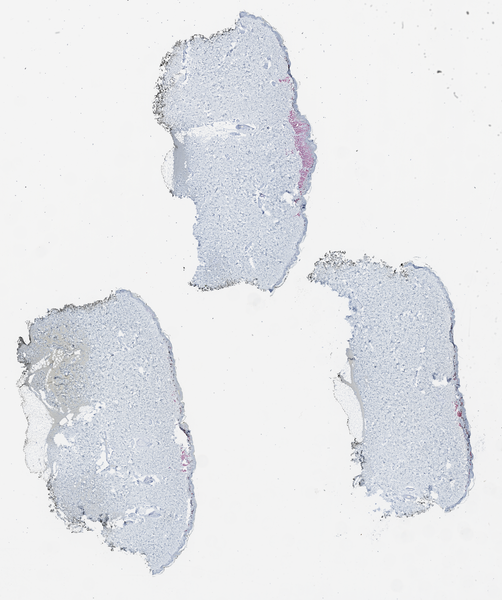

Supplement: S1 Dataset — (ZIP) [file pone.0297146.s007.zip › MelanA/430532_MelanA.png]

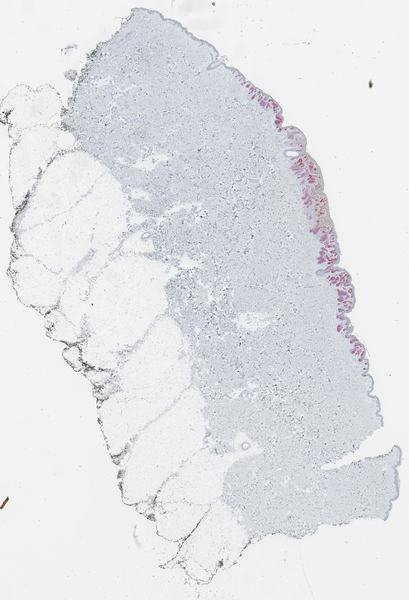

Supplement: S1 Dataset — (ZIP) [file pone.0297146.s007.zip › MelanA/293101_MelanA.png]

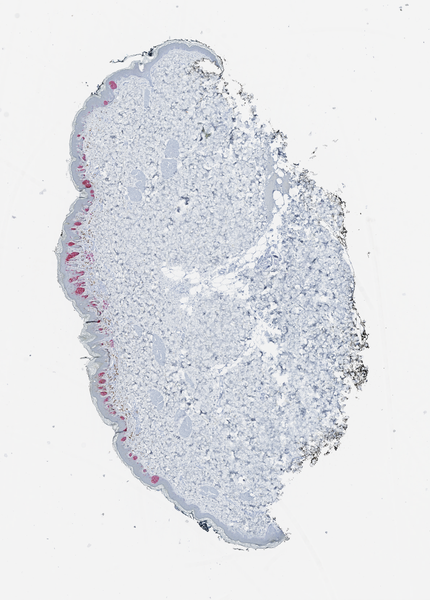

Supplement: S1 Dataset — (ZIP) [file pone.0297146.s007.zip › MelanA/484480_MelanA.png]

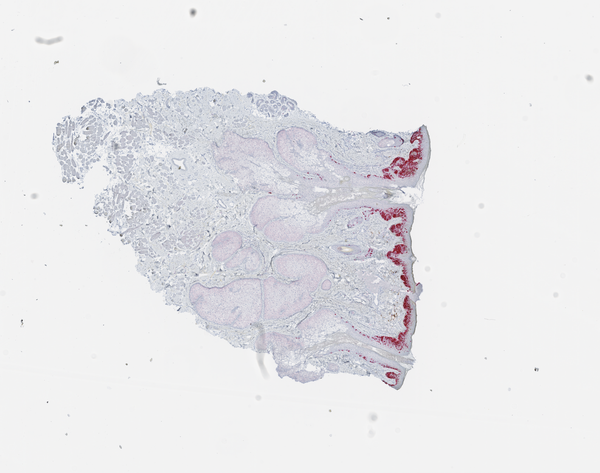

Supplement: S1 Dataset — (ZIP) [file pone.0297146.s007.zip › MelanA/697339_MelanA.png]

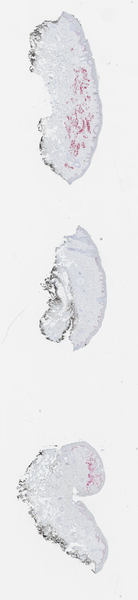

Supplement: S1 Dataset — (ZIP) [file pone.0297146.s007.zip › MelanA/300473_MelanA.png]

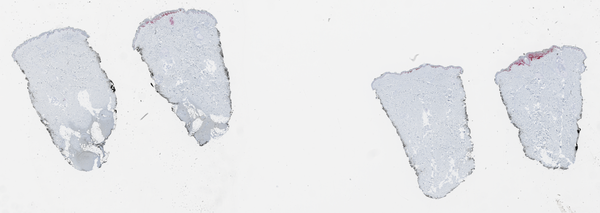

Supplement: S1 Dataset — (ZIP) [file pone.0297146.s007.zip › MelanA/425593-1_MelanA.png]

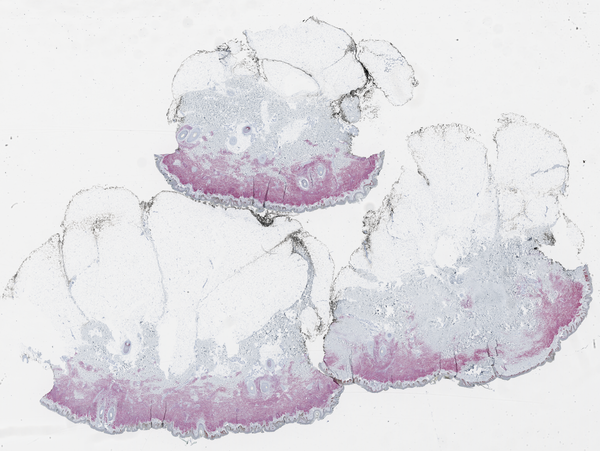

Supplement: S1 Dataset — (ZIP) [file pone.0297146.s007.zip › MelanA/482540_MelanA.png]

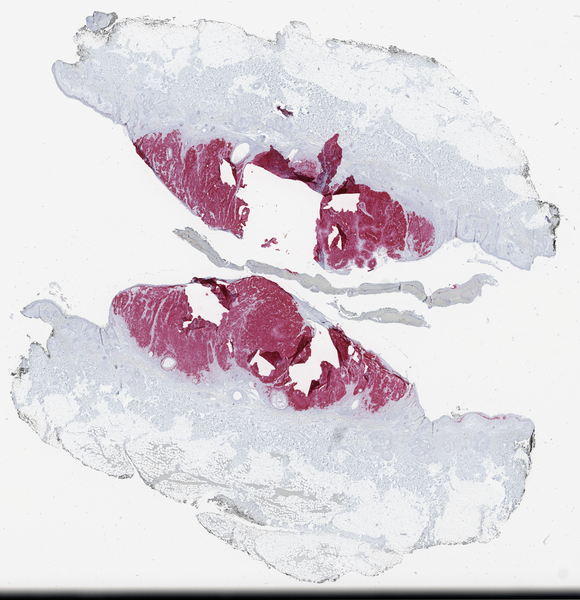

Supplement: S1 Dataset — (ZIP) [file pone.0297146.s007.zip › MelanA/745192-1_MelanA.png]

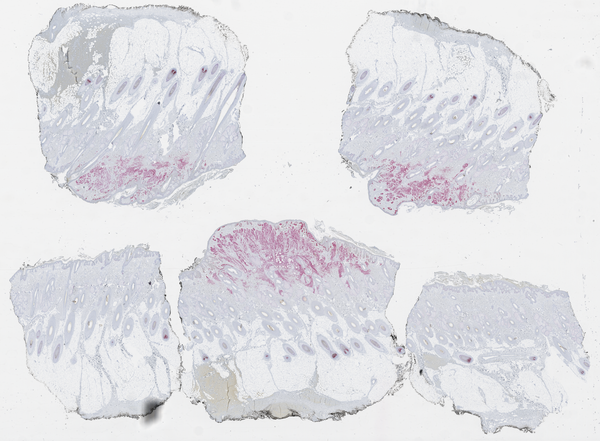

Supplement: S1 Dataset — (ZIP) [file pone.0297146.s007.zip › MelanA/546569-2_MelanA.png]

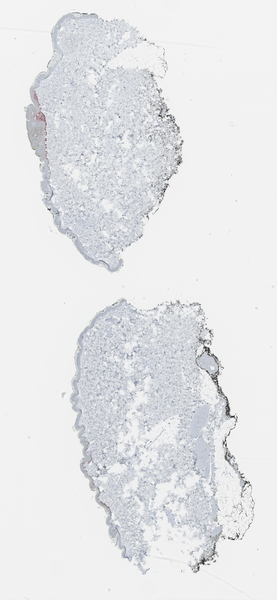

Supplement: S1 Dataset — (ZIP) [file pone.0297146.s007.zip › MelanA/823353_MelanA.png]

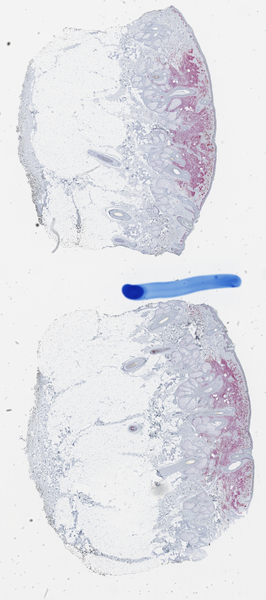

Supplement: S1 Dataset — (ZIP) [file pone.0297146.s007.zip › MelanA/667443_MelanA.png]

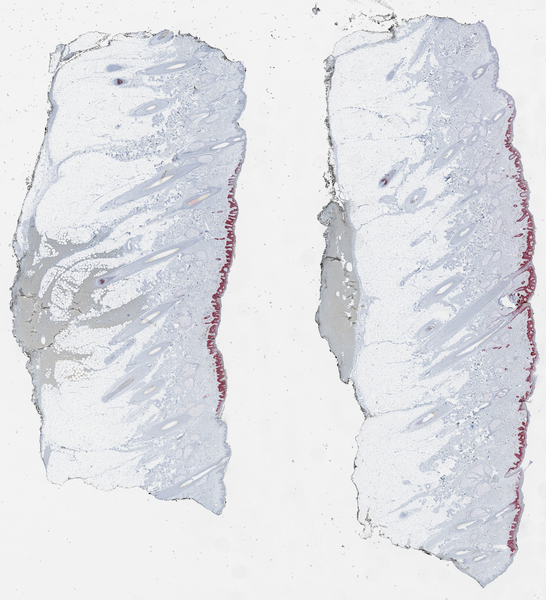

Supplement: S1 Dataset — (ZIP) [file pone.0297146.s007.zip › MelanA/583846_MelanA.png]

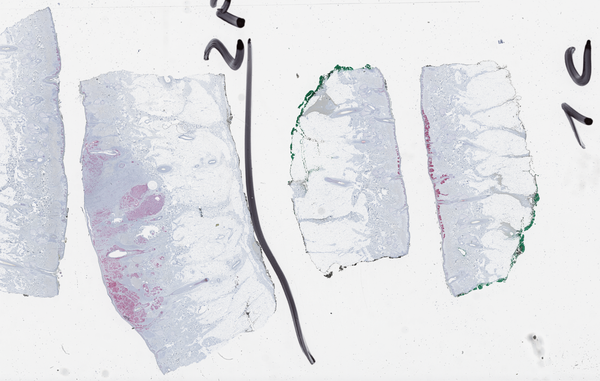

Supplement: S1 Dataset — (ZIP) [file pone.0297146.s007.zip › MelanA/431691-2_MelanA.png]

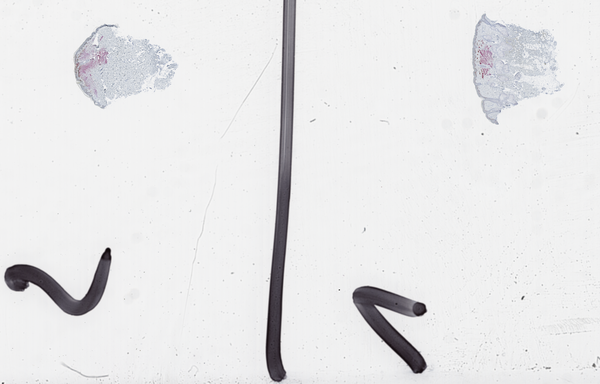

Supplement: S1 Dataset — (ZIP) [file pone.0297146.s007.zip › MelanA/314572-2_MelanA.png]

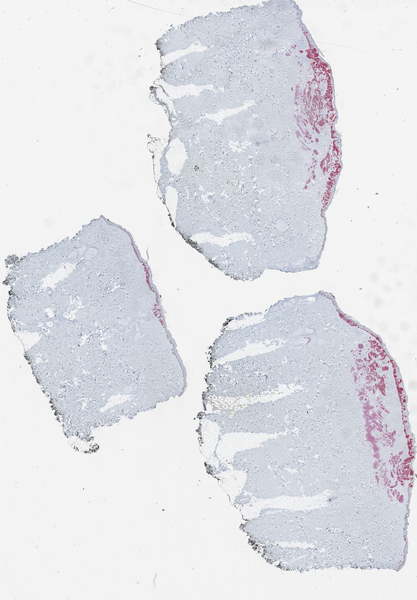

Supplement: S1 Dataset — (ZIP) [file pone.0297146.s007.zip › MelanA/487457_MelanA.png]

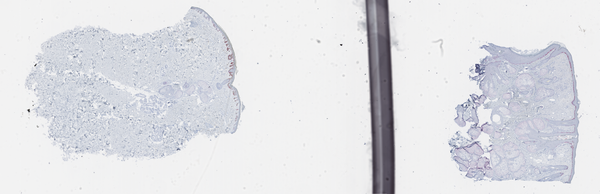

Supplement: S1 Dataset — (ZIP) [file pone.0297146.s007.zip › MelanA/502423_MelanA.png]

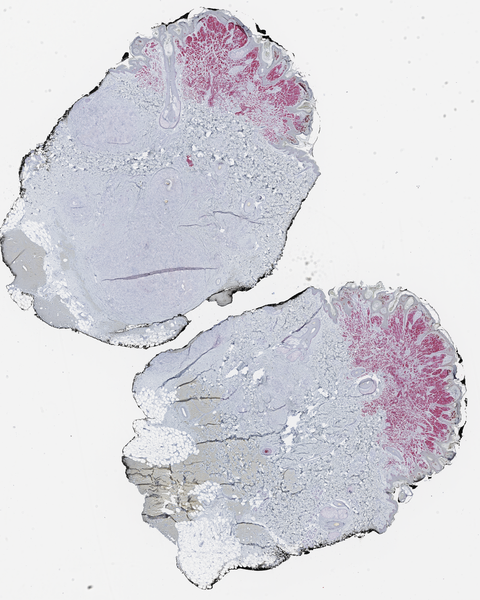

Supplement: S1 Dataset — (ZIP) [file pone.0297146.s007.zip › MelanA/336057_MelanA.png]

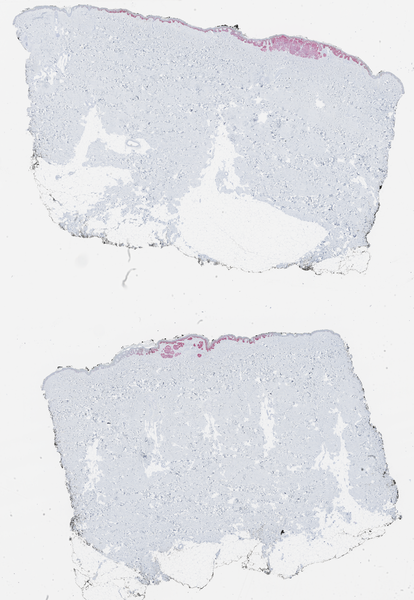

Supplement: S1 Dataset — (ZIP) [file pone.0297146.s007.zip › MelanA/411147_MelanA.png]

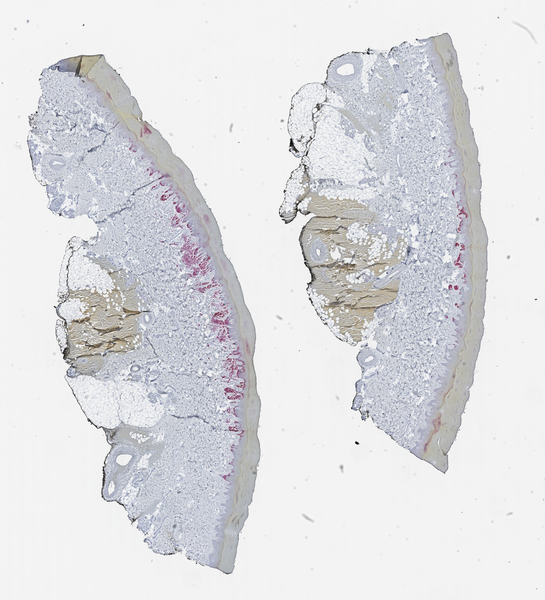

Supplement: S1 Dataset — (ZIP) [file pone.0297146.s007.zip › MelanA/334622_MelanA.png]

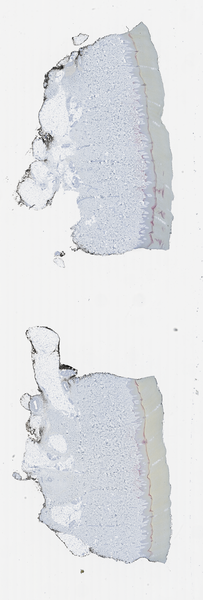

Supplement: S1 Dataset — (ZIP) [file pone.0297146.s007.zip › MelanA/450912-2_MelanA.png]

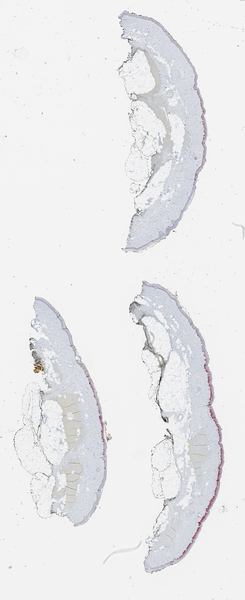

Supplement: S1 Dataset — (ZIP) [file pone.0297146.s007.zip › MelanA/792992_MelanA.png]

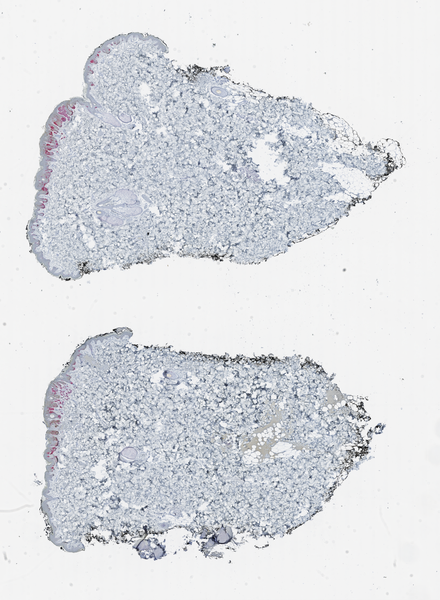

Supplement: S1 Dataset — (ZIP) [file pone.0297146.s007.zip › MelanA/407837_MelanA.png]

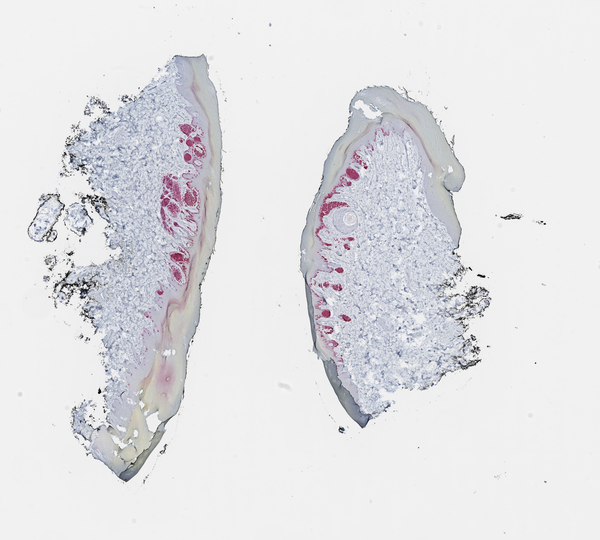

Supplement: S1 Dataset — (ZIP) [file pone.0297146.s007.zip › MelanA/713600_MelanA.png]

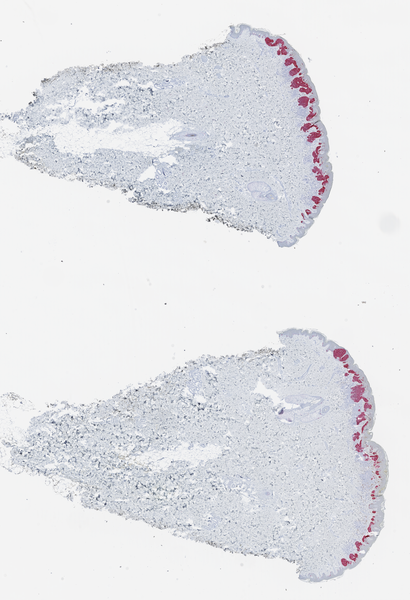

Supplement: S1 Dataset — (ZIP) [file pone.0297146.s007.zip › MelanA/105587_MelanA.png]

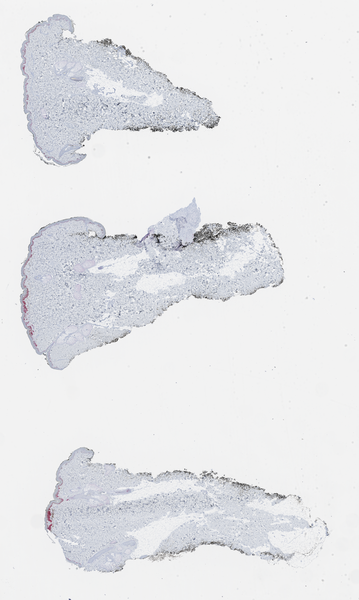

Supplement: S1 Dataset — (ZIP) [file pone.0297146.s007.zip › MelanA/483191_MelanA.png]

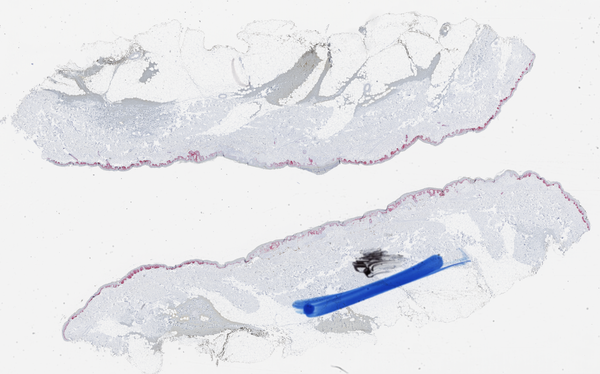

Supplement: S1 Dataset — (ZIP) [file pone.0297146.s007.zip › MelanA/678133_MelanA.png]

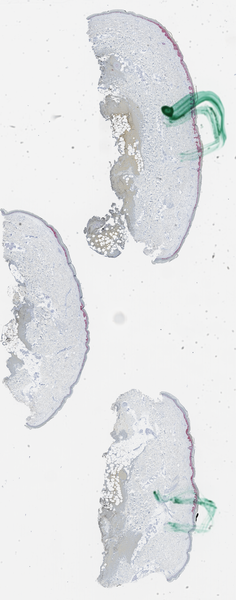

Supplement: S1 Dataset — (ZIP) [file pone.0297146.s007.zip › MelanA/663974_MelanA.png]

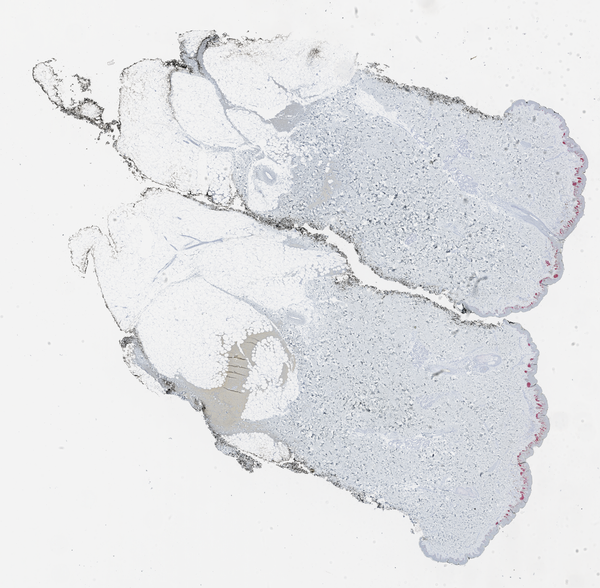

Supplement: S1 Dataset — (ZIP) [file pone.0297146.s007.zip › MelanA/550193-1_MelanA.png]

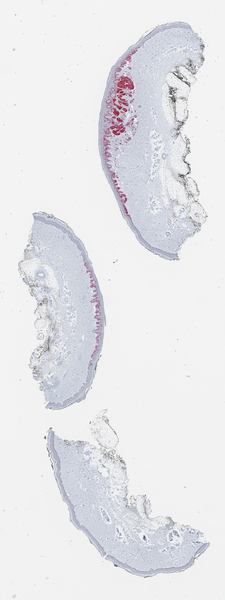

Supplement: S1 Dataset — (ZIP) [file pone.0297146.s007.zip › MelanA/699110_MelanA.png]

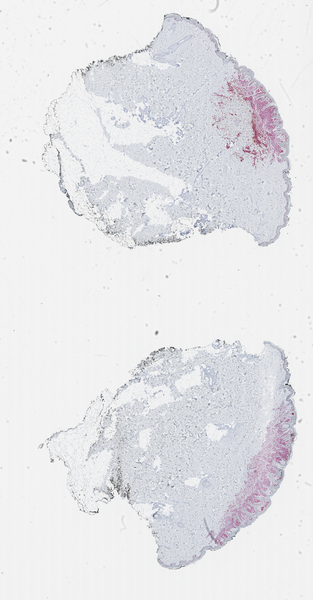

Supplement: S1 Dataset — (ZIP) [file pone.0297146.s007.zip › MelanA/531946_MelanA.png]

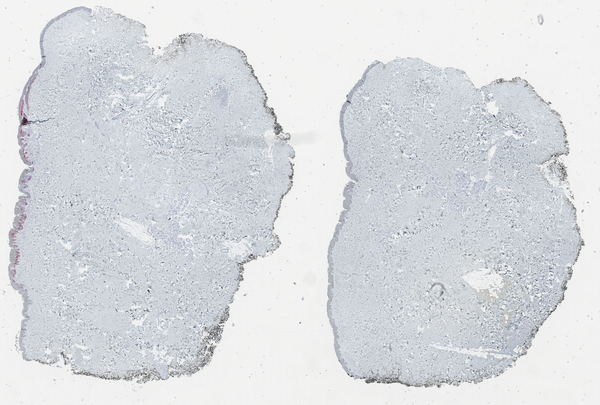

Supplement: S1 Dataset — (ZIP) [file pone.0297146.s007.zip › MelanA/473038_MelanA.png]

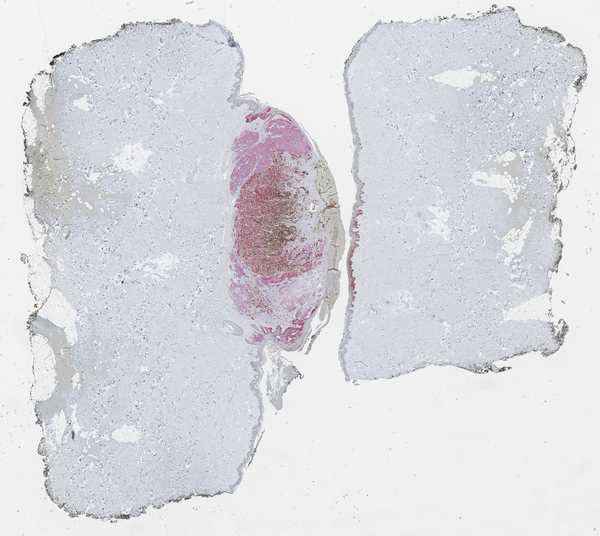

Supplement: S1 Dataset — (ZIP) [file pone.0297146.s007.zip › MelanA/303141_MelanA.png]

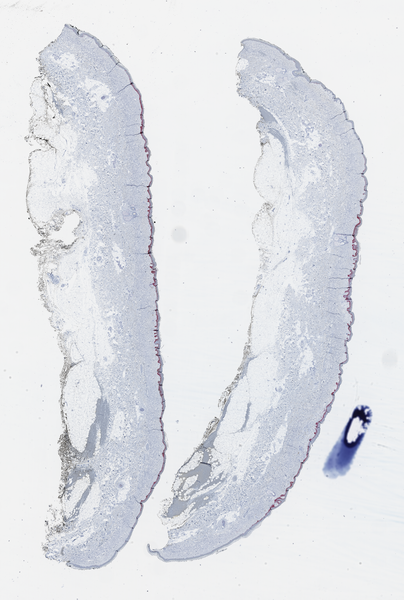

Supplement: S1 Dataset — (ZIP) [file pone.0297146.s007.zip › MelanA/608216_MelanA.png]

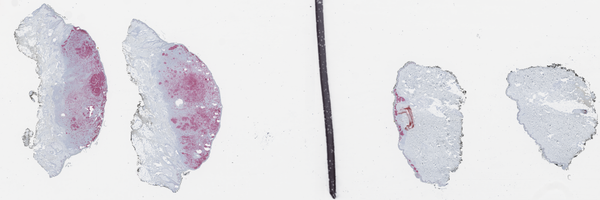

Supplement: S1 Dataset — (ZIP) [file pone.0297146.s007.zip › MelanA/853282-2_MelanA.png]

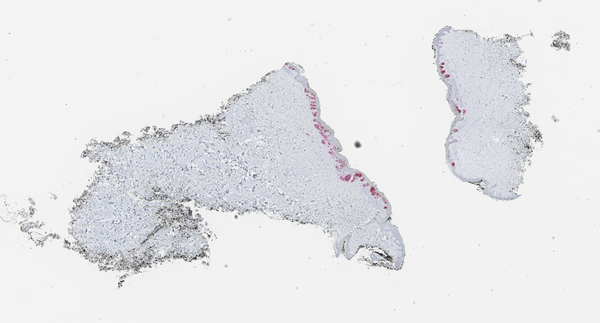

Supplement: S1 Dataset — (ZIP) [file pone.0297146.s007.zip › MelanA/409386-2_MelanA.png]

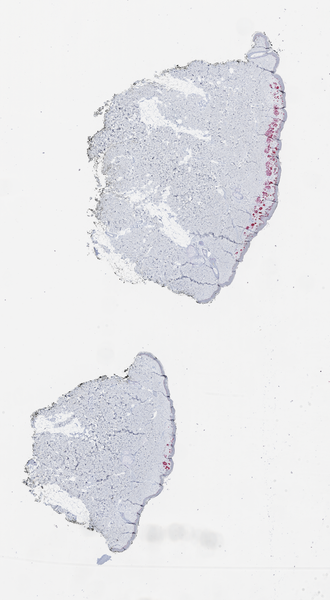

Supplement: S1 Dataset — (ZIP) [file pone.0297146.s007.zip › MelanA/446684_MelanA.png]

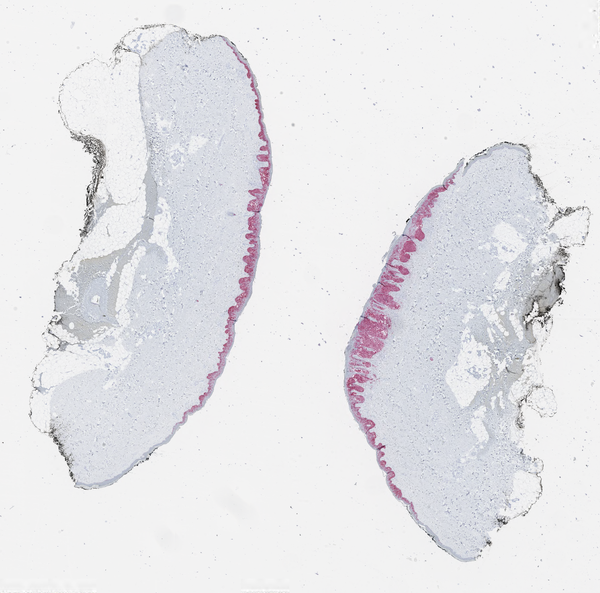

Supplement: S1 Dataset — (ZIP) [file pone.0297146.s007.zip › MelanA/849969-1_MelanA.png]

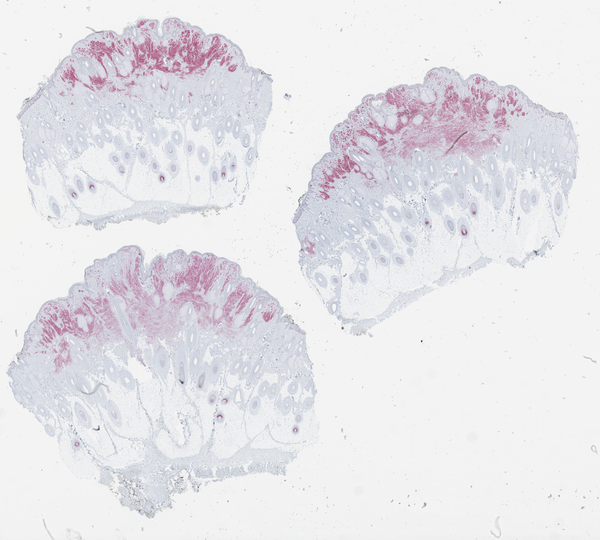

Supplement: S1 Dataset — (ZIP) [file pone.0297146.s007.zip › MelanA/691272_MelanA.png]

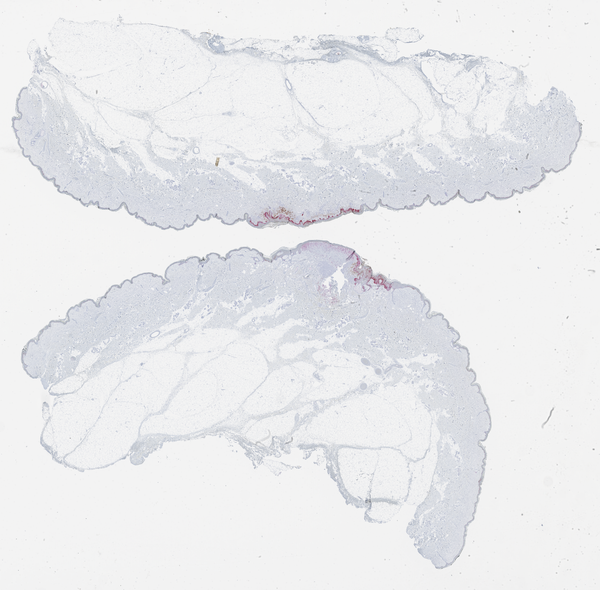

Supplement: S1 Dataset — (ZIP) [file pone.0297146.s007.zip › MelanA/643765_MelanA.png]

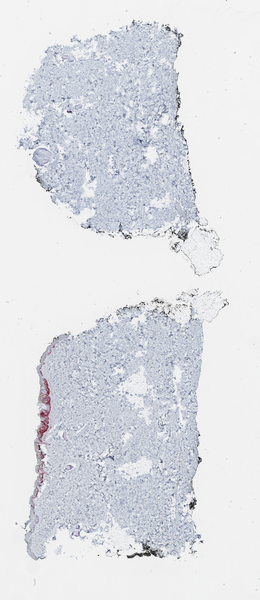

Supplement: S1 Dataset — (ZIP) [file pone.0297146.s007.zip › MelanA/570214_MelanA.png]

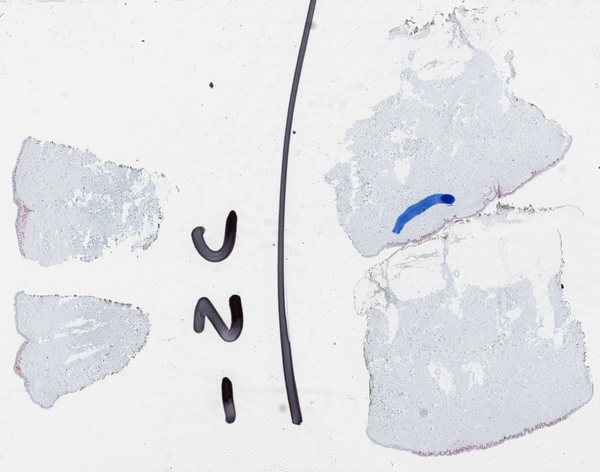

Supplement: S1 Dataset — (ZIP) [file pone.0297146.s007.zip › MelanA/401945-2_MelanA.png]

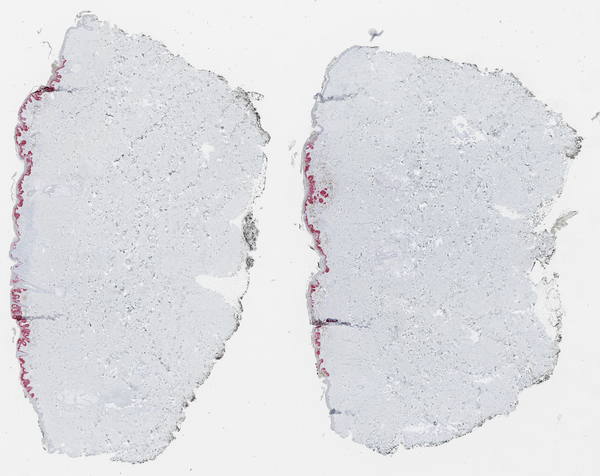

Supplement: S1 Dataset — (ZIP) [file pone.0297146.s007.zip › MelanA/213658_MelanA.png]

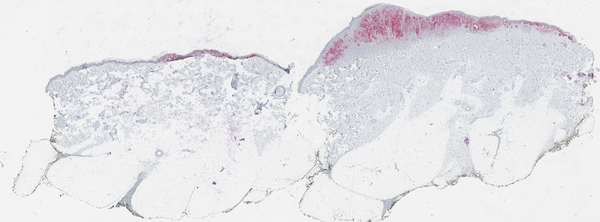

Supplement: S1 Dataset — (ZIP) [file pone.0297146.s007.zip › MelanA/250459_MelanA.png]

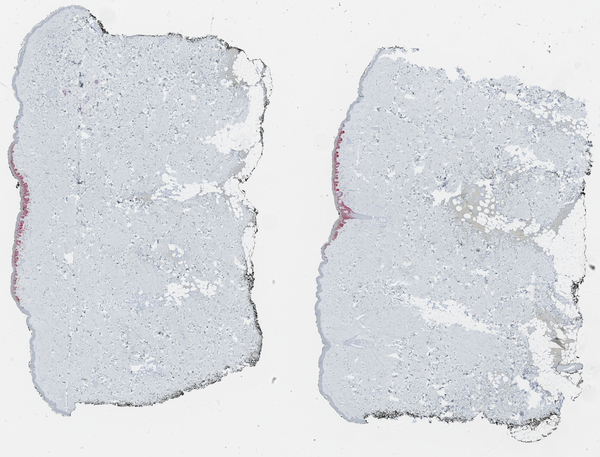

Supplement: S1 Dataset — (ZIP) [file pone.0297146.s007.zip › MelanA/247307_MelanA.png]

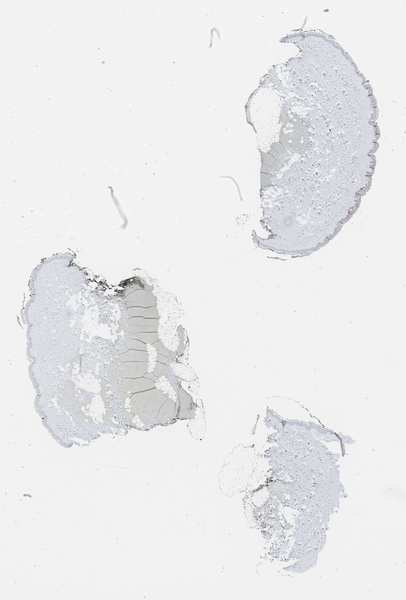

Supplement: S1 Dataset — (ZIP) [file pone.0297146.s007.zip › MelanA/792025_MelanA.png]

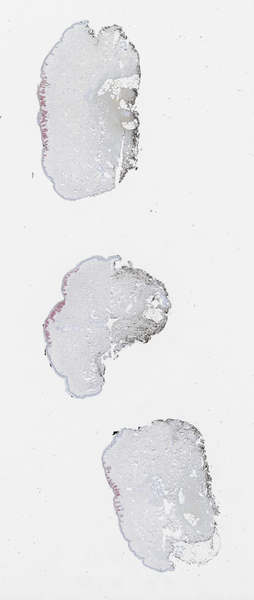

Supplement: S1 Dataset — (ZIP) [file pone.0297146.s007.zip › MelanA/470624_MelanA.png]
