# Supplementary figures and images for: Evaluating deep learning-based melanoma classification using immunohistochemistry and routine histology: A three center study (part 2 of 7)
Source: PLoS One. 2024 Jan 19;19(1):e0297146. doi: 10.1371/journal.pone.0297146 (PMC10798511; doi:10.1371/journal.pone.0297146)

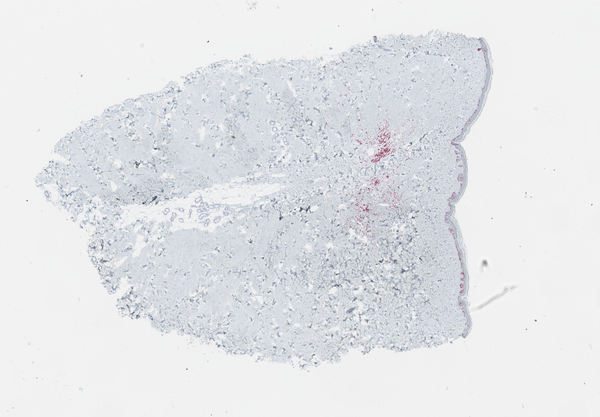

Supplement: S1 Dataset — (ZIP) [file pone.0297146.s007.zip › MelanA/233282_MelanA.png]

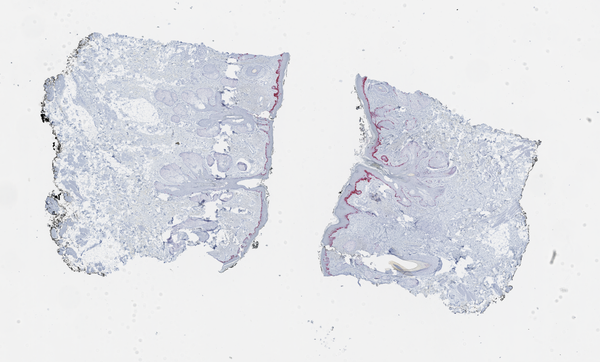

Supplement: S1 Dataset — (ZIP) [file pone.0297146.s007.zip › MelanA/559318_MelanA.png]

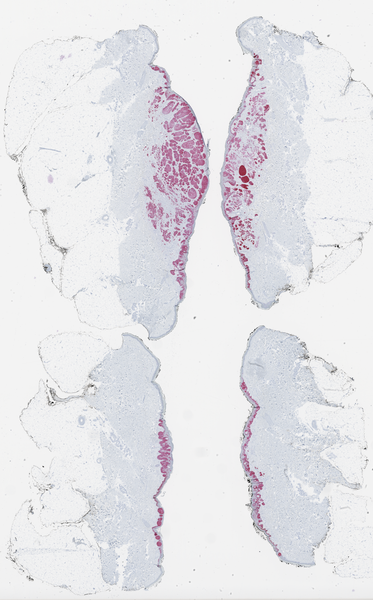

Supplement: S1 Dataset — (ZIP) [file pone.0297146.s007.zip › MelanA/257169_MelanA.png]

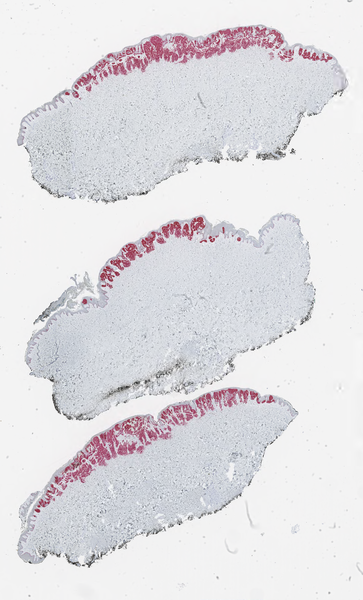

Supplement: S1 Dataset — (ZIP) [file pone.0297146.s007.zip › MelanA/764181_MelanA.png]

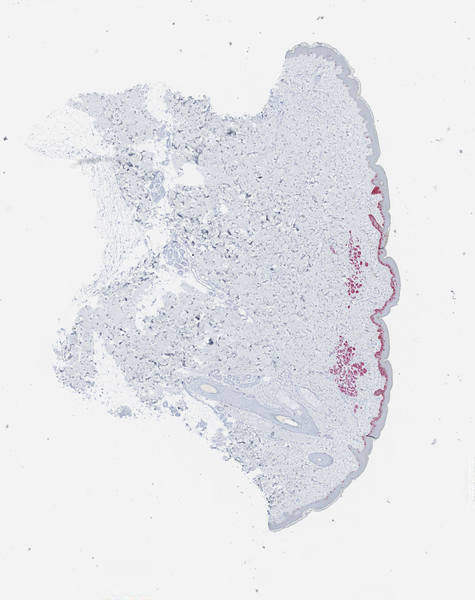

Supplement: S1 Dataset — (ZIP) [file pone.0297146.s007.zip › MelanA/119687_MelanA.png]

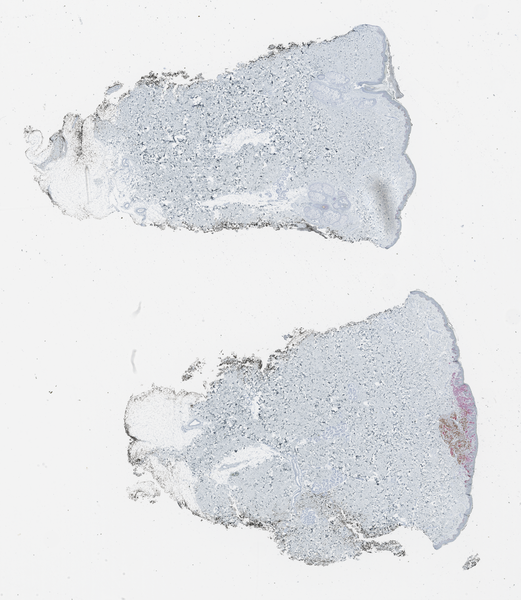

Supplement: S1 Dataset — (ZIP) [file pone.0297146.s007.zip › MelanA/308477_MelanA.png]

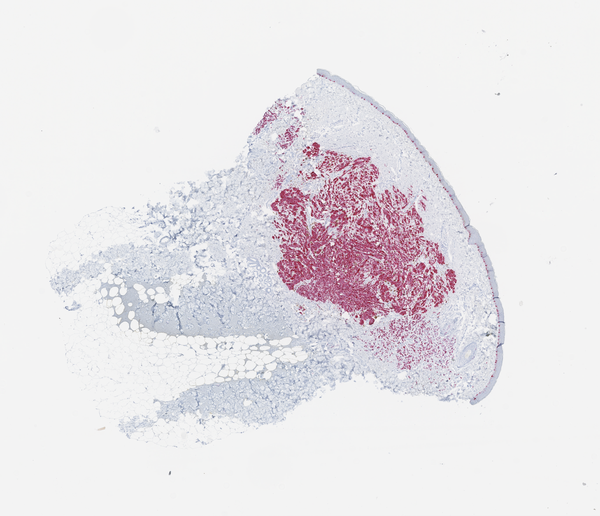

Supplement: S1 Dataset — (ZIP) [file pone.0297146.s007.zip › MelanA/196757_MelanA.png]

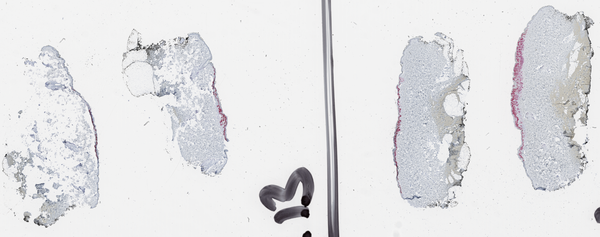

Supplement: S1 Dataset — (ZIP) [file pone.0297146.s007.zip › MelanA/232419-1_MelanA.png]

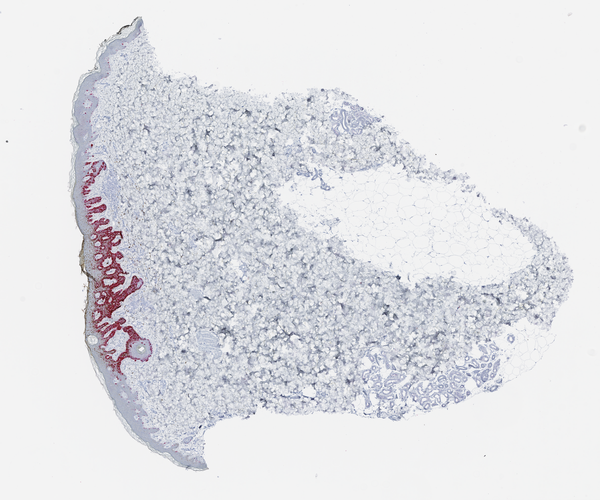

Supplement: S1 Dataset — (ZIP) [file pone.0297146.s007.zip › MelanA/662985_MelanA.png]

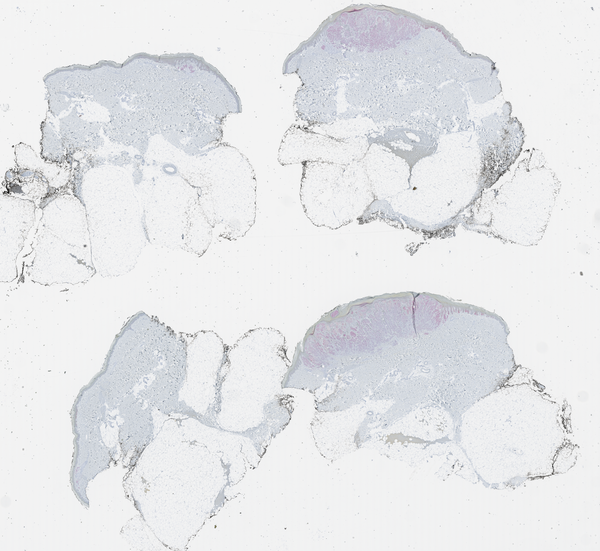

Supplement: S1 Dataset — (ZIP) [file pone.0297146.s007.zip › MelanA/289639_MelanA.png]

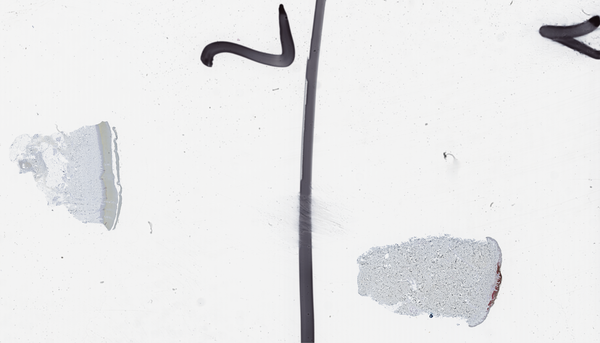

Supplement: S1 Dataset — (ZIP) [file pone.0297146.s007.zip › MelanA/573563-2_MelanA.png]

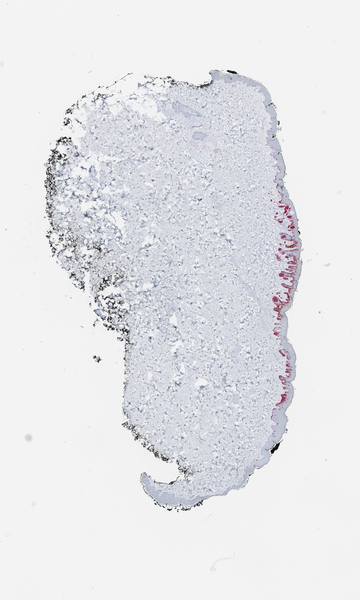

Supplement: S1 Dataset — (ZIP) [file pone.0297146.s007.zip › MelanA/849969-2_MelanA.png]

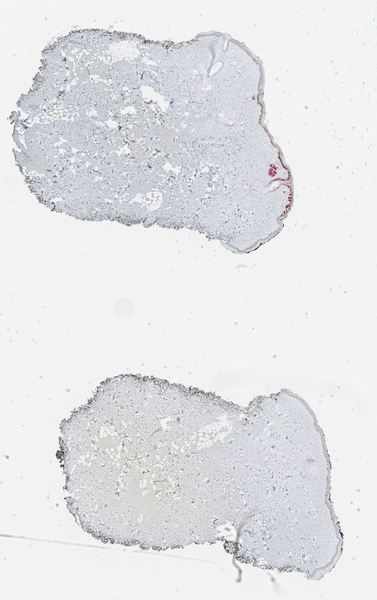

Supplement: S1 Dataset — (ZIP) [file pone.0297146.s007.zip › MelanA/652632_MelanA.png]

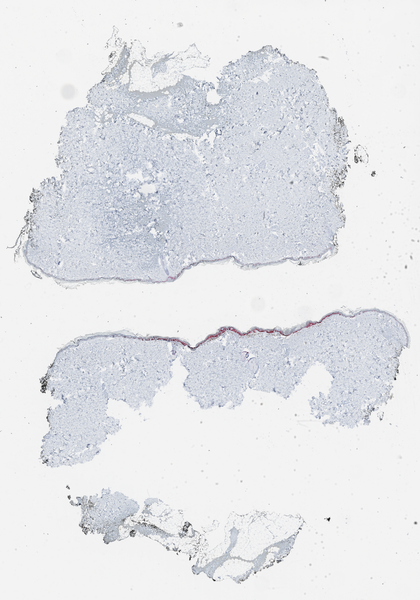

Supplement: S1 Dataset — (ZIP) [file pone.0297146.s007.zip › MelanA/413935_MelanA.png]

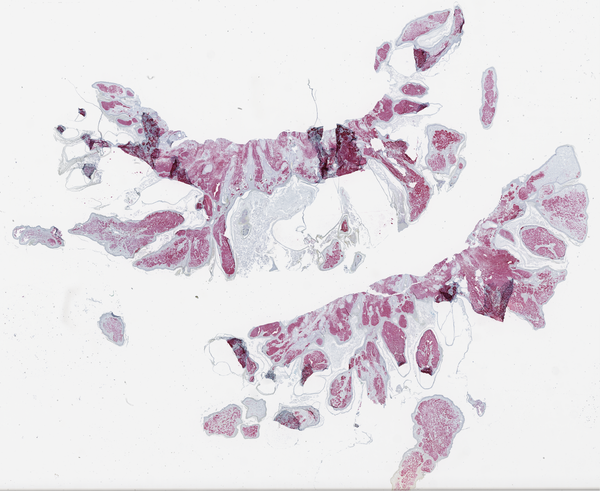

Supplement: S1 Dataset — (ZIP) [file pone.0297146.s007.zip › MelanA/414417-2_MelanA.png]

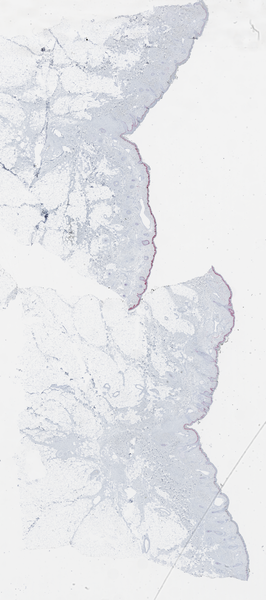

Supplement: S1 Dataset — (ZIP) [file pone.0297146.s007.zip › MelanA/639877_MelanA.png]

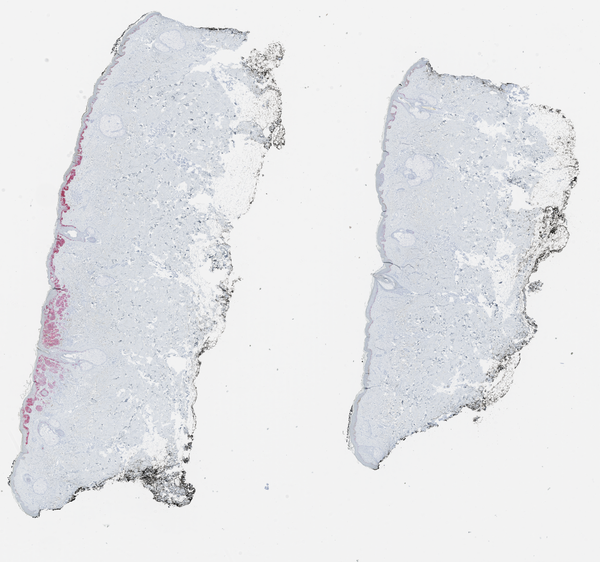

Supplement: S1 Dataset — (ZIP) [file pone.0297146.s007.zip › MelanA/216647_MelanA.png]

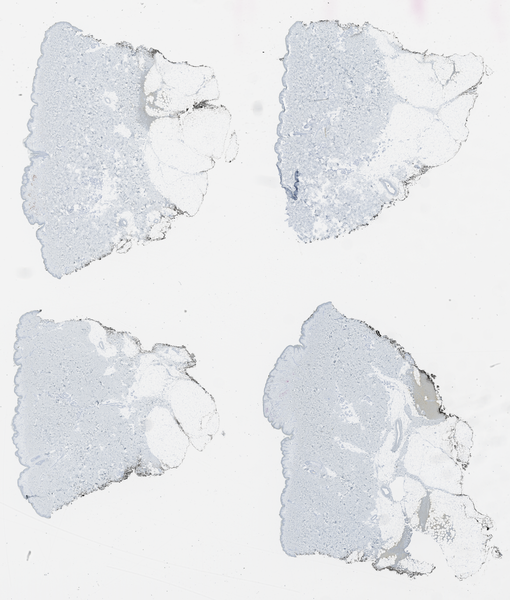

Supplement: S1 Dataset — (ZIP) [file pone.0297146.s007.zip › MelanA/285234_MelanA.png]

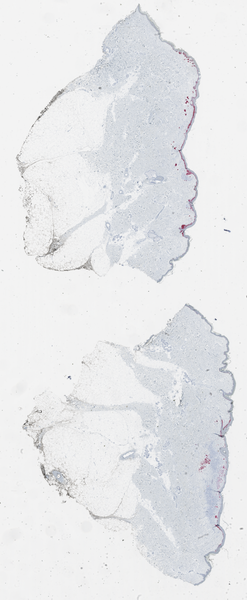

Supplement: S1 Dataset — (ZIP) [file pone.0297146.s007.zip › MelanA/228762_MelanA.png]

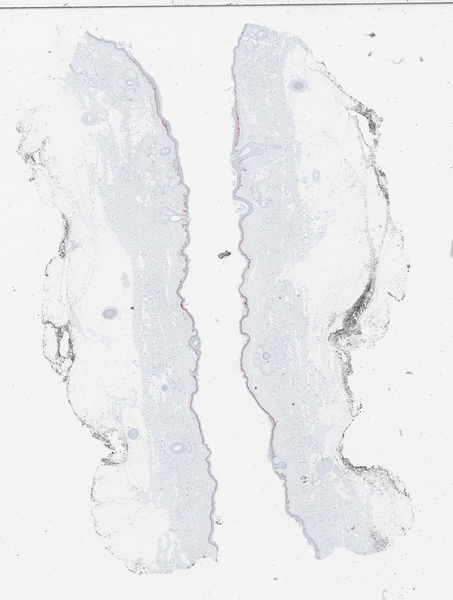

Supplement: S1 Dataset — (ZIP) [file pone.0297146.s007.zip › MelanA/717314_MelanA.png]

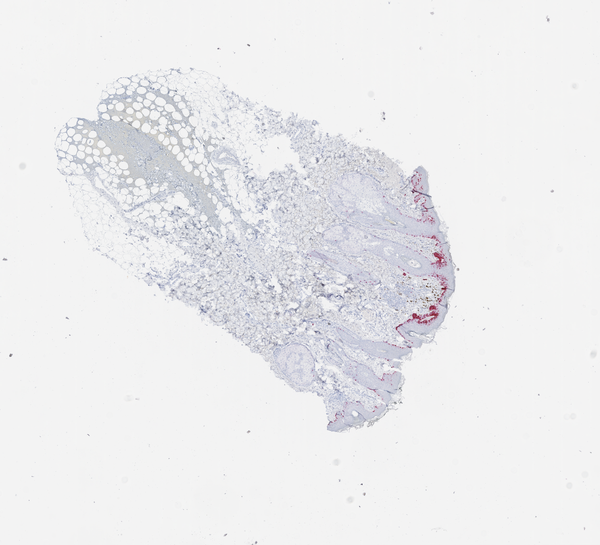

Supplement: S1 Dataset — (ZIP) [file pone.0297146.s007.zip › MelanA/638990_MelanA.png]

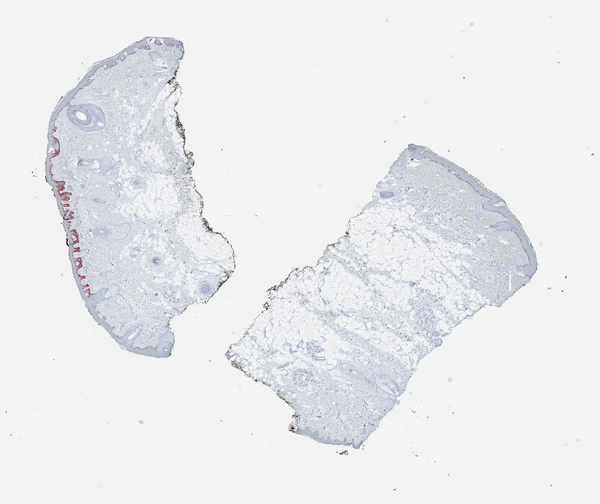

Supplement: S1 Dataset — (ZIP) [file pone.0297146.s007.zip › MelanA/830638_MelanA.png]

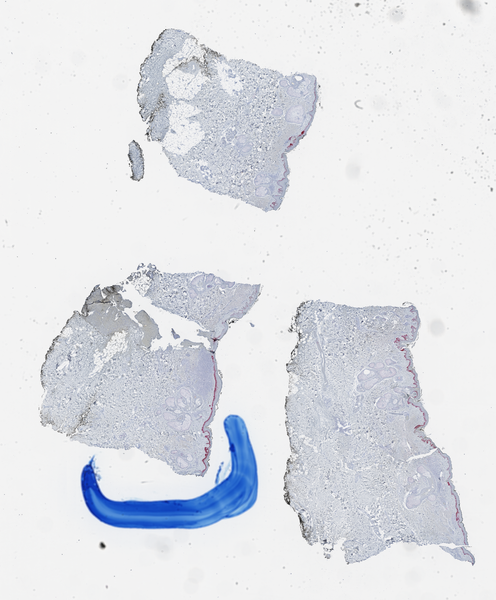

Supplement: S1 Dataset — (ZIP) [file pone.0297146.s007.zip › MelanA/525132-1_MelanA.png]

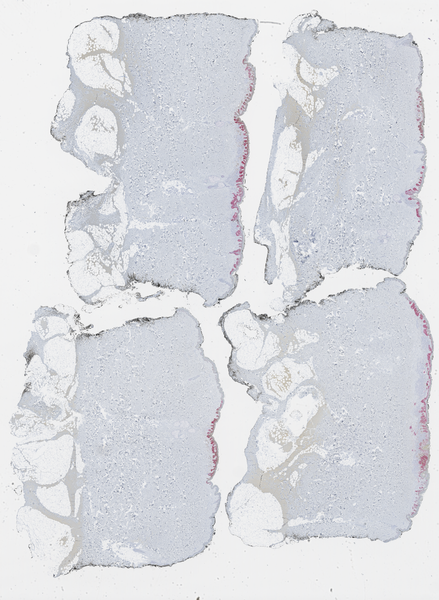

Supplement: S1 Dataset — (ZIP) [file pone.0297146.s007.zip › MelanA/515966_MelanA.png]

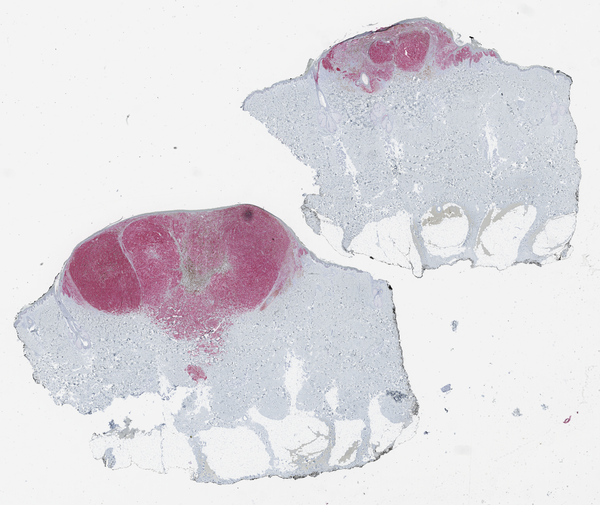

Supplement: S1 Dataset — (ZIP) [file pone.0297146.s007.zip › MelanA/222180_MelanA.png]

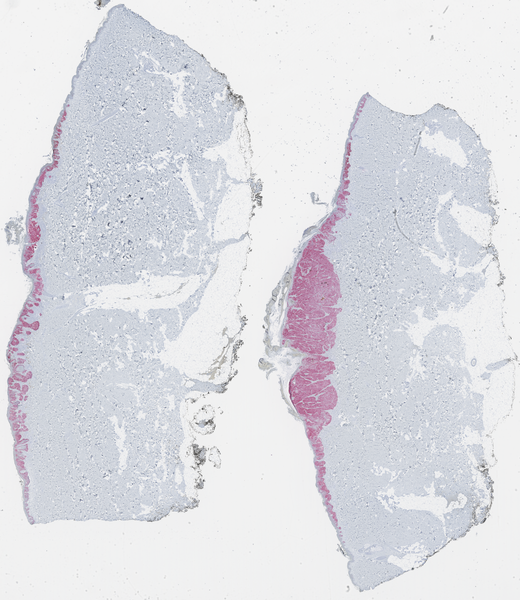

Supplement: S1 Dataset — (ZIP) [file pone.0297146.s007.zip › MelanA/408086_MelanA.png]

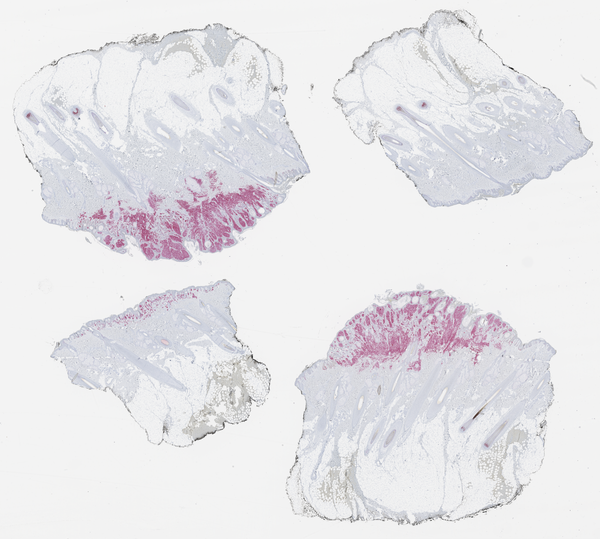

Supplement: S1 Dataset — (ZIP) [file pone.0297146.s007.zip › MelanA/546569-1_MelanA.png]

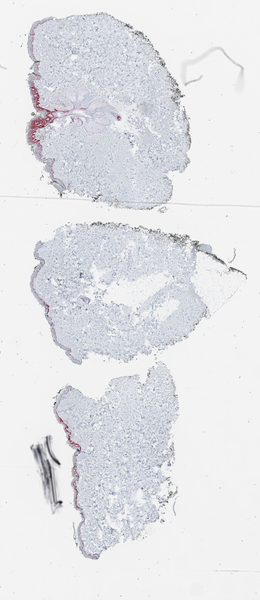

Supplement: S1 Dataset — (ZIP) [file pone.0297146.s007.zip › MelanA/113308_MelanA.png]

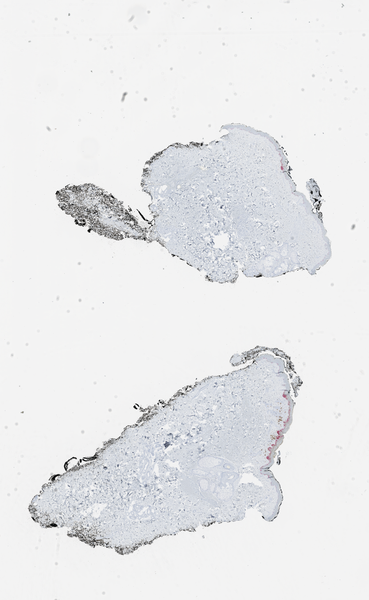

Supplement: S1 Dataset — (ZIP) [file pone.0297146.s007.zip › MelanA/200197_MelanA.png]

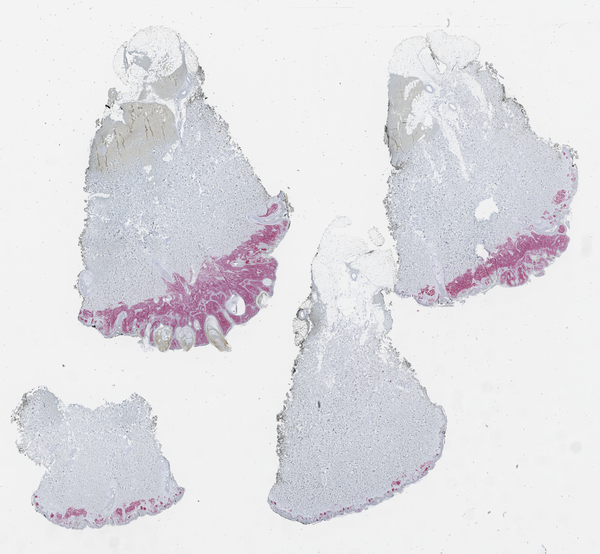

Supplement: S1 Dataset — (ZIP) [file pone.0297146.s007.zip › MelanA/200427-1_MelanA.png]

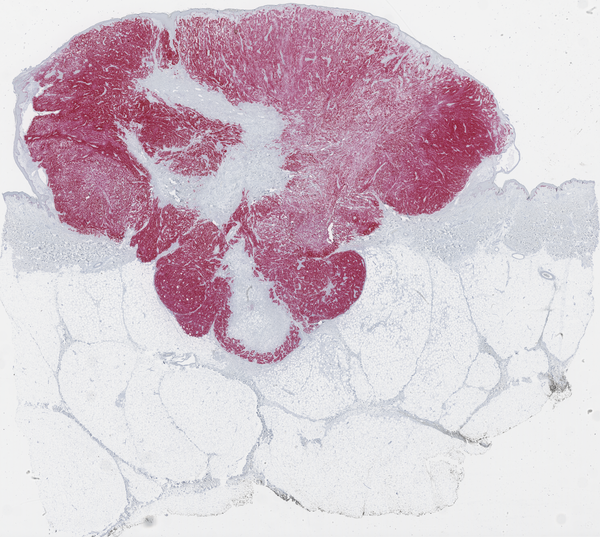

Supplement: S1 Dataset — (ZIP) [file pone.0297146.s007.zip › MelanA/237476_MelanA.png]

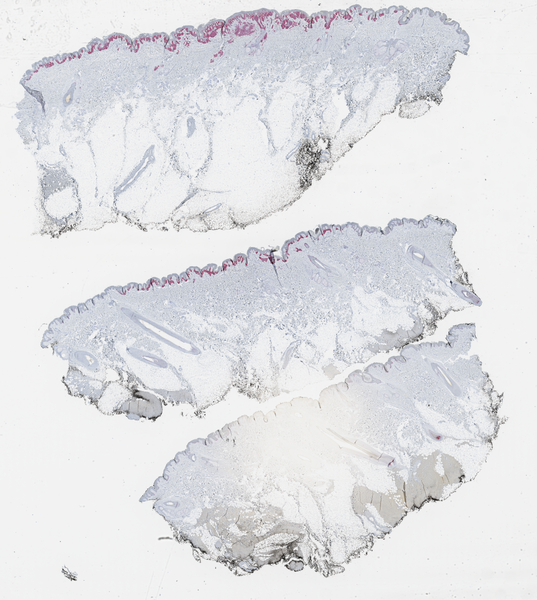

Supplement: S1 Dataset — (ZIP) [file pone.0297146.s007.zip › MelanA/486741_MelanA.png]

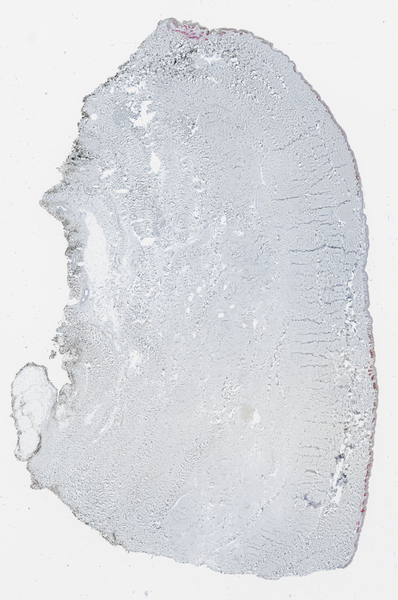

Supplement: S1 Dataset — (ZIP) [file pone.0297146.s007.zip › MelanA/763152_MelanA.png]

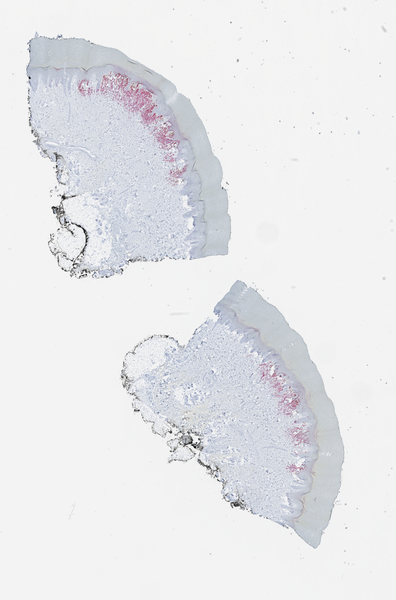

Supplement: S1 Dataset — (ZIP) [file pone.0297146.s007.zip › MelanA/450912-1_MelanA.png]

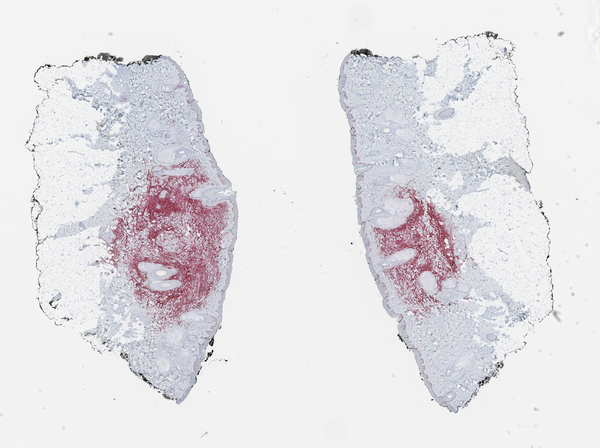

Supplement: S1 Dataset — (ZIP) [file pone.0297146.s007.zip › MelanA/842825_MelanA.png]

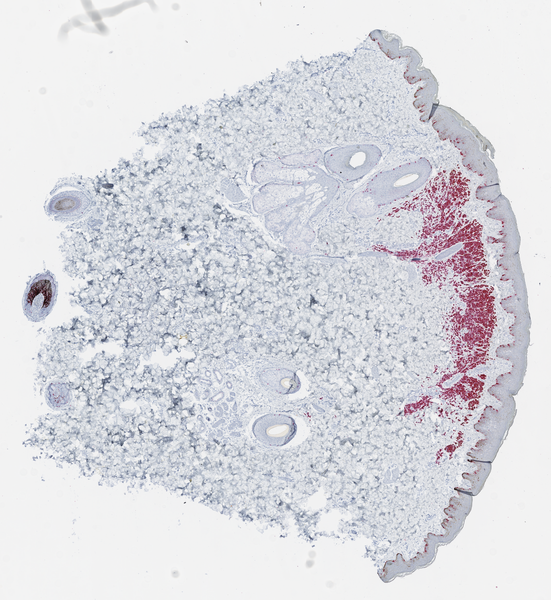

Supplement: S1 Dataset — (ZIP) [file pone.0297146.s007.zip › MelanA/105229_MelanA.png]

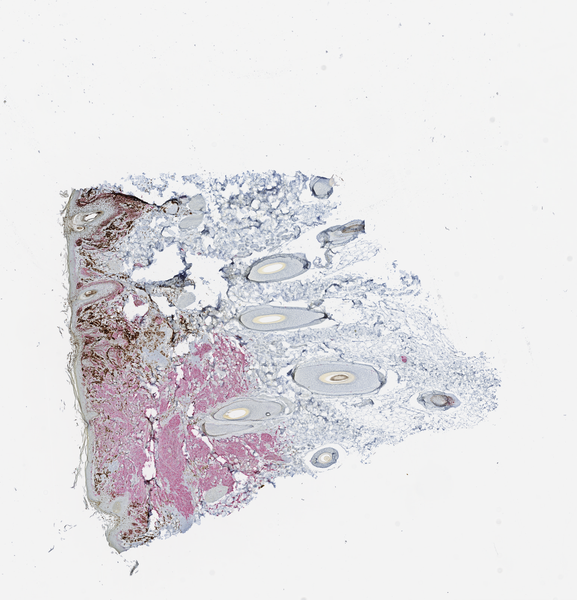

Supplement: S1 Dataset — (ZIP) [file pone.0297146.s007.zip › MelanA/783370_MelanA.png]

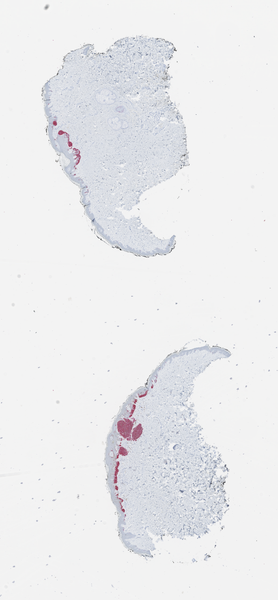

Supplement: S1 Dataset — (ZIP) [file pone.0297146.s007.zip › MelanA/716005_MelanA.png]

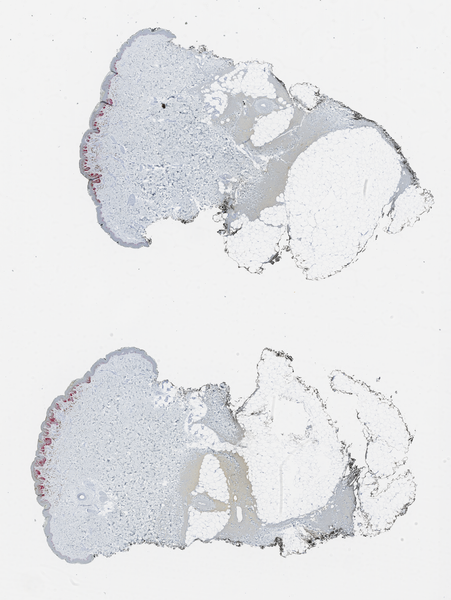

Supplement: S1 Dataset — (ZIP) [file pone.0297146.s007.zip › MelanA/515829_MelanA.png]

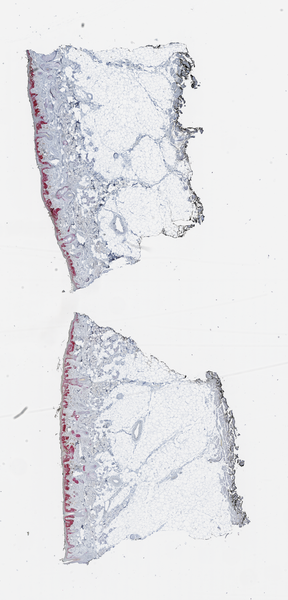

Supplement: S1 Dataset — (ZIP) [file pone.0297146.s007.zip › MelanA/679218_MelanA.png]

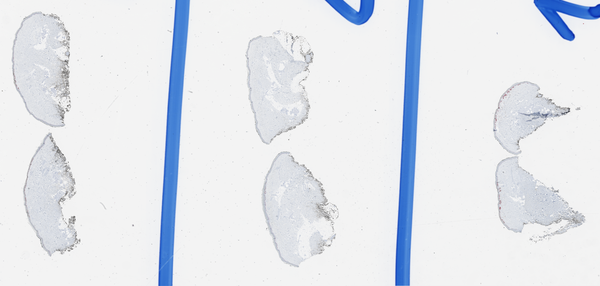

Supplement: S1 Dataset — (ZIP) [file pone.0297146.s007.zip › MelanA/726303-1_MelanA.png]

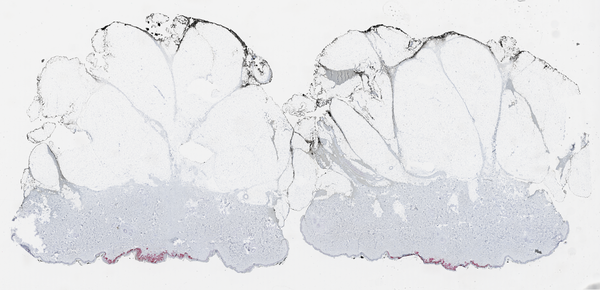

Supplement: S1 Dataset — (ZIP) [file pone.0297146.s007.zip › MelanA/626414_MelanA.png]

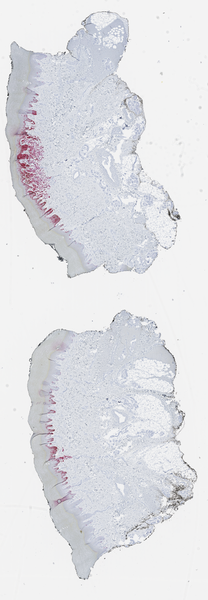

Supplement: S1 Dataset — (ZIP) [file pone.0297146.s007.zip › MelanA/676277_MelanA.png]

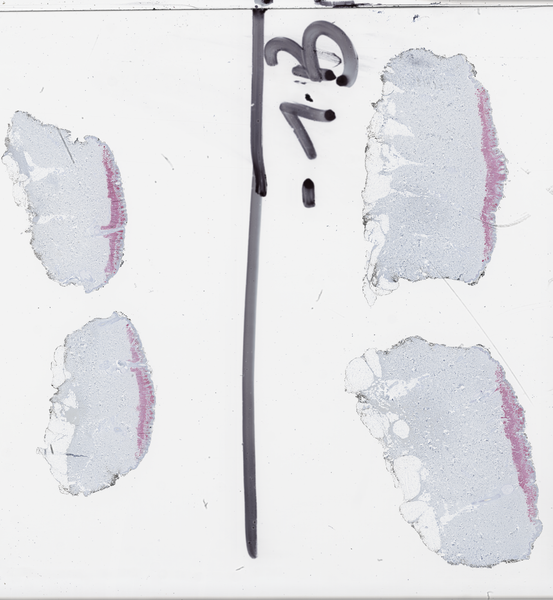

Supplement: S1 Dataset — (ZIP) [file pone.0297146.s007.zip › MelanA/729980-2_MelanA.png]

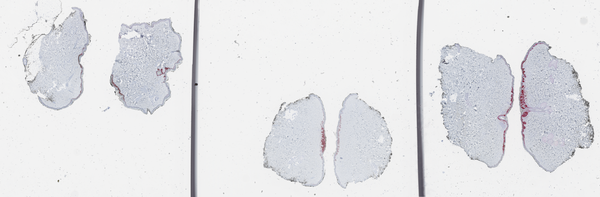

Supplement: S1 Dataset — (ZIP) [file pone.0297146.s007.zip › MelanA/665087-2_MelanA.png]

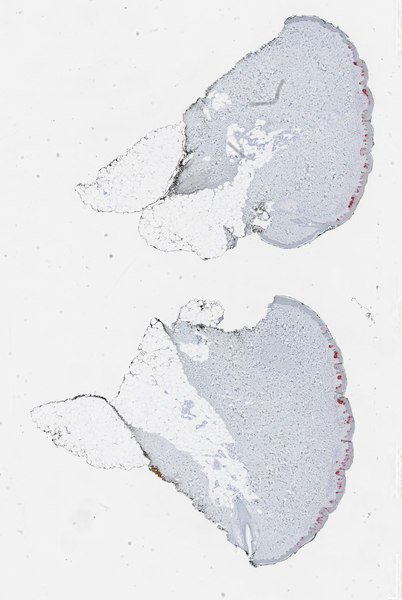

Supplement: S1 Dataset — (ZIP) [file pone.0297146.s007.zip › MelanA/335663_MelanA.png]

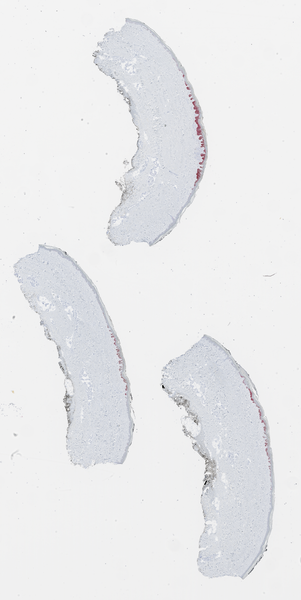

Supplement: S1 Dataset — (ZIP) [file pone.0297146.s007.zip › MelanA/542754-1_MelanA.png]

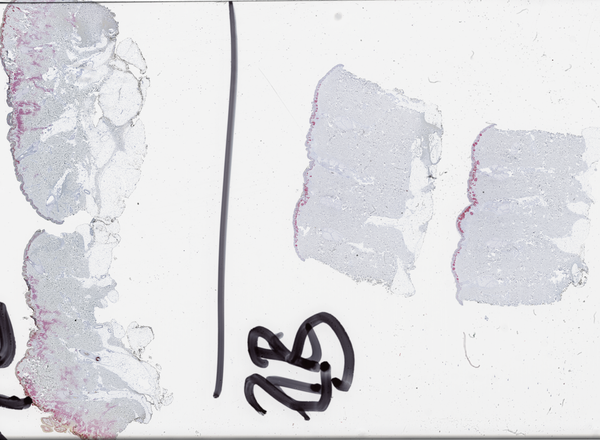

Supplement: S1 Dataset — (ZIP) [file pone.0297146.s007.zip › MelanA/676708-1_MelanA.png]

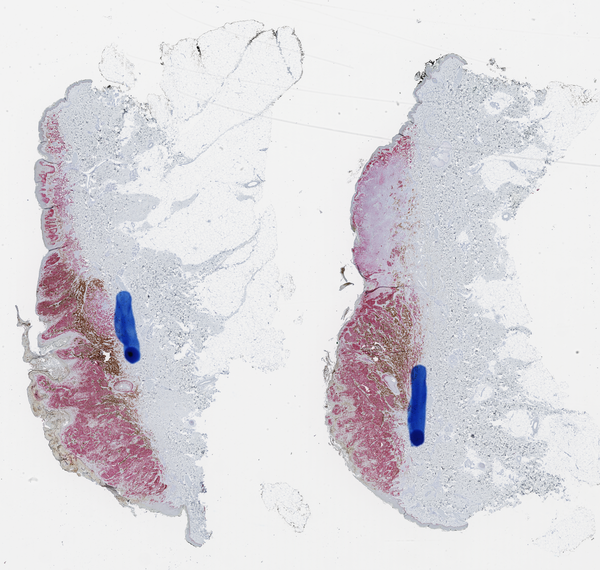

Supplement: S1 Dataset — (ZIP) [file pone.0297146.s007.zip › MelanA/753457_MelanA.png]

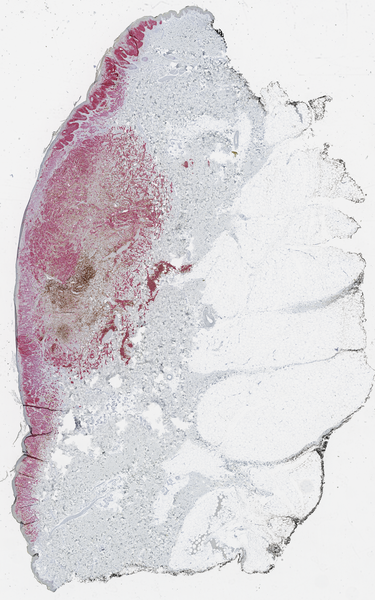

Supplement: S1 Dataset — (ZIP) [file pone.0297146.s007.zip › MelanA/205260_MelanA.png]

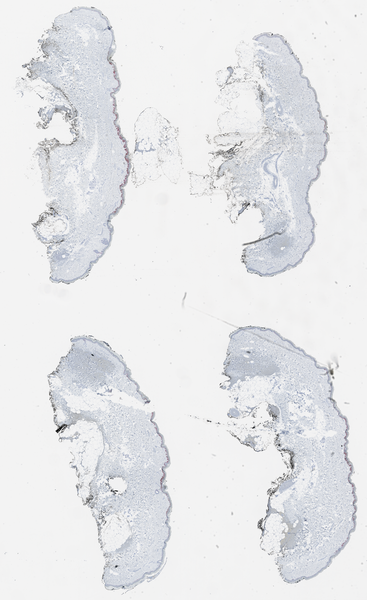

Supplement: S1 Dataset — (ZIP) [file pone.0297146.s007.zip › MelanA/495868_MelanA.png]

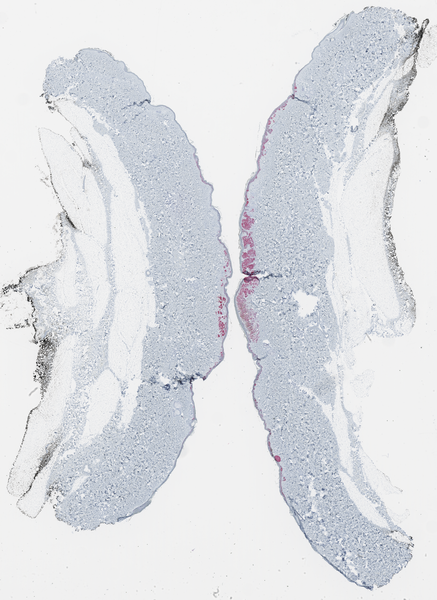

Supplement: S1 Dataset — (ZIP) [file pone.0297146.s007.zip › MelanA/260415_MelanA.png]

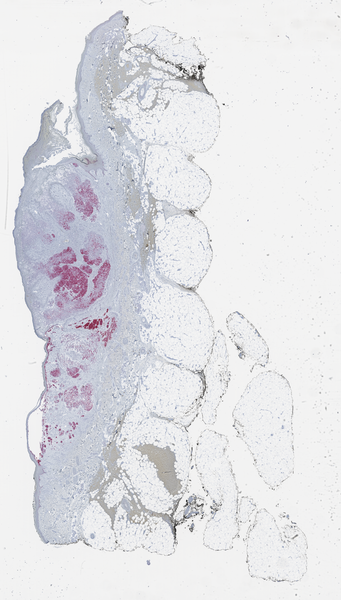

Supplement: S1 Dataset — (ZIP) [file pone.0297146.s007.zip › MelanA/618720_MelanA.png]

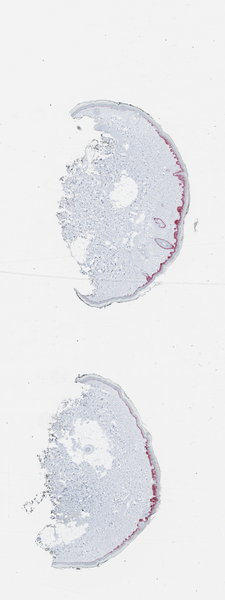

Supplement: S1 Dataset — (ZIP) [file pone.0297146.s007.zip › MelanA/698154_MelanA.png]

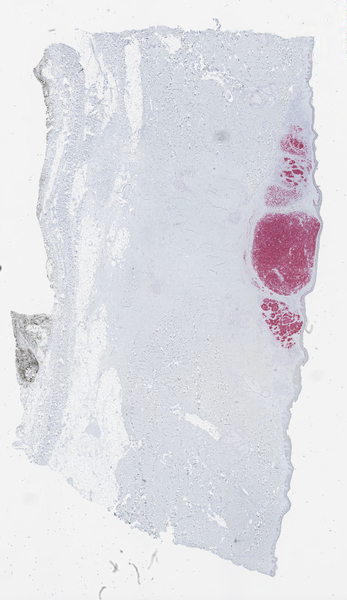

Supplement: S1 Dataset — (ZIP) [file pone.0297146.s007.zip › MelanA/826016_MelanA.png]

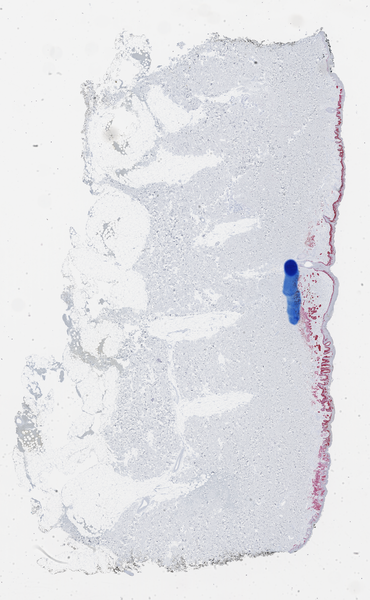

Supplement: S1 Dataset — (ZIP) [file pone.0297146.s007.zip › MelanA/703576_MelanA.png]

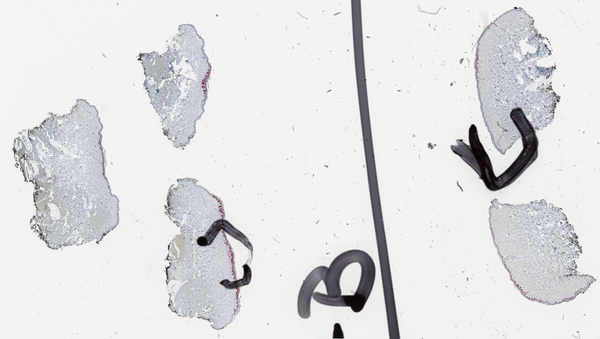

Supplement: S1 Dataset — (ZIP) [file pone.0297146.s007.zip › MelanA/668403-1_MelanA.png]

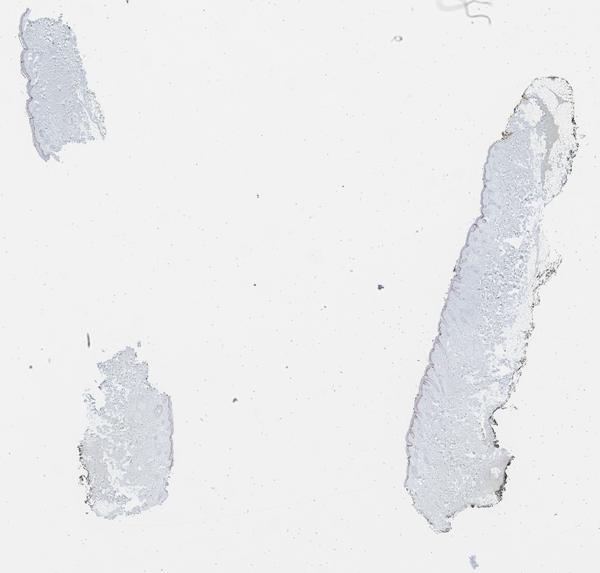

Supplement: S1 Dataset — (ZIP) [file pone.0297146.s007.zip › MelanA/782692_MelanA.png]

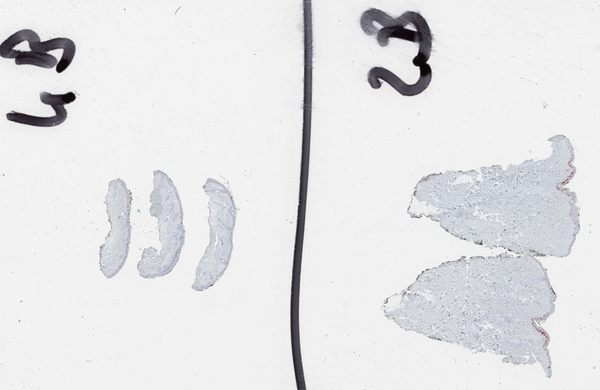

Supplement: S1 Dataset — (ZIP) [file pone.0297146.s007.zip › MelanA/563033-1_MelanA.png]

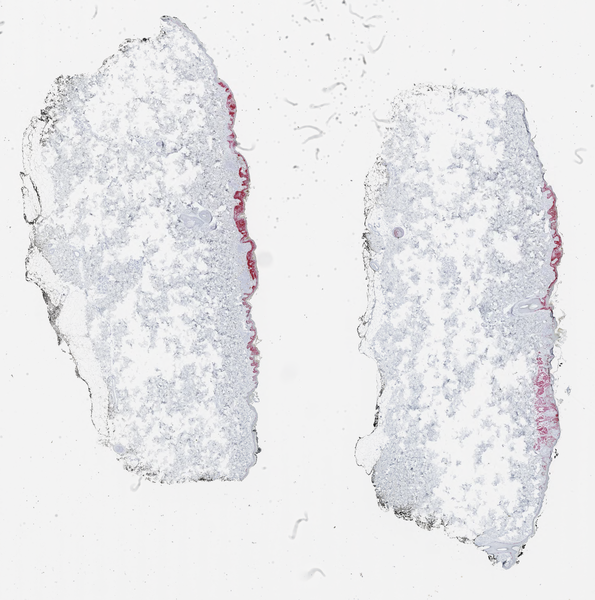

Supplement: S1 Dataset — (ZIP) [file pone.0297146.s007.zip › MelanA/813888_MelanA.png]

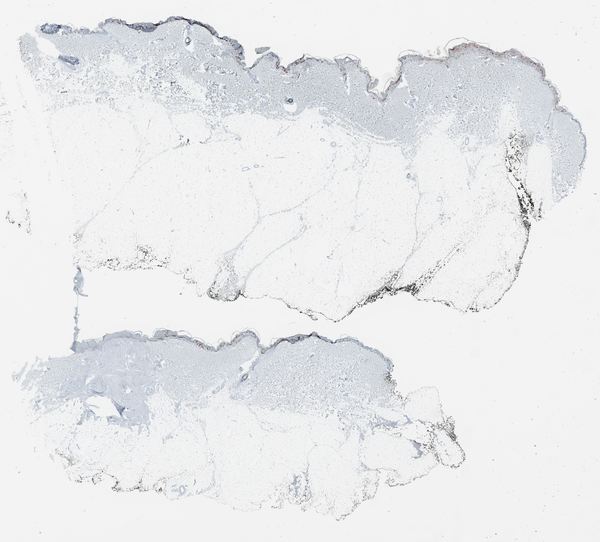

Supplement: S1 Dataset — (ZIP) [file pone.0297146.s007.zip › MelanA/287913_MelanA.png]

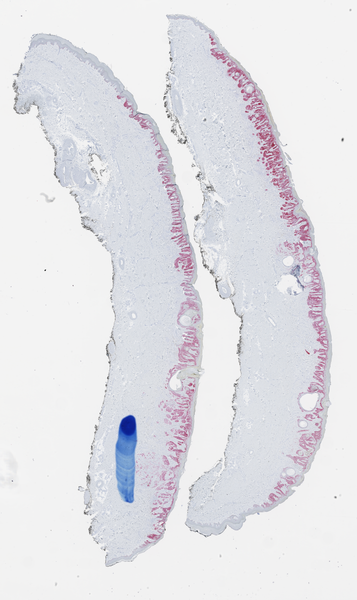

Supplement: S1 Dataset — (ZIP) [file pone.0297146.s007.zip › MelanA/768250_MelanA.png]

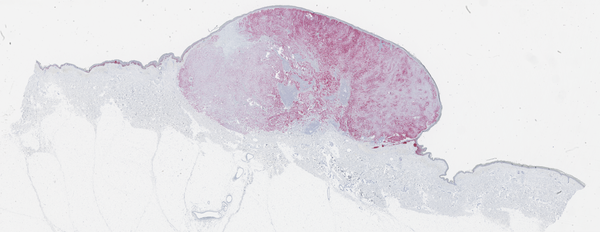

Supplement: S1 Dataset — (ZIP) [file pone.0297146.s007.zip › MelanA/558024_MelanA.png]

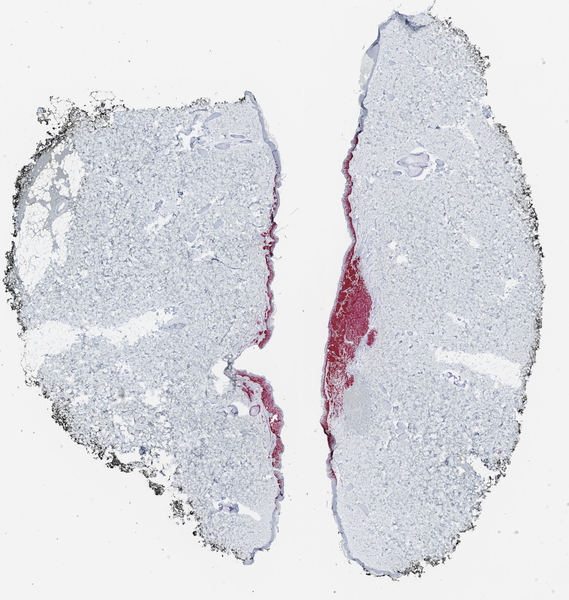

Supplement: S1 Dataset — (ZIP) [file pone.0297146.s007.zip › MelanA/672910-1_MelanA.png]

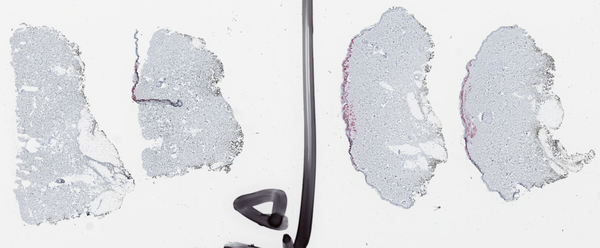

Supplement: S1 Dataset — (ZIP) [file pone.0297146.s007.zip › MelanA/232419-2_MelanA.png]

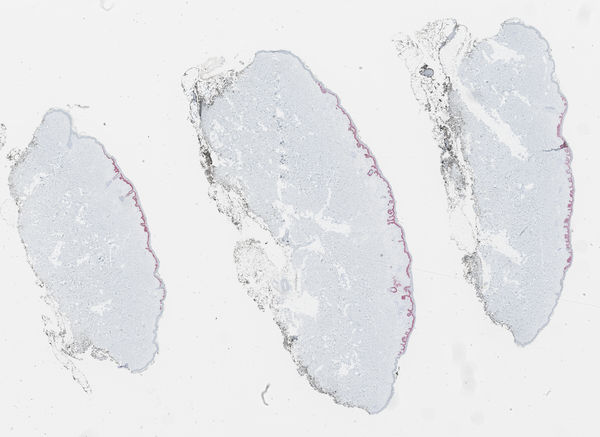

Supplement: S1 Dataset — (ZIP) [file pone.0297146.s007.zip › MelanA/745192-2_MelanA.png]

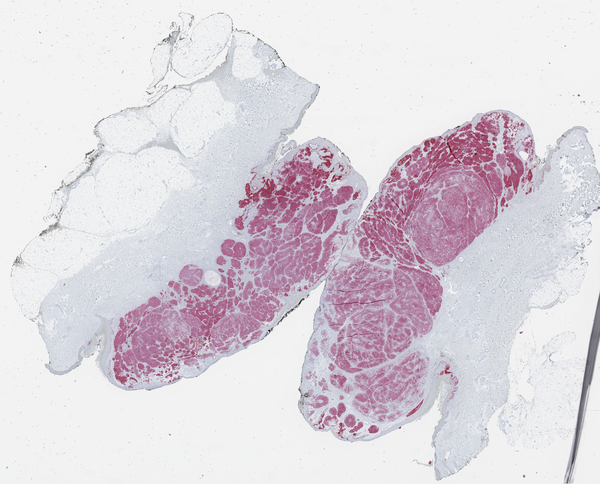

Supplement: S1 Dataset — (ZIP) [file pone.0297146.s007.zip › MelanA/101292_MelanA.png]

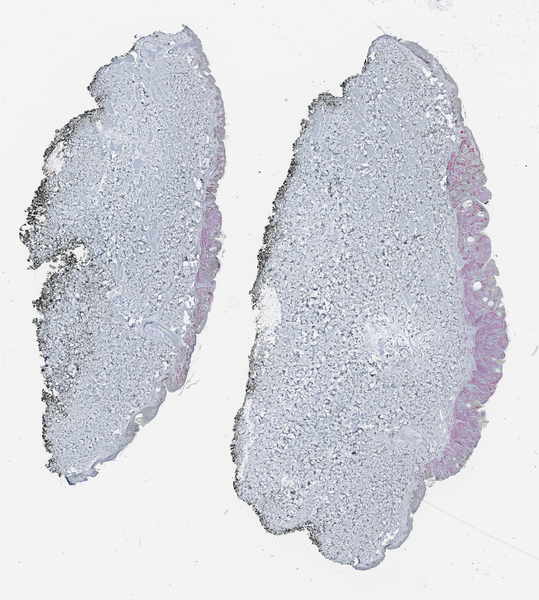

Supplement: S1 Dataset — (ZIP) [file pone.0297146.s007.zip › MelanA/278485-1_MelanA.png]

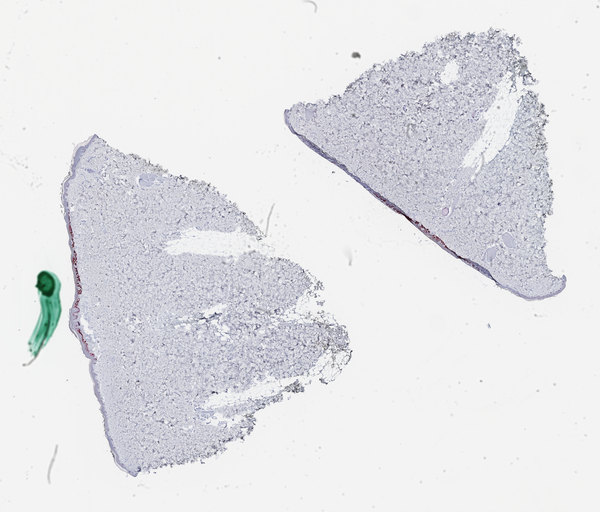

Supplement: S1 Dataset — (ZIP) [file pone.0297146.s007.zip › MelanA/642570_MelanA.png]

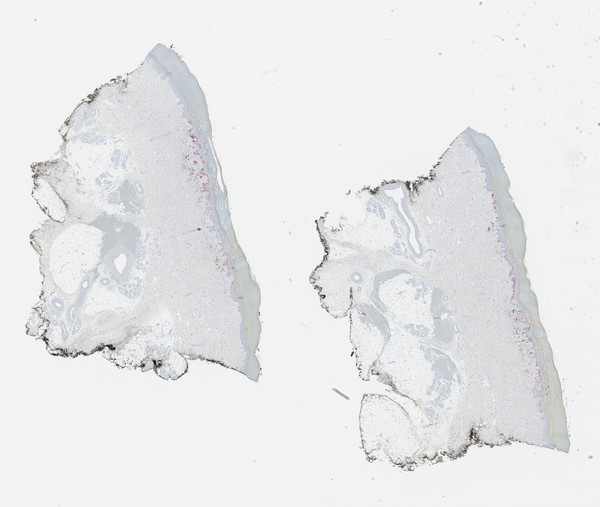

Supplement: S1 Dataset — (ZIP) [file pone.0297146.s007.zip › MelanA/238717_MelanA.png]

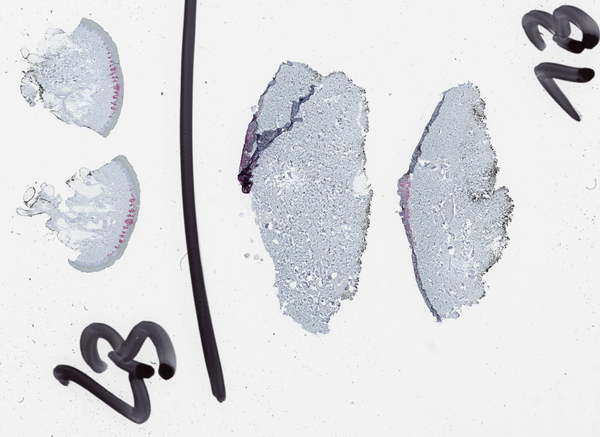

Supplement: S1 Dataset — (ZIP) [file pone.0297146.s007.zip › MelanA/278485-2_MelanA.png]

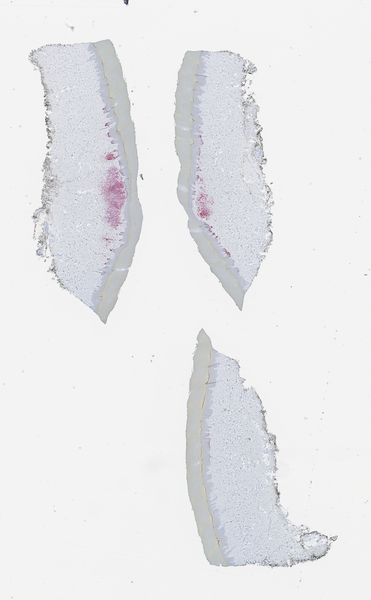

Supplement: S1 Dataset — (ZIP) [file pone.0297146.s007.zip › MelanA/724056_MelanA.png]

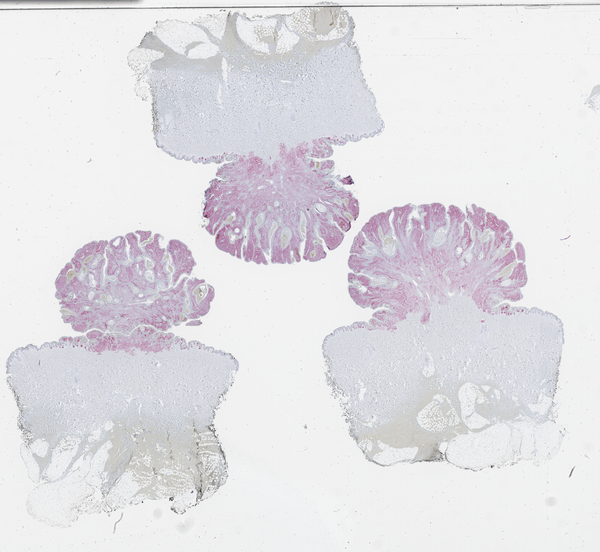

Supplement: S1 Dataset — (ZIP) [file pone.0297146.s007.zip › MelanA/200427-2_MelanA.png]

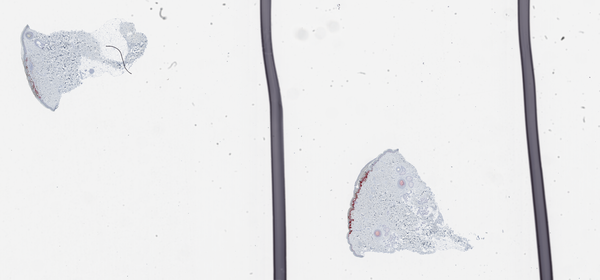

Supplement: S1 Dataset — (ZIP) [file pone.0297146.s007.zip › MelanA/652414-2_MelanA.png]

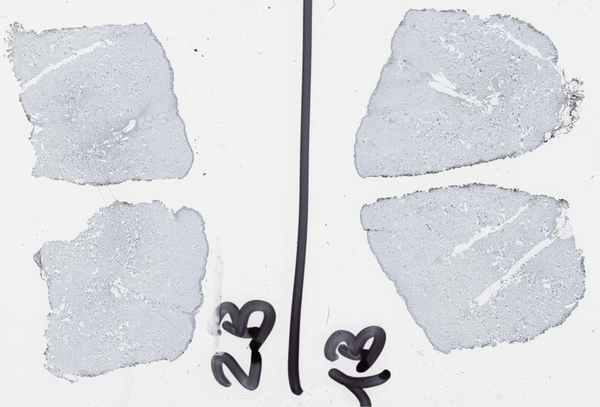

Supplement: S1 Dataset — (ZIP) [file pone.0297146.s007.zip › MelanA/465986-2_MelanA.png]

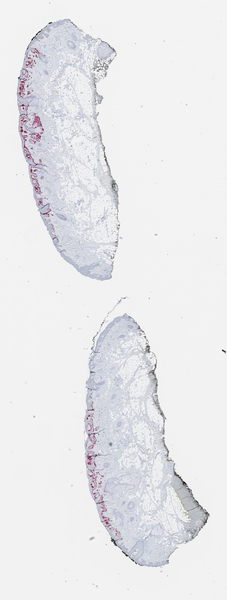

Supplement: S1 Dataset — (ZIP) [file pone.0297146.s007.zip › MelanA/599560-2_MelanA.png]

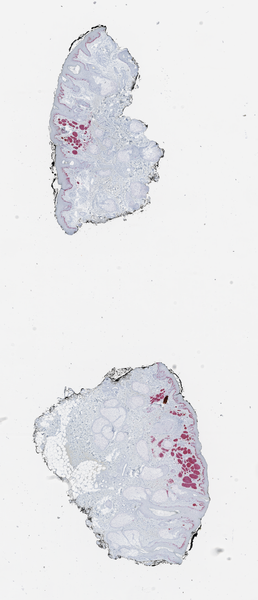

Supplement: S1 Dataset — (ZIP) [file pone.0297146.s007.zip › MelanA/109256_MelanA.png]

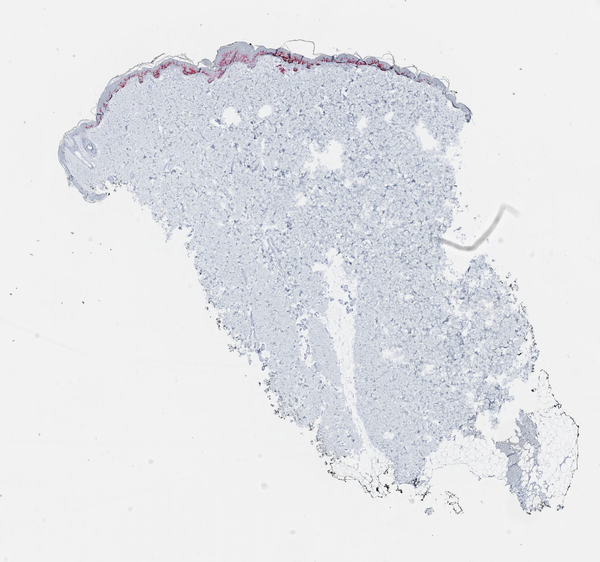

Supplement: S1 Dataset — (ZIP) [file pone.0297146.s007.zip › MelanA/570315_MelanA.png]

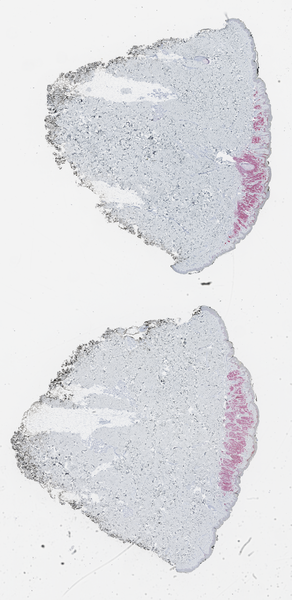

Supplement: S1 Dataset — (ZIP) [file pone.0297146.s007.zip › MelanA/224028_MelanA.png]

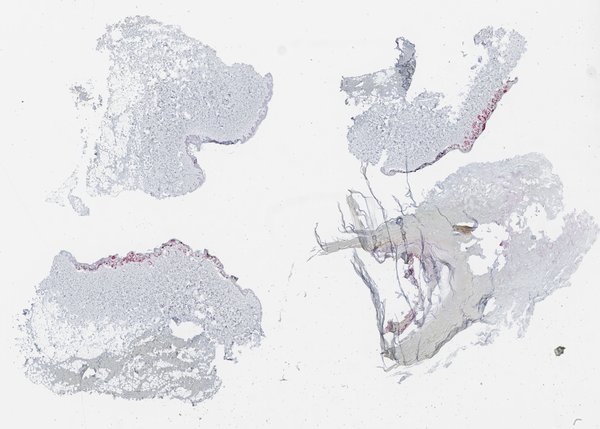

Supplement: S1 Dataset — (ZIP) [file pone.0297146.s007.zip › MelanA/646146_MelanA.png]

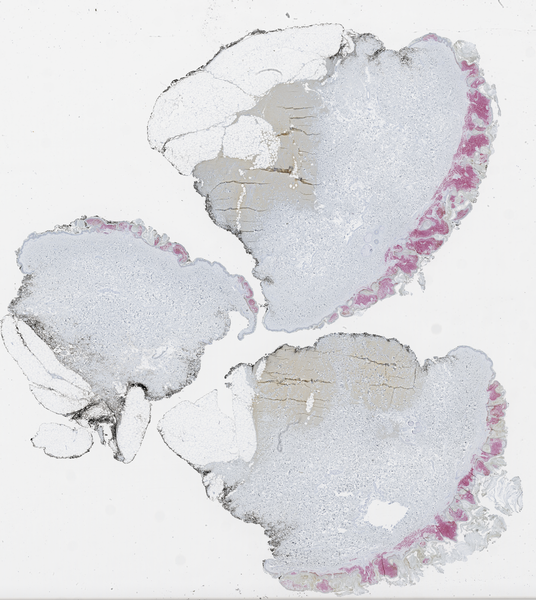

Supplement: S1 Dataset — (ZIP) [file pone.0297146.s007.zip › MelanA/414417-1_MelanA.png]

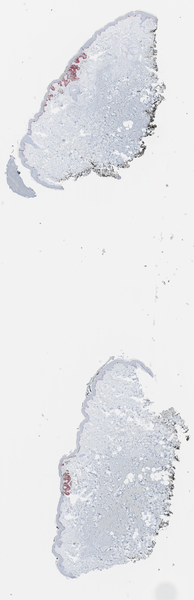

Supplement: S1 Dataset — (ZIP) [file pone.0297146.s007.zip › MelanA/653604_MelanA.png]

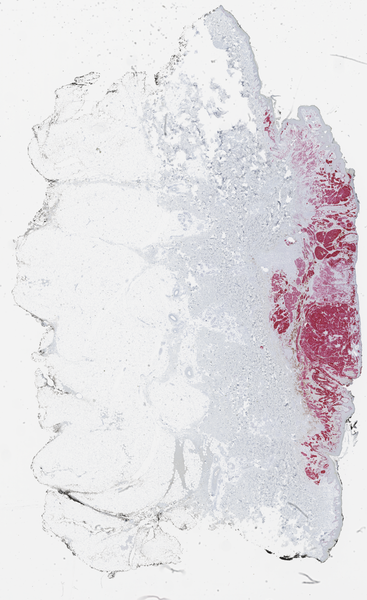

Supplement: S1 Dataset — (ZIP) [file pone.0297146.s007.zip › MelanA/827832_MelanA.png]

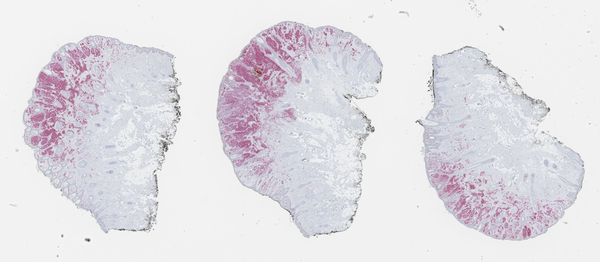

Supplement: S1 Dataset — (ZIP) [file pone.0297146.s007.zip › MelanA/700800_MelanA.png]

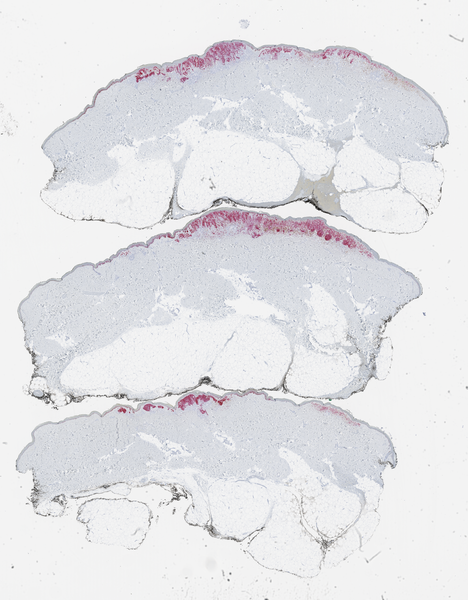

Supplement: S1 Dataset — (ZIP) [file pone.0297146.s007.zip › MelanA/521912_MelanA.png]

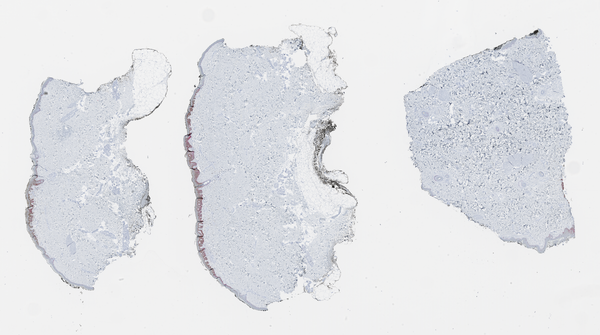

Supplement: S1 Dataset — (ZIP) [file pone.0297146.s007.zip › MelanA/716389_MelanA.png]

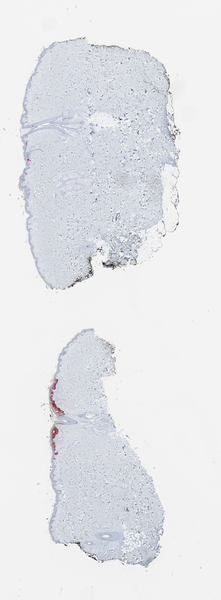

Supplement: S1 Dataset — (ZIP) [file pone.0297146.s007.zip › MelanA/759437_MelanA.png]

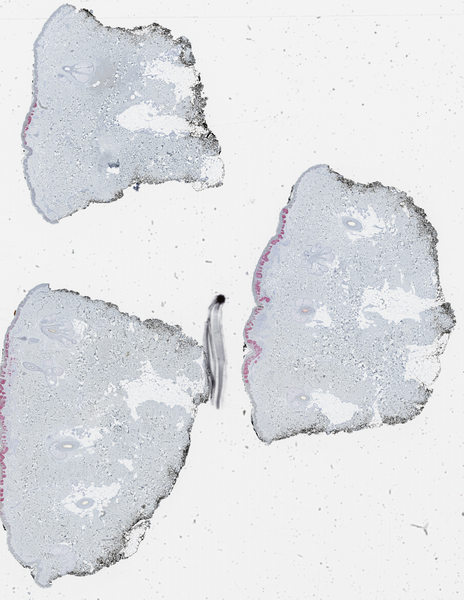

Supplement: S1 Dataset — (ZIP) [file pone.0297146.s007.zip › MelanA/259985_MelanA.png]

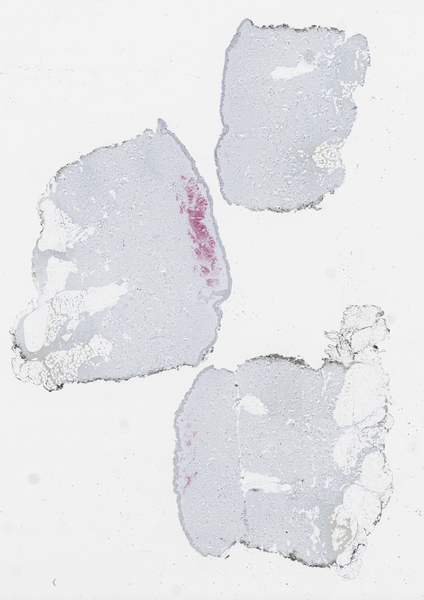

Supplement: S1 Dataset — (ZIP) [file pone.0297146.s007.zip › MelanA/549760_MelanA.png]

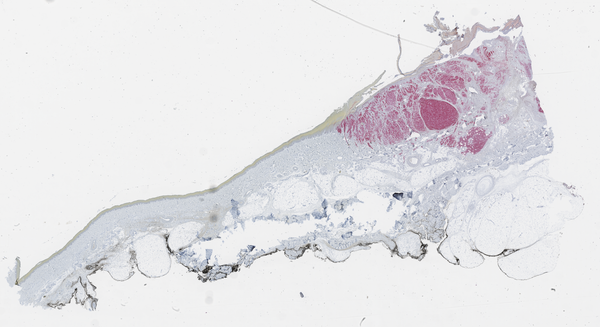

Supplement: S1 Dataset — (ZIP) [file pone.0297146.s007.zip › MelanA/787884_MelanA.png]

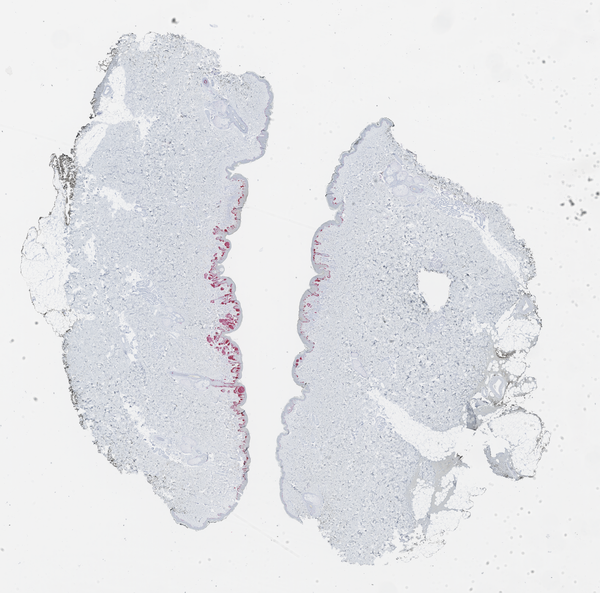

Supplement: S1 Dataset — (ZIP) [file pone.0297146.s007.zip › MelanA/619807-1_MelanA.png]

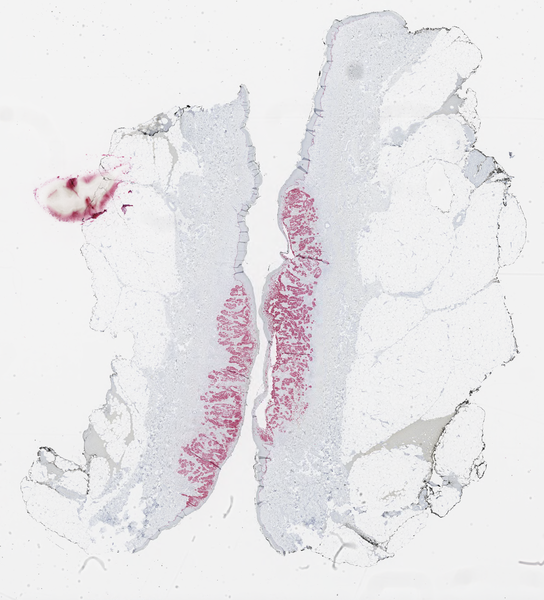

Supplement: S1 Dataset — (ZIP) [file pone.0297146.s007.zip › MelanA/800231_MelanA.png]

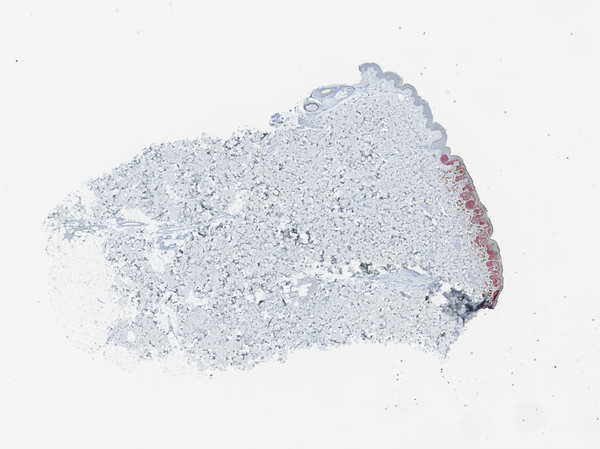

Supplement: S1 Dataset — (ZIP) [file pone.0297146.s007.zip › MelanA/318580_MelanA.png]

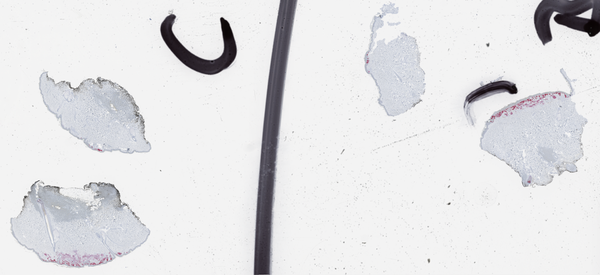

Supplement: S1 Dataset — (ZIP) [file pone.0297146.s007.zip › MelanA/408057_MelanA.png]

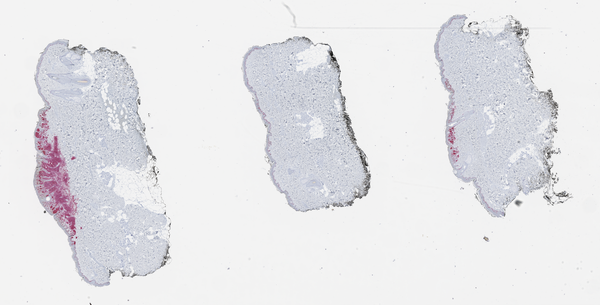

Supplement: S1 Dataset — (ZIP) [file pone.0297146.s007.zip › MelanA/243474_MelanA.png]

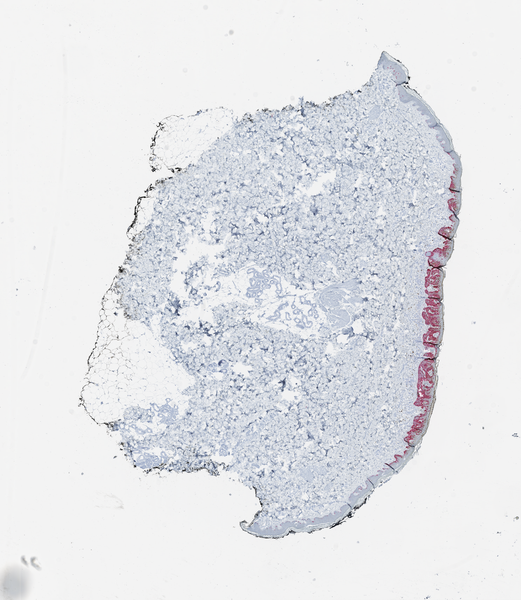

Supplement: S1 Dataset — (ZIP) [file pone.0297146.s007.zip › MelanA/417366_MelanA.png]

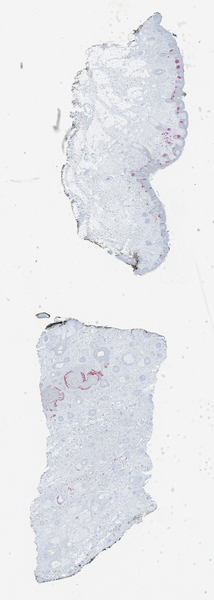

Supplement: S1 Dataset — (ZIP) [file pone.0297146.s007.zip › MelanA/499846_MelanA.png]

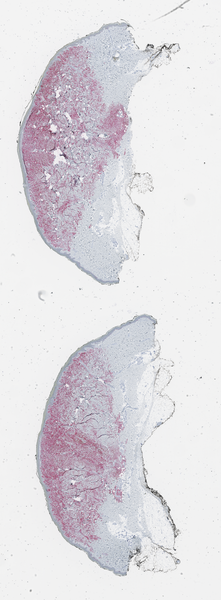

Supplement: S1 Dataset — (ZIP) [file pone.0297146.s007.zip › MelanA/231847_MelanA.png]

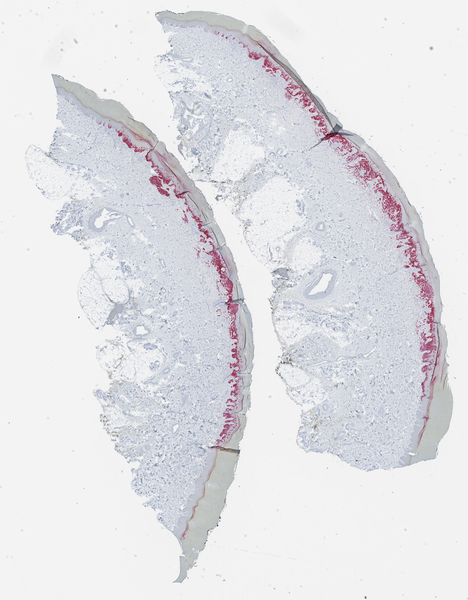

Supplement: S1 Dataset — (ZIP) [file pone.0297146.s007.zip › MelanA/673151_MelanA.png]

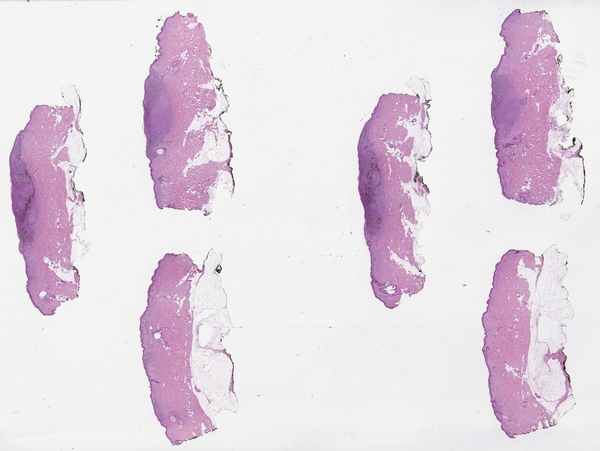

Supplement: S2 Dataset — (ZIP) [file pone.0297146.s008.zip › HE/522500_HE.png]
