# Supplementary figures and images for: Evaluating deep learning-based melanoma classification using immunohistochemistry and routine histology: A three center study (part 4 of 7)
Source: PLoS One. 2024 Jan 19;19(1):e0297146. doi: 10.1371/journal.pone.0297146 (PMC10798511; doi:10.1371/journal.pone.0297146)

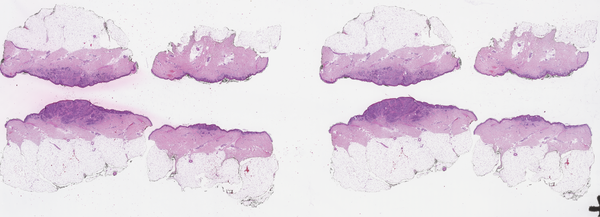

Supplement: S2 Dataset — (ZIP) [file pone.0297146.s008.zip › HE/257169_HE.png]

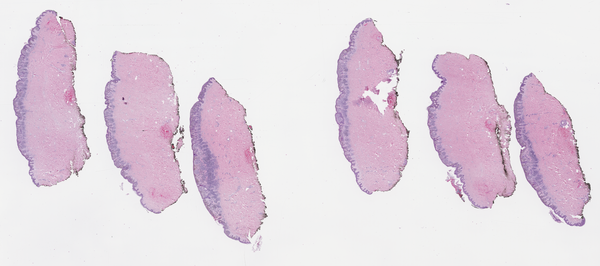

Supplement: S2 Dataset — (ZIP) [file pone.0297146.s008.zip › HE/764181_HE.png]

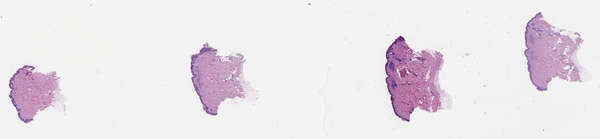

Supplement: S2 Dataset — (ZIP) [file pone.0297146.s008.zip › HE/119687_HE.png]

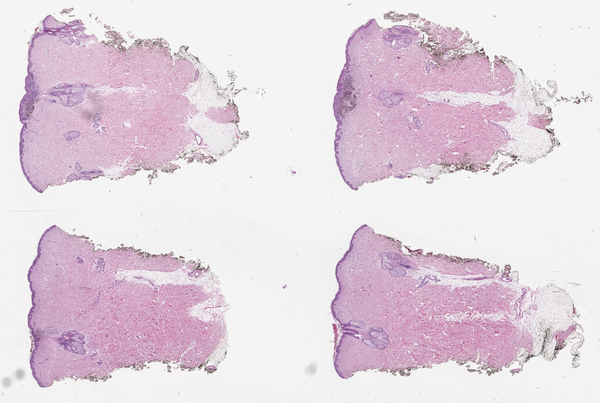

Supplement: S2 Dataset — (ZIP) [file pone.0297146.s008.zip › HE/308477_HE.png]

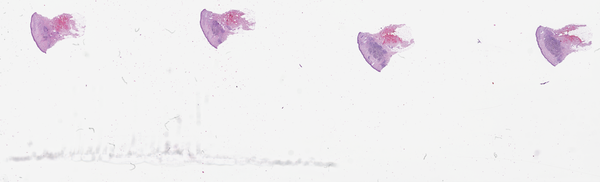

Supplement: S2 Dataset — (ZIP) [file pone.0297146.s008.zip › HE/196757_HE.png]

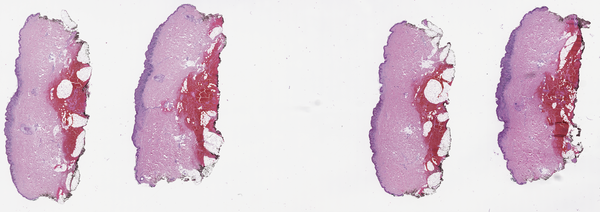

Supplement: S2 Dataset — (ZIP) [file pone.0297146.s008.zip › HE/232419-1_HE.png]

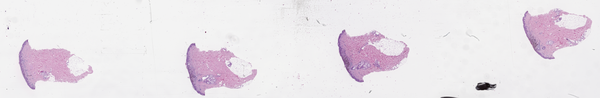

Supplement: S2 Dataset — (ZIP) [file pone.0297146.s008.zip › HE/662985_HE.png]

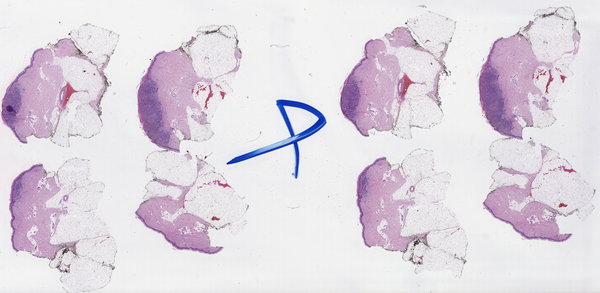

Supplement: S2 Dataset — (ZIP) [file pone.0297146.s008.zip › HE/289639_HE.png]

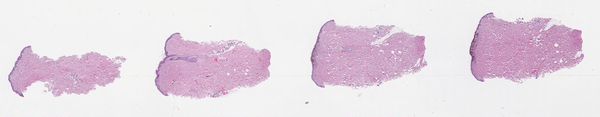

Supplement: S2 Dataset — (ZIP) [file pone.0297146.s008.zip › HE/573563-2_HE.png]

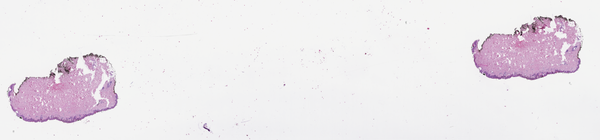

Supplement: S2 Dataset — (ZIP) [file pone.0297146.s008.zip › HE/849969-2_HE.png]

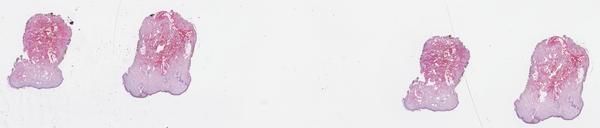

Supplement: S2 Dataset — (ZIP) [file pone.0297146.s008.zip › HE/652632_HE.png]

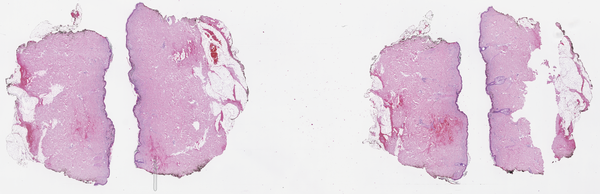

Supplement: S2 Dataset — (ZIP) [file pone.0297146.s008.zip › HE/413935_HE.png]

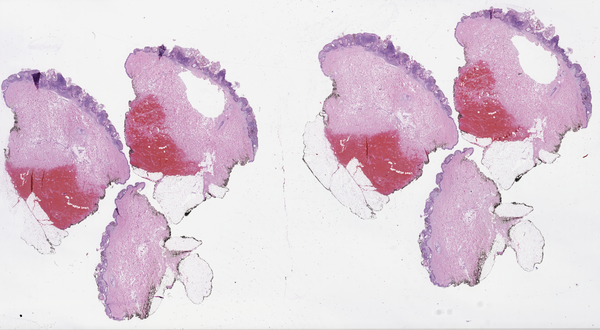

Supplement: S2 Dataset — (ZIP) [file pone.0297146.s008.zip › HE/414417-2_HE.png]

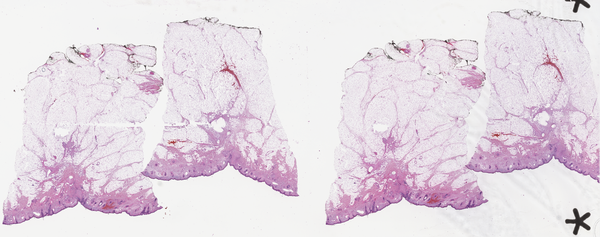

Supplement: S2 Dataset — (ZIP) [file pone.0297146.s008.zip › HE/639877_HE.png]

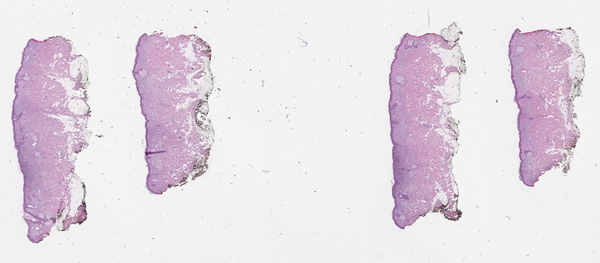

Supplement: S2 Dataset — (ZIP) [file pone.0297146.s008.zip › HE/216647_HE.png]

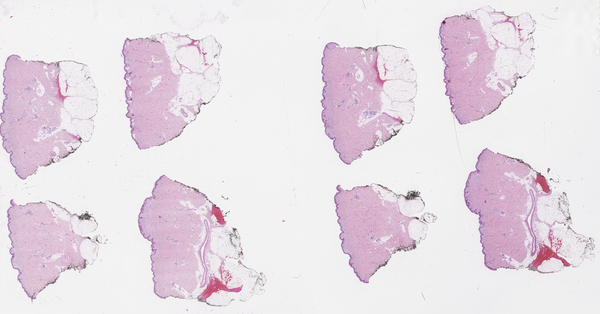

Supplement: S2 Dataset — (ZIP) [file pone.0297146.s008.zip › HE/285234_HE.png]

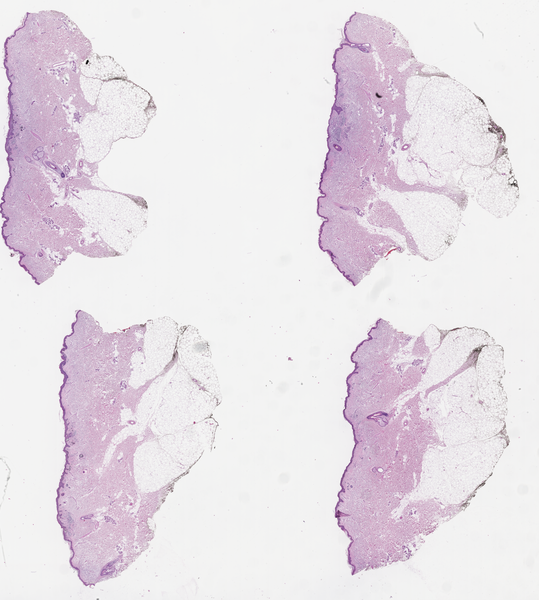

Supplement: S2 Dataset — (ZIP) [file pone.0297146.s008.zip › HE/228762_HE.png]

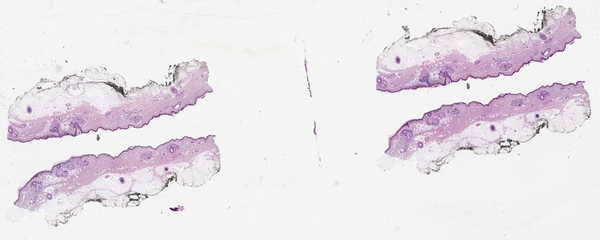

Supplement: S2 Dataset — (ZIP) [file pone.0297146.s008.zip › HE/717314_HE.png]

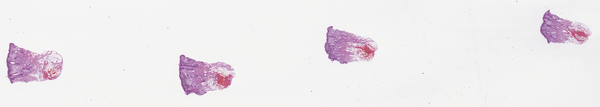

Supplement: S2 Dataset — (ZIP) [file pone.0297146.s008.zip › HE/638990_HE.png]

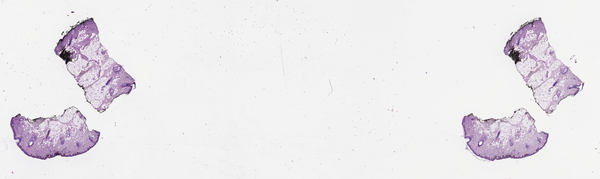

Supplement: S2 Dataset — (ZIP) [file pone.0297146.s008.zip › HE/830638_HE.png]

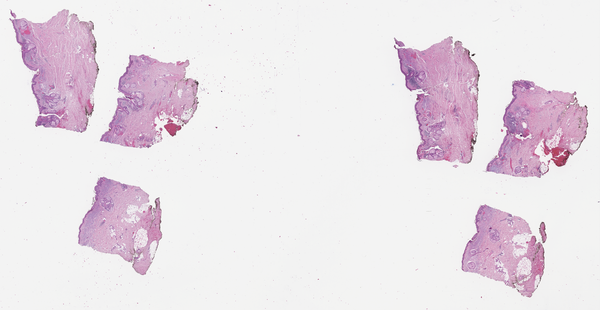

Supplement: S2 Dataset — (ZIP) [file pone.0297146.s008.zip › HE/525132-1_HE.png]

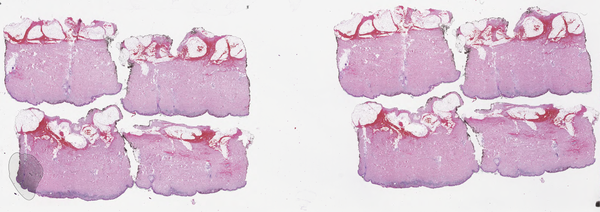

Supplement: S2 Dataset — (ZIP) [file pone.0297146.s008.zip › HE/515966_HE.png]

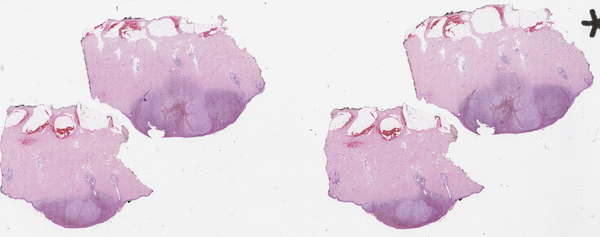

Supplement: S2 Dataset — (ZIP) [file pone.0297146.s008.zip › HE/222180_HE.png]

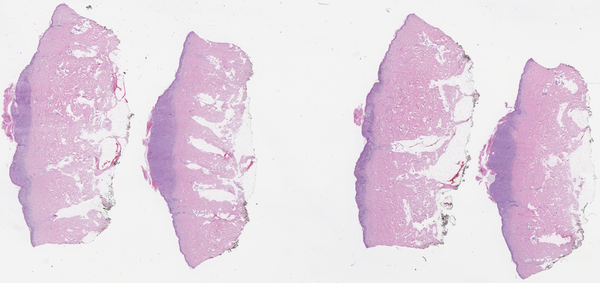

Supplement: S2 Dataset — (ZIP) [file pone.0297146.s008.zip › HE/408086_HE.png]

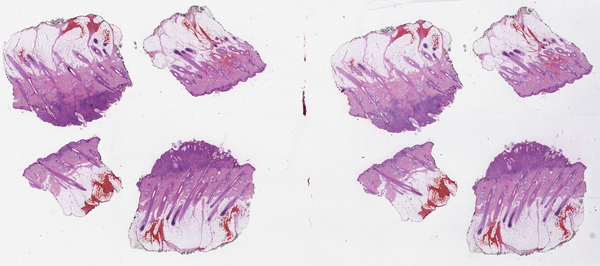

Supplement: S2 Dataset — (ZIP) [file pone.0297146.s008.zip › HE/546569-1_HE.png]

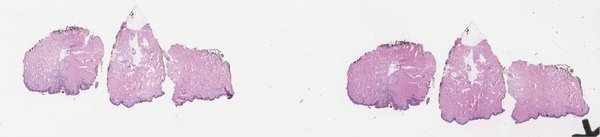

Supplement: S2 Dataset — (ZIP) [file pone.0297146.s008.zip › HE/113308_HE.png]

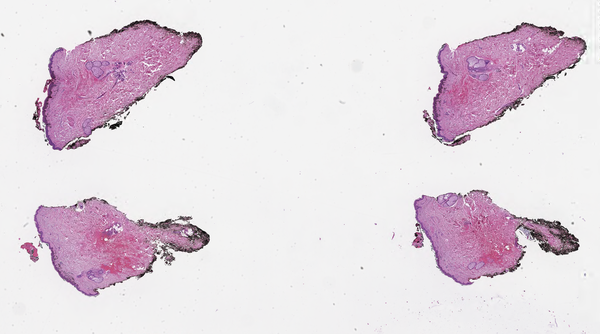

Supplement: S2 Dataset — (ZIP) [file pone.0297146.s008.zip › HE/200197_HE.png]

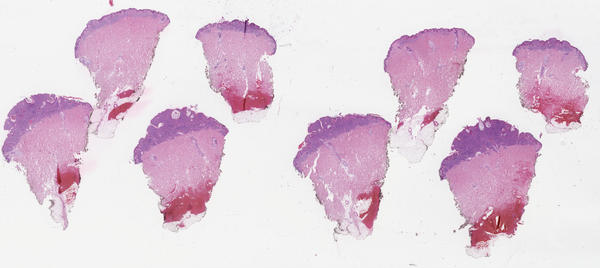

Supplement: S2 Dataset — (ZIP) [file pone.0297146.s008.zip › HE/200427-1_HE.png]

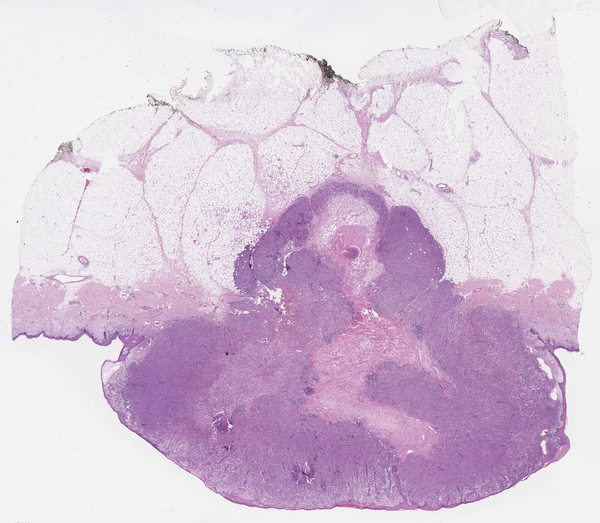

Supplement: S2 Dataset — (ZIP) [file pone.0297146.s008.zip › HE/237476_HE.png]

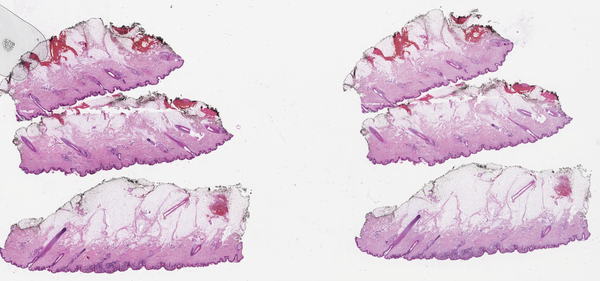

Supplement: S2 Dataset — (ZIP) [file pone.0297146.s008.zip › HE/486741_HE.png]

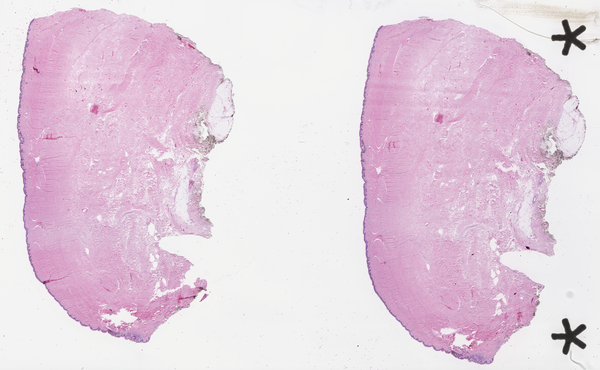

Supplement: S2 Dataset — (ZIP) [file pone.0297146.s008.zip › HE/763152_HE.png]

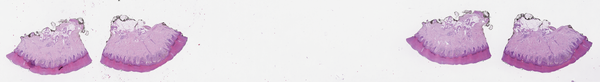

Supplement: S2 Dataset — (ZIP) [file pone.0297146.s008.zip › HE/450912-1_HE.png]

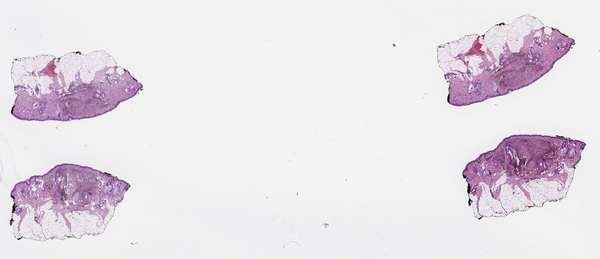

Supplement: S2 Dataset — (ZIP) [file pone.0297146.s008.zip › HE/842825_HE.png]

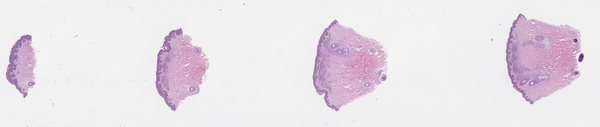

Supplement: S2 Dataset — (ZIP) [file pone.0297146.s008.zip › HE/105229_HE.png]

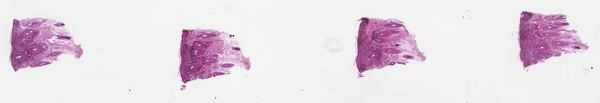

Supplement: S2 Dataset — (ZIP) [file pone.0297146.s008.zip › HE/783370_HE.png]

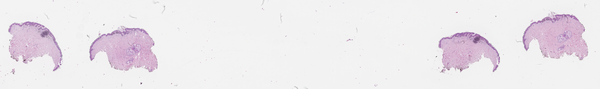

Supplement: S2 Dataset — (ZIP) [file pone.0297146.s008.zip › HE/716005_HE.png]

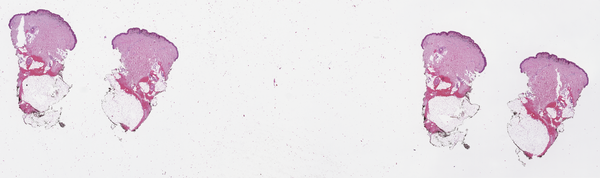

Supplement: S2 Dataset — (ZIP) [file pone.0297146.s008.zip › HE/515829_HE.png]

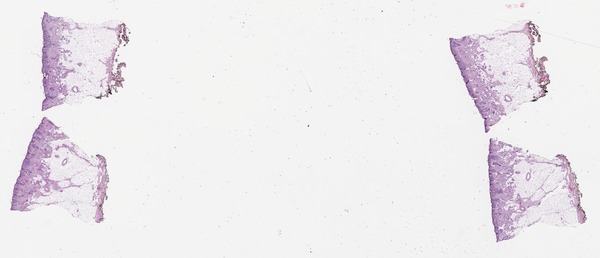

Supplement: S2 Dataset — (ZIP) [file pone.0297146.s008.zip › HE/679218_HE.png]

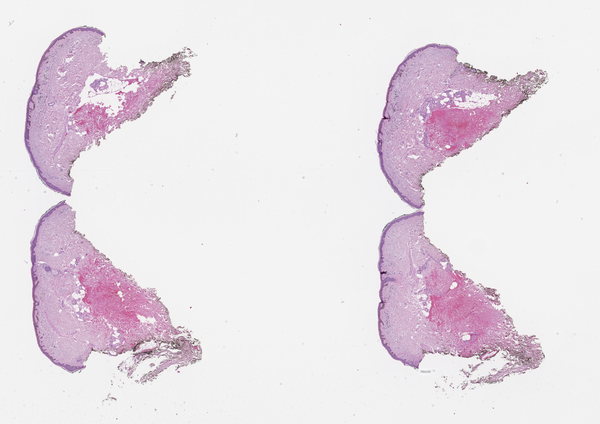

Supplement: S2 Dataset — (ZIP) [file pone.0297146.s008.zip › HE/726303-1_HE.png]

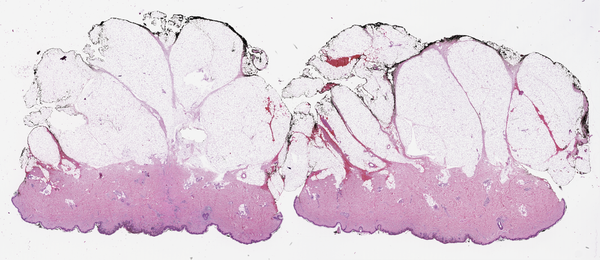

Supplement: S2 Dataset — (ZIP) [file pone.0297146.s008.zip › HE/626414_HE.png]

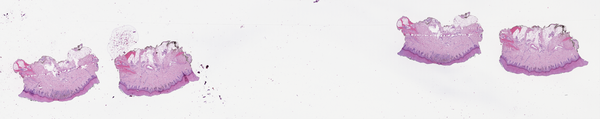

Supplement: S2 Dataset — (ZIP) [file pone.0297146.s008.zip › HE/676277_HE.png]

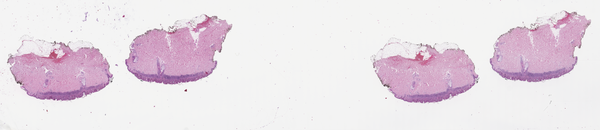

Supplement: S2 Dataset — (ZIP) [file pone.0297146.s008.zip › HE/729980-2_HE.png]

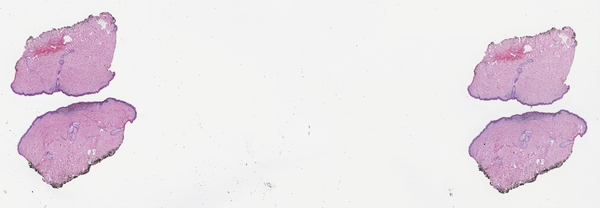

Supplement: S2 Dataset — (ZIP) [file pone.0297146.s008.zip › HE/665087-2_HE.png]

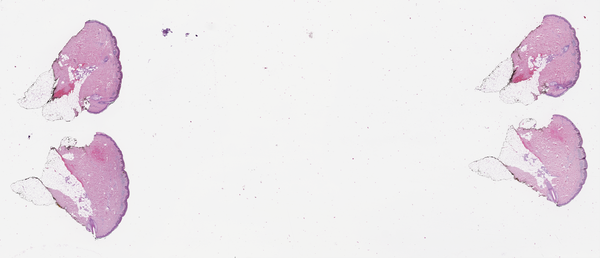

Supplement: S2 Dataset — (ZIP) [file pone.0297146.s008.zip › HE/335663_HE.png]

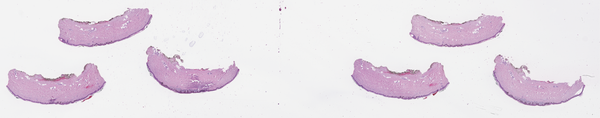

Supplement: S2 Dataset — (ZIP) [file pone.0297146.s008.zip › HE/542754-1_HE.png]

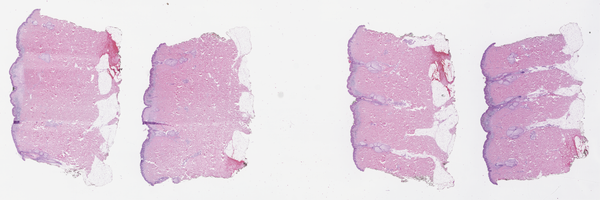

Supplement: S2 Dataset — (ZIP) [file pone.0297146.s008.zip › HE/676708-1_HE.png]

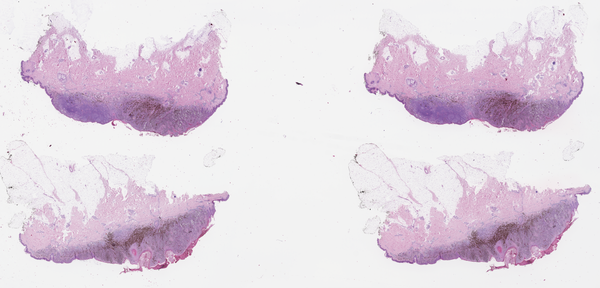

Supplement: S2 Dataset — (ZIP) [file pone.0297146.s008.zip › HE/753457_HE.png]

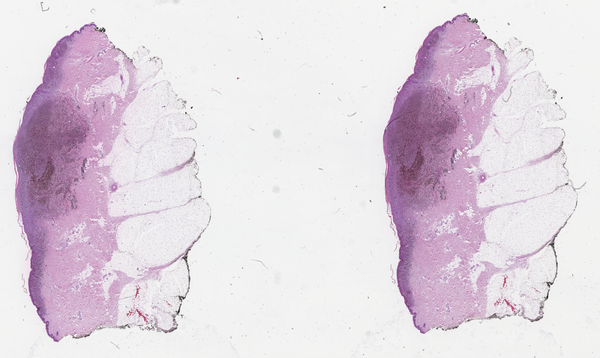

Supplement: S2 Dataset — (ZIP) [file pone.0297146.s008.zip › HE/205260_HE.png]

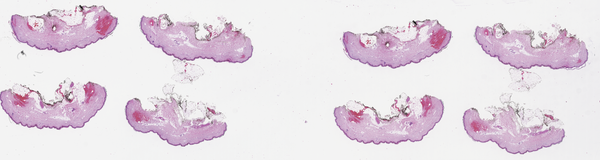

Supplement: S2 Dataset — (ZIP) [file pone.0297146.s008.zip › HE/495868_HE.png]

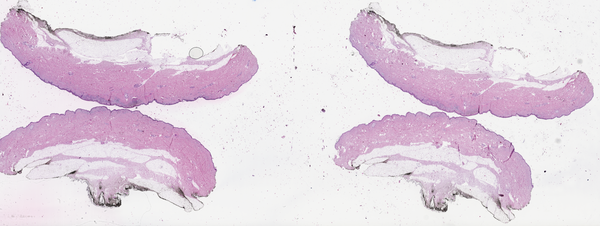

Supplement: S2 Dataset — (ZIP) [file pone.0297146.s008.zip › HE/260415_HE.png]

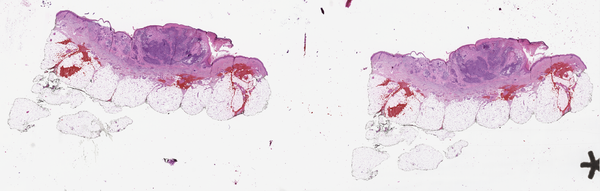

Supplement: S2 Dataset — (ZIP) [file pone.0297146.s008.zip › HE/618720_HE.png]

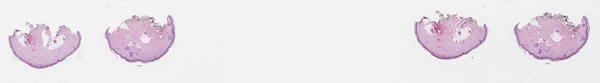

Supplement: S2 Dataset — (ZIP) [file pone.0297146.s008.zip › HE/698154_HE.png]

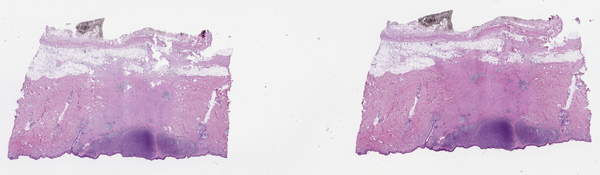

Supplement: S2 Dataset — (ZIP) [file pone.0297146.s008.zip › HE/826016_HE.png]

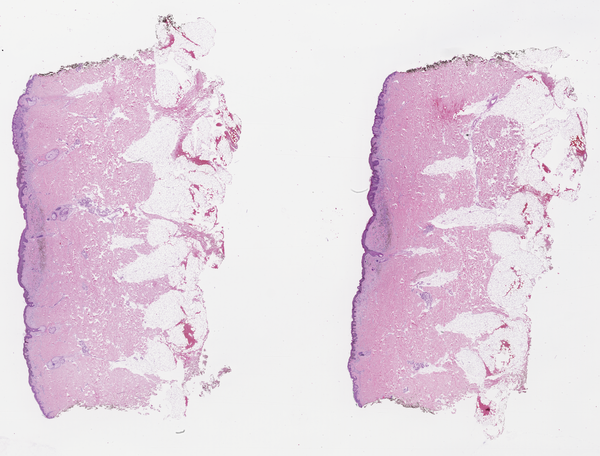

Supplement: S2 Dataset — (ZIP) [file pone.0297146.s008.zip › HE/703576_HE.png]

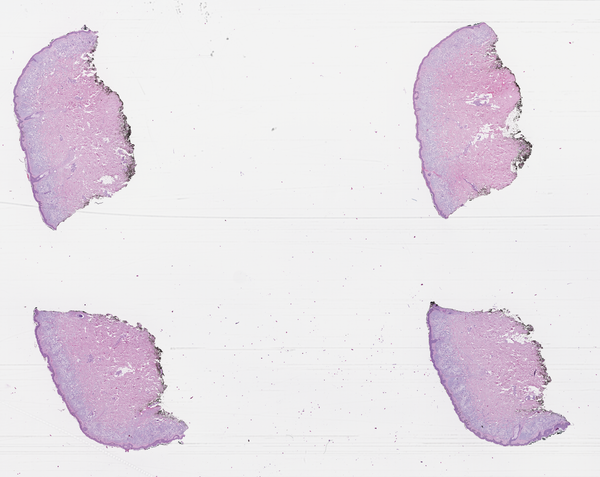

Supplement: S2 Dataset — (ZIP) [file pone.0297146.s008.zip › HE/668403-1_HE.png]

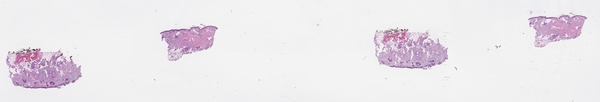

Supplement: S2 Dataset — (ZIP) [file pone.0297146.s008.zip › HE/782692_HE.png]

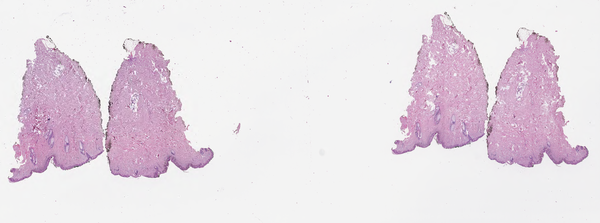

Supplement: S2 Dataset — (ZIP) [file pone.0297146.s008.zip › HE/563033-1_HE.png]

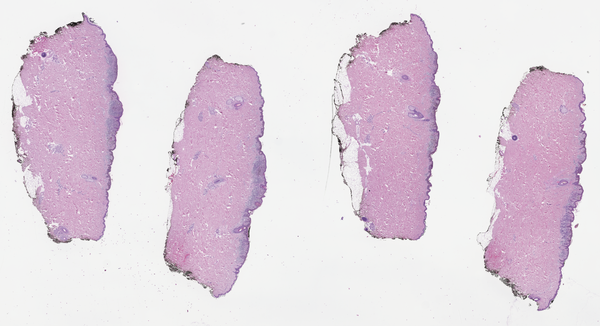

Supplement: S2 Dataset — (ZIP) [file pone.0297146.s008.zip › HE/813888_HE.png]

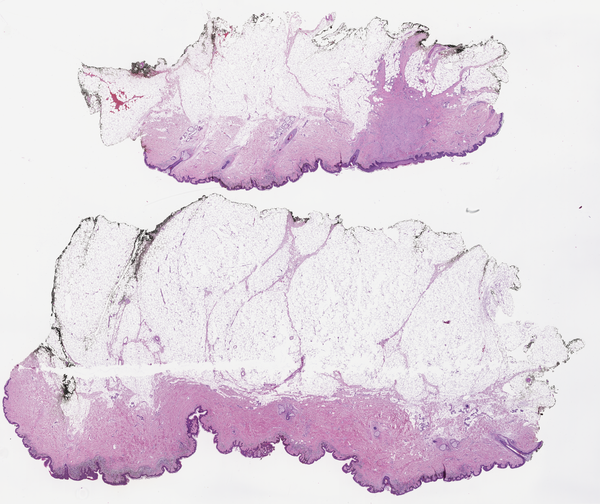

Supplement: S2 Dataset — (ZIP) [file pone.0297146.s008.zip › HE/287913_HE.png]

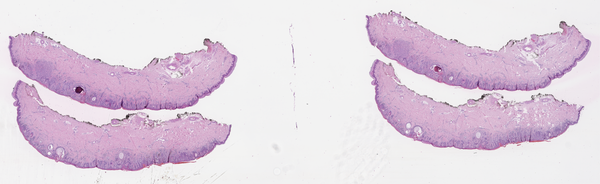

Supplement: S2 Dataset — (ZIP) [file pone.0297146.s008.zip › HE/768250_HE.png]

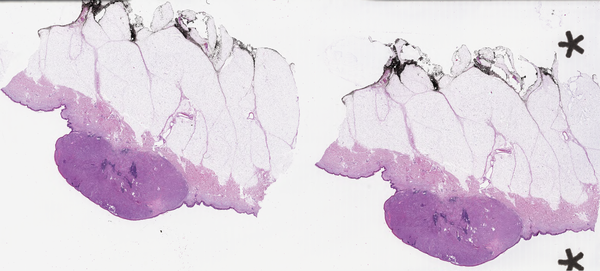

Supplement: S2 Dataset — (ZIP) [file pone.0297146.s008.zip › HE/558024_HE.png]

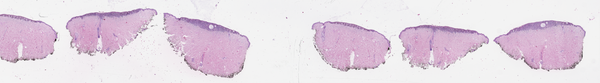

Supplement: S2 Dataset — (ZIP) [file pone.0297146.s008.zip › HE/672910-1_HE.png]

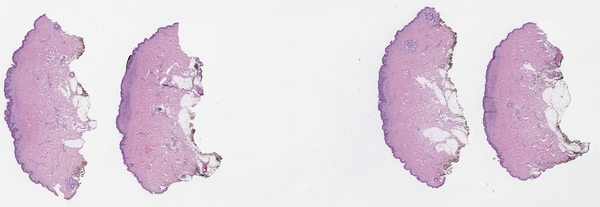

Supplement: S2 Dataset — (ZIP) [file pone.0297146.s008.zip › HE/232419-2_HE.png]

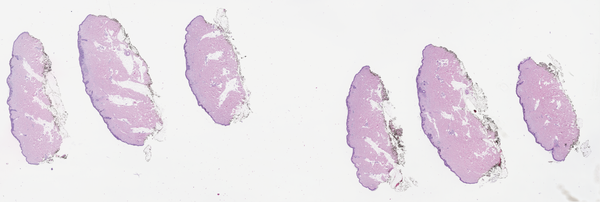

Supplement: S2 Dataset — (ZIP) [file pone.0297146.s008.zip › HE/745192-2_HE.png]

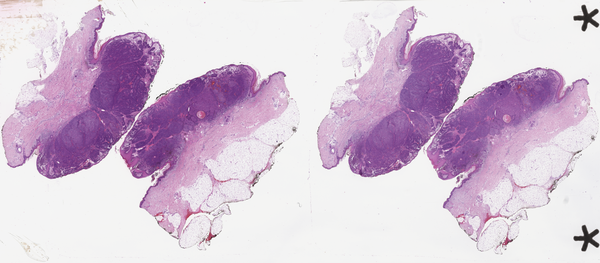

Supplement: S2 Dataset — (ZIP) [file pone.0297146.s008.zip › HE/101292_HE.png]

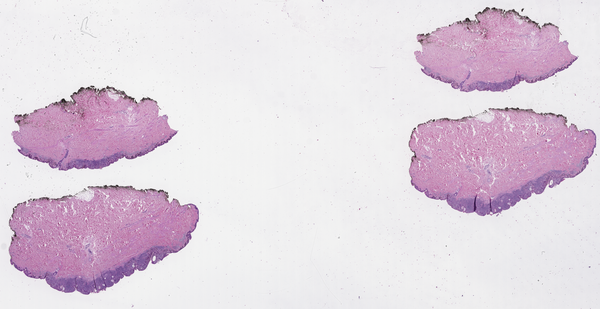

Supplement: S2 Dataset — (ZIP) [file pone.0297146.s008.zip › HE/278485-1_HE.png]

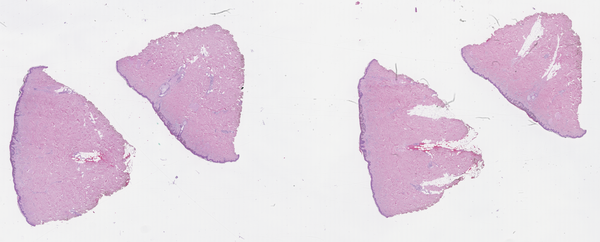

Supplement: S2 Dataset — (ZIP) [file pone.0297146.s008.zip › HE/642570_HE.png]

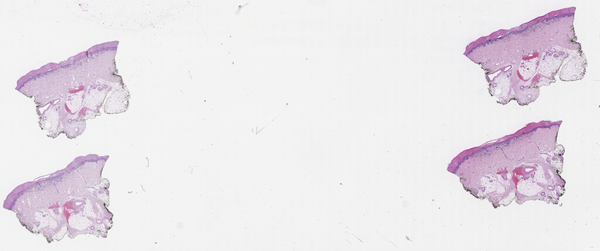

Supplement: S2 Dataset — (ZIP) [file pone.0297146.s008.zip › HE/238717_HE.png]

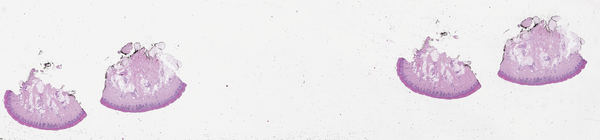

Supplement: S2 Dataset — (ZIP) [file pone.0297146.s008.zip › HE/278485-2_HE.png]

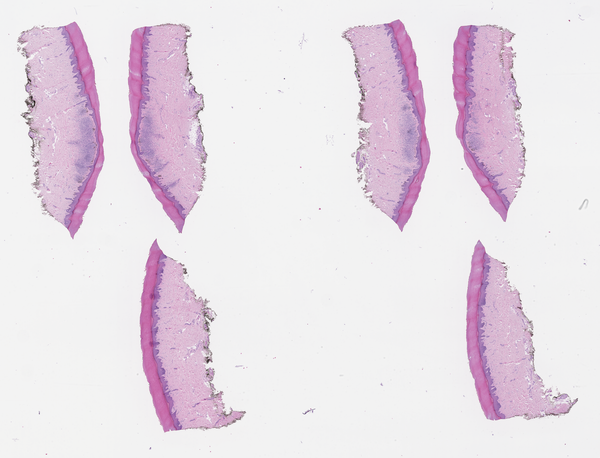

Supplement: S2 Dataset — (ZIP) [file pone.0297146.s008.zip › HE/724056_HE.png]

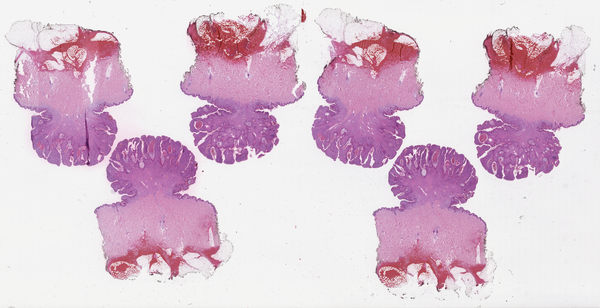

Supplement: S2 Dataset — (ZIP) [file pone.0297146.s008.zip › HE/200427-2_HE.png]

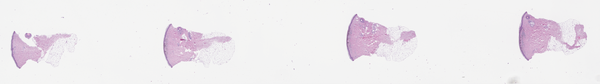

Supplement: S2 Dataset — (ZIP) [file pone.0297146.s008.zip › HE/652414-2_HE.png]

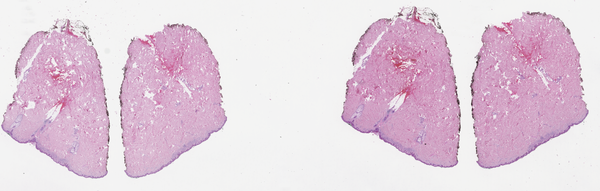

Supplement: S2 Dataset — (ZIP) [file pone.0297146.s008.zip › HE/465986-2_HE.png]

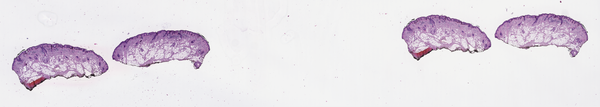

Supplement: S2 Dataset — (ZIP) [file pone.0297146.s008.zip › HE/599560-2_HE.png]

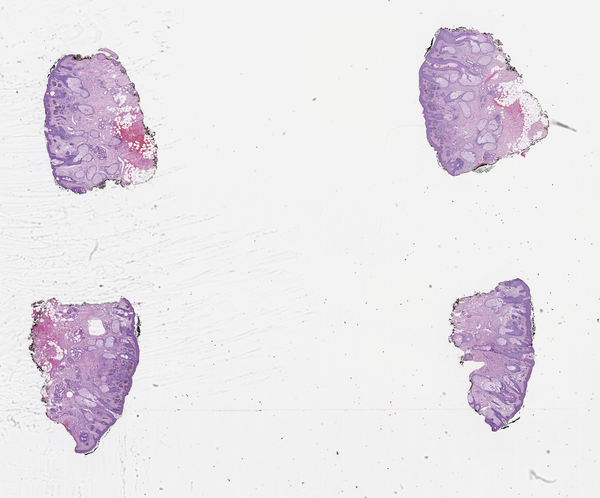

Supplement: S2 Dataset — (ZIP) [file pone.0297146.s008.zip › HE/109256_HE.png]

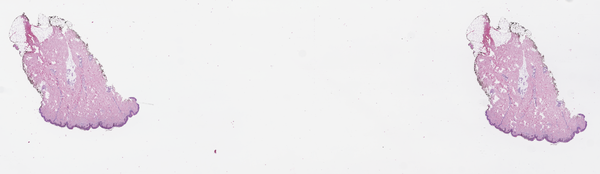

Supplement: S2 Dataset — (ZIP) [file pone.0297146.s008.zip › HE/570315_HE.png]

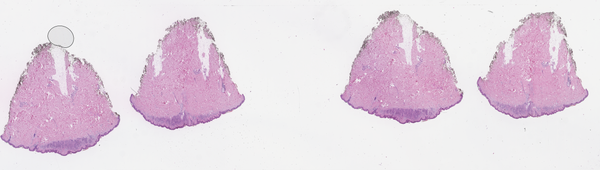

Supplement: S2 Dataset — (ZIP) [file pone.0297146.s008.zip › HE/224028_HE.png]

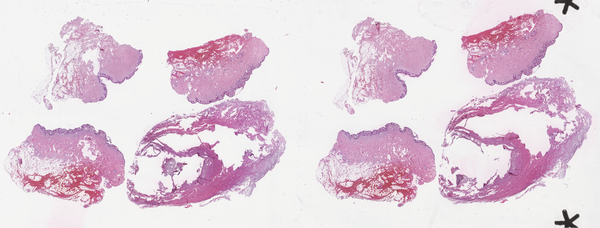

Supplement: S2 Dataset — (ZIP) [file pone.0297146.s008.zip › HE/646146_HE.png]

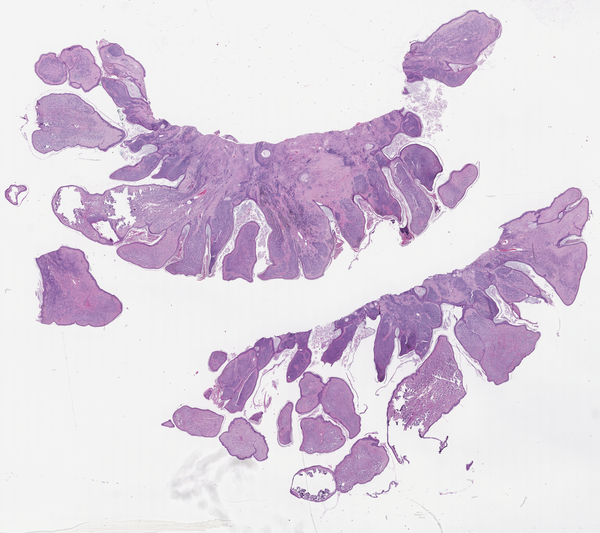

Supplement: S2 Dataset — (ZIP) [file pone.0297146.s008.zip › HE/414417-1_HE.png]

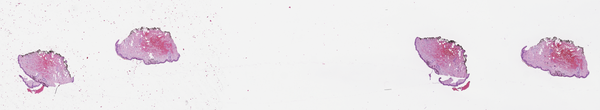

Supplement: S2 Dataset — (ZIP) [file pone.0297146.s008.zip › HE/653604_HE.png]

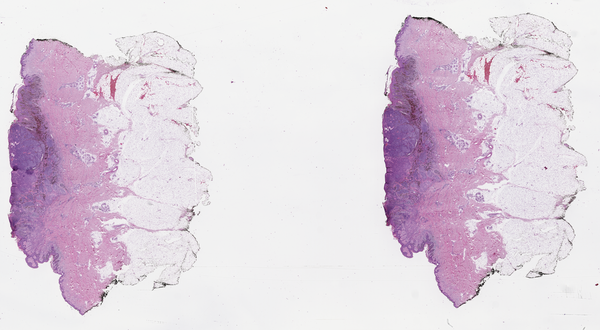

Supplement: S2 Dataset — (ZIP) [file pone.0297146.s008.zip › HE/827832_HE.png]

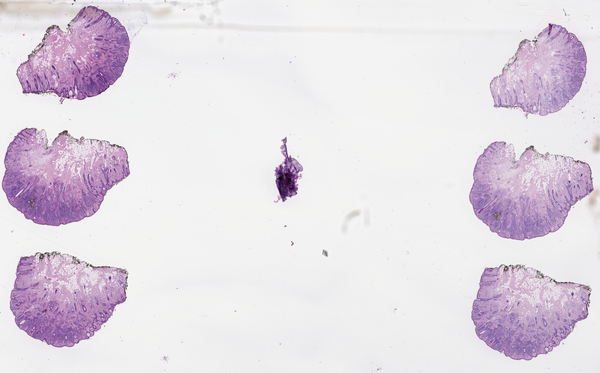

Supplement: S2 Dataset — (ZIP) [file pone.0297146.s008.zip › HE/700800_HE.png]

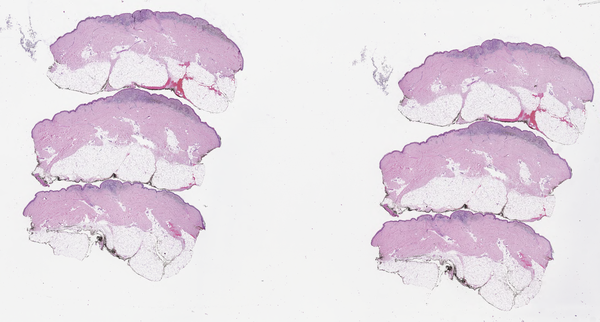

Supplement: S2 Dataset — (ZIP) [file pone.0297146.s008.zip › HE/521912_HE.png]

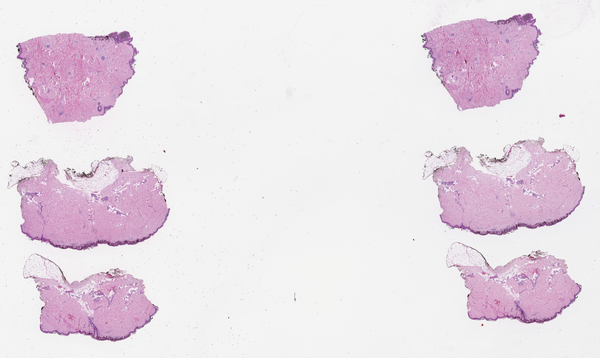

Supplement: S2 Dataset — (ZIP) [file pone.0297146.s008.zip › HE/716389_HE.png]

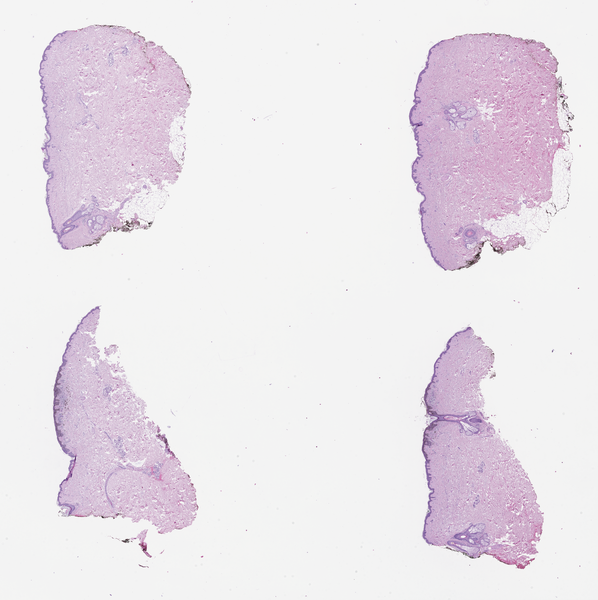

Supplement: S2 Dataset — (ZIP) [file pone.0297146.s008.zip › HE/759437_HE.png]

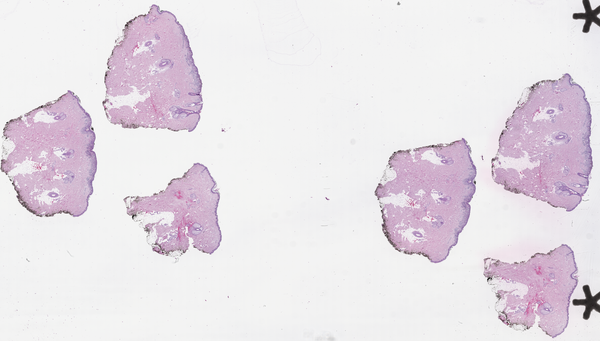

Supplement: S2 Dataset — (ZIP) [file pone.0297146.s008.zip › HE/259985_HE.png]

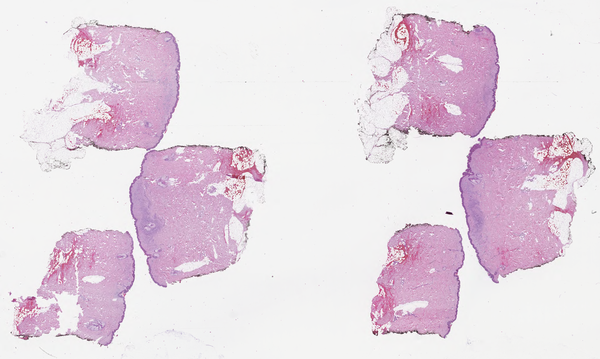

Supplement: S2 Dataset — (ZIP) [file pone.0297146.s008.zip › HE/549760_HE.png]

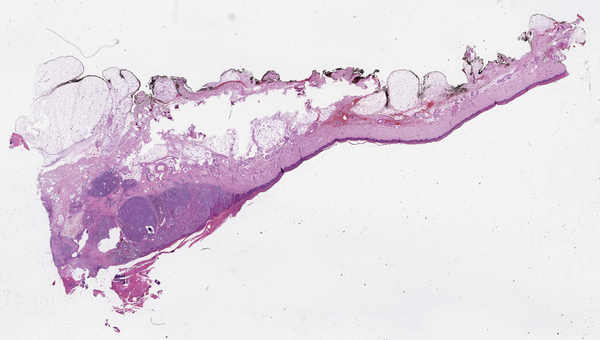

Supplement: S2 Dataset — (ZIP) [file pone.0297146.s008.zip › HE/787884_HE.png]

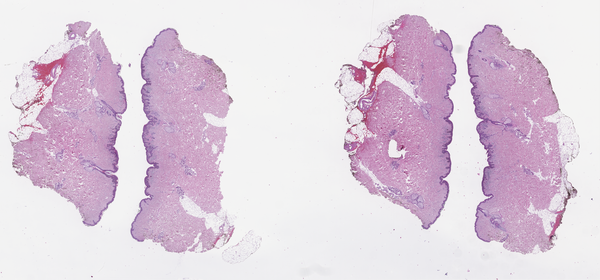

Supplement: S2 Dataset — (ZIP) [file pone.0297146.s008.zip › HE/619807-1_HE.png]

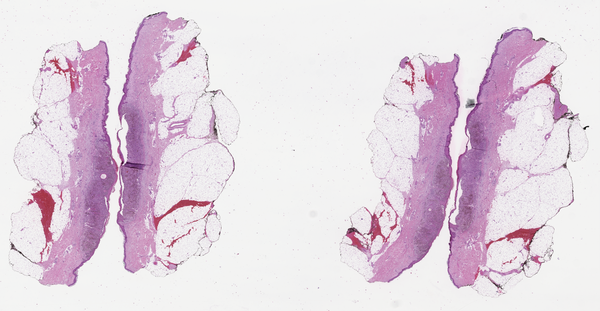

Supplement: S2 Dataset — (ZIP) [file pone.0297146.s008.zip › HE/800231_HE.png]

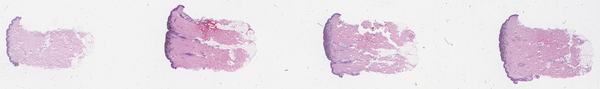

Supplement: S2 Dataset — (ZIP) [file pone.0297146.s008.zip › HE/318580_HE.png]

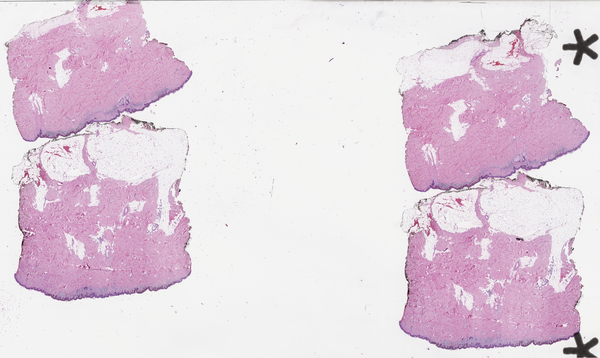

Supplement: S2 Dataset — (ZIP) [file pone.0297146.s008.zip › HE/401945-1_HE.png]

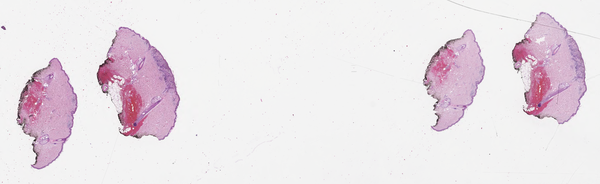

Supplement: S2 Dataset — (ZIP) [file pone.0297146.s008.zip › HE/408057_HE.png]

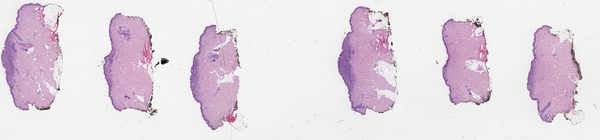

Supplement: S2 Dataset — (ZIP) [file pone.0297146.s008.zip › HE/243474_HE.png]

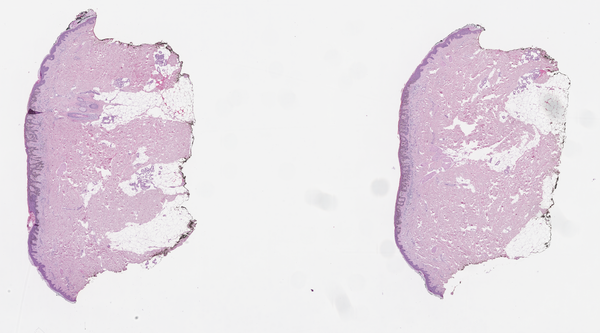

Supplement: S2 Dataset — (ZIP) [file pone.0297146.s008.zip › HE/417366_HE.png]

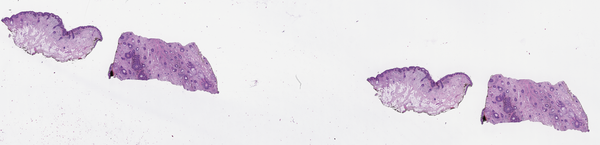

Supplement: S2 Dataset — (ZIP) [file pone.0297146.s008.zip › HE/499846_HE.png]

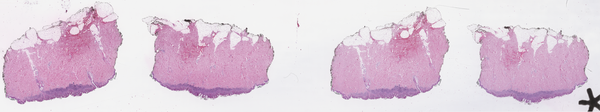

Supplement: S2 Dataset — (ZIP) [file pone.0297146.s008.zip › HE/729980-1_HE.png]

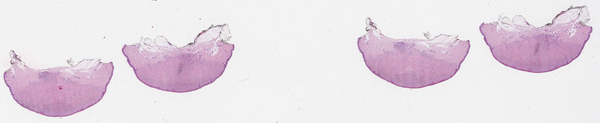

Supplement: S2 Dataset — (ZIP) [file pone.0297146.s008.zip › HE/231847_HE.png]

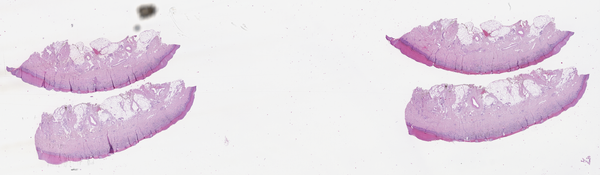

Supplement: S2 Dataset — (ZIP) [file pone.0297146.s008.zip › HE/673151_HE.png]

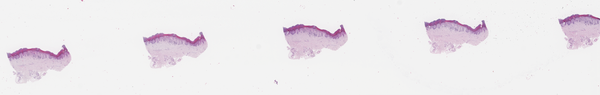

Supplement: S3 Dataset — (ZIP) [file pone.0297146.s009.zip › erlangen/HE/421431_HE.png]
