# Supplementary figures and images for: Evaluating deep learning-based melanoma classification using immunohistochemistry and routine histology: A three center study (part 3 of 7)
Source: PLoS One. 2024 Jan 19;19(1):e0297146. doi: 10.1371/journal.pone.0297146 (PMC10798511; doi:10.1371/journal.pone.0297146)

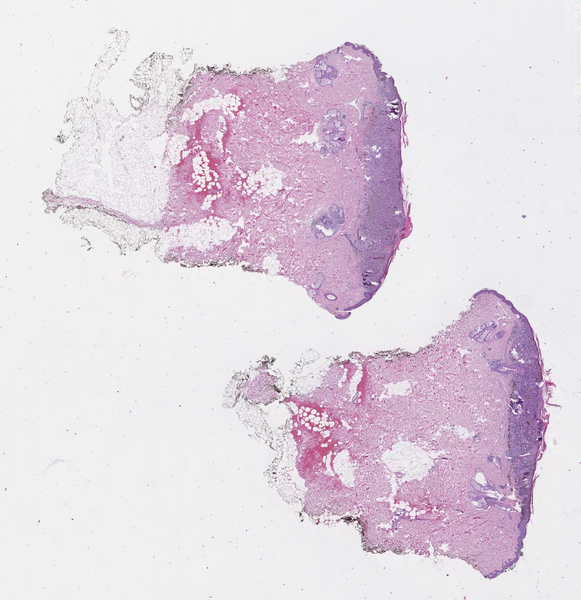

Supplement: S2 Dataset — (ZIP) [file pone.0297146.s008.zip › HE/341033_HE.png]

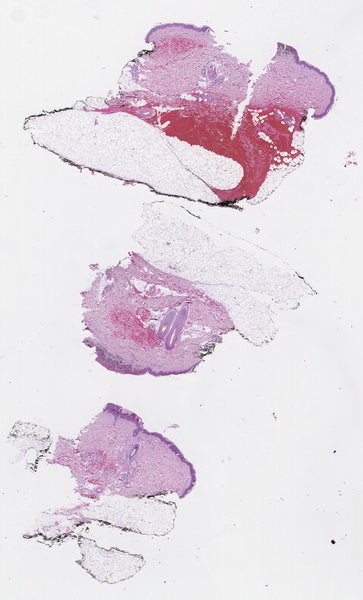

Supplement: S2 Dataset — (ZIP) [file pone.0297146.s008.zip › HE/516621_HE.png]

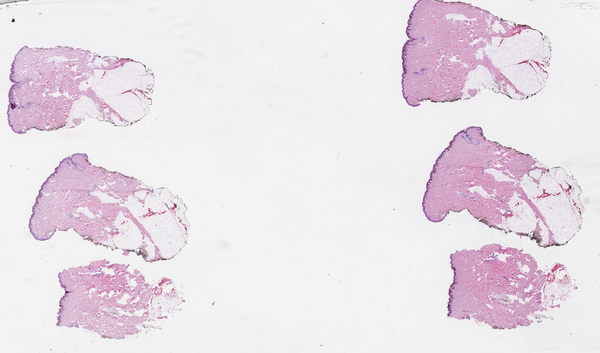

Supplement: S2 Dataset — (ZIP) [file pone.0297146.s008.zip › HE/725823_HE.png]

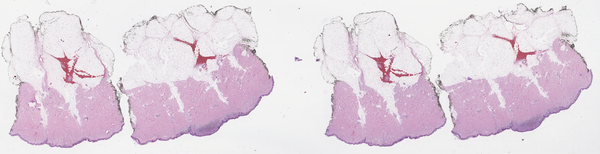

Supplement: S2 Dataset — (ZIP) [file pone.0297146.s008.zip › HE/261250_HE.png]

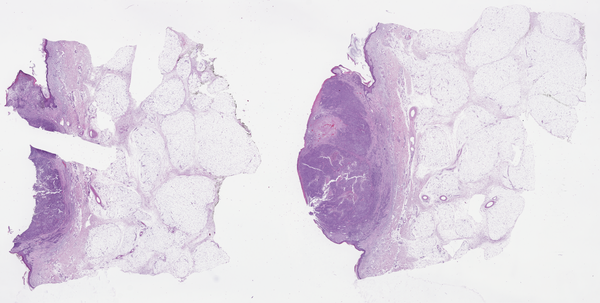

Supplement: S2 Dataset — (ZIP) [file pone.0297146.s008.zip › HE/797778_HE.png]

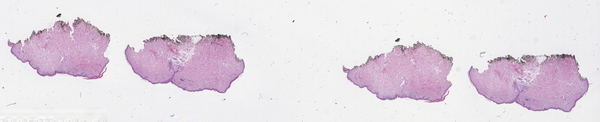

Supplement: S2 Dataset — (ZIP) [file pone.0297146.s008.zip › HE/203200_HE.png]

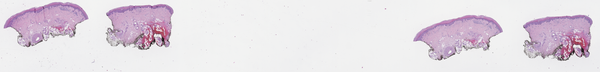

Supplement: S2 Dataset — (ZIP) [file pone.0297146.s008.zip › HE/774309-2_HE.png]

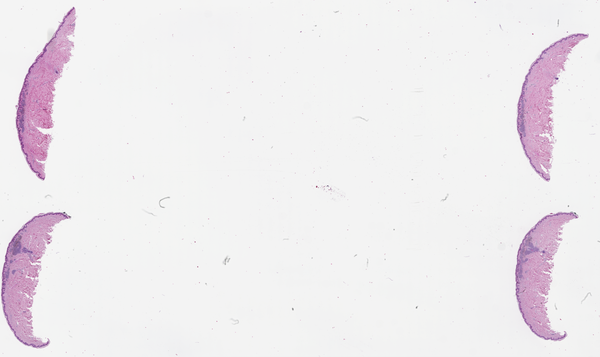

Supplement: S2 Dataset — (ZIP) [file pone.0297146.s008.zip › HE/428772_HE.png]

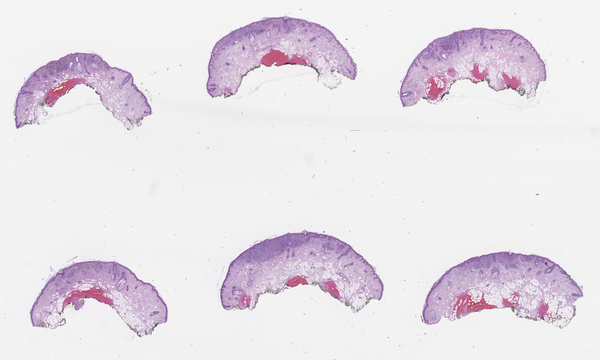

Supplement: S2 Dataset — (ZIP) [file pone.0297146.s008.zip › HE/799469_HE.png]

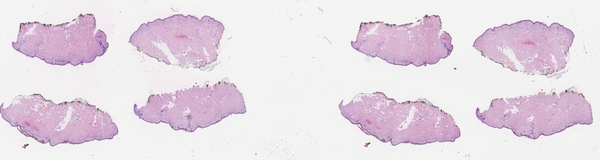

Supplement: S2 Dataset — (ZIP) [file pone.0297146.s008.zip › HE/796338_HE.png]

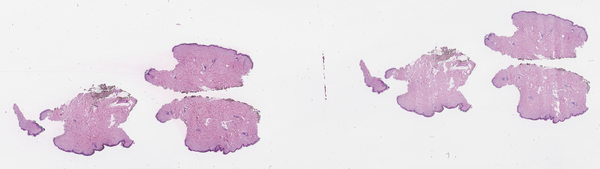

Supplement: S2 Dataset — (ZIP) [file pone.0297146.s008.zip › HE/688070-1_HE.png]

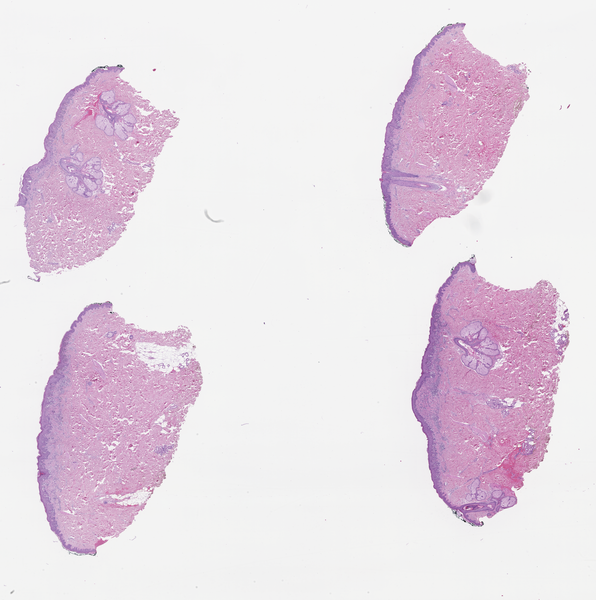

Supplement: S2 Dataset — (ZIP) [file pone.0297146.s008.zip › HE/692561_HE.png]

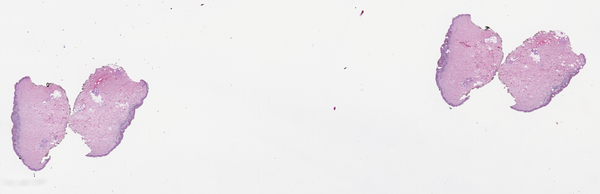

Supplement: S2 Dataset — (ZIP) [file pone.0297146.s008.zip › HE/333072_HE.png]

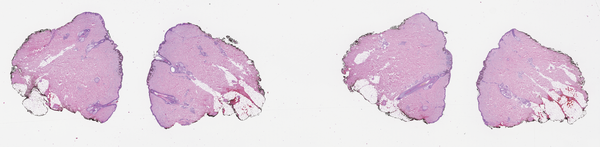

Supplement: S2 Dataset — (ZIP) [file pone.0297146.s008.zip › HE/773004-2_HE.png]

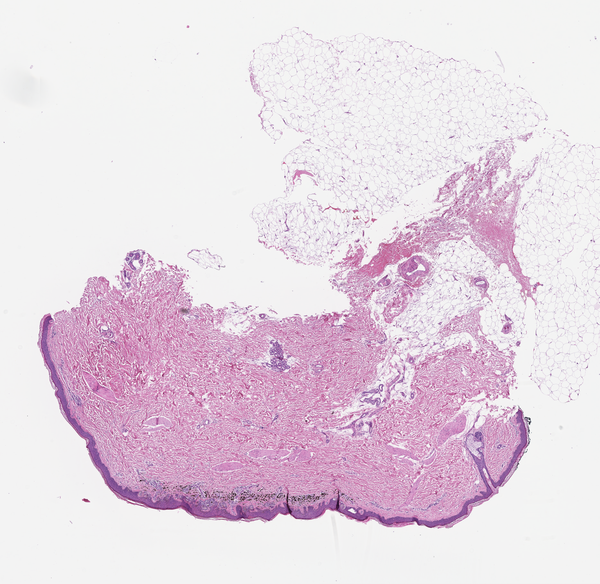

Supplement: S2 Dataset — (ZIP) [file pone.0297146.s008.zip › HE/420270_HE.png]

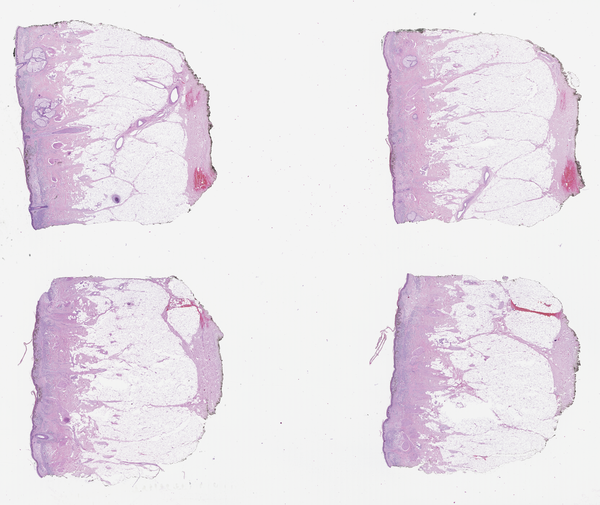

Supplement: S2 Dataset — (ZIP) [file pone.0297146.s008.zip › HE/773387_HE.png]

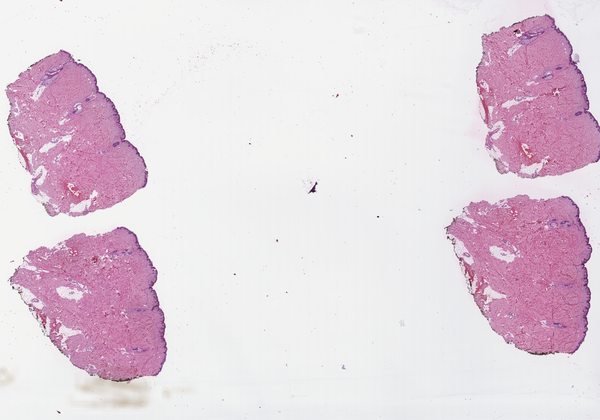

Supplement: S2 Dataset — (ZIP) [file pone.0297146.s008.zip › HE/732460_HE.png]

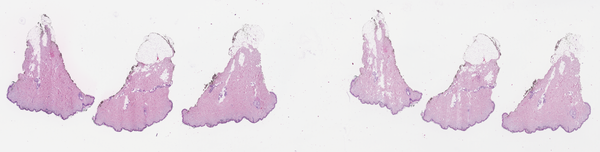

Supplement: S2 Dataset — (ZIP) [file pone.0297146.s008.zip › HE/688070-2_HE.png]

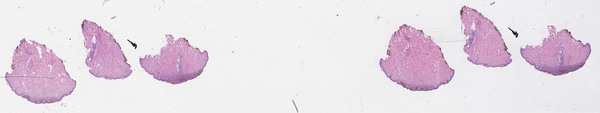

Supplement: S2 Dataset — (ZIP) [file pone.0297146.s008.zip › HE/550664-1_HE.png]

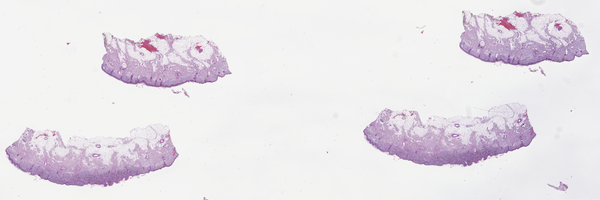

Supplement: S2 Dataset — (ZIP) [file pone.0297146.s008.zip › HE/856413_HE.png]

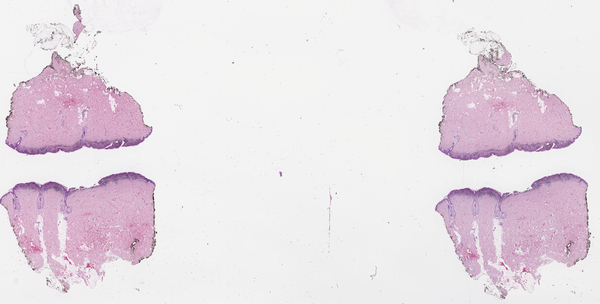

Supplement: S2 Dataset — (ZIP) [file pone.0297146.s008.zip › HE/303511_HE.png]

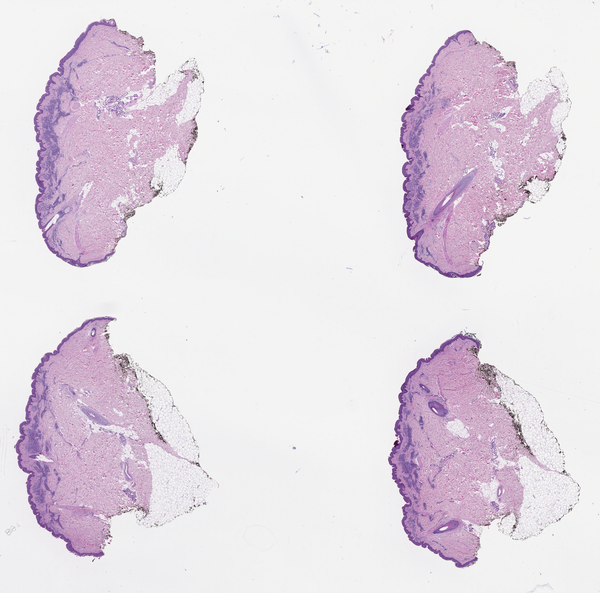

Supplement: S2 Dataset — (ZIP) [file pone.0297146.s008.zip › HE/115528_HE.png]

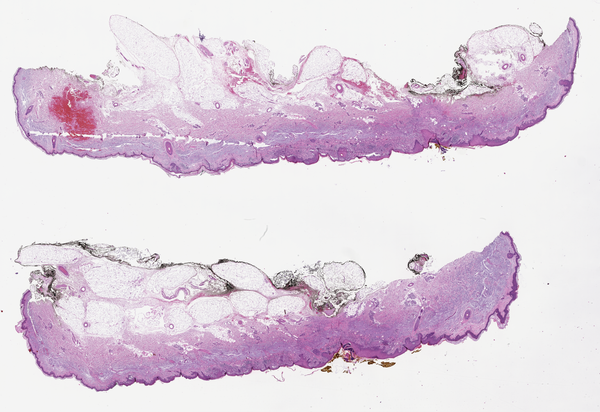

Supplement: S2 Dataset — (ZIP) [file pone.0297146.s008.zip › HE/545094_HE.png]

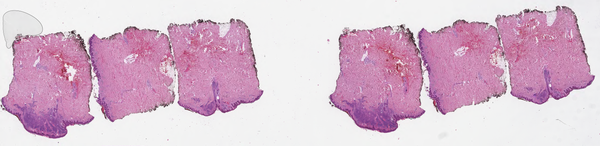

Supplement: S2 Dataset — (ZIP) [file pone.0297146.s008.zip › HE/546670_HE.png]

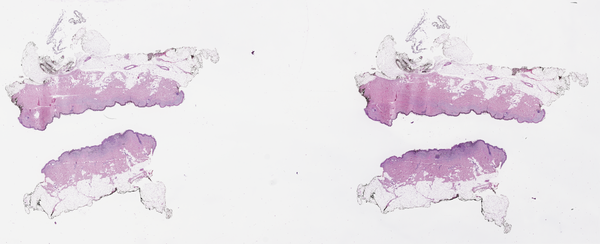

Supplement: S2 Dataset — (ZIP) [file pone.0297146.s008.zip › HE/693169_HE.png]

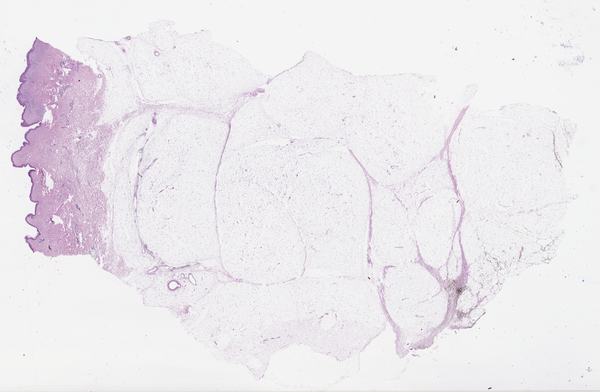

Supplement: S2 Dataset — (ZIP) [file pone.0297146.s008.zip › HE/619807-2_HE.png]

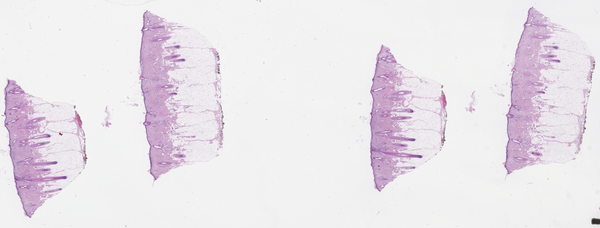

Supplement: S2 Dataset — (ZIP) [file pone.0297146.s008.zip › HE/451085_HE.png]

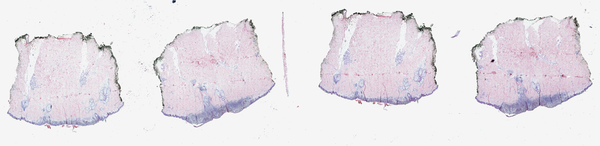

Supplement: S2 Dataset — (ZIP) [file pone.0297146.s008.zip › HE/108136_HE.png]

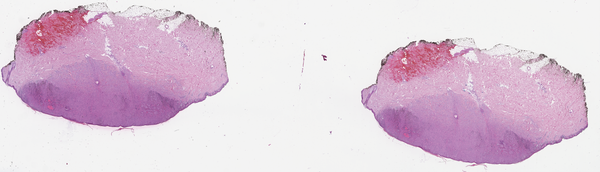

Supplement: S2 Dataset — (ZIP) [file pone.0297146.s008.zip › HE/219350_HE.png]

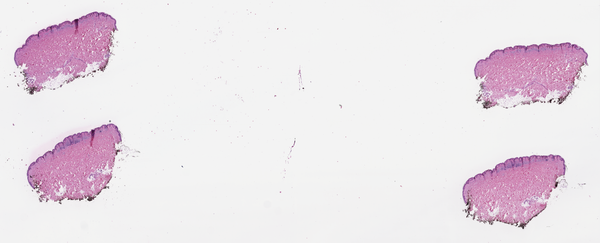

Supplement: S2 Dataset — (ZIP) [file pone.0297146.s008.zip › HE/115980_HE.png]

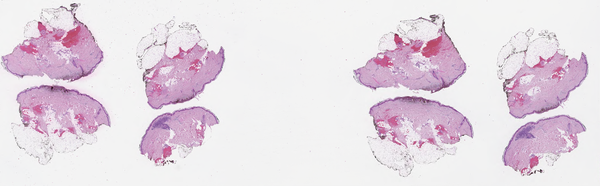

Supplement: S2 Dataset — (ZIP) [file pone.0297146.s008.zip › HE/262918_HE.png]

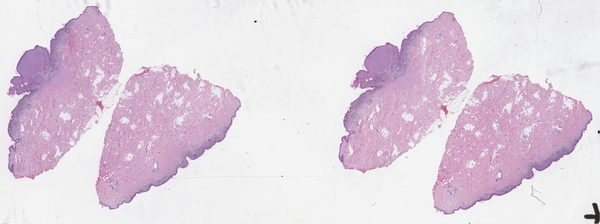

Supplement: S2 Dataset — (ZIP) [file pone.0297146.s008.zip › HE/661753_HE.png]

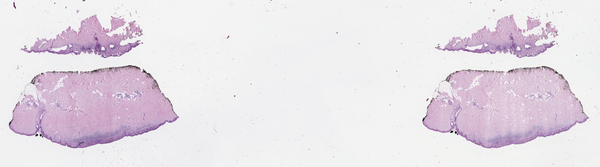

Supplement: S2 Dataset — (ZIP) [file pone.0297146.s008.zip › HE/115670_HE.png]

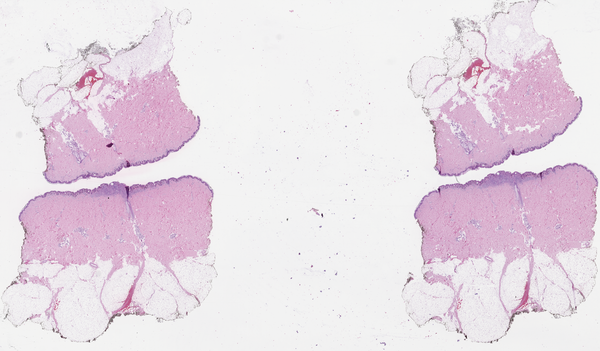

Supplement: S2 Dataset — (ZIP) [file pone.0297146.s008.zip › HE/282956-1_HE.png]

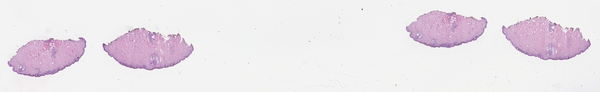

Supplement: S2 Dataset — (ZIP) [file pone.0297146.s008.zip › HE/792537_HE.png]

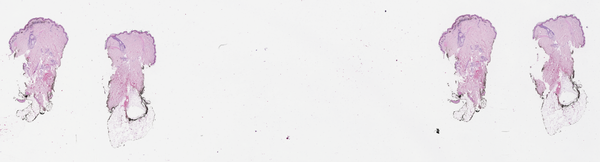

Supplement: S2 Dataset — (ZIP) [file pone.0297146.s008.zip › HE/425593-2_HE.png]

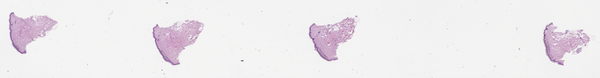

Supplement: S2 Dataset — (ZIP) [file pone.0297146.s008.zip › HE/495834_HE.png]

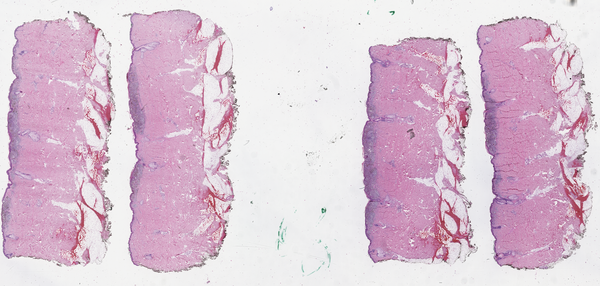

Supplement: S2 Dataset — (ZIP) [file pone.0297146.s008.zip › HE/220100_HE.png]

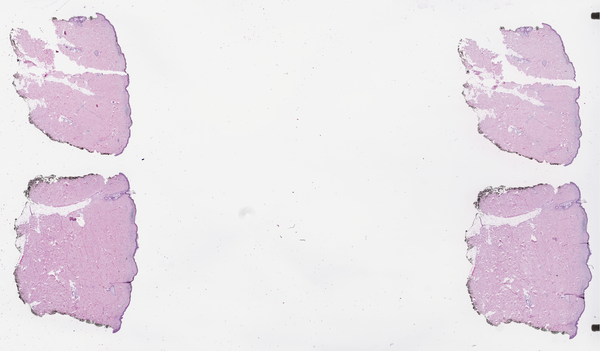

Supplement: S2 Dataset — (ZIP) [file pone.0297146.s008.zip › HE/749176_HE.png]

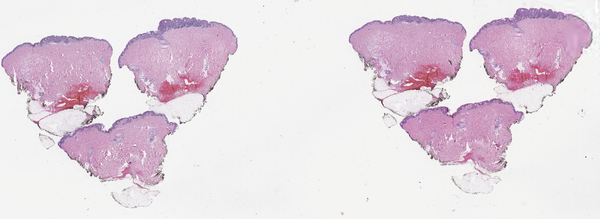

Supplement: S2 Dataset — (ZIP) [file pone.0297146.s008.zip › HE/556823_HE.png]

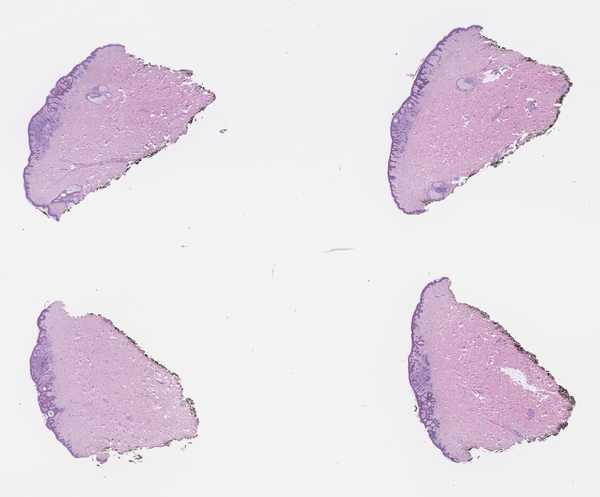

Supplement: S2 Dataset — (ZIP) [file pone.0297146.s008.zip › HE/118092_HE.png]

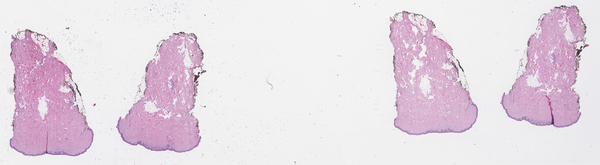

Supplement: S2 Dataset — (ZIP) [file pone.0297146.s008.zip › HE/230812_HE.png]

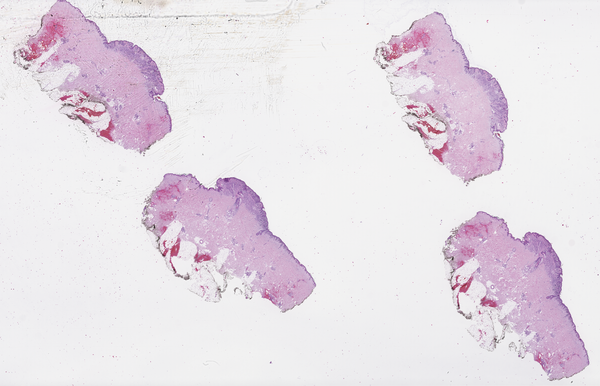

Supplement: S2 Dataset — (ZIP) [file pone.0297146.s008.zip › HE/809379_HE.png]

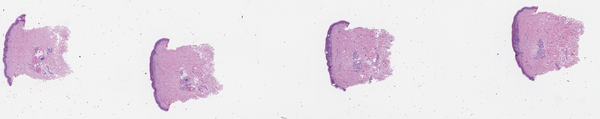

Supplement: S2 Dataset — (ZIP) [file pone.0297146.s008.zip › HE/329337_HE.png]

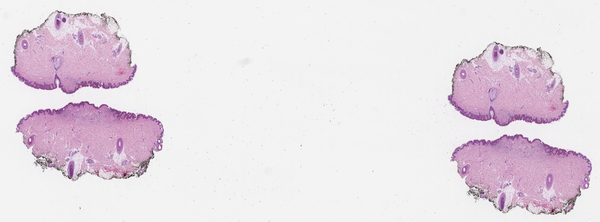

Supplement: S2 Dataset — (ZIP) [file pone.0297146.s008.zip › HE/437272_HE.png]

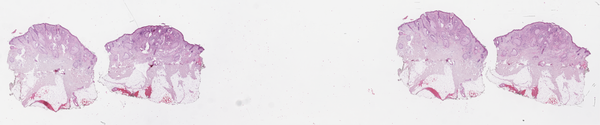

Supplement: S2 Dataset — (ZIP) [file pone.0297146.s008.zip › HE/680443_HE.png]

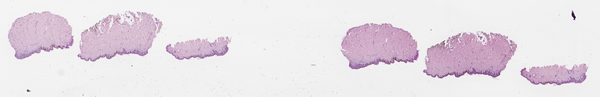

Supplement: S2 Dataset — (ZIP) [file pone.0297146.s008.zip › HE/542754-2_HE.png]

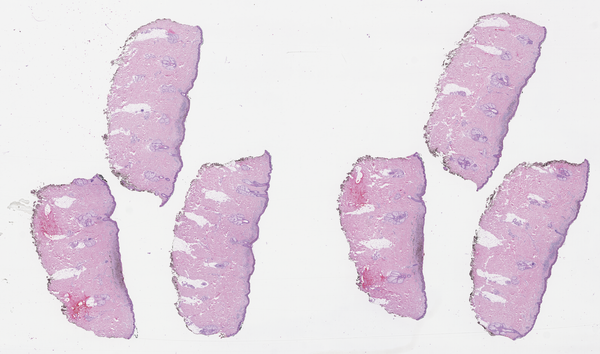

Supplement: S2 Dataset — (ZIP) [file pone.0297146.s008.zip › HE/761124_HE.png]

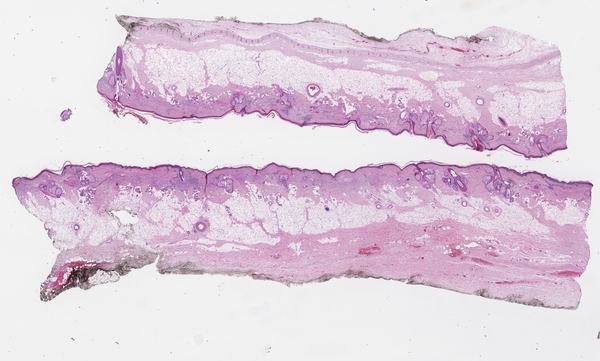

Supplement: S2 Dataset — (ZIP) [file pone.0297146.s008.zip › HE/777041_HE.png]

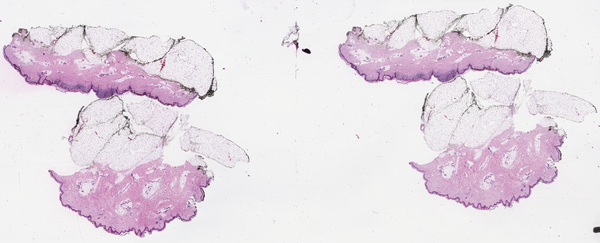

Supplement: S2 Dataset — (ZIP) [file pone.0297146.s008.zip › HE/284284_HE.png]

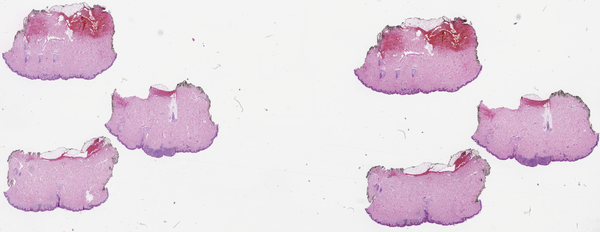

Supplement: S2 Dataset — (ZIP) [file pone.0297146.s008.zip › HE/430532_HE.png]

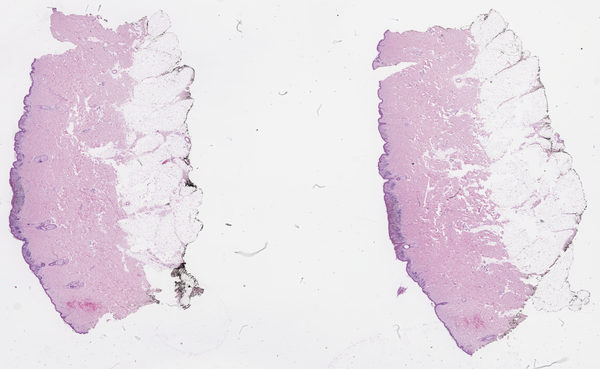

Supplement: S2 Dataset — (ZIP) [file pone.0297146.s008.zip › HE/293101_HE.png]

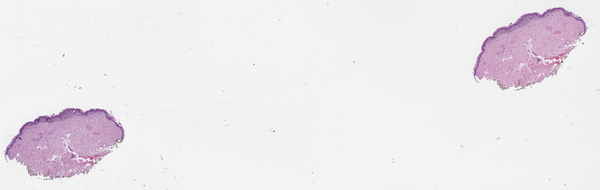

Supplement: S2 Dataset — (ZIP) [file pone.0297146.s008.zip › HE/484480_HE.png]

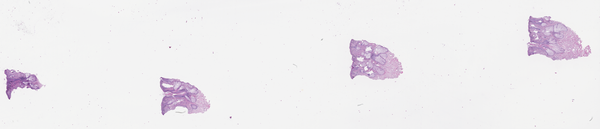

Supplement: S2 Dataset — (ZIP) [file pone.0297146.s008.zip › HE/697339_HE.png]

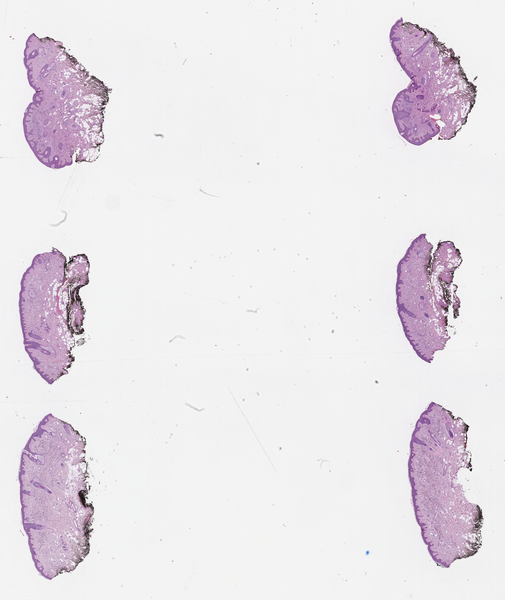

Supplement: S2 Dataset — (ZIP) [file pone.0297146.s008.zip › HE/300473_HE.png]

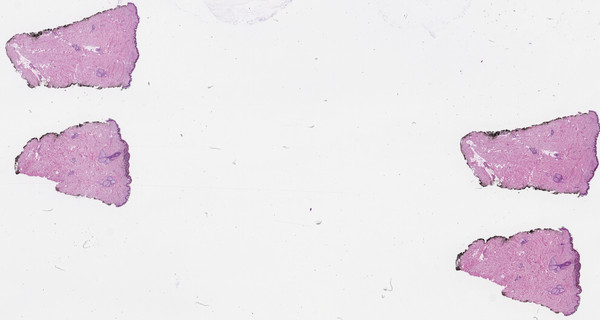

Supplement: S2 Dataset — (ZIP) [file pone.0297146.s008.zip › HE/425593-1_HE.png]

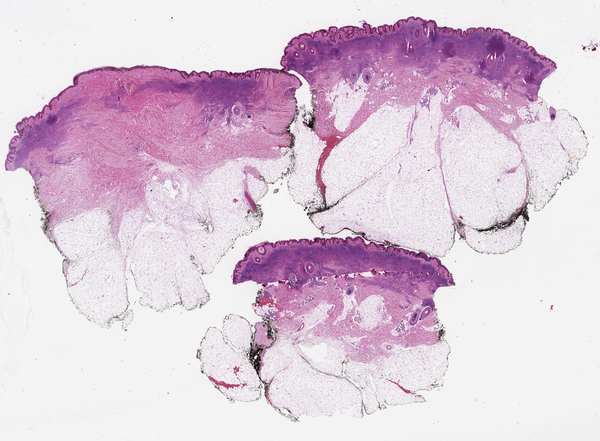

Supplement: S2 Dataset — (ZIP) [file pone.0297146.s008.zip › HE/482540_HE.png]

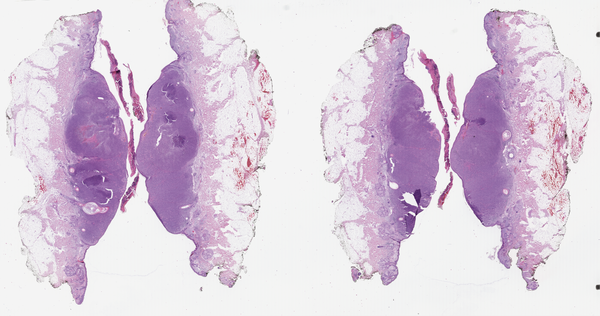

Supplement: S2 Dataset — (ZIP) [file pone.0297146.s008.zip › HE/745192-1_HE.png]

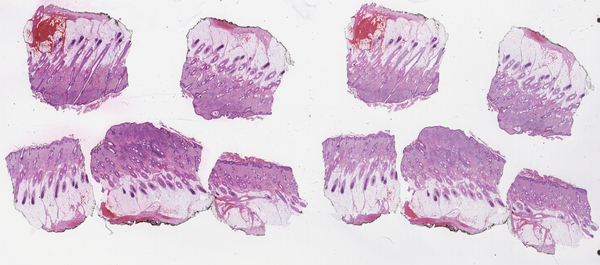

Supplement: S2 Dataset — (ZIP) [file pone.0297146.s008.zip › HE/546569-2_HE.png]

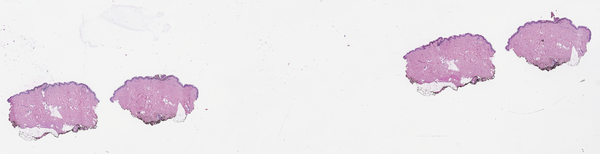

Supplement: S2 Dataset — (ZIP) [file pone.0297146.s008.zip › HE/823353_HE.png]

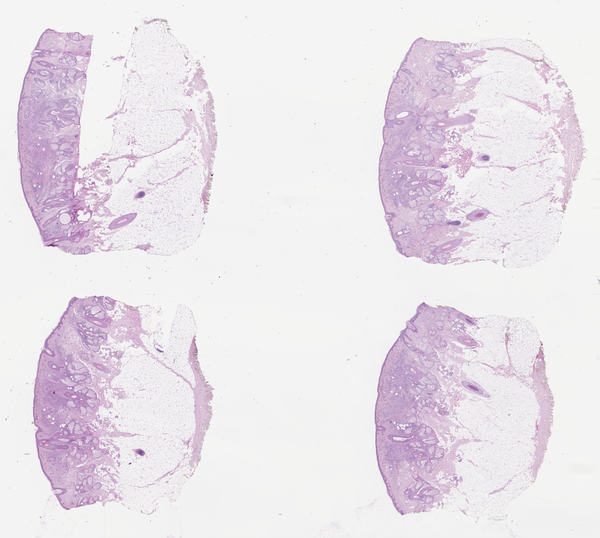

Supplement: S2 Dataset — (ZIP) [file pone.0297146.s008.zip › HE/667443_HE.png]

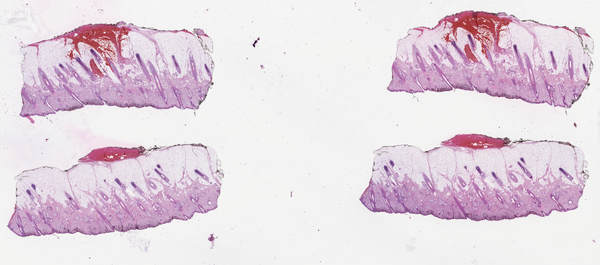

Supplement: S2 Dataset — (ZIP) [file pone.0297146.s008.zip › HE/583846_HE.png]

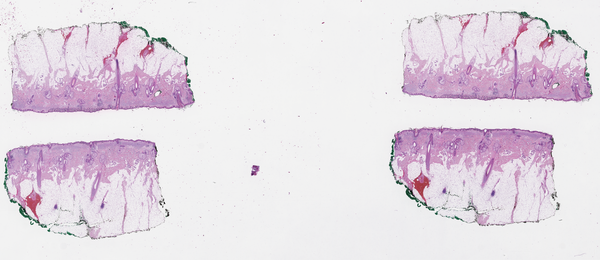

Supplement: S2 Dataset — (ZIP) [file pone.0297146.s008.zip › HE/431691-2_HE.png]

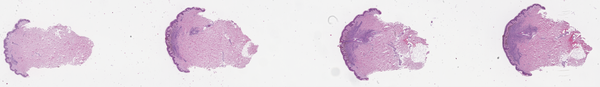

Supplement: S2 Dataset — (ZIP) [file pone.0297146.s008.zip › HE/314572-2_HE.png]

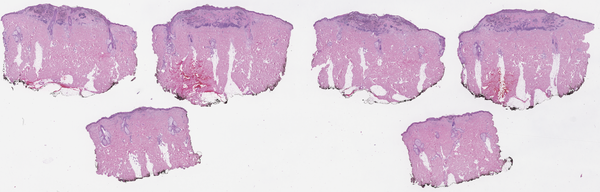

Supplement: S2 Dataset — (ZIP) [file pone.0297146.s008.zip › HE/487457_HE.png]

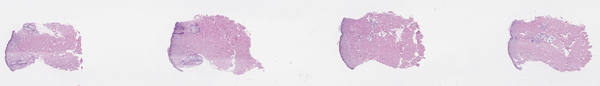

Supplement: S2 Dataset — (ZIP) [file pone.0297146.s008.zip › HE/502423_HE.png]

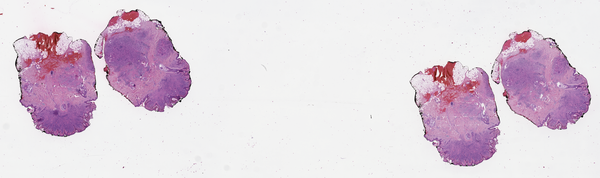

Supplement: S2 Dataset — (ZIP) [file pone.0297146.s008.zip › HE/336057_HE.png]

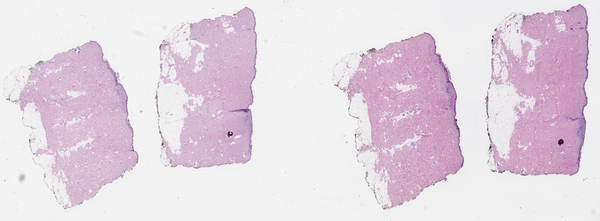

Supplement: S2 Dataset — (ZIP) [file pone.0297146.s008.zip › HE/411147_HE.png]

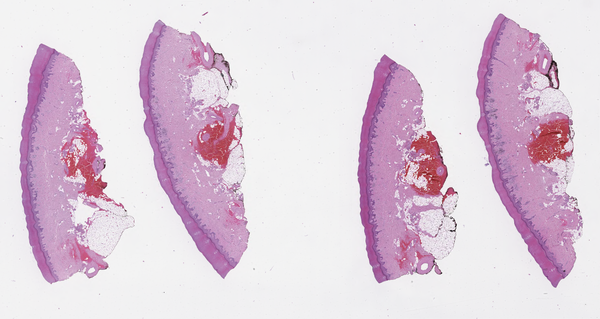

Supplement: S2 Dataset — (ZIP) [file pone.0297146.s008.zip › HE/334622_HE.png]

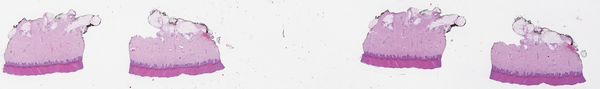

Supplement: S2 Dataset — (ZIP) [file pone.0297146.s008.zip › HE/450912-2_HE.png]

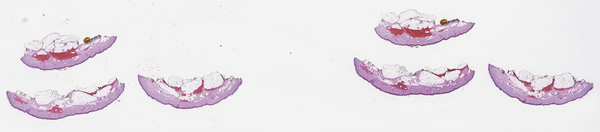

Supplement: S2 Dataset — (ZIP) [file pone.0297146.s008.zip › HE/792992_HE.png]

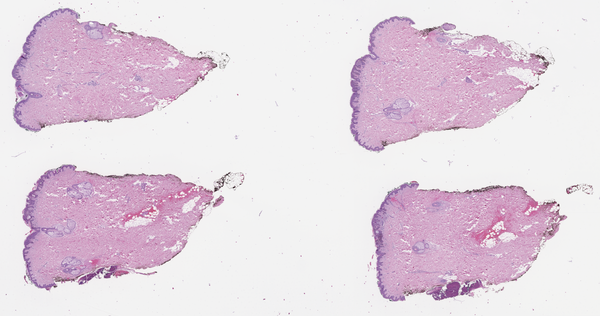

Supplement: S2 Dataset — (ZIP) [file pone.0297146.s008.zip › HE/407837_HE.png]

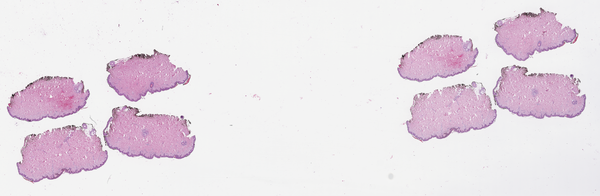

Supplement: S2 Dataset — (ZIP) [file pone.0297146.s008.zip › HE/550664-2_HE.png]

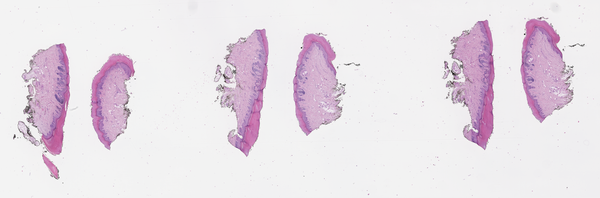

Supplement: S2 Dataset — (ZIP) [file pone.0297146.s008.zip › HE/713600_HE.png]

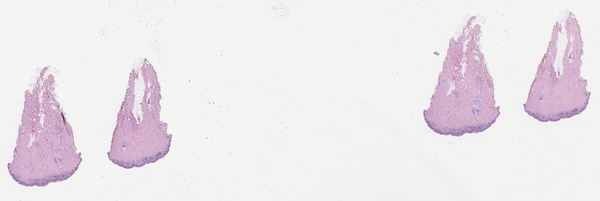

Supplement: S2 Dataset — (ZIP) [file pone.0297146.s008.zip › HE/105587_HE.png]

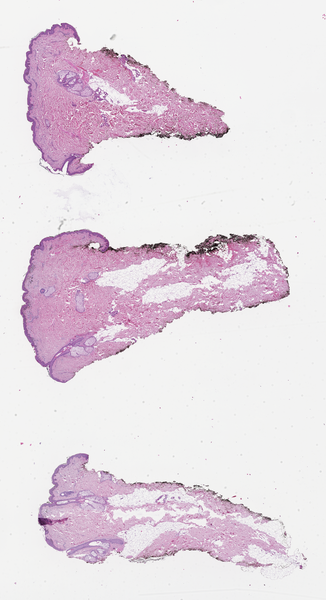

Supplement: S2 Dataset — (ZIP) [file pone.0297146.s008.zip › HE/483191_HE.png]

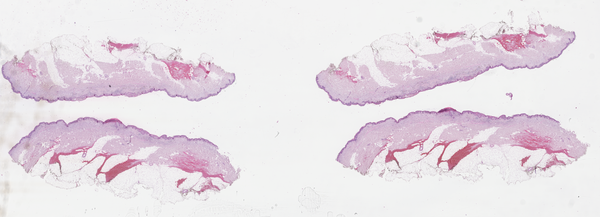

Supplement: S2 Dataset — (ZIP) [file pone.0297146.s008.zip › HE/678133_HE.png]

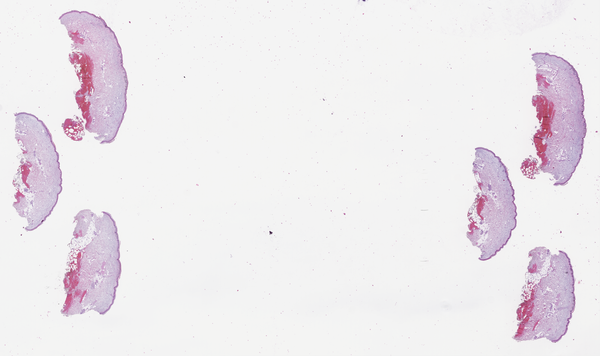

Supplement: S2 Dataset — (ZIP) [file pone.0297146.s008.zip › HE/663974_HE.png]

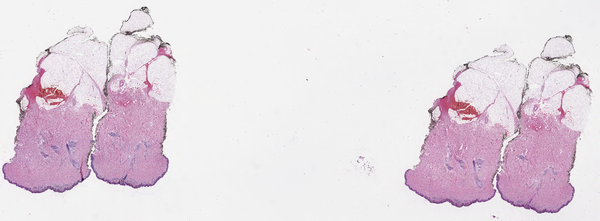

Supplement: S2 Dataset — (ZIP) [file pone.0297146.s008.zip › HE/550193-1_HE.png]

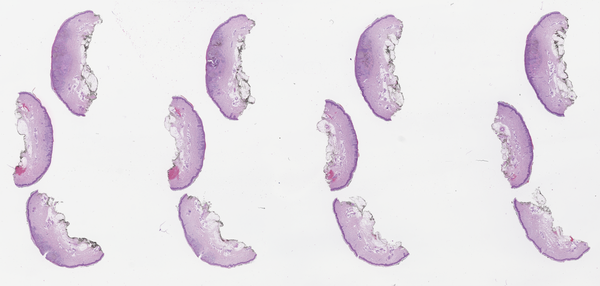

Supplement: S2 Dataset — (ZIP) [file pone.0297146.s008.zip › HE/699110_HE.png]

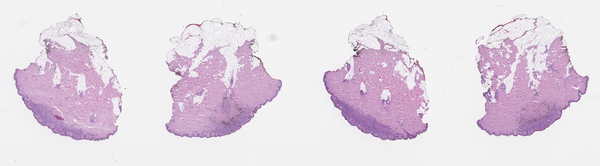

Supplement: S2 Dataset — (ZIP) [file pone.0297146.s008.zip › HE/531946_HE.png]

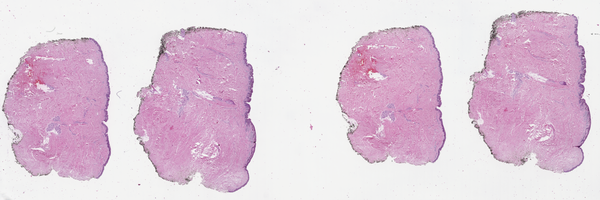

Supplement: S2 Dataset — (ZIP) [file pone.0297146.s008.zip › HE/473038_HE.png]

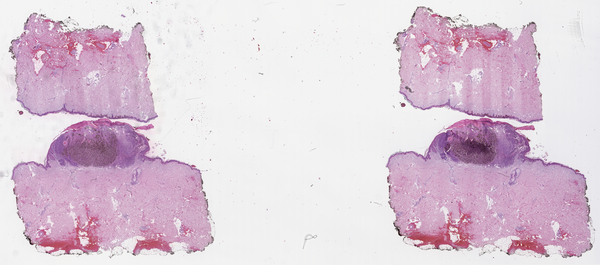

Supplement: S2 Dataset — (ZIP) [file pone.0297146.s008.zip › HE/303141_HE.png]

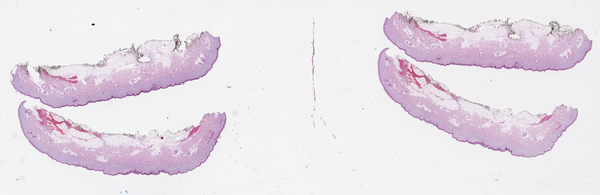

Supplement: S2 Dataset — (ZIP) [file pone.0297146.s008.zip › HE/608216_HE.png]

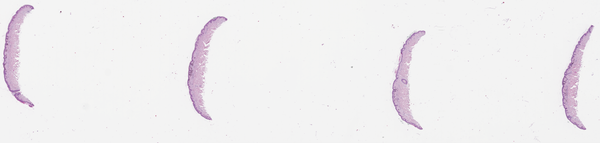

Supplement: S2 Dataset — (ZIP) [file pone.0297146.s008.zip › HE/431691-1_HE.png]

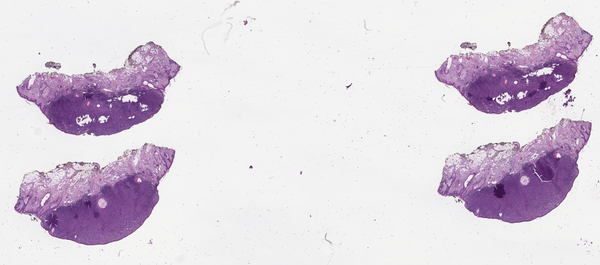

Supplement: S2 Dataset — (ZIP) [file pone.0297146.s008.zip › HE/853282-2_HE.png]

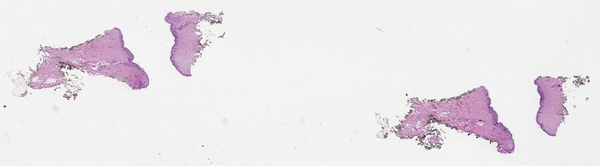

Supplement: S2 Dataset — (ZIP) [file pone.0297146.s008.zip › HE/409386-2_HE.png]

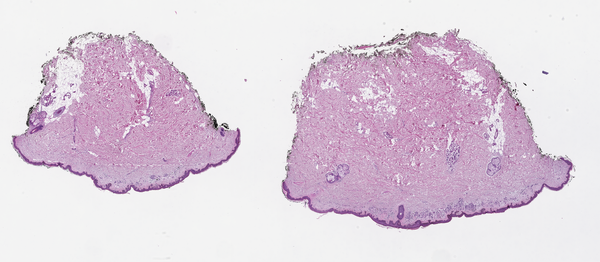

Supplement: S2 Dataset — (ZIP) [file pone.0297146.s008.zip › HE/446684_HE.png]

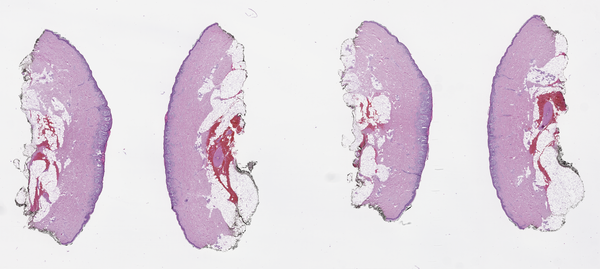

Supplement: S2 Dataset — (ZIP) [file pone.0297146.s008.zip › HE/849969-1_HE.png]

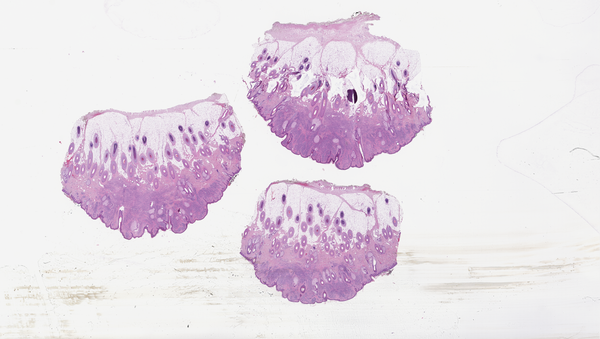

Supplement: S2 Dataset — (ZIP) [file pone.0297146.s008.zip › HE/691272_HE.png]

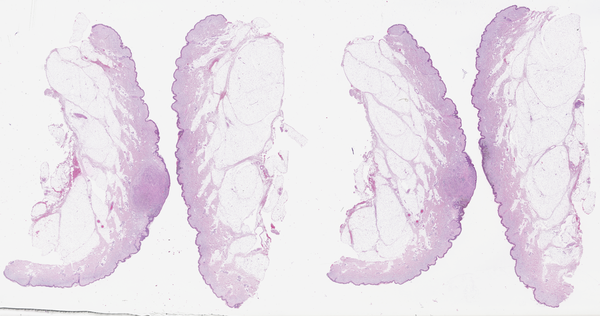

Supplement: S2 Dataset — (ZIP) [file pone.0297146.s008.zip › HE/643765_HE.png]

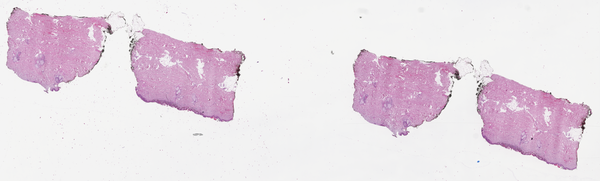

Supplement: S2 Dataset — (ZIP) [file pone.0297146.s008.zip › HE/570214_HE.png]

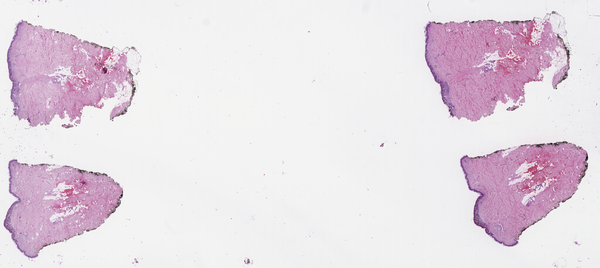

Supplement: S2 Dataset — (ZIP) [file pone.0297146.s008.zip › HE/401945-2_HE.png]

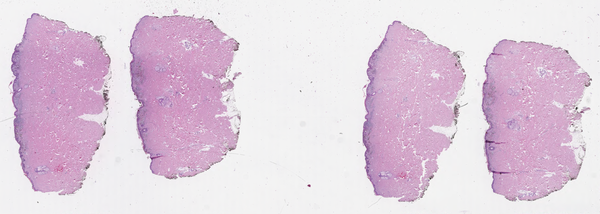

Supplement: S2 Dataset — (ZIP) [file pone.0297146.s008.zip › HE/213658_HE.png]

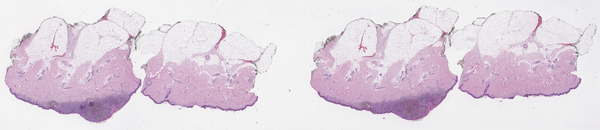

Supplement: S2 Dataset — (ZIP) [file pone.0297146.s008.zip › HE/250459_HE.png]

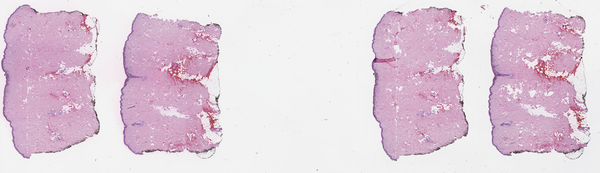

Supplement: S2 Dataset — (ZIP) [file pone.0297146.s008.zip › HE/247307_HE.png]

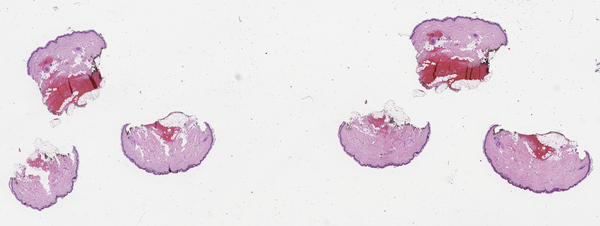

Supplement: S2 Dataset — (ZIP) [file pone.0297146.s008.zip › HE/792025_HE.png]

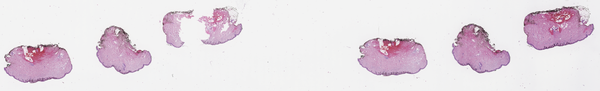

Supplement: S2 Dataset — (ZIP) [file pone.0297146.s008.zip › HE/470624_HE.png]

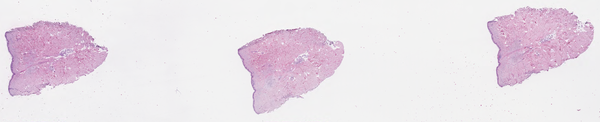

Supplement: S2 Dataset — (ZIP) [file pone.0297146.s008.zip › HE/233282_HE.png]

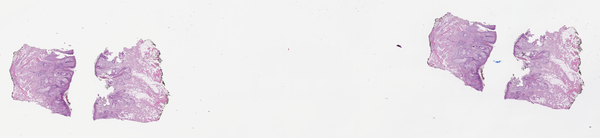

Supplement: S2 Dataset — (ZIP) [file pone.0297146.s008.zip › HE/559318_HE.png]
