# Supplementary figures and images for: Evaluating deep learning-based melanoma classification using immunohistochemistry and routine histology: A three center study (part 6 of 7)
Source: PLoS One. 2024 Jan 19;19(1):e0297146. doi: 10.1371/journal.pone.0297146 (PMC10798511; doi:10.1371/journal.pone.0297146)

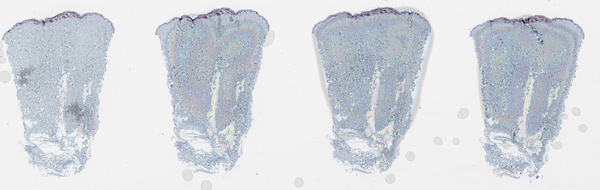

Supplement: S3 Dataset — (ZIP) [file pone.0297146.s009.zip › erlangen/MelanA/290858-1_MelanA.png]

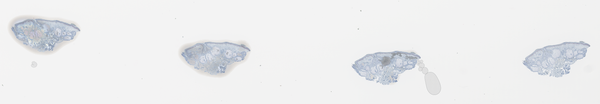

Supplement: S3 Dataset — (ZIP) [file pone.0297146.s009.zip › erlangen/MelanA/157765_MelanA.png]

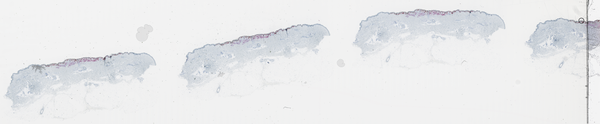

Supplement: S3 Dataset — (ZIP) [file pone.0297146.s009.zip › erlangen/MelanA/419114_MelanA.png]

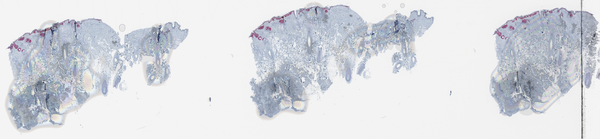

Supplement: S3 Dataset — (ZIP) [file pone.0297146.s009.zip › erlangen/MelanA/186342-A_MelanA.png]

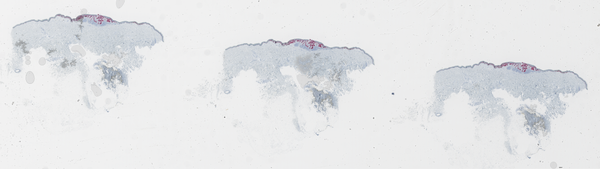

Supplement: S3 Dataset — (ZIP) [file pone.0297146.s009.zip › erlangen/MelanA/439062-B_MelanA.png]

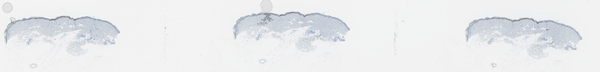

Supplement: S3 Dataset — (ZIP) [file pone.0297146.s009.zip › erlangen/MelanA/217214_MelanA.png]

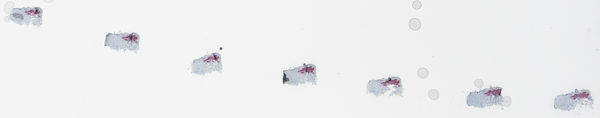

Supplement: S3 Dataset — (ZIP) [file pone.0297146.s009.zip › erlangen/MelanA/268475_MelanA.png]

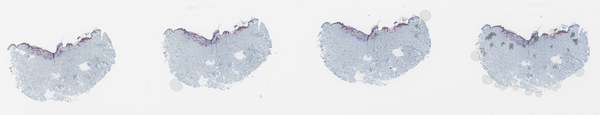

Supplement: S3 Dataset — (ZIP) [file pone.0297146.s009.zip › erlangen/MelanA/212352-B_MelanA.png]

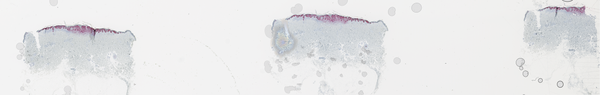

Supplement: S3 Dataset — (ZIP) [file pone.0297146.s009.zip › erlangen/MelanA/112823_MelanA.png]

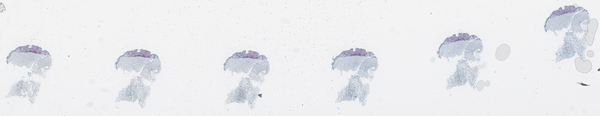

Supplement: S3 Dataset — (ZIP) [file pone.0297146.s009.zip › erlangen/MelanA/185775-2_MelanA.png]

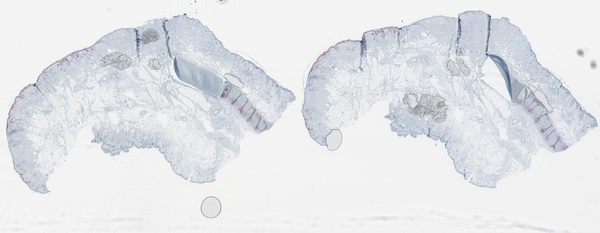

Supplement: S3 Dataset — (ZIP) [file pone.0297146.s009.zip › erlangen/MelanA/217797_MelanA.png]

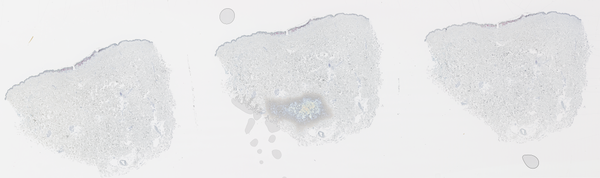

Supplement: S3 Dataset — (ZIP) [file pone.0297146.s009.zip › erlangen/MelanA/144637-A_MelanA.png]

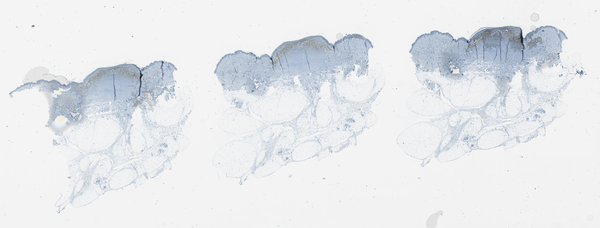

Supplement: S3 Dataset — (ZIP) [file pone.0297146.s009.zip › erlangen/MelanA/217052_MelanA.png]

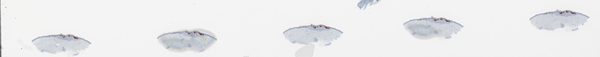

Supplement: S3 Dataset — (ZIP) [file pone.0297146.s009.zip › erlangen/MelanA/104888_MelanA.png]

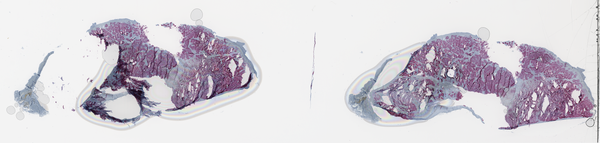

Supplement: S3 Dataset — (ZIP) [file pone.0297146.s009.zip › erlangen/MelanA/226010-A_MelanA.png]

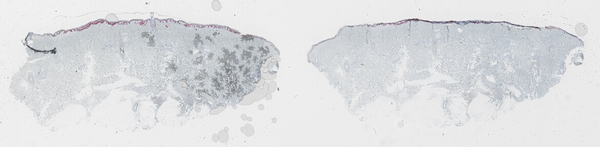

Supplement: S3 Dataset — (ZIP) [file pone.0297146.s009.zip › erlangen/MelanA/375136-A_MelanA.png]

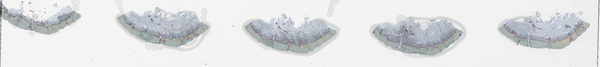

Supplement: S3 Dataset — (ZIP) [file pone.0297146.s009.zip › erlangen/MelanA/100111_MelanA.png]

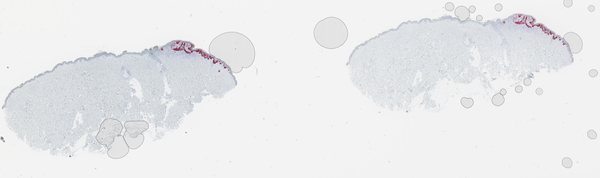

Supplement: S3 Dataset — (ZIP) [file pone.0297146.s009.zip › erlangen/MelanA/521208_MelanA.png]

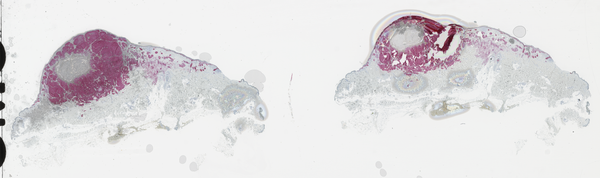

Supplement: S3 Dataset — (ZIP) [file pone.0297146.s009.zip › erlangen/MelanA/125801_MelanA.png]

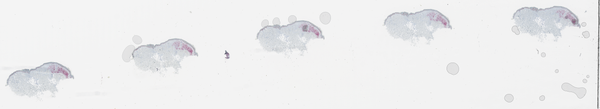

Supplement: S3 Dataset — (ZIP) [file pone.0297146.s009.zip › erlangen/MelanA/440663_MelanA.png]

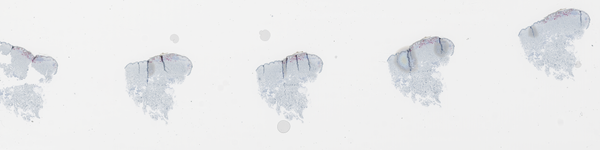

Supplement: S3 Dataset — (ZIP) [file pone.0297146.s009.zip › erlangen/MelanA/458014_MelanA.png]

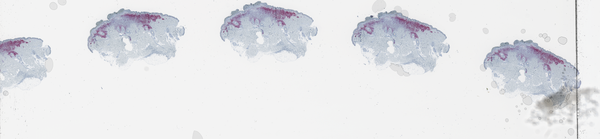

Supplement: S3 Dataset — (ZIP) [file pone.0297146.s009.zip › erlangen/MelanA/453916_MelanA.png]

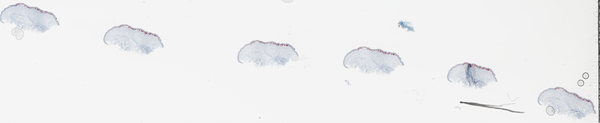

Supplement: S3 Dataset — (ZIP) [file pone.0297146.s009.zip › erlangen/MelanA/400143-1_MelanA.png]

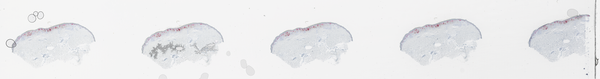

Supplement: S3 Dataset — (ZIP) [file pone.0297146.s009.zip › erlangen/MelanA/496323-A_MelanA.png]

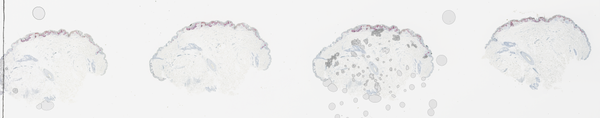

Supplement: S3 Dataset — (ZIP) [file pone.0297146.s009.zip › erlangen/MelanA/137491_MelanA.png]

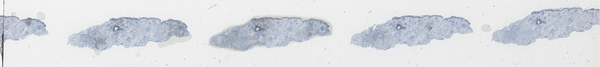

Supplement: S3 Dataset — (ZIP) [file pone.0297146.s009.zip › erlangen/MelanA/343276_MelanA.png]

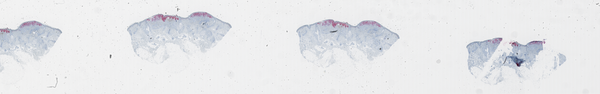

Supplement: S3 Dataset — (ZIP) [file pone.0297146.s009.zip › erlangen/MelanA/314706-1_MelanA.png]

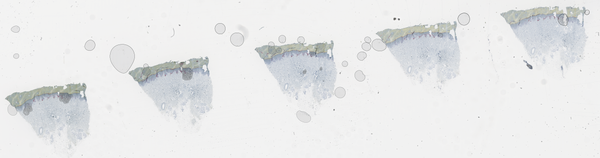

Supplement: S3 Dataset — (ZIP) [file pone.0297146.s009.zip › erlangen/MelanA/189769_MelanA.png]

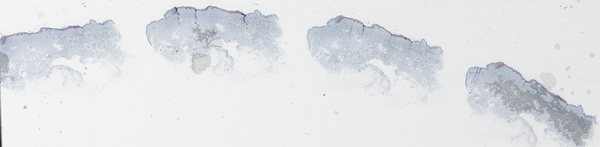

Supplement: S3 Dataset — (ZIP) [file pone.0297146.s009.zip › erlangen/MelanA/373976_MelanA.png]

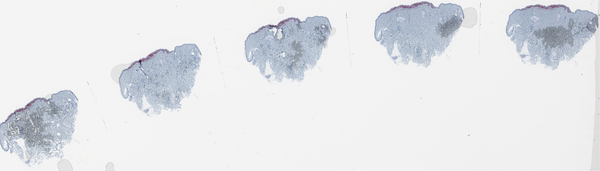

Supplement: S3 Dataset — (ZIP) [file pone.0297146.s009.zip › erlangen/MelanA/225837_MelanA.png]

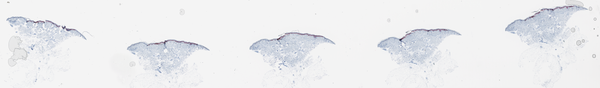

Supplement: S3 Dataset — (ZIP) [file pone.0297146.s009.zip › erlangen/MelanA/337110_MelanA.png]

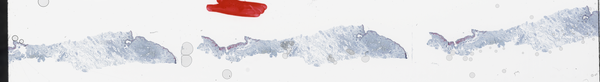

Supplement: S3 Dataset — (ZIP) [file pone.0297146.s009.zip › erlangen/MelanA/368694_MelanA.png]

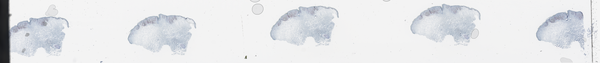

Supplement: S3 Dataset — (ZIP) [file pone.0297146.s009.zip › erlangen/MelanA/269003_MelanA.png]

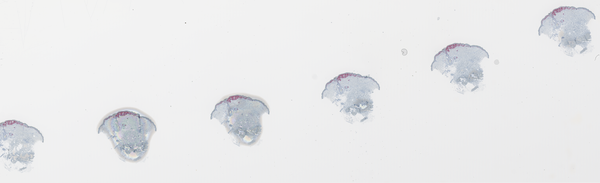

Supplement: S3 Dataset — (ZIP) [file pone.0297146.s009.zip › erlangen/MelanA/150297_MelanA.png]

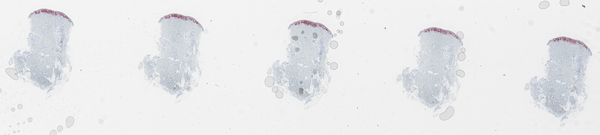

Supplement: S3 Dataset — (ZIP) [file pone.0297146.s009.zip › erlangen/MelanA/268152_MelanA.png]

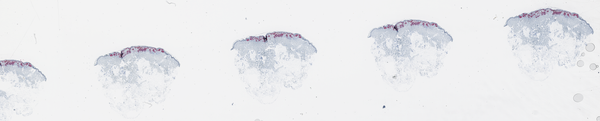

Supplement: S3 Dataset — (ZIP) [file pone.0297146.s009.zip › erlangen/MelanA/232317_MelanA.png]

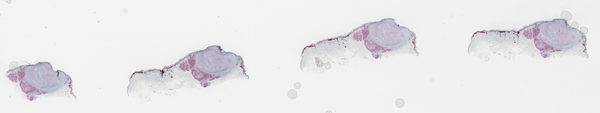

Supplement: S3 Dataset — (ZIP) [file pone.0297146.s009.zip › erlangen/MelanA/343178_MelanA.png]

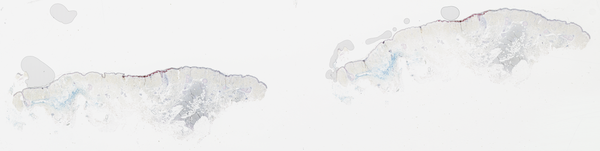

Supplement: S3 Dataset — (ZIP) [file pone.0297146.s009.zip › erlangen/MelanA/195837-A_MelanA.png]

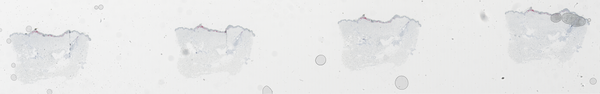

Supplement: S3 Dataset — (ZIP) [file pone.0297146.s009.zip › erlangen/MelanA/129216-A_MelanA.png]

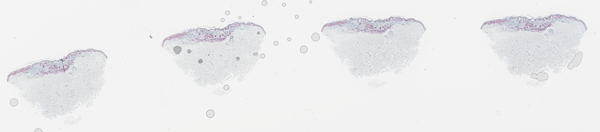

Supplement: S3 Dataset — (ZIP) [file pone.0297146.s009.zip › erlangen/MelanA/466480_MelanA.png]

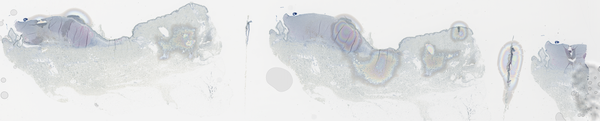

Supplement: S3 Dataset — (ZIP) [file pone.0297146.s009.zip › erlangen/MelanA/166949_MelanA.png]

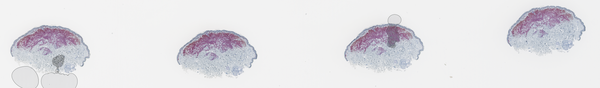

Supplement: S3 Dataset — (ZIP) [file pone.0297146.s009.zip › erlangen/MelanA/213578_MelanA.png]

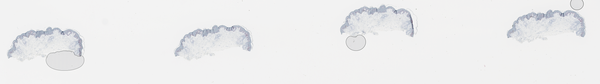

Supplement: S3 Dataset — (ZIP) [file pone.0297146.s009.zip › erlangen/MelanA/325002_MelanA.png]

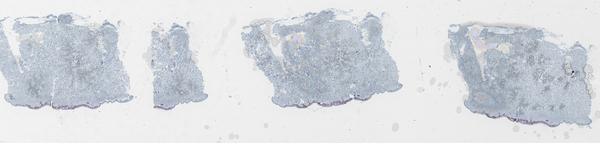

Supplement: S3 Dataset — (ZIP) [file pone.0297146.s009.zip › erlangen/MelanA/363877_MelanA.png]

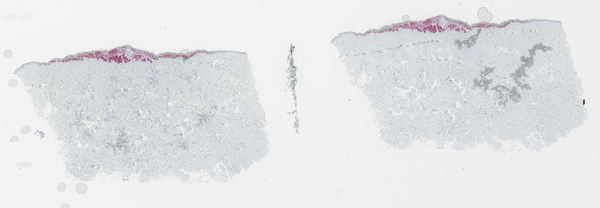

Supplement: S3 Dataset — (ZIP) [file pone.0297146.s009.zip › erlangen/MelanA/454218_MelanA.png]

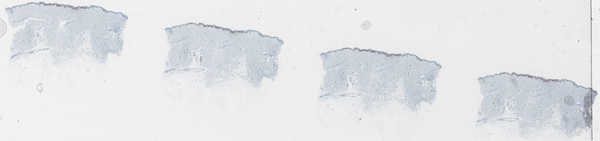

Supplement: S3 Dataset — (ZIP) [file pone.0297146.s009.zip › erlangen/MelanA/312697_MelanA.png]

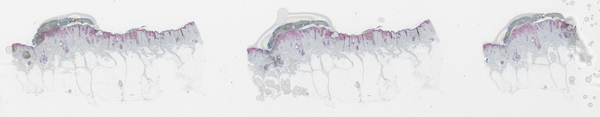

Supplement: S3 Dataset — (ZIP) [file pone.0297146.s009.zip › erlangen/MelanA/122032_MelanA.png]

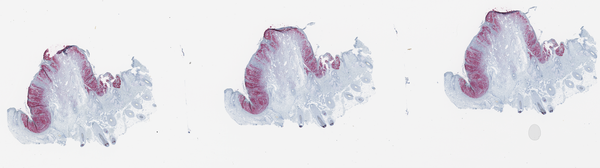

Supplement: S3 Dataset — (ZIP) [file pone.0297146.s009.zip › erlangen/MelanA/314706-2_MelanA.png]

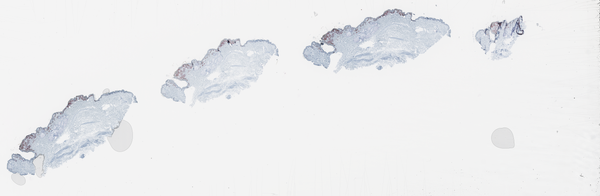

Supplement: S3 Dataset — (ZIP) [file pone.0297146.s009.zip › erlangen/MelanA/206605_MelanA.png]

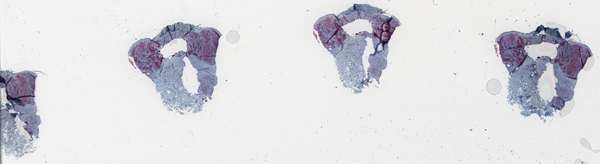

Supplement: S3 Dataset — (ZIP) [file pone.0297146.s009.zip › erlangen/MelanA/353896_MelanA.png]

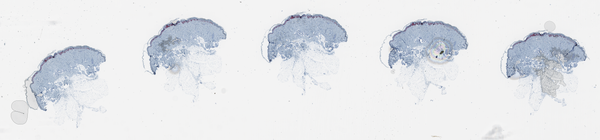

Supplement: S3 Dataset — (ZIP) [file pone.0297146.s009.zip › erlangen/MelanA/269748_MelanA.png]

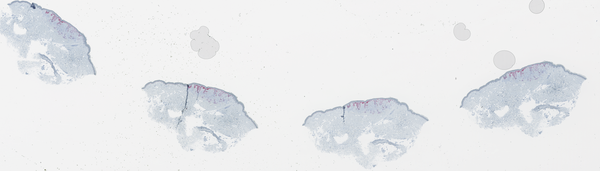

Supplement: S3 Dataset — (ZIP) [file pone.0297146.s009.zip › erlangen/MelanA/478728-B_MelanA.png]

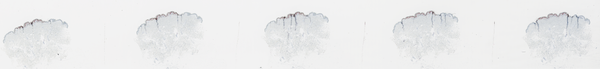

Supplement: S3 Dataset — (ZIP) [file pone.0297146.s009.zip › erlangen/MelanA/255314_MelanA.png]

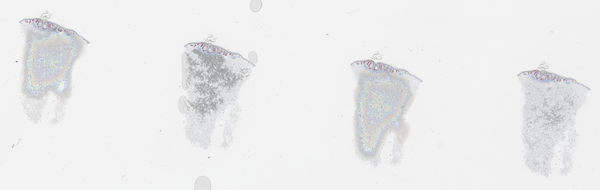

Supplement: S3 Dataset — (ZIP) [file pone.0297146.s009.zip › erlangen/MelanA/281410_MelanA.png]

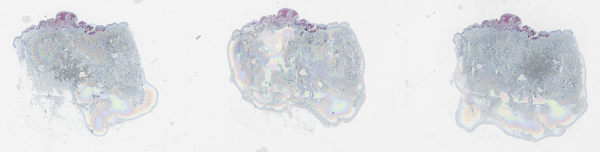

Supplement: S3 Dataset — (ZIP) [file pone.0297146.s009.zip › erlangen/MelanA/275869_MelanA.png]

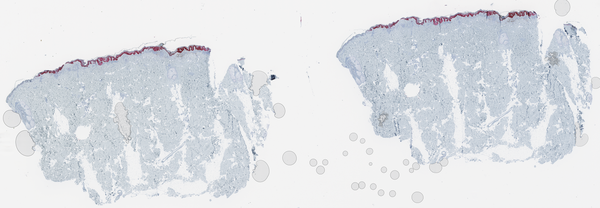

Supplement: S3 Dataset — (ZIP) [file pone.0297146.s009.zip › erlangen/MelanA/511591_MelanA.png]

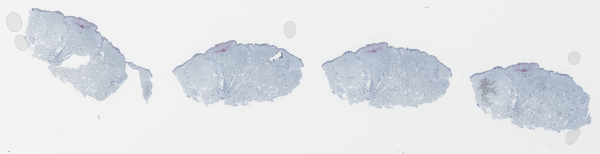

Supplement: S3 Dataset — (ZIP) [file pone.0297146.s009.zip › erlangen/MelanA/210077-2_MelanA.png]

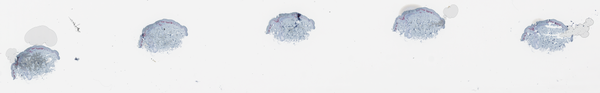

Supplement: S3 Dataset — (ZIP) [file pone.0297146.s009.zip › erlangen/MelanA/228537-2_MelanA.png]

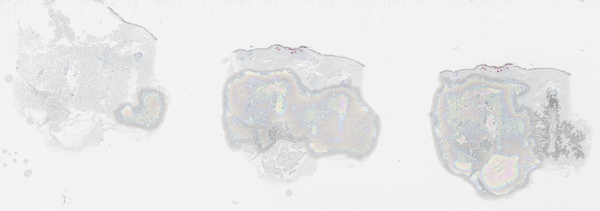

Supplement: S3 Dataset — (ZIP) [file pone.0297146.s009.zip › erlangen/MelanA/455155_MelanA.png]

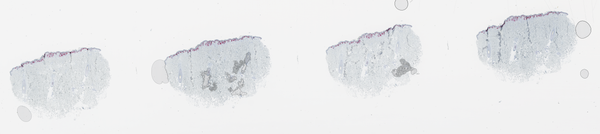

Supplement: S3 Dataset — (ZIP) [file pone.0297146.s009.zip › erlangen/MelanA/370237-1_MelanA.png]

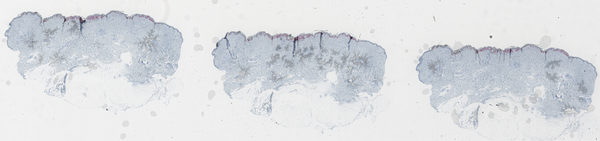

Supplement: S3 Dataset — (ZIP) [file pone.0297146.s009.zip › erlangen/MelanA/344572_MelanA.png]

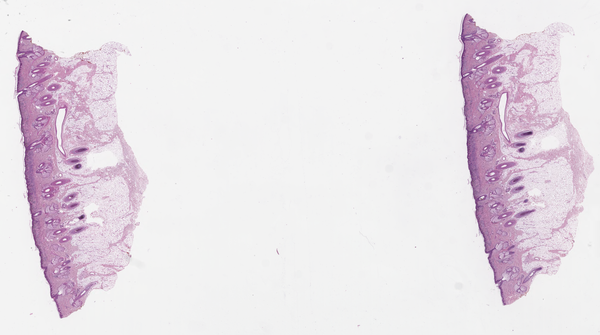

Supplement: S4 Dataset — (ZIP) [file pone.0297146.s010.zip › naples/HE/14743-22_HE.png]

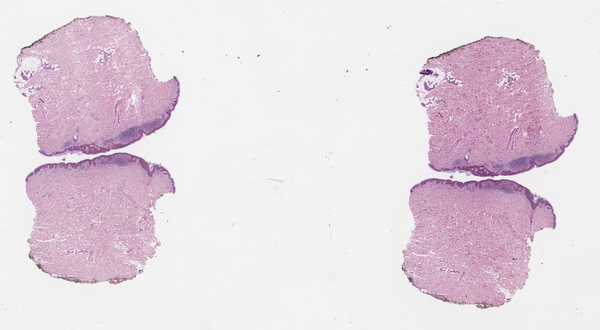

Supplement: S4 Dataset — (ZIP) [file pone.0297146.s010.zip › naples/HE/3245-23_HE.png]

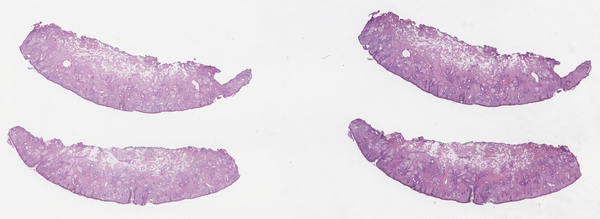

Supplement: S4 Dataset — (ZIP) [file pone.0297146.s010.zip › naples/HE/685-23_HE.png]

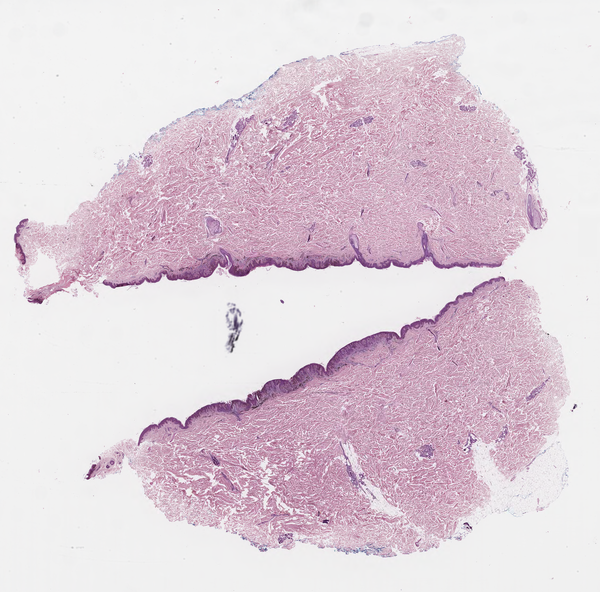

Supplement: S4 Dataset — (ZIP) [file pone.0297146.s010.zip › naples/HE/10811-22_HE.png]

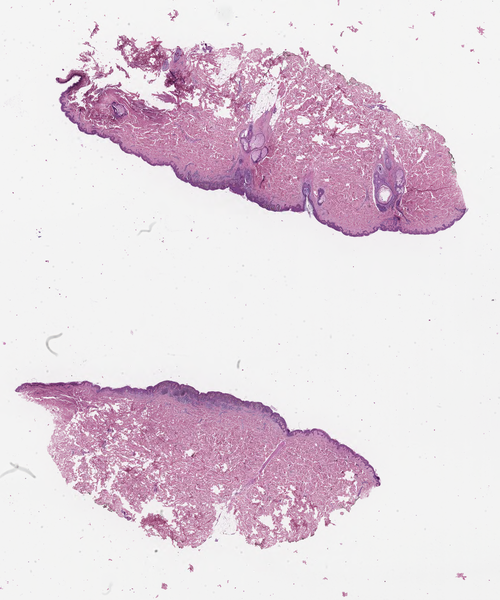

Supplement: S4 Dataset — (ZIP) [file pone.0297146.s010.zip › naples/HE/12939-22_HE.png]

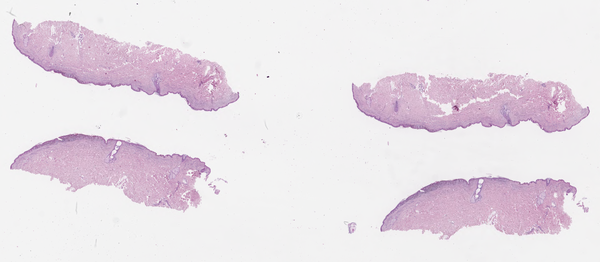

Supplement: S4 Dataset — (ZIP) [file pone.0297146.s010.zip › naples/HE/1832-23_HE.png]

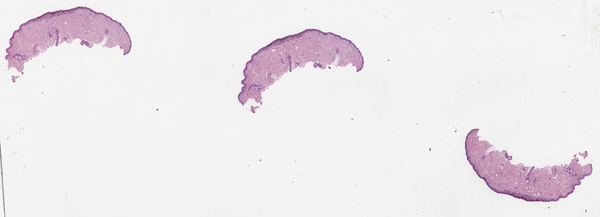

Supplement: S4 Dataset — (ZIP) [file pone.0297146.s010.zip › naples/HE/568-23_HE.png]

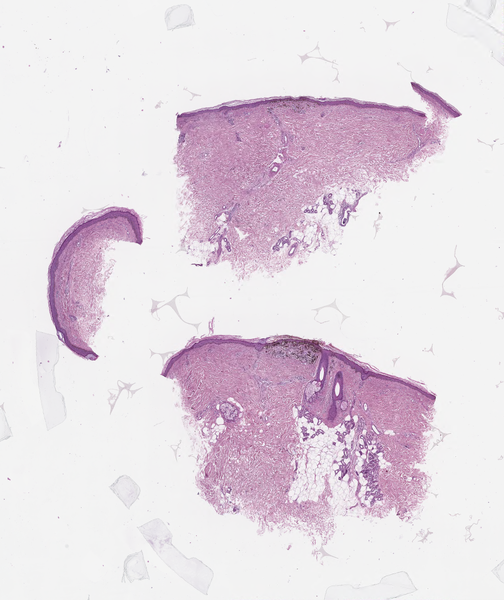

Supplement: S4 Dataset — (ZIP) [file pone.0297146.s010.zip › naples/HE/207-23_HE.png]

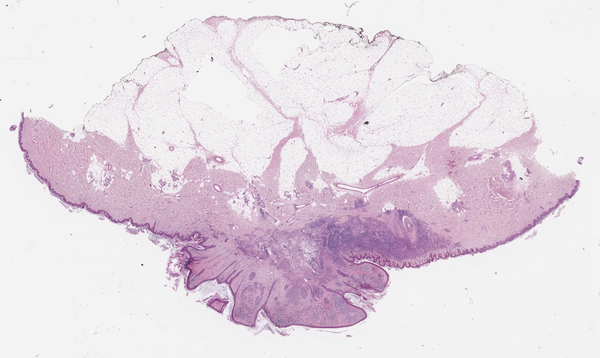

Supplement: S4 Dataset — (ZIP) [file pone.0297146.s010.zip › naples/HE/12242-22_HE.png]

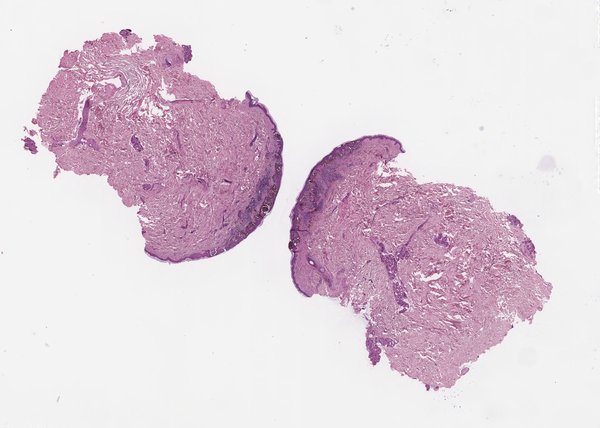

Supplement: S4 Dataset — (ZIP) [file pone.0297146.s010.zip › naples/HE/5438-22_HE.png]

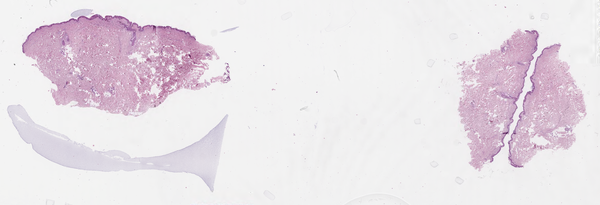

Supplement: S4 Dataset — (ZIP) [file pone.0297146.s010.zip › naples/HE/Y23-A_HE.png]

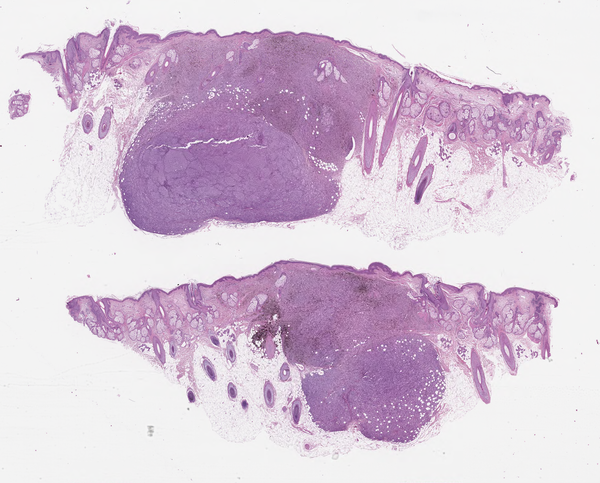

Supplement: S4 Dataset — (ZIP) [file pone.0297146.s010.zip › naples/HE/PM97-23_HE.png]

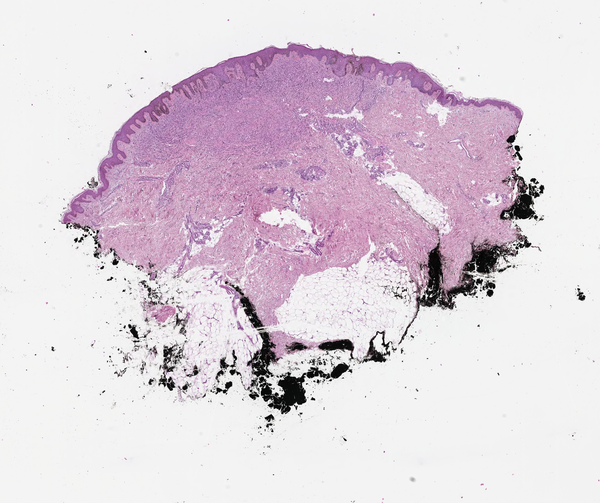

Supplement: S4 Dataset — (ZIP) [file pone.0297146.s010.zip › naples/HE/74-23_HE.png]

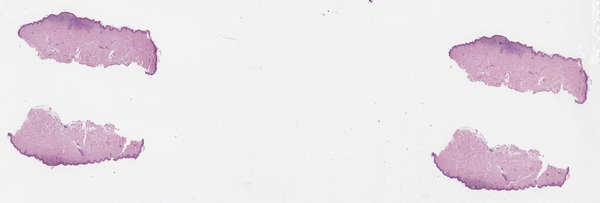

Supplement: S4 Dataset — (ZIP) [file pone.0297146.s010.zip › naples/HE/11586-22_HE.png]

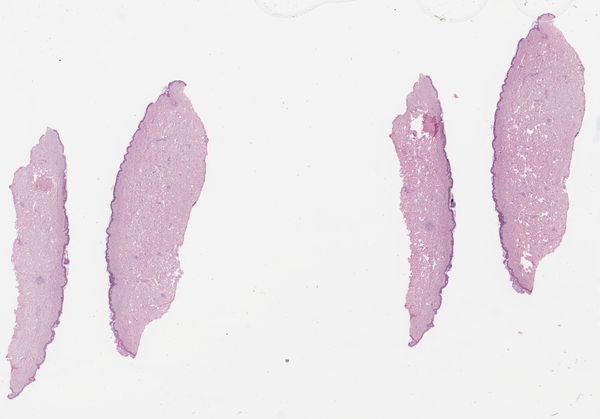

Supplement: S4 Dataset — (ZIP) [file pone.0297146.s010.zip › naples/HE/12315-22_HE.png]

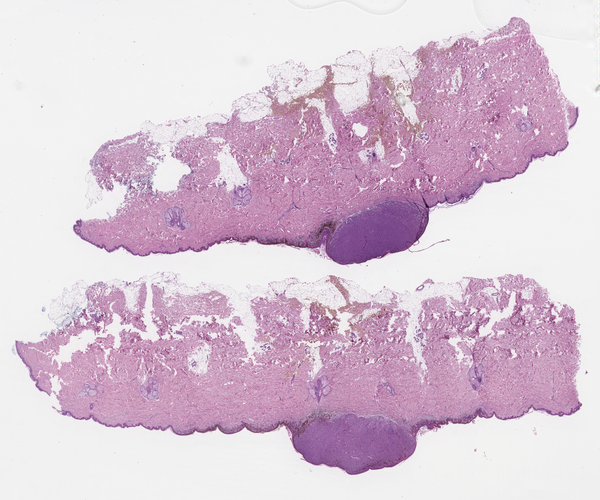

Supplement: S4 Dataset — (ZIP) [file pone.0297146.s010.zip › naples/HE/2313-23_HE.png]

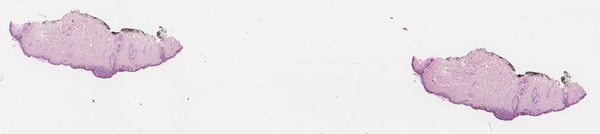

Supplement: S4 Dataset — (ZIP) [file pone.0297146.s010.zip › naples/HE/12257-22_HE.png]

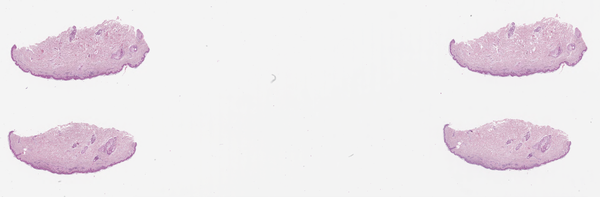

Supplement: S4 Dataset — (ZIP) [file pone.0297146.s010.zip › naples/HE/14744-22-2_HE.png]

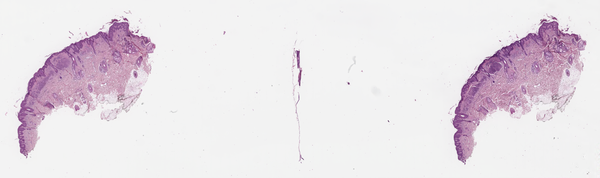

Supplement: S4 Dataset — (ZIP) [file pone.0297146.s010.zip › naples/HE/13969-22_HE.png]

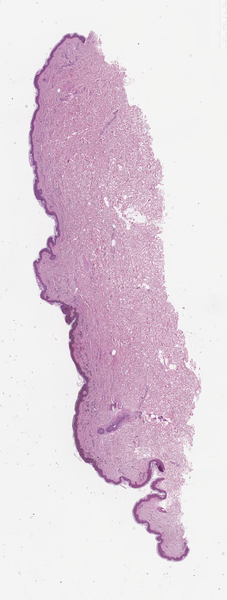

Supplement: S4 Dataset — (ZIP) [file pone.0297146.s010.zip › naples/HE/1210-23_HE.png]

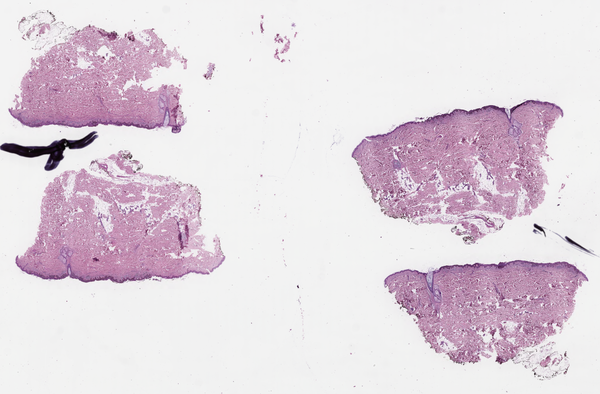

Supplement: S4 Dataset — (ZIP) [file pone.0297146.s010.zip › naples/HE/269-23_HE.png]

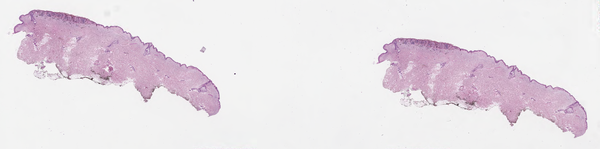

Supplement: S4 Dataset — (ZIP) [file pone.0297146.s010.zip › naples/HE/14202-21_HE.png]

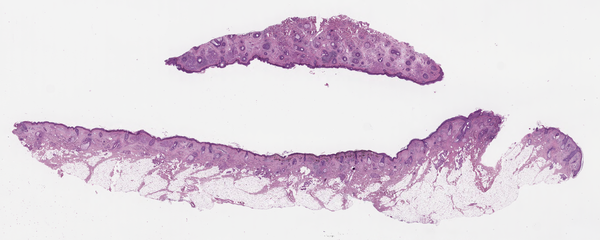

Supplement: S4 Dataset — (ZIP) [file pone.0297146.s010.zip › naples/HE/1127-23_HE.png]

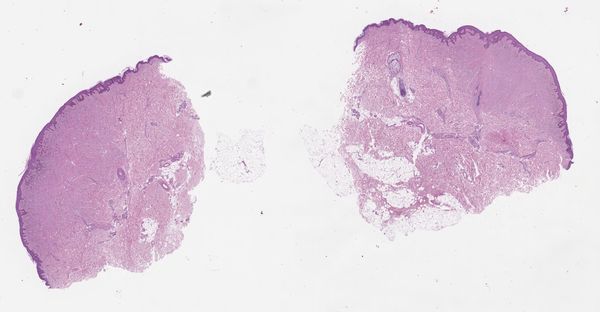

Supplement: S4 Dataset — (ZIP) [file pone.0297146.s010.zip › naples/HE/3232-23_HE.png]

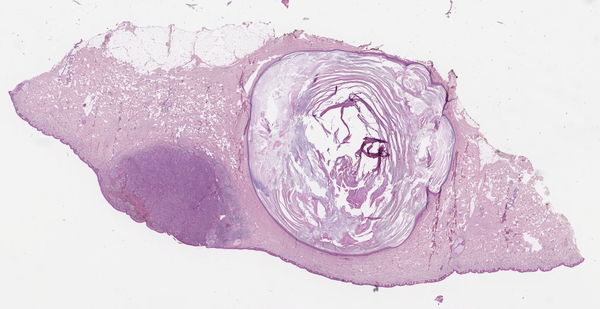

Supplement: S4 Dataset — (ZIP) [file pone.0297146.s010.zip › naples/HE/2363-23_HE.png]

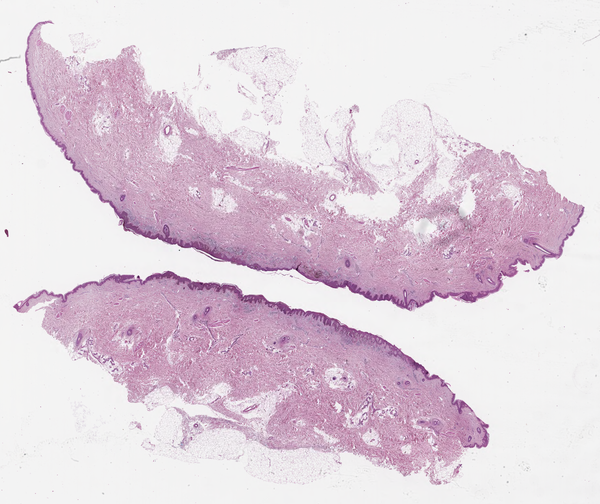

Supplement: S4 Dataset — (ZIP) [file pone.0297146.s010.zip › naples/HE/1231-23_HE.png]

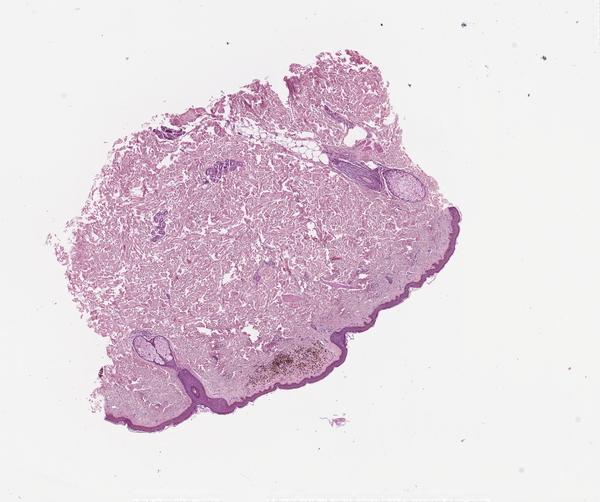

Supplement: S4 Dataset — (ZIP) [file pone.0297146.s010.zip › naples/HE/8808-22_HE.png]

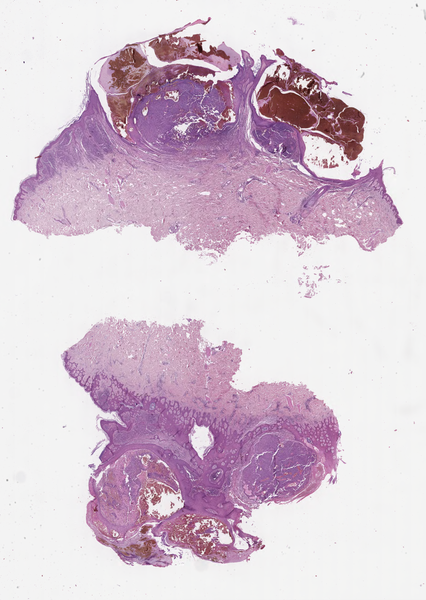

Supplement: S4 Dataset — (ZIP) [file pone.0297146.s010.zip › naples/HE/14151-22_HE.png]

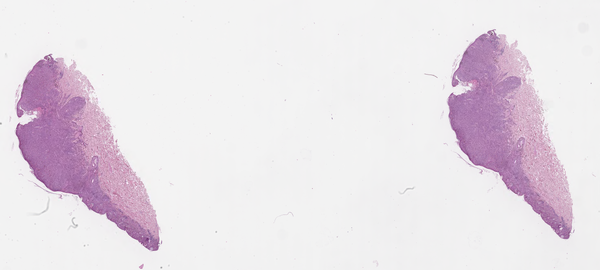

Supplement: S4 Dataset — (ZIP) [file pone.0297146.s010.zip › naples/HE/2412-23_HE.png]

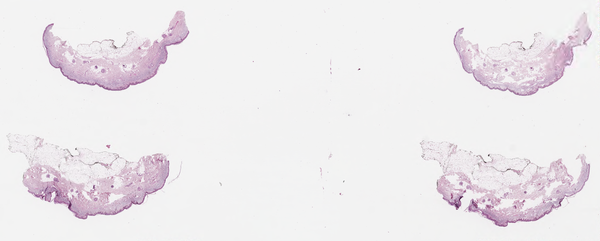

Supplement: S4 Dataset — (ZIP) [file pone.0297146.s010.zip › naples/HE/12250-22_HE.png]

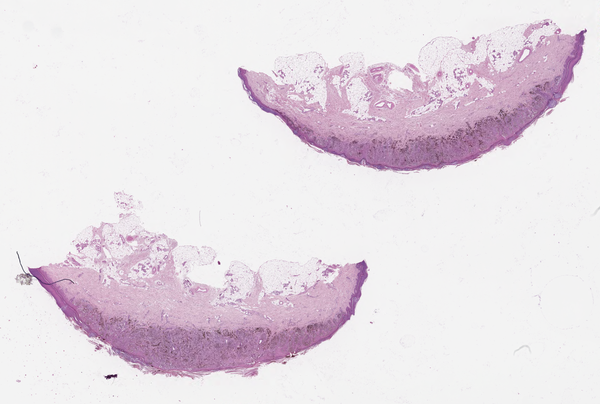

Supplement: S4 Dataset — (ZIP) [file pone.0297146.s010.zip › naples/HE/14645-22_HE.png]

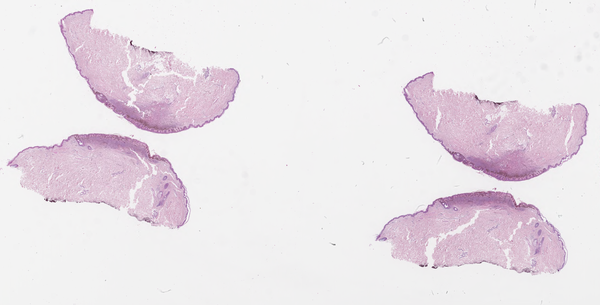

Supplement: S4 Dataset — (ZIP) [file pone.0297146.s010.zip › naples/HE/1363-23_HE.png]

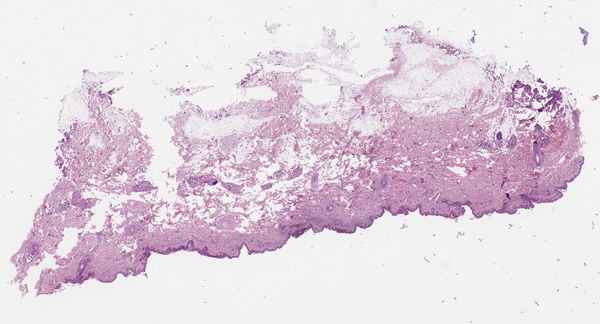

Supplement: S4 Dataset — (ZIP) [file pone.0297146.s010.zip › naples/HE/2550-23_HE.png]

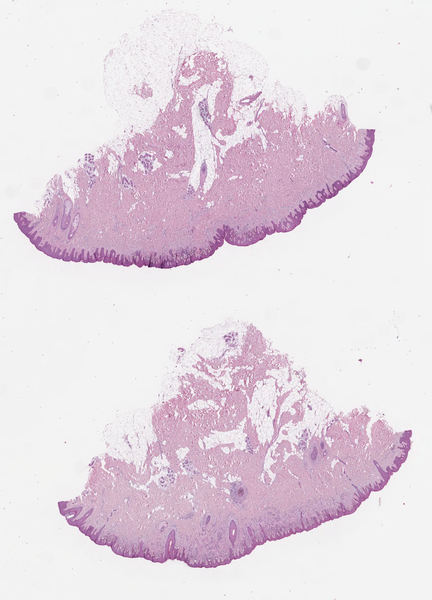

Supplement: S4 Dataset — (ZIP) [file pone.0297146.s010.zip › naples/HE/4023-22_HE.png]

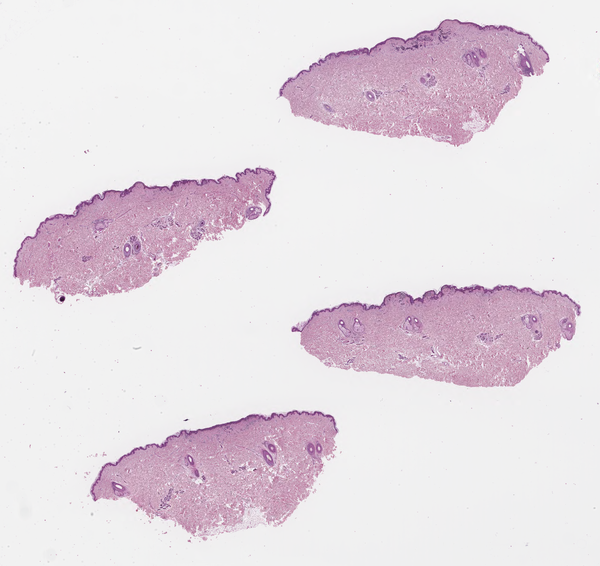

Supplement: S4 Dataset — (ZIP) [file pone.0297146.s010.zip › naples/HE/14744-22-1_HE.png]

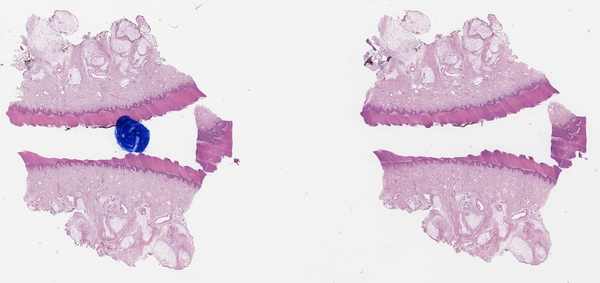

Supplement: S4 Dataset — (ZIP) [file pone.0297146.s010.zip › naples/HE/14816-22_HE.png]

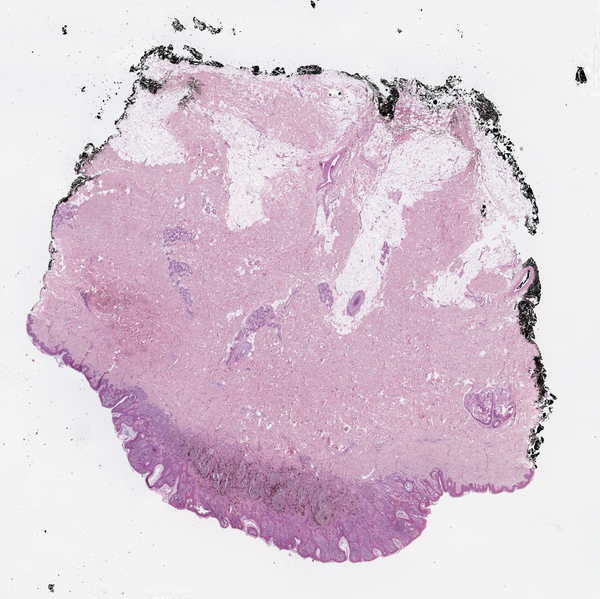

Supplement: S4 Dataset — (ZIP) [file pone.0297146.s010.zip › naples/HE/11188-22_HE.png]

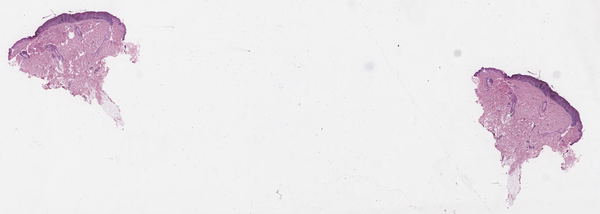

Supplement: S4 Dataset — (ZIP) [file pone.0297146.s010.zip › naples/HE/11711-22_HE.png]

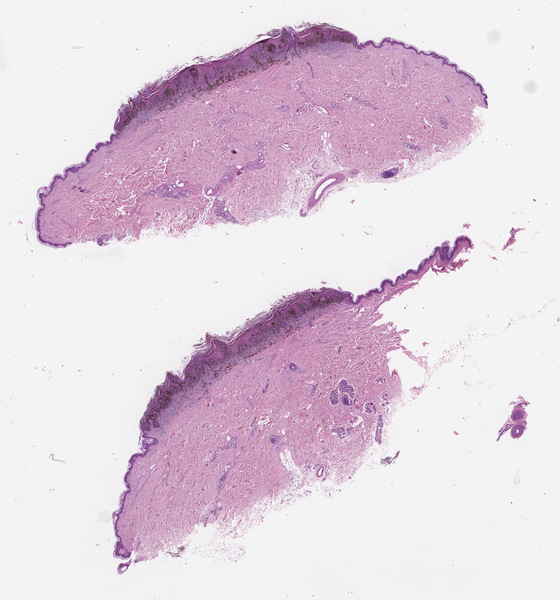

Supplement: S4 Dataset — (ZIP) [file pone.0297146.s010.zip › naples/HE/3231-23_HE.png]
