# Supplementary figures and images for: Evaluating deep learning-based melanoma classification using immunohistochemistry and routine histology: A three center study (part 5 of 7)
Source: PLoS One. 2024 Jan 19;19(1):e0297146. doi: 10.1371/journal.pone.0297146 (PMC10798511; doi:10.1371/journal.pone.0297146)

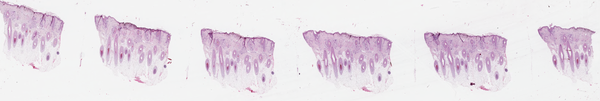

Supplement: S3 Dataset — (ZIP) [file pone.0297146.s009.zip › erlangen/HE/236447_HE.png]

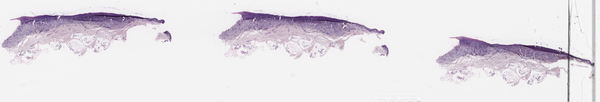

Supplement: S3 Dataset — (ZIP) [file pone.0297146.s009.zip › erlangen/HE/107862_HE.png]

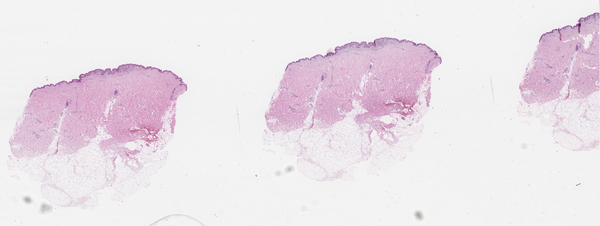

Supplement: S3 Dataset — (ZIP) [file pone.0297146.s009.zip › erlangen/HE/513942_HE.png]

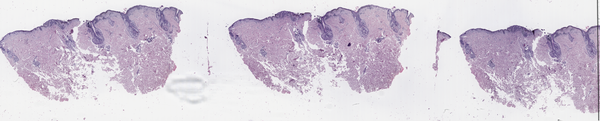

Supplement: S3 Dataset — (ZIP) [file pone.0297146.s009.zip › erlangen/HE/188206-B_HE.png]

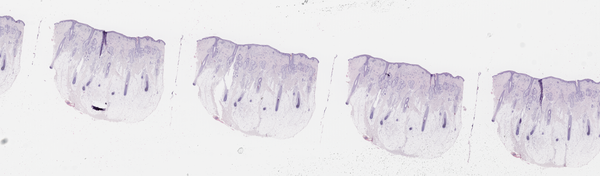

Supplement: S3 Dataset — (ZIP) [file pone.0297146.s009.zip › erlangen/HE/475184_HE.png]

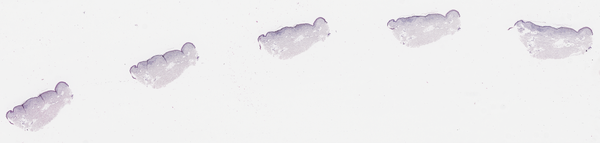

Supplement: S3 Dataset — (ZIP) [file pone.0297146.s009.zip › erlangen/HE/460281-1_HE.png]

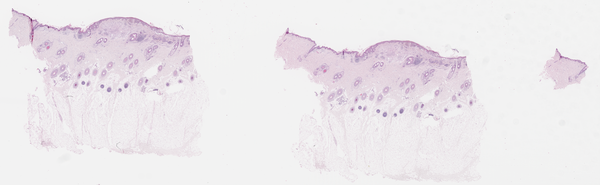

Supplement: S3 Dataset — (ZIP) [file pone.0297146.s009.zip › erlangen/HE/504265_HE.png]

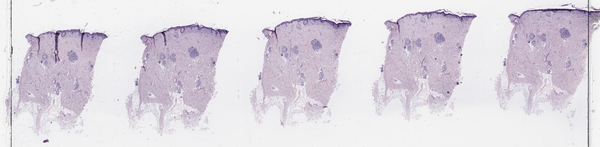

Supplement: S3 Dataset — (ZIP) [file pone.0297146.s009.zip › erlangen/HE/127193_HE.png]

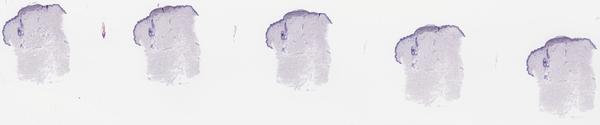

Supplement: S3 Dataset — (ZIP) [file pone.0297146.s009.zip › erlangen/HE/187825-1_HE.png]

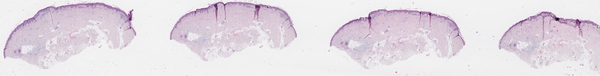

Supplement: S3 Dataset — (ZIP) [file pone.0297146.s009.zip › erlangen/HE/367891-A_HE.png]

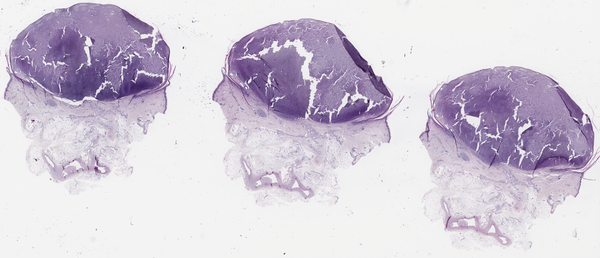

Supplement: S3 Dataset — (ZIP) [file pone.0297146.s009.zip › erlangen/HE/184217_HE.png]

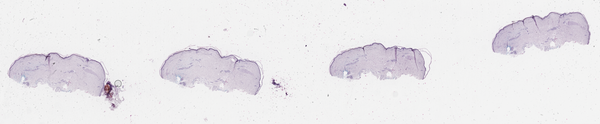

Supplement: S3 Dataset — (ZIP) [file pone.0297146.s009.zip › erlangen/HE/445907_HE.png]

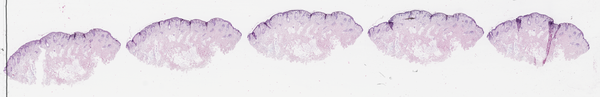

Supplement: S3 Dataset — (ZIP) [file pone.0297146.s009.zip › erlangen/HE/432326_HE.png]

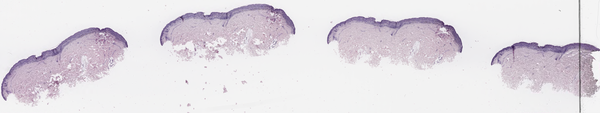

Supplement: S3 Dataset — (ZIP) [file pone.0297146.s009.zip › erlangen/HE/109342_HE.png]

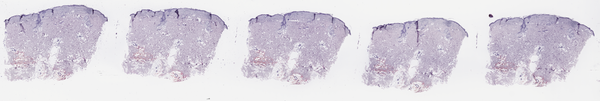

Supplement: S3 Dataset — (ZIP) [file pone.0297146.s009.zip › erlangen/HE/213076_HE.png]

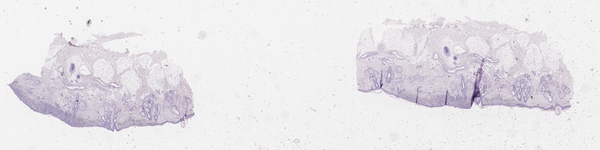

Supplement: S3 Dataset — (ZIP) [file pone.0297146.s009.zip › erlangen/HE/479037-B_HE.png]

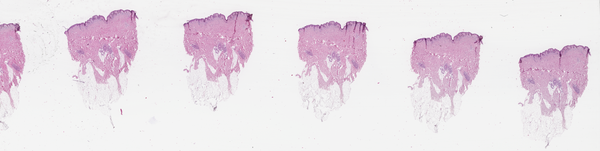

Supplement: S3 Dataset — (ZIP) [file pone.0297146.s009.zip › erlangen/HE/317472_HE.png]

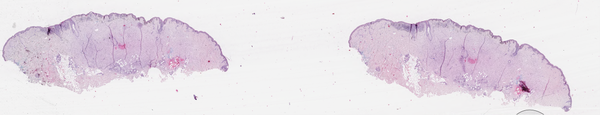

Supplement: S3 Dataset — (ZIP) [file pone.0297146.s009.zip › erlangen/HE/388600-B_HE.png]

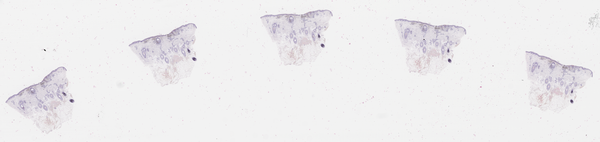

Supplement: S3 Dataset — (ZIP) [file pone.0297146.s009.zip › erlangen/HE/490828_HE.png]

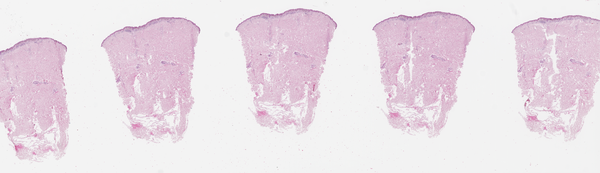

Supplement: S3 Dataset — (ZIP) [file pone.0297146.s009.zip › erlangen/HE/290858-1_HE.png]

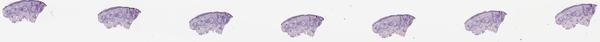

Supplement: S3 Dataset — (ZIP) [file pone.0297146.s009.zip › erlangen/HE/157765_HE.png]

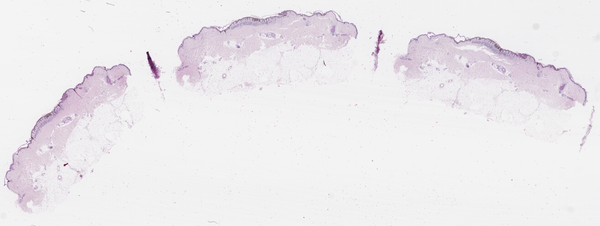

Supplement: S3 Dataset — (ZIP) [file pone.0297146.s009.zip › erlangen/HE/419114_HE.png]

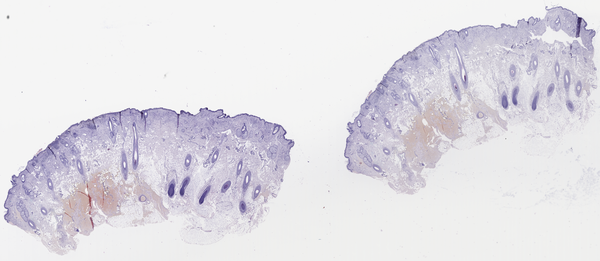

Supplement: S3 Dataset — (ZIP) [file pone.0297146.s009.zip › erlangen/HE/186342-A_HE.png]

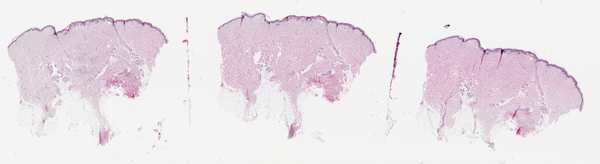

Supplement: S3 Dataset — (ZIP) [file pone.0297146.s009.zip › erlangen/HE/439062-B_HE.png]

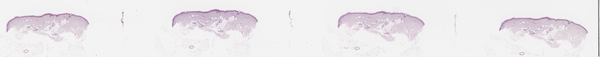

Supplement: S3 Dataset — (ZIP) [file pone.0297146.s009.zip › erlangen/HE/217214_HE.png]

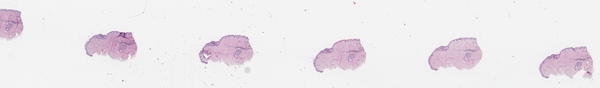

Supplement: S3 Dataset — (ZIP) [file pone.0297146.s009.zip › erlangen/HE/268475_HE.png]

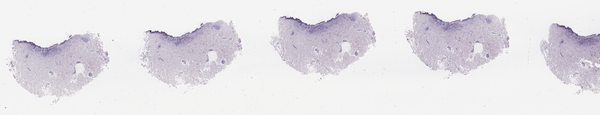

Supplement: S3 Dataset — (ZIP) [file pone.0297146.s009.zip › erlangen/HE/212352-B_HE.png]

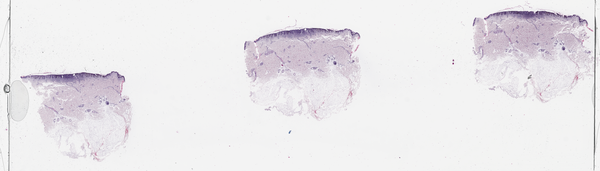

Supplement: S3 Dataset — (ZIP) [file pone.0297146.s009.zip › erlangen/HE/112823_HE.png]

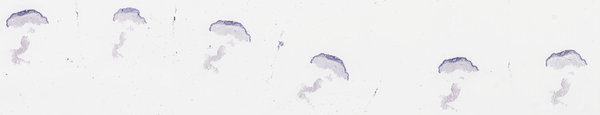

Supplement: S3 Dataset — (ZIP) [file pone.0297146.s009.zip › erlangen/HE/185775-2_HE.png]

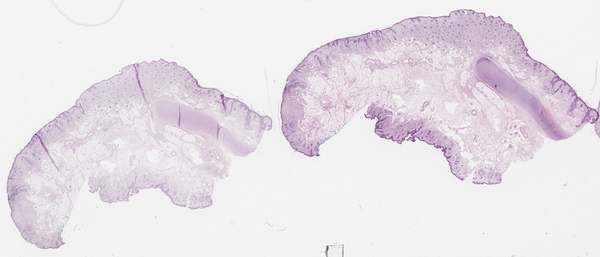

Supplement: S3 Dataset — (ZIP) [file pone.0297146.s009.zip › erlangen/HE/217797_HE.png]

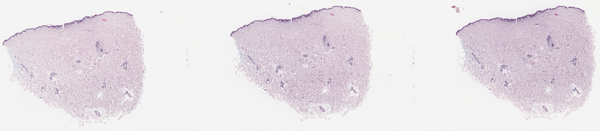

Supplement: S3 Dataset — (ZIP) [file pone.0297146.s009.zip › erlangen/HE/144637-A_HE.png]

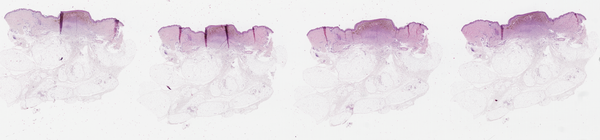

Supplement: S3 Dataset — (ZIP) [file pone.0297146.s009.zip › erlangen/HE/217052_HE.png]

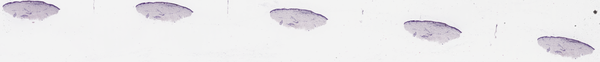

Supplement: S3 Dataset — (ZIP) [file pone.0297146.s009.zip › erlangen/HE/104888_HE.png]

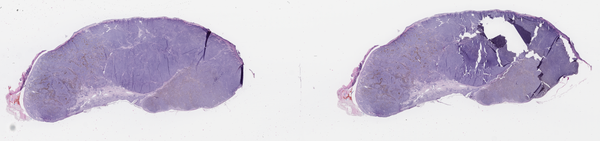

Supplement: S3 Dataset — (ZIP) [file pone.0297146.s009.zip › erlangen/HE/226010-A_HE.png]

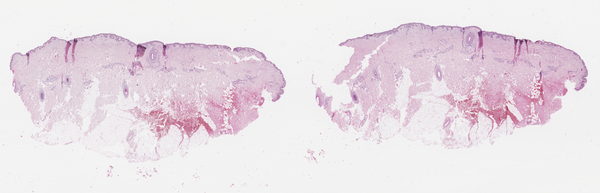

Supplement: S3 Dataset — (ZIP) [file pone.0297146.s009.zip › erlangen/HE/375136-A_HE.png]

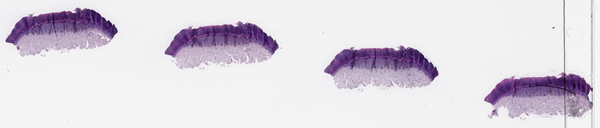

Supplement: S3 Dataset — (ZIP) [file pone.0297146.s009.zip › erlangen/HE/100111_HE.png]

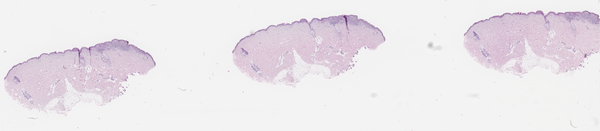

Supplement: S3 Dataset — (ZIP) [file pone.0297146.s009.zip › erlangen/HE/521208_HE.png]

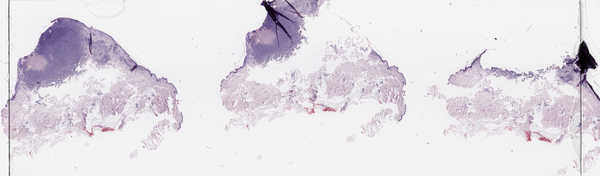

Supplement: S3 Dataset — (ZIP) [file pone.0297146.s009.zip › erlangen/HE/125801_HE.png]

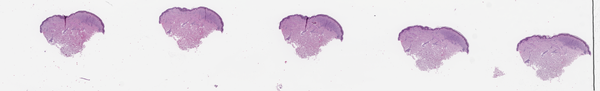

Supplement: S3 Dataset — (ZIP) [file pone.0297146.s009.zip › erlangen/HE/440663_HE.png]

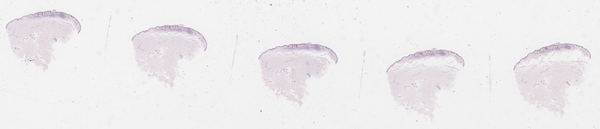

Supplement: S3 Dataset — (ZIP) [file pone.0297146.s009.zip › erlangen/HE/458014_HE.png]

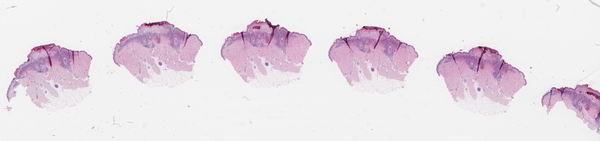

Supplement: S3 Dataset — (ZIP) [file pone.0297146.s009.zip › erlangen/HE/453916_HE.png]

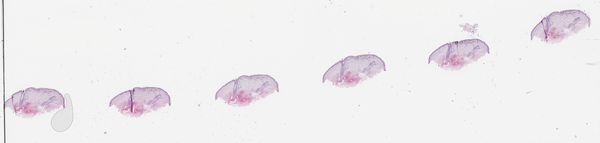

Supplement: S3 Dataset — (ZIP) [file pone.0297146.s009.zip › erlangen/HE/400143-1_HE.png]

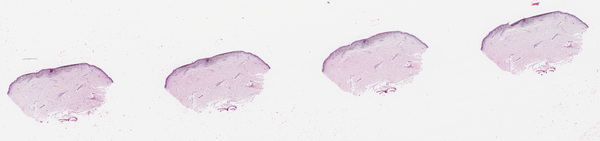

Supplement: S3 Dataset — (ZIP) [file pone.0297146.s009.zip › erlangen/HE/496323-A_HE.png]

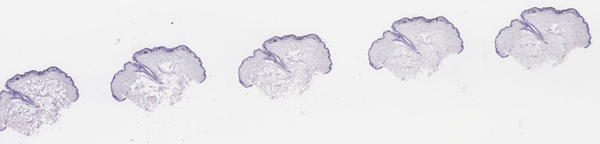

Supplement: S3 Dataset — (ZIP) [file pone.0297146.s009.zip › erlangen/HE/137491_HE.png]

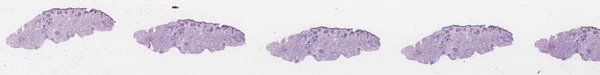

Supplement: S3 Dataset — (ZIP) [file pone.0297146.s009.zip › erlangen/HE/343276_HE.png]

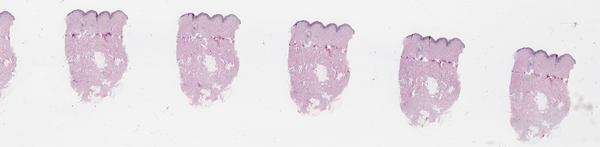

Supplement: S3 Dataset — (ZIP) [file pone.0297146.s009.zip › erlangen/HE/314706-1_HE.png]

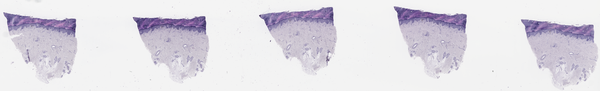

Supplement: S3 Dataset — (ZIP) [file pone.0297146.s009.zip › erlangen/HE/189769_HE.png]

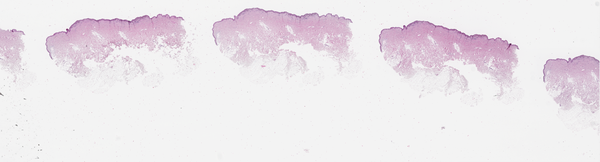

Supplement: S3 Dataset — (ZIP) [file pone.0297146.s009.zip › erlangen/HE/373976_HE.png]

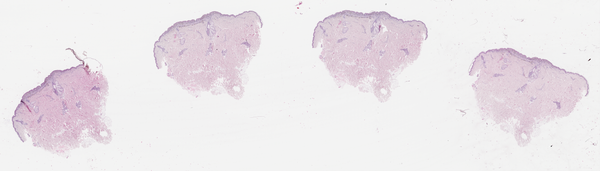

Supplement: S3 Dataset — (ZIP) [file pone.0297146.s009.zip › erlangen/HE/225837_HE.png]

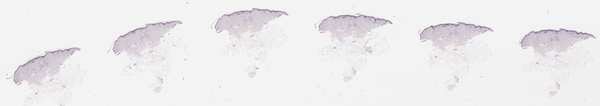

Supplement: S3 Dataset — (ZIP) [file pone.0297146.s009.zip › erlangen/HE/337110_HE.png]

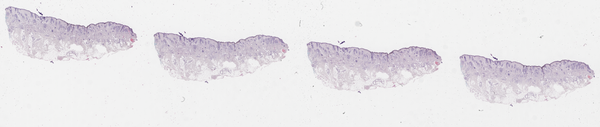

Supplement: S3 Dataset — (ZIP) [file pone.0297146.s009.zip › erlangen/HE/368694_HE.png]

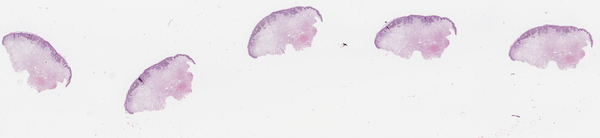

Supplement: S3 Dataset — (ZIP) [file pone.0297146.s009.zip › erlangen/HE/269003_HE.png]

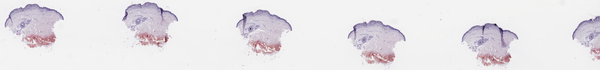

Supplement: S3 Dataset — (ZIP) [file pone.0297146.s009.zip › erlangen/HE/150297_HE.png]

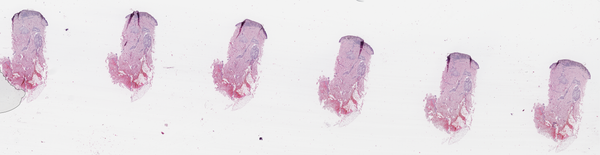

Supplement: S3 Dataset — (ZIP) [file pone.0297146.s009.zip › erlangen/HE/268152_HE.png]

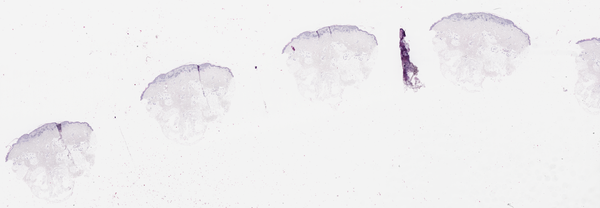

Supplement: S3 Dataset — (ZIP) [file pone.0297146.s009.zip › erlangen/HE/232317_HE.png]

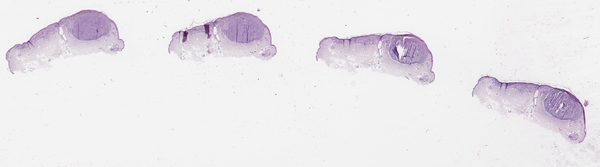

Supplement: S3 Dataset — (ZIP) [file pone.0297146.s009.zip › erlangen/HE/343178_HE.png]

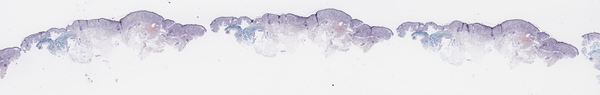

Supplement: S3 Dataset — (ZIP) [file pone.0297146.s009.zip › erlangen/HE/195837-A_HE.png]

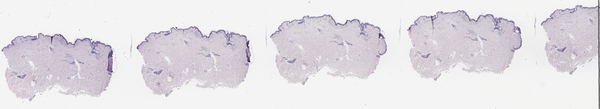

Supplement: S3 Dataset — (ZIP) [file pone.0297146.s009.zip › erlangen/HE/129216-A_HE.png]

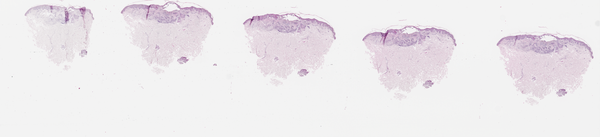

Supplement: S3 Dataset — (ZIP) [file pone.0297146.s009.zip › erlangen/HE/466480_HE.png]

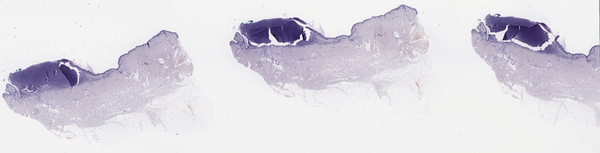

Supplement: S3 Dataset — (ZIP) [file pone.0297146.s009.zip › erlangen/HE/166949_HE.png]

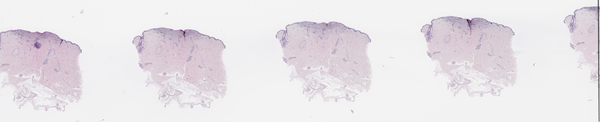

Supplement: S3 Dataset — (ZIP) [file pone.0297146.s009.zip › erlangen/HE/213578_HE.png]

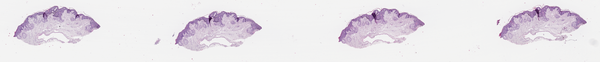

Supplement: S3 Dataset — (ZIP) [file pone.0297146.s009.zip › erlangen/HE/325002_HE.png]

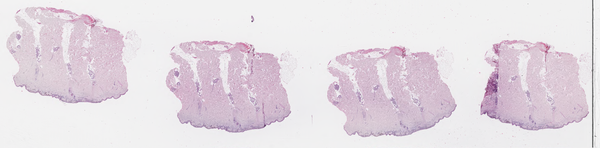

Supplement: S3 Dataset — (ZIP) [file pone.0297146.s009.zip › erlangen/HE/363877_HE.png]

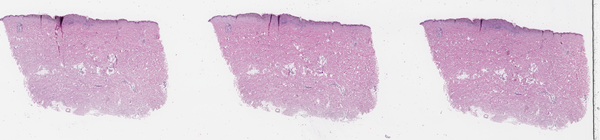

Supplement: S3 Dataset — (ZIP) [file pone.0297146.s009.zip › erlangen/HE/454218_HE.png]

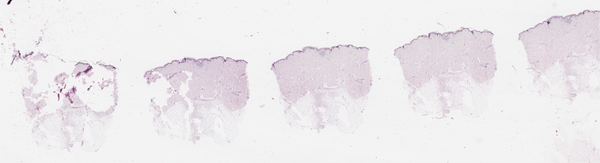

Supplement: S3 Dataset — (ZIP) [file pone.0297146.s009.zip › erlangen/HE/312697_HE.png]

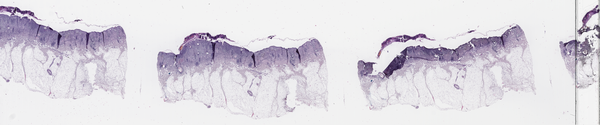

Supplement: S3 Dataset — (ZIP) [file pone.0297146.s009.zip › erlangen/HE/122032_HE.png]

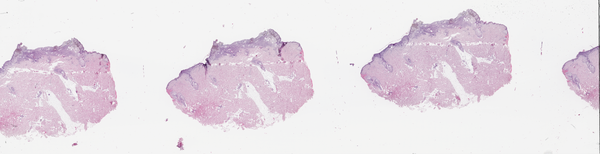

Supplement: S3 Dataset — (ZIP) [file pone.0297146.s009.zip › erlangen/HE/314706-2_HE.png]

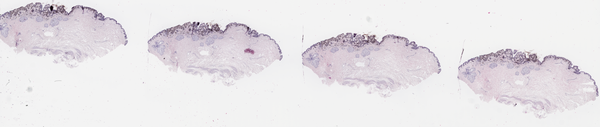

Supplement: S3 Dataset — (ZIP) [file pone.0297146.s009.zip › erlangen/HE/206605_HE.png]

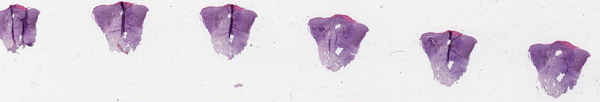

Supplement: S3 Dataset — (ZIP) [file pone.0297146.s009.zip › erlangen/HE/353896_HE.png]

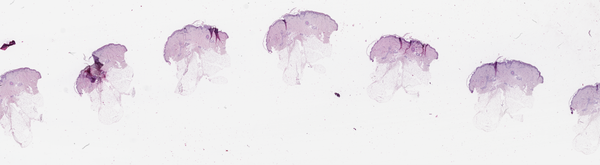

Supplement: S3 Dataset — (ZIP) [file pone.0297146.s009.zip › erlangen/HE/269748_HE.png]

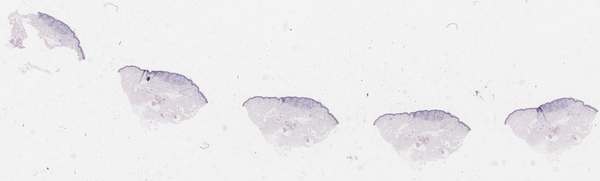

Supplement: S3 Dataset — (ZIP) [file pone.0297146.s009.zip › erlangen/HE/478728-B_HE.png]

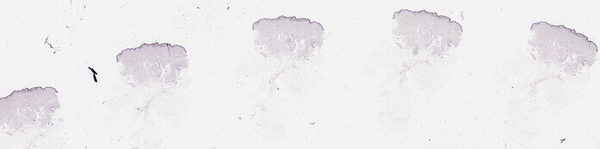

Supplement: S3 Dataset — (ZIP) [file pone.0297146.s009.zip › erlangen/HE/255314_HE.png]

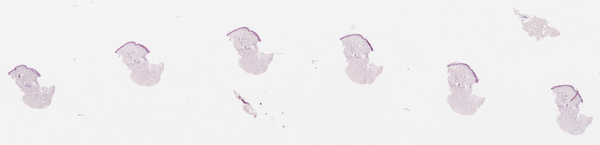

Supplement: S3 Dataset — (ZIP) [file pone.0297146.s009.zip › erlangen/HE/281410_HE.png]

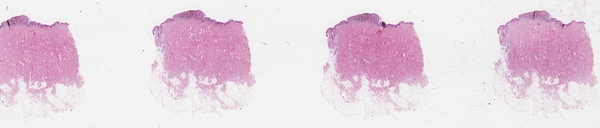

Supplement: S3 Dataset — (ZIP) [file pone.0297146.s009.zip › erlangen/HE/275869_HE.png]

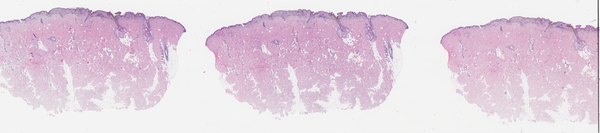

Supplement: S3 Dataset — (ZIP) [file pone.0297146.s009.zip › erlangen/HE/511591_HE.png]

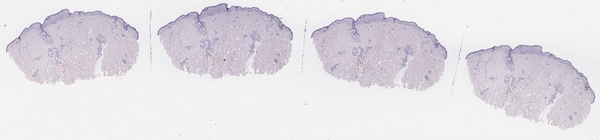

Supplement: S3 Dataset — (ZIP) [file pone.0297146.s009.zip › erlangen/HE/210077-2_HE.png]

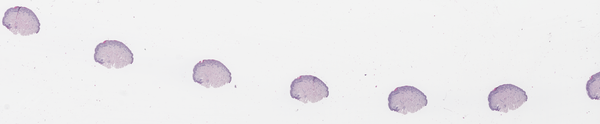

Supplement: S3 Dataset — (ZIP) [file pone.0297146.s009.zip › erlangen/HE/228537-2_HE.png]

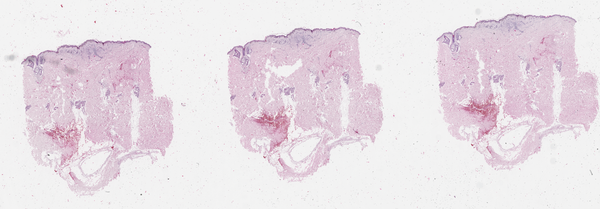

Supplement: S3 Dataset — (ZIP) [file pone.0297146.s009.zip › erlangen/HE/455155_HE.png]

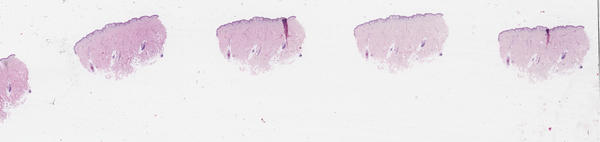

Supplement: S3 Dataset — (ZIP) [file pone.0297146.s009.zip › erlangen/HE/370237-1_HE.png]

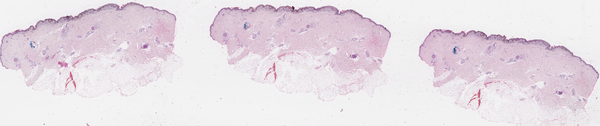

Supplement: S3 Dataset — (ZIP) [file pone.0297146.s009.zip › erlangen/HE/344572_HE.png]

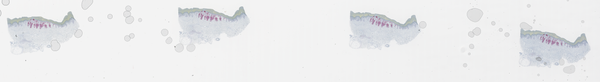

Supplement: S3 Dataset — (ZIP) [file pone.0297146.s009.zip › erlangen/MelanA/421431_MelanA.png]

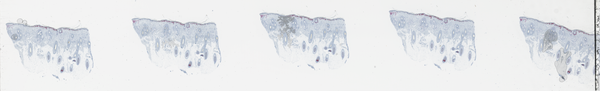

Supplement: S3 Dataset — (ZIP) [file pone.0297146.s009.zip › erlangen/MelanA/236447_MelanA.png]

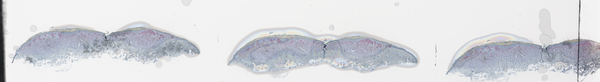

Supplement: S3 Dataset — (ZIP) [file pone.0297146.s009.zip › erlangen/MelanA/107862_MelanA.png]

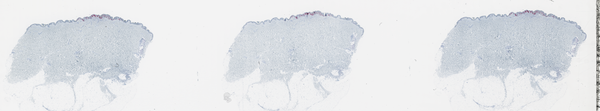

Supplement: S3 Dataset — (ZIP) [file pone.0297146.s009.zip › erlangen/MelanA/513942_MelanA.png]

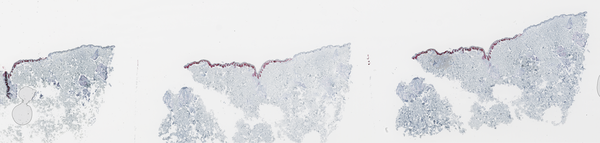

Supplement: S3 Dataset — (ZIP) [file pone.0297146.s009.zip › erlangen/MelanA/188206-B_MelanA.png]

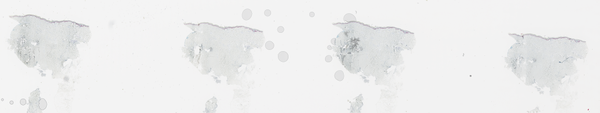

Supplement: S3 Dataset — (ZIP) [file pone.0297146.s009.zip › erlangen/MelanA/475184_MelanA.png]

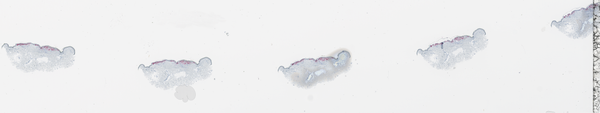

Supplement: S3 Dataset — (ZIP) [file pone.0297146.s009.zip › erlangen/MelanA/460281-1_MelanA.png]

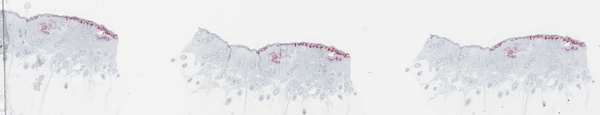

Supplement: S3 Dataset — (ZIP) [file pone.0297146.s009.zip › erlangen/MelanA/504265_MelanA.png]

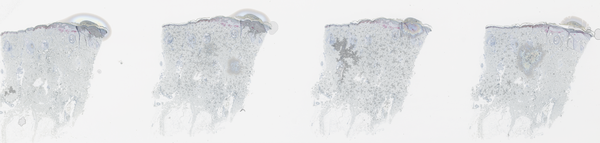

Supplement: S3 Dataset — (ZIP) [file pone.0297146.s009.zip › erlangen/MelanA/127193_MelanA.png]

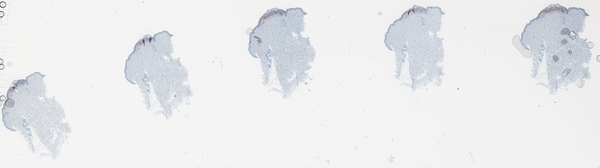

Supplement: S3 Dataset — (ZIP) [file pone.0297146.s009.zip › erlangen/MelanA/187825-1_MelanA.png]

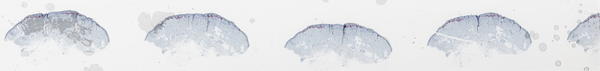

Supplement: S3 Dataset — (ZIP) [file pone.0297146.s009.zip › erlangen/MelanA/367891-A_MelanA.png]

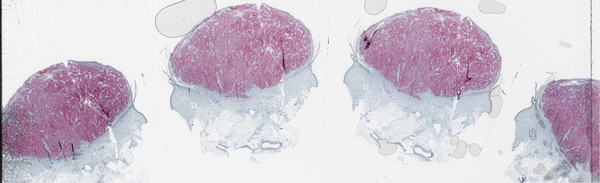

Supplement: S3 Dataset — (ZIP) [file pone.0297146.s009.zip › erlangen/MelanA/184217_MelanA.png]

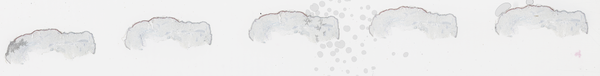

Supplement: S3 Dataset — (ZIP) [file pone.0297146.s009.zip › erlangen/MelanA/445907_MelanA.png]

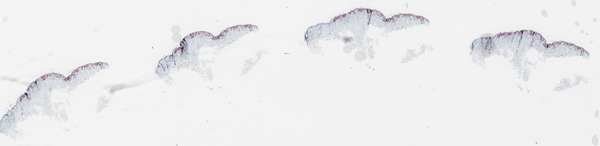

Supplement: S3 Dataset — (ZIP) [file pone.0297146.s009.zip › erlangen/MelanA/432326_MelanA.png]

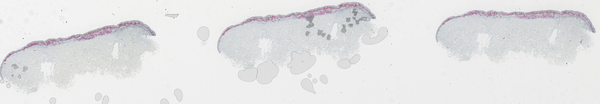

Supplement: S3 Dataset — (ZIP) [file pone.0297146.s009.zip › erlangen/MelanA/109342_MelanA.png]

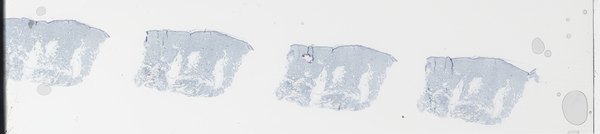

Supplement: S3 Dataset — (ZIP) [file pone.0297146.s009.zip › erlangen/MelanA/213076_MelanA.png]

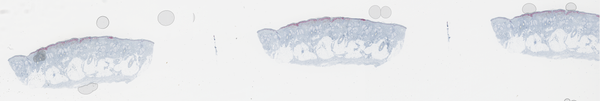

Supplement: S3 Dataset — (ZIP) [file pone.0297146.s009.zip › erlangen/MelanA/479037-B_MelanA.png]

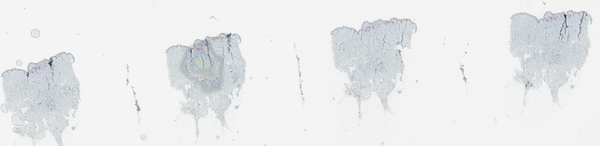

Supplement: S3 Dataset — (ZIP) [file pone.0297146.s009.zip › erlangen/MelanA/317472_MelanA.png]

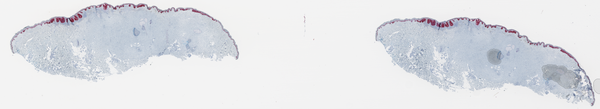

Supplement: S3 Dataset — (ZIP) [file pone.0297146.s009.zip › erlangen/MelanA/388600-B_MelanA.png]

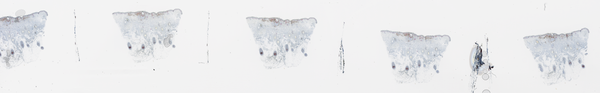

Supplement: S3 Dataset — (ZIP) [file pone.0297146.s009.zip › erlangen/MelanA/490828_MelanA.png]
