# Supplementary figures and images for: Evaluating deep learning-based melanoma classification using immunohistochemistry and routine histology: A three center study (part 7 of 7)
Source: PLoS One. 2024 Jan 19;19(1):e0297146. doi: 10.1371/journal.pone.0297146 (PMC10798511; doi:10.1371/journal.pone.0297146)

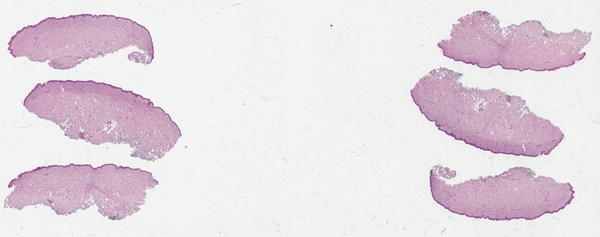

Supplement: S4 Dataset — (ZIP) [file pone.0297146.s010.zip › naples/HE/13885-22_HE.png]

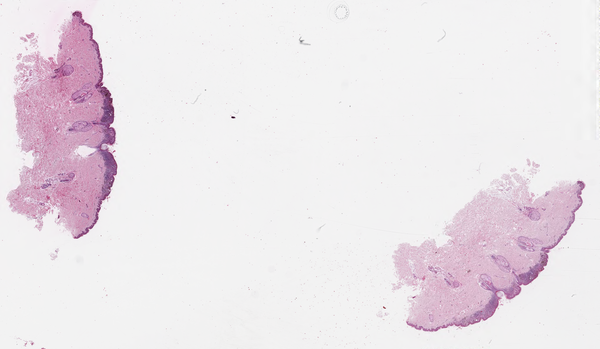

Supplement: S4 Dataset — (ZIP) [file pone.0297146.s010.zip › naples/HE/2145-23_HE.png]

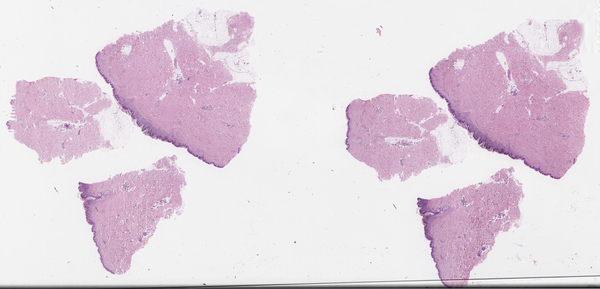

Supplement: S4 Dataset — (ZIP) [file pone.0297146.s010.zip › naples/HE/52-23_HE.png]

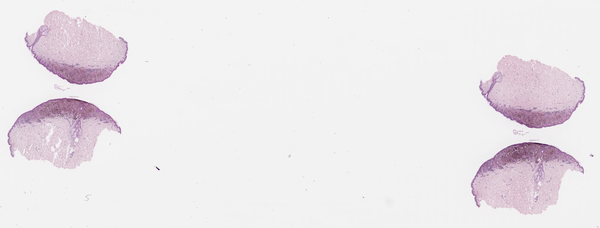

Supplement: S4 Dataset — (ZIP) [file pone.0297146.s010.zip › naples/HE/8728-22_HE.png]

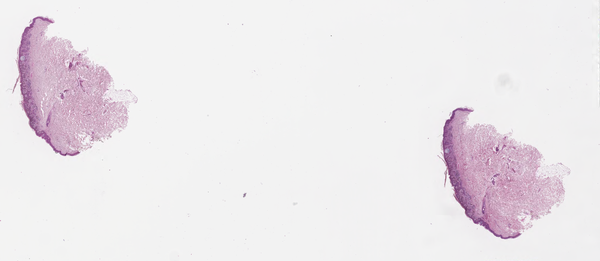

Supplement: S4 Dataset — (ZIP) [file pone.0297146.s010.zip › naples/HE/13754-22_HE.png]

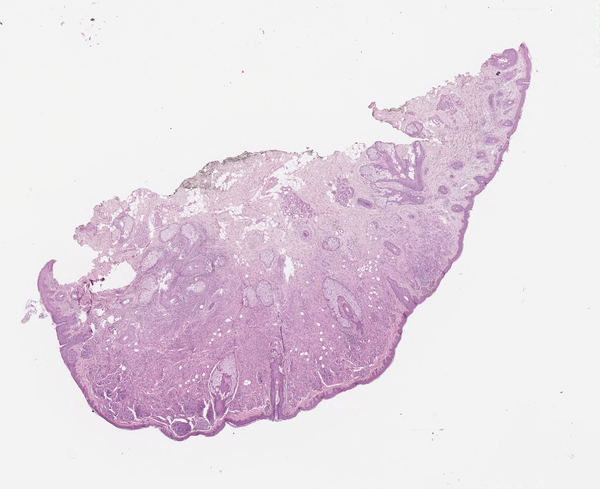

Supplement: S4 Dataset — (ZIP) [file pone.0297146.s010.zip › naples/HE/12247-22_HE.png]

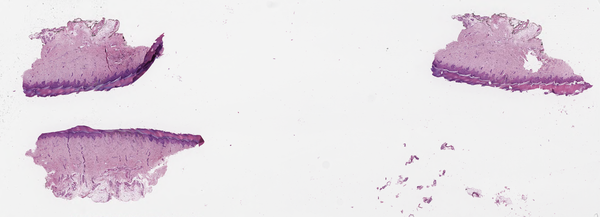

Supplement: S4 Dataset — (ZIP) [file pone.0297146.s010.zip › naples/HE/12528-22_HE.png]

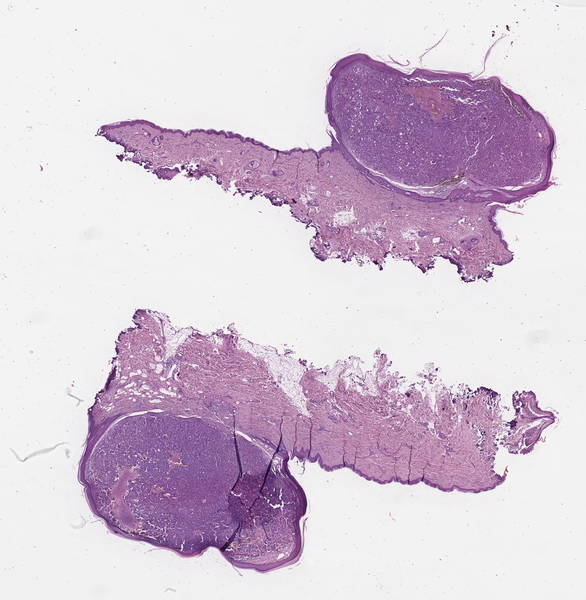

Supplement: S4 Dataset — (ZIP) [file pone.0297146.s010.zip › naples/HE/2730-23_HE.png]

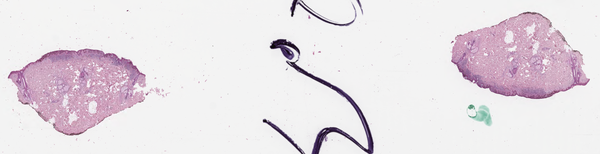

Supplement: S4 Dataset — (ZIP) [file pone.0297146.s010.zip › naples/HE/12136-22_HE.png]

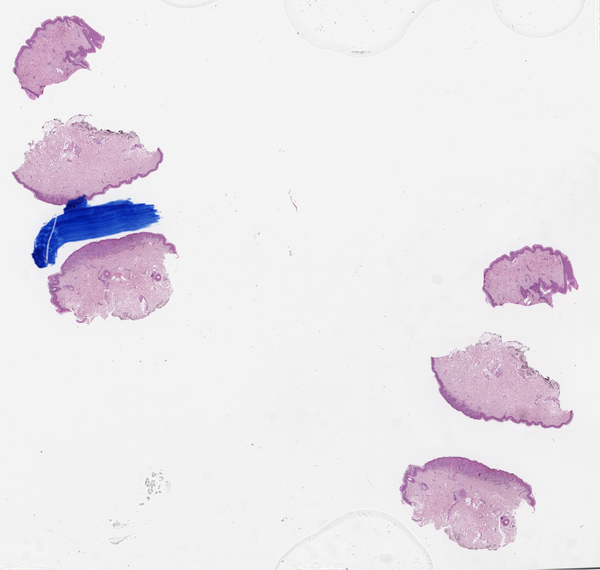

Supplement: S4 Dataset — (ZIP) [file pone.0297146.s010.zip › naples/HE/2325-23_HE.png]

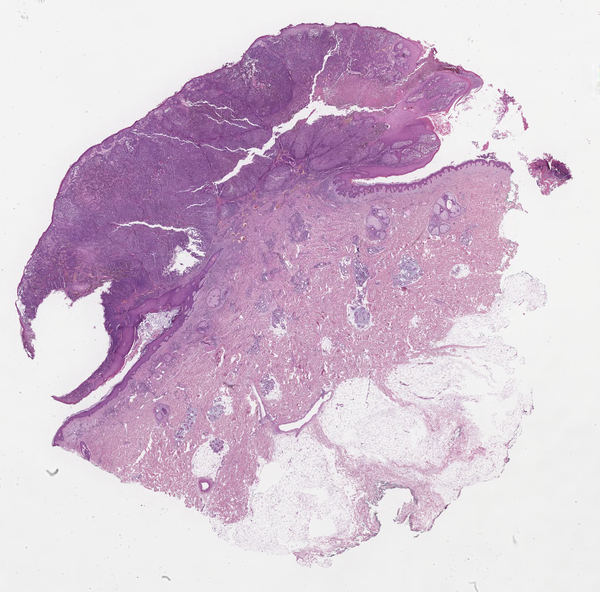

Supplement: S4 Dataset — (ZIP) [file pone.0297146.s010.zip › naples/HE/13135-22_HE.png]

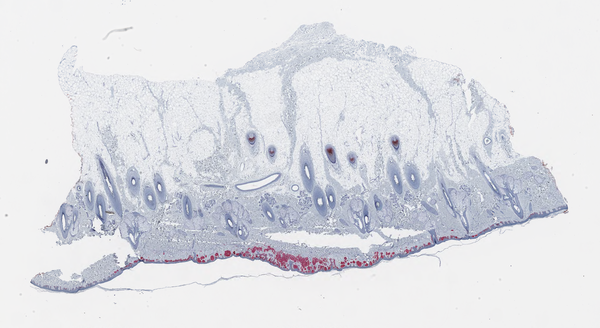

Supplement: S4 Dataset — (ZIP) [file pone.0297146.s010.zip › naples/MelanA/14743-22_MelanA.png]

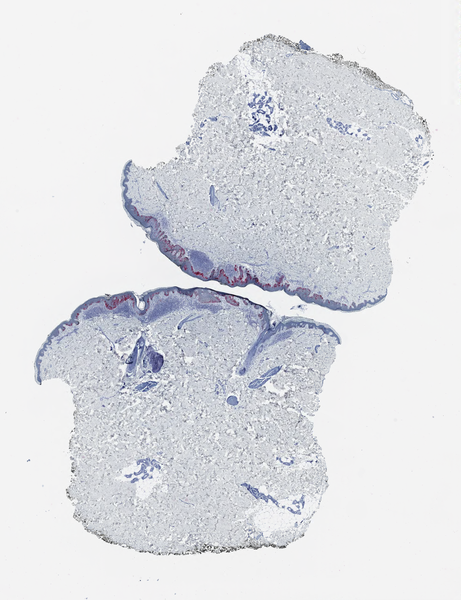

Supplement: S4 Dataset — (ZIP) [file pone.0297146.s010.zip › naples/MelanA/3245-23_MelanA.png]

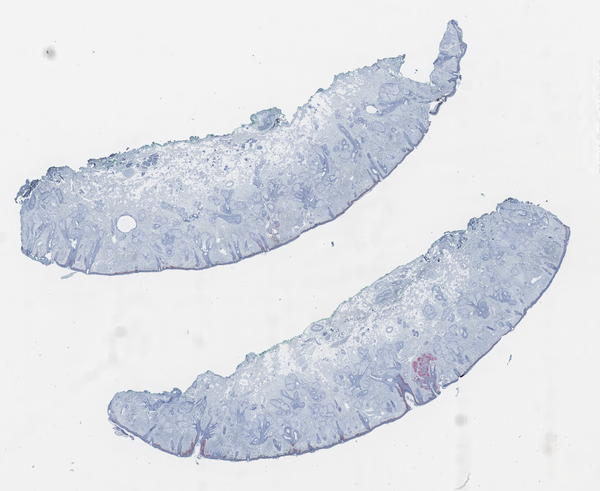

Supplement: S4 Dataset — (ZIP) [file pone.0297146.s010.zip › naples/MelanA/685-23_MelanA.png]

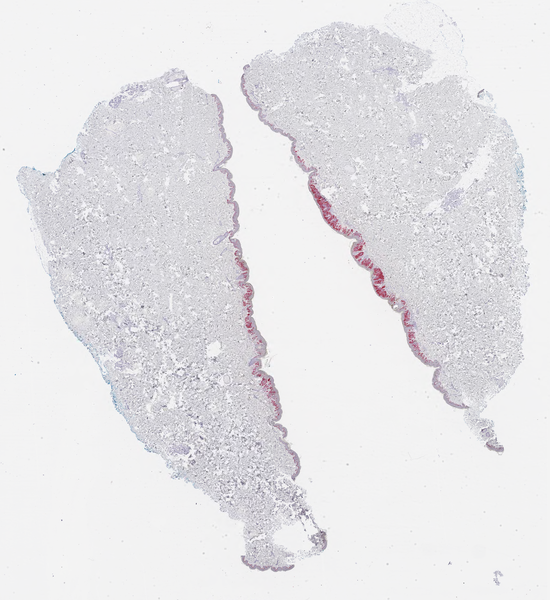

Supplement: S4 Dataset — (ZIP) [file pone.0297146.s010.zip › naples/MelanA/10811-22_MelanA.png]

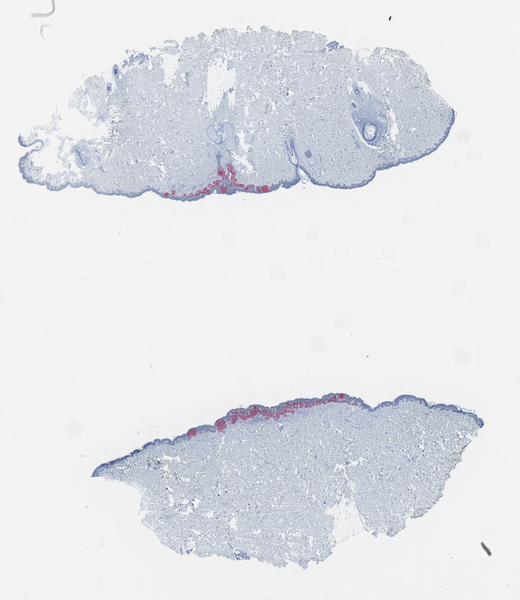

Supplement: S4 Dataset — (ZIP) [file pone.0297146.s010.zip › naples/MelanA/12939-22_MelanA.png]

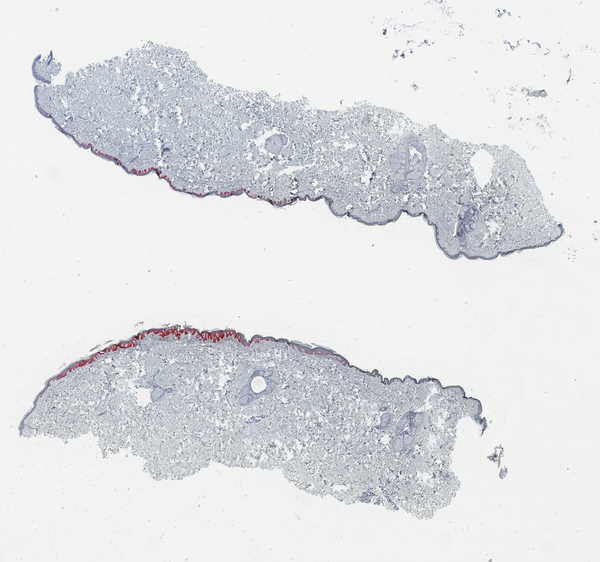

Supplement: S4 Dataset — (ZIP) [file pone.0297146.s010.zip › naples/MelanA/1832-23_MelanA.png]

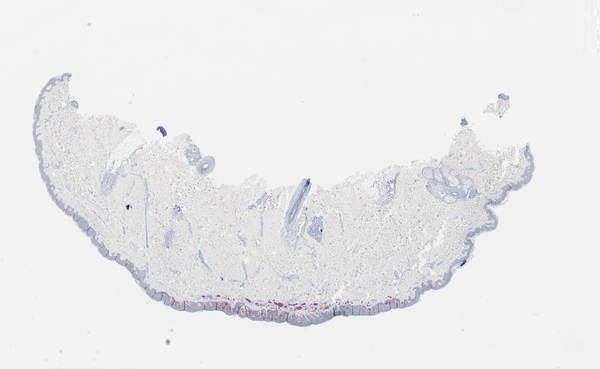

Supplement: S4 Dataset — (ZIP) [file pone.0297146.s010.zip › naples/MelanA/568-23_MelanA.png]

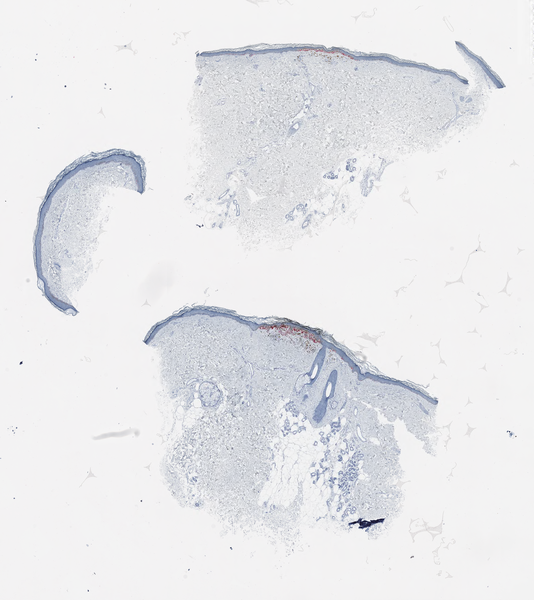

Supplement: S4 Dataset — (ZIP) [file pone.0297146.s010.zip › naples/MelanA/207-23_MelanA.png]

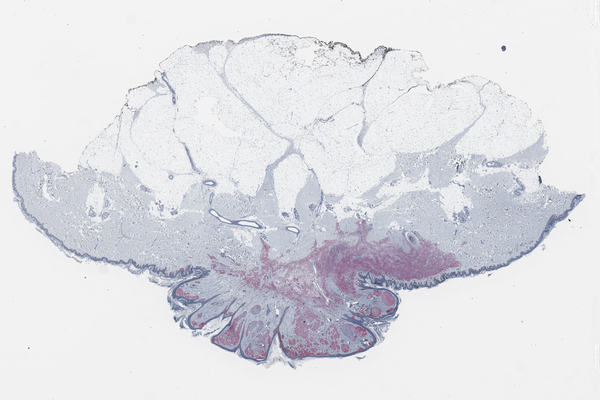

Supplement: S4 Dataset — (ZIP) [file pone.0297146.s010.zip › naples/MelanA/12242-22_MelanA.png]

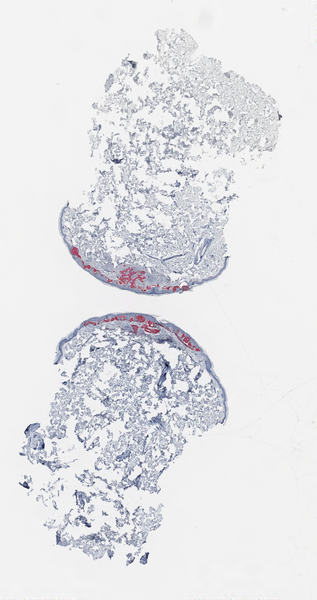

Supplement: S4 Dataset — (ZIP) [file pone.0297146.s010.zip › naples/MelanA/5438-22_MelanA.png]

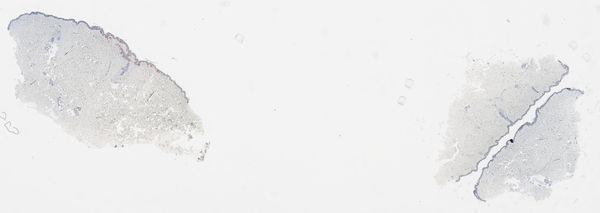

Supplement: S4 Dataset — (ZIP) [file pone.0297146.s010.zip › naples/MelanA/Y23-A_MelanA.png]

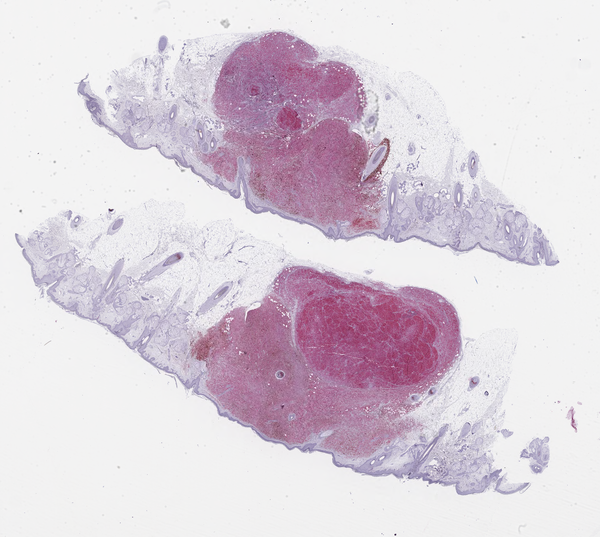

Supplement: S4 Dataset — (ZIP) [file pone.0297146.s010.zip › naples/MelanA/PM97-23_MelanA.png]

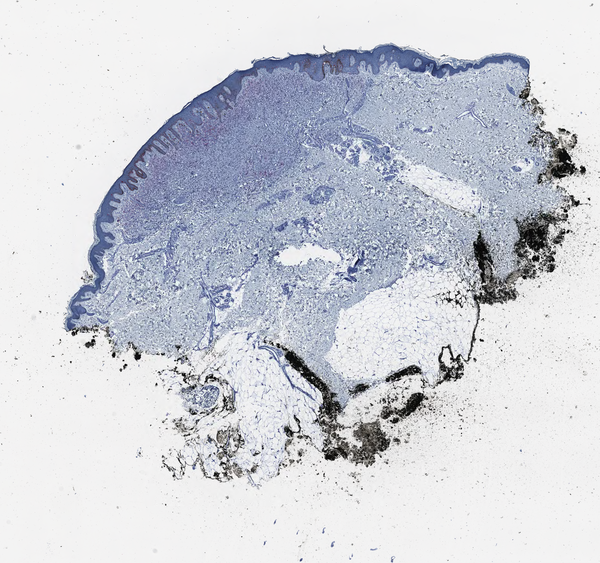

Supplement: S4 Dataset — (ZIP) [file pone.0297146.s010.zip › naples/MelanA/74-23_MelanA.png]

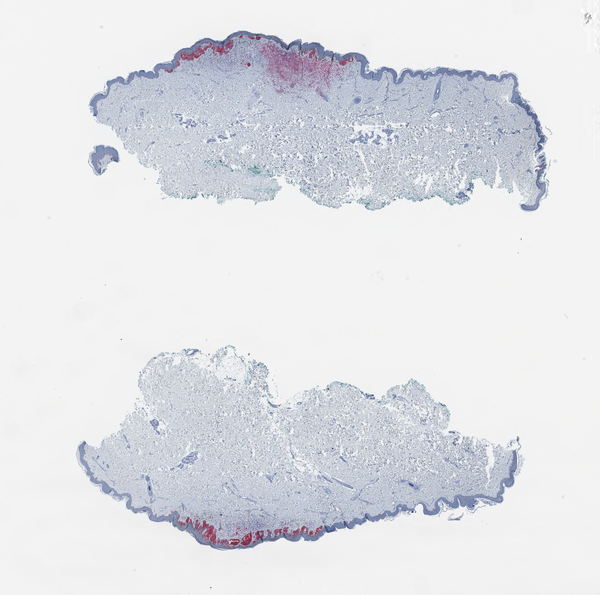

Supplement: S4 Dataset — (ZIP) [file pone.0297146.s010.zip › naples/MelanA/11586-22_MelanA.png]

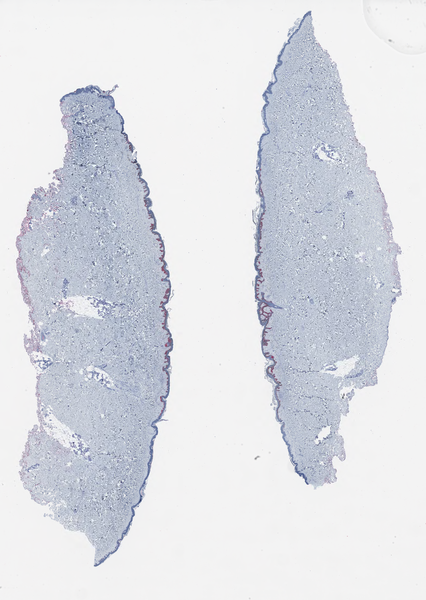

Supplement: S4 Dataset — (ZIP) [file pone.0297146.s010.zip › naples/MelanA/12315-22_MelanA.png]

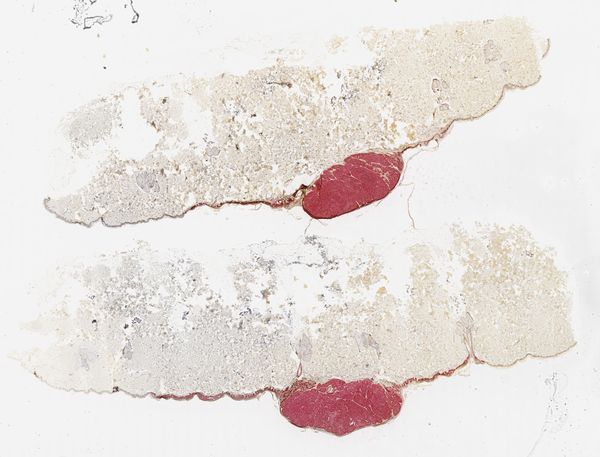

Supplement: S4 Dataset — (ZIP) [file pone.0297146.s010.zip › naples/MelanA/2313-23_MelanA.png]

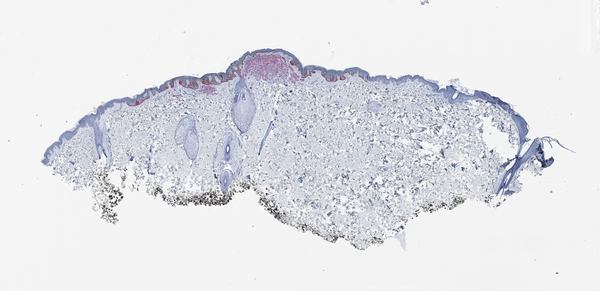

Supplement: S4 Dataset — (ZIP) [file pone.0297146.s010.zip › naples/MelanA/12257-22_MelanA.png]

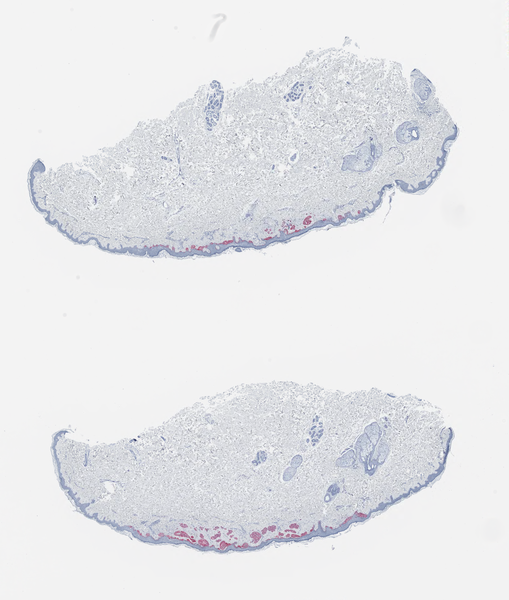

Supplement: S4 Dataset — (ZIP) [file pone.0297146.s010.zip › naples/MelanA/14744-22-2_MelanA.png]

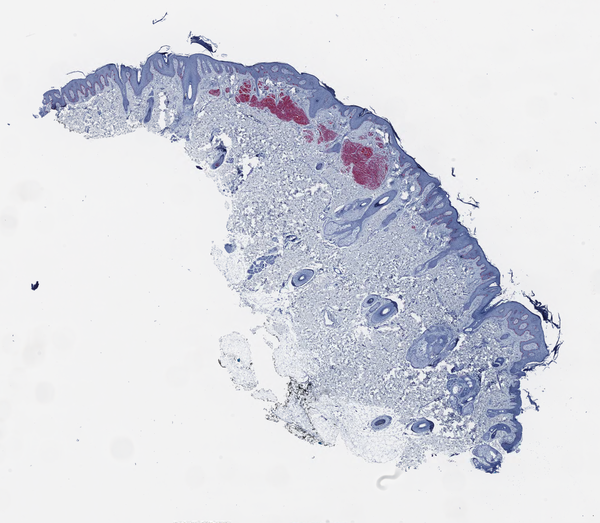

Supplement: S4 Dataset — (ZIP) [file pone.0297146.s010.zip › naples/MelanA/13969-22_MelanA.png]

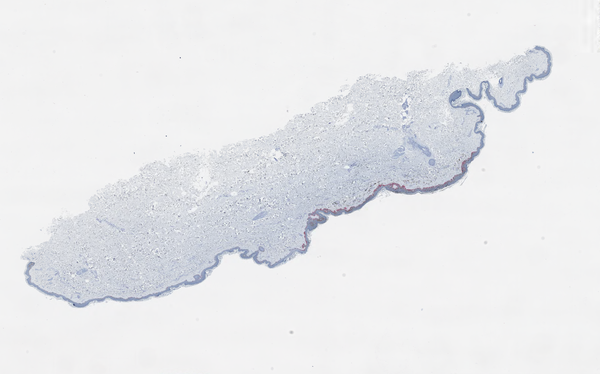

Supplement: S4 Dataset — (ZIP) [file pone.0297146.s010.zip › naples/MelanA/1210-23_MelanA.png]

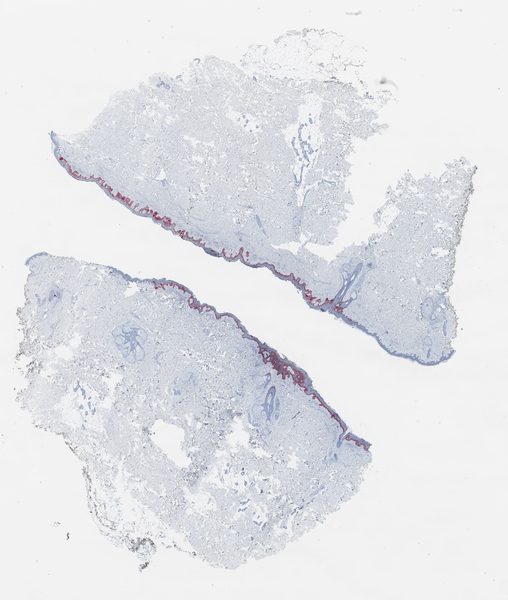

Supplement: S4 Dataset — (ZIP) [file pone.0297146.s010.zip › naples/MelanA/269-23_MelanA.png]

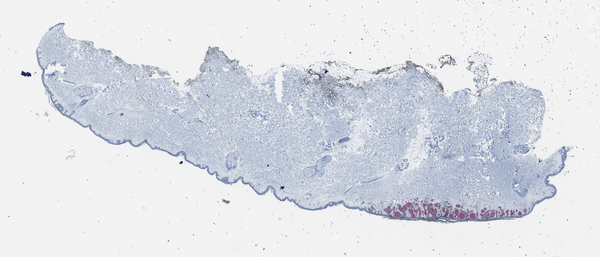

Supplement: S4 Dataset — (ZIP) [file pone.0297146.s010.zip › naples/MelanA/14202-21_MelanA.png]

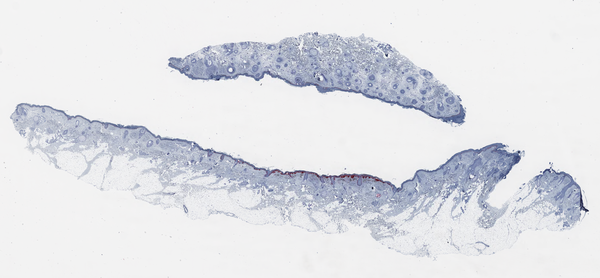

Supplement: S4 Dataset — (ZIP) [file pone.0297146.s010.zip › naples/MelanA/1127-23_MelanA.png]

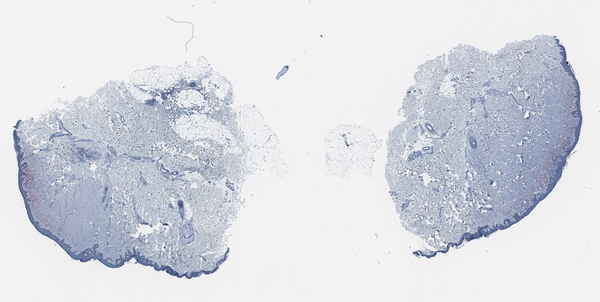

Supplement: S4 Dataset — (ZIP) [file pone.0297146.s010.zip › naples/MelanA/3232-23_MelanA.png]

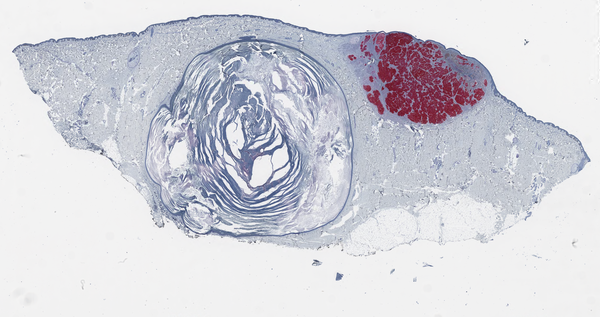

Supplement: S4 Dataset — (ZIP) [file pone.0297146.s010.zip › naples/MelanA/2363-23_MelanA.png]

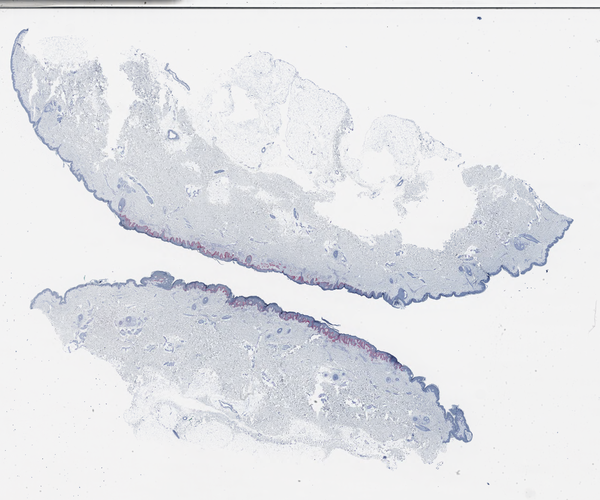

Supplement: S4 Dataset — (ZIP) [file pone.0297146.s010.zip › naples/MelanA/1231-23_MelanA.png]

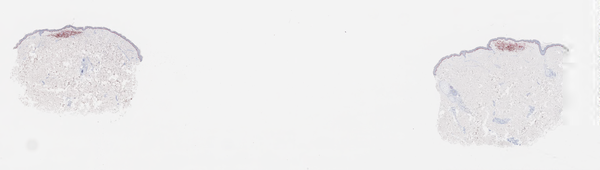

Supplement: S4 Dataset — (ZIP) [file pone.0297146.s010.zip › naples/MelanA/8808-22_MelanA.png]

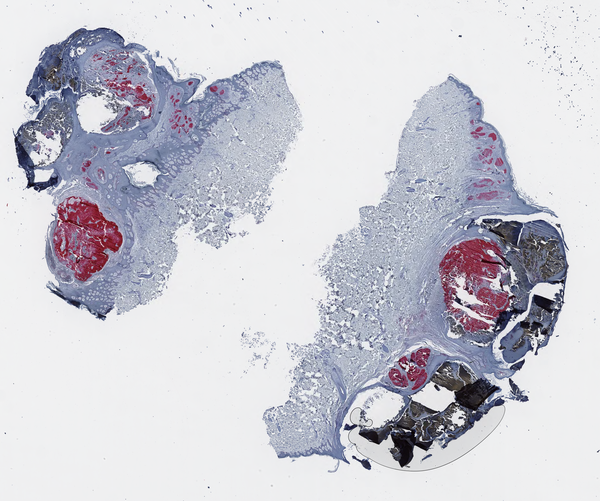

Supplement: S4 Dataset — (ZIP) [file pone.0297146.s010.zip › naples/MelanA/14151-22_MelanA.png]

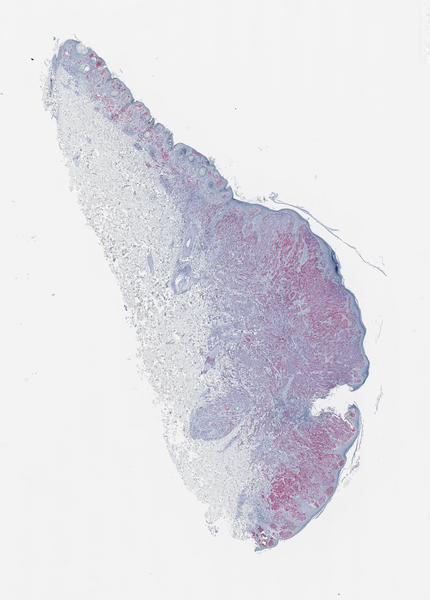

Supplement: S4 Dataset — (ZIP) [file pone.0297146.s010.zip › naples/MelanA/2412-23_MelanA.png]

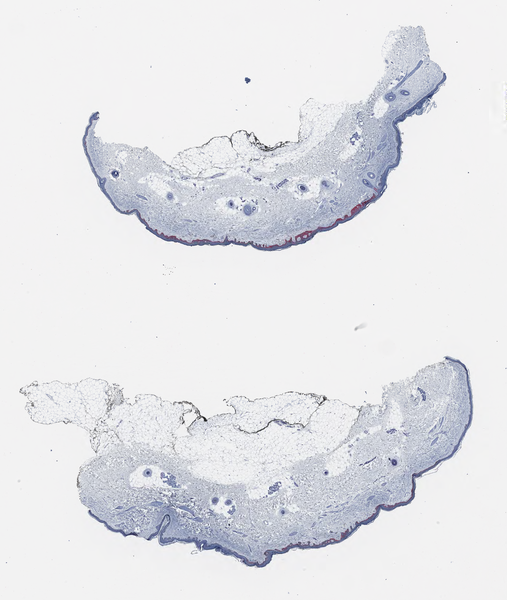

Supplement: S4 Dataset — (ZIP) [file pone.0297146.s010.zip › naples/MelanA/12250-22_MelanA.png]

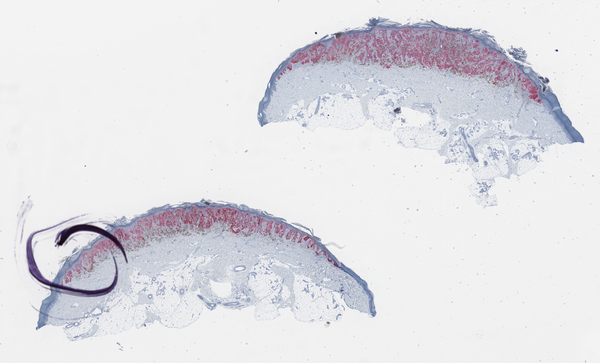

Supplement: S4 Dataset — (ZIP) [file pone.0297146.s010.zip › naples/MelanA/14645-22_MelanA.png]

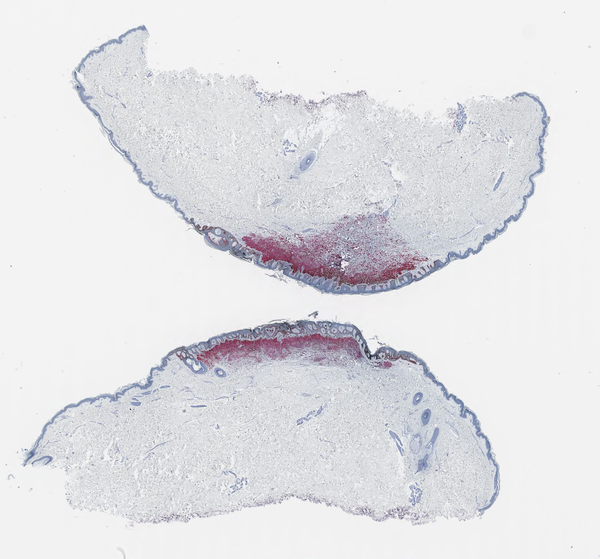

Supplement: S4 Dataset — (ZIP) [file pone.0297146.s010.zip › naples/MelanA/1363-23_MelanA.png]

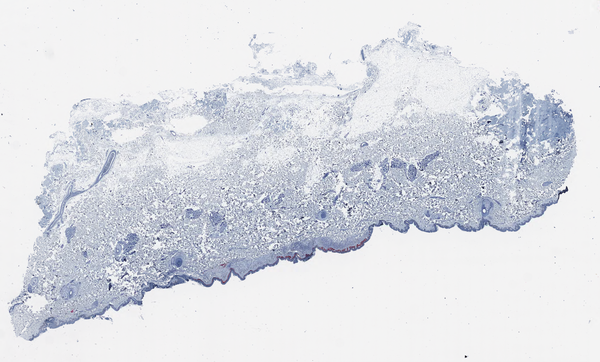

Supplement: S4 Dataset — (ZIP) [file pone.0297146.s010.zip › naples/MelanA/2550-23_MelanA.png]

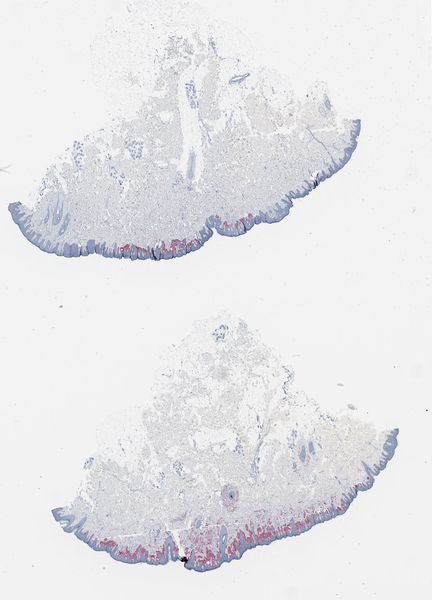

Supplement: S4 Dataset — (ZIP) [file pone.0297146.s010.zip › naples/MelanA/4023-22_MelanA.png]

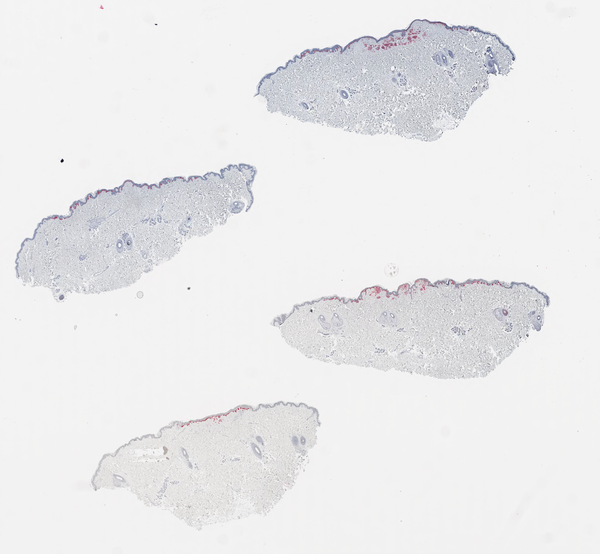

Supplement: S4 Dataset — (ZIP) [file pone.0297146.s010.zip › naples/MelanA/14744-22-1_MelanA.png]

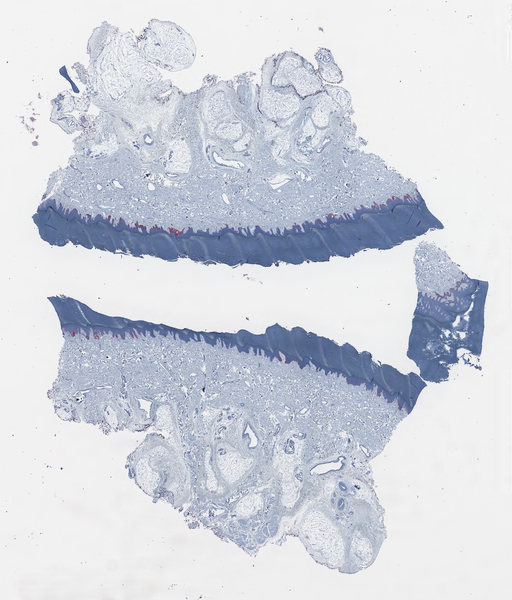

Supplement: S4 Dataset — (ZIP) [file pone.0297146.s010.zip › naples/MelanA/14816-22_MelanA.png]

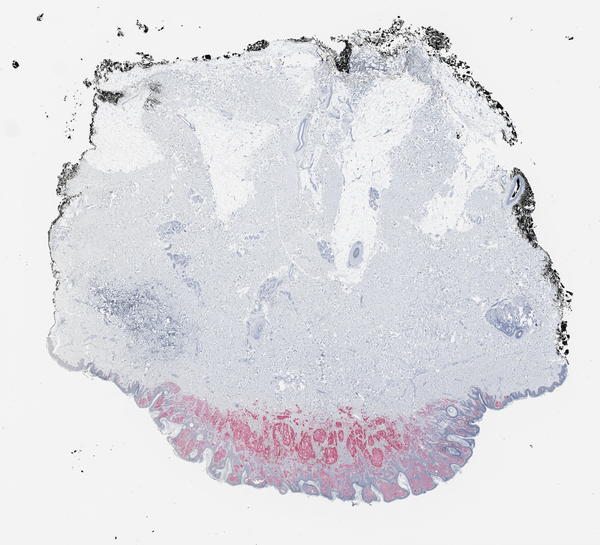

Supplement: S4 Dataset — (ZIP) [file pone.0297146.s010.zip › naples/MelanA/11188-22_MelanA.png]

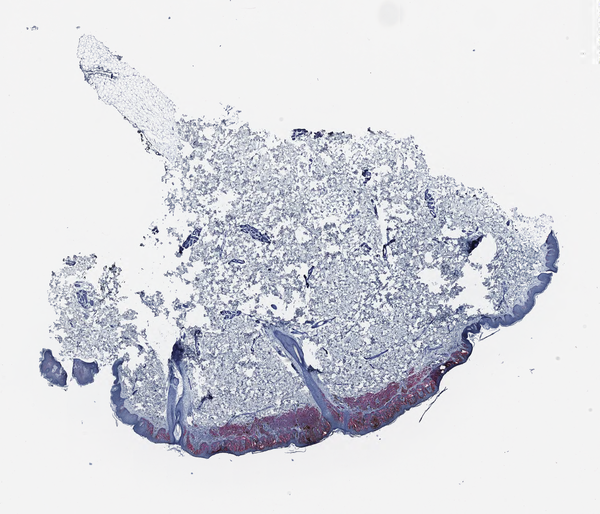

Supplement: S4 Dataset — (ZIP) [file pone.0297146.s010.zip › naples/MelanA/11711-22_MelanA.png]

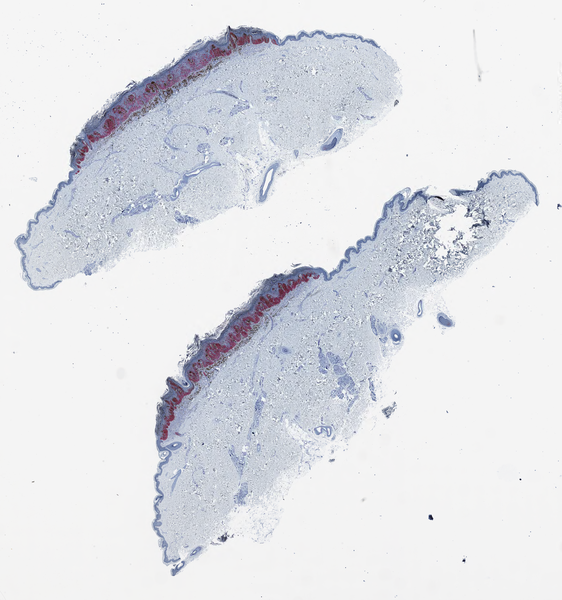

Supplement: S4 Dataset — (ZIP) [file pone.0297146.s010.zip › naples/MelanA/3231-23_MelanA.png]

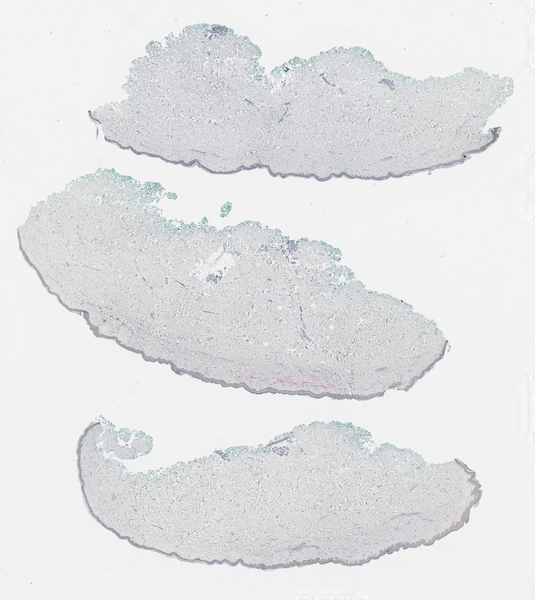

Supplement: S4 Dataset — (ZIP) [file pone.0297146.s010.zip › naples/MelanA/13885-22_MelanA.png]

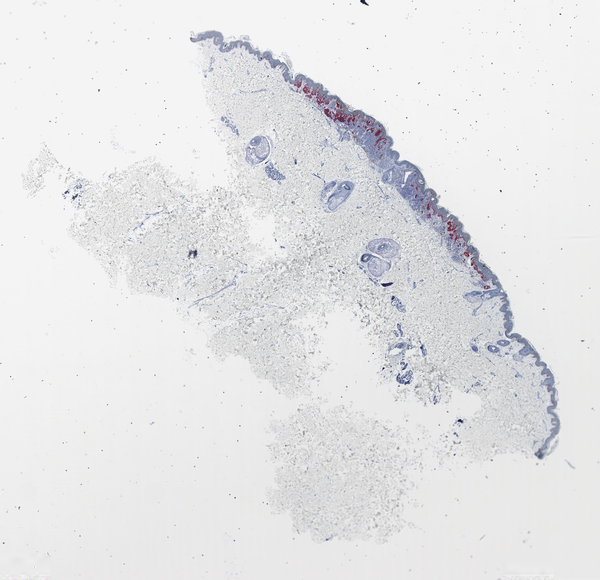

Supplement: S4 Dataset — (ZIP) [file pone.0297146.s010.zip › naples/MelanA/2145-23_MelanA.png]

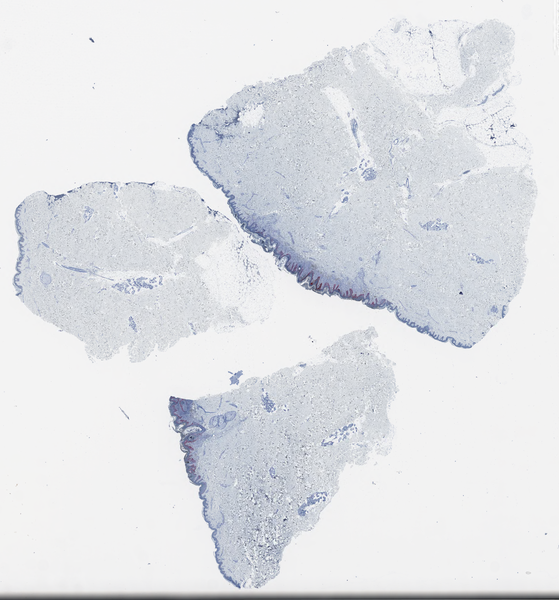

Supplement: S4 Dataset — (ZIP) [file pone.0297146.s010.zip › naples/MelanA/52-23_MelanA.png]

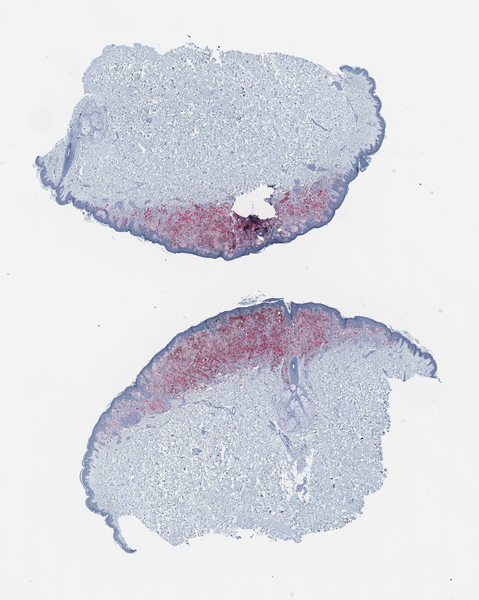

Supplement: S4 Dataset — (ZIP) [file pone.0297146.s010.zip › naples/MelanA/8728-22_MelanA.png]

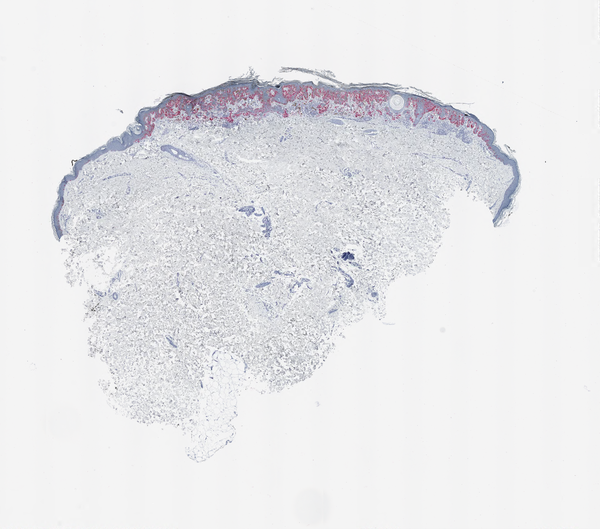

Supplement: S4 Dataset — (ZIP) [file pone.0297146.s010.zip › naples/MelanA/13754-22_MelanA.png]

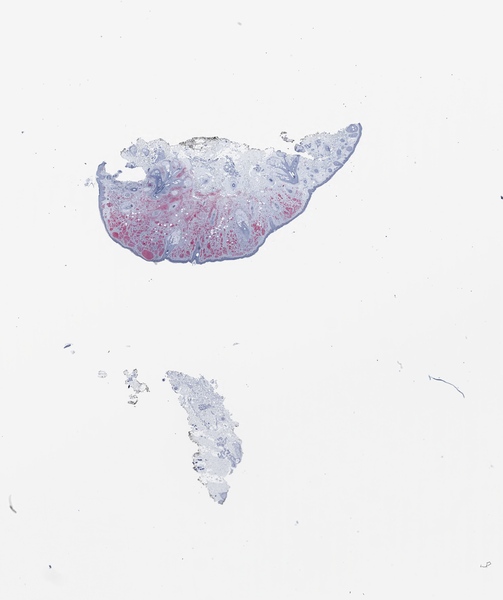

Supplement: S4 Dataset — (ZIP) [file pone.0297146.s010.zip › naples/MelanA/12247-22_MelanA.png]

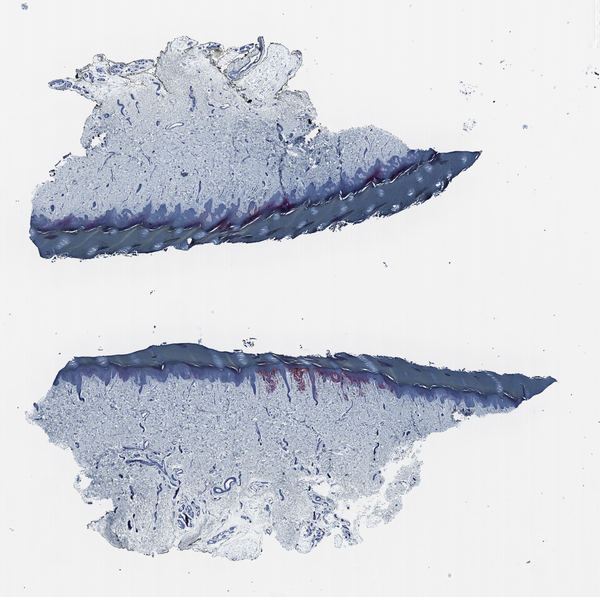

Supplement: S4 Dataset — (ZIP) [file pone.0297146.s010.zip › naples/MelanA/12528-22_MelanA.png]

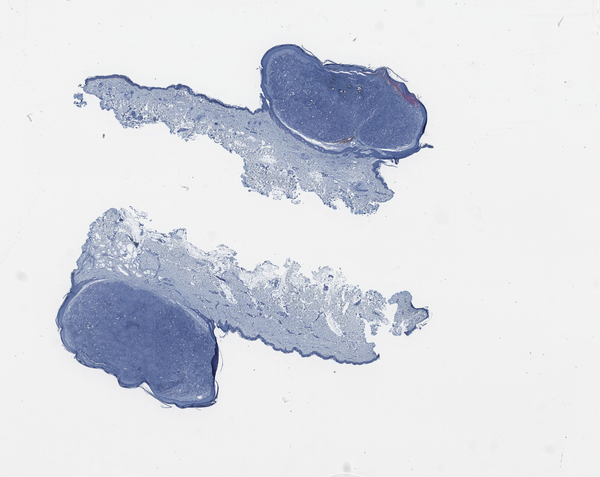

Supplement: S4 Dataset — (ZIP) [file pone.0297146.s010.zip › naples/MelanA/2730-23_MelanA.png]

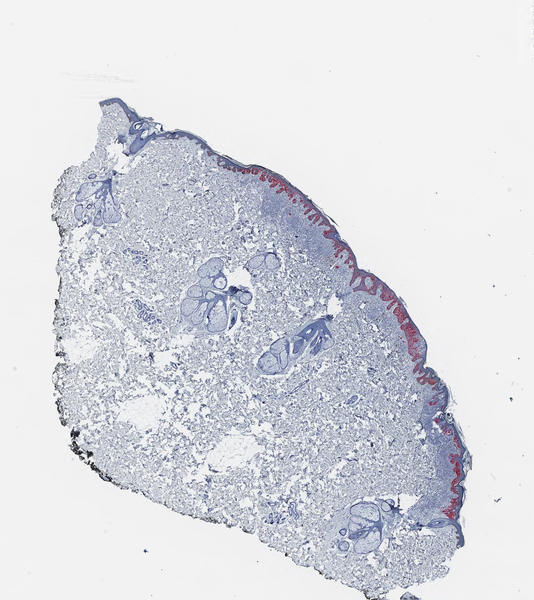

Supplement: S4 Dataset — (ZIP) [file pone.0297146.s010.zip › naples/MelanA/12136-22_MelanA.png]

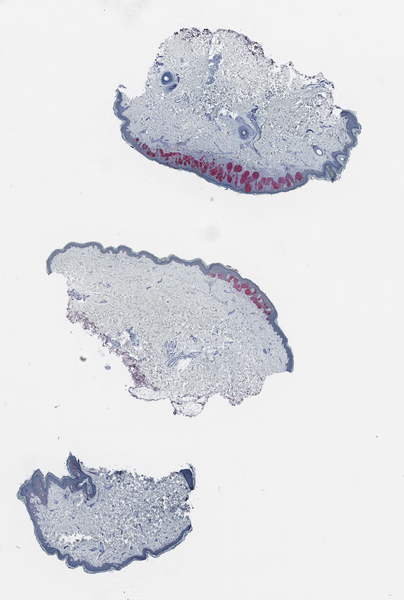

Supplement: S4 Dataset — (ZIP) [file pone.0297146.s010.zip › naples/MelanA/2325-23_MelanA.png]

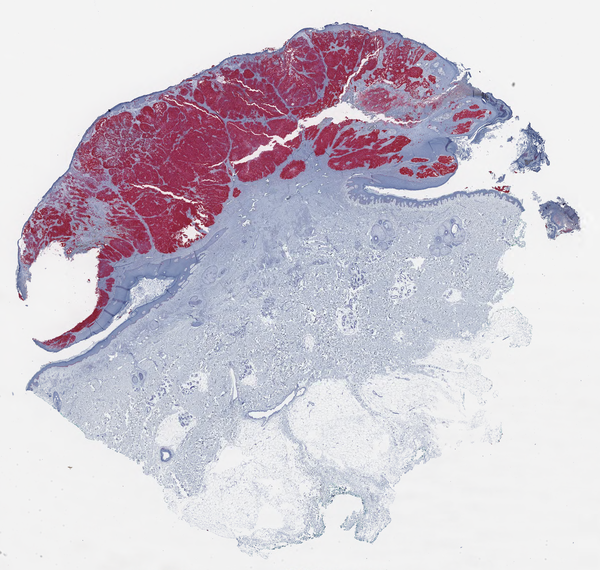

Supplement: S4 Dataset — (ZIP) [file pone.0297146.s010.zip › naples/MelanA/13135-22_MelanA.png]
