# Supplementary material for: Evaluating deep learning-based melanoma classification using immunohistochemistry and routine histology: A three center study
Source: PLoS One. 2024 Jan 19;19(1):e0297146. doi: 10.1371/journal.pone.0297146 (PMC10798511; doi:10.1371/journal.pone.0297146)
Supplement: Attachment — Submitted filename: Review-Letter_with_comments.pdf [file pone.0297146.s012.pdf]

Journal Requirements:

When submitting your revision, we need you to address these additional requirements.

1. Please ensure that your manuscript meets PLOS ONE's style requirements, including those for file naming. The PLOS ONE style templates can be found at https://journals.plos.org/plosone/s/file?id=wjVg/PLOSOne_formatting_sample_main_body.pdf and https://journals.plos.org/plosone/s/file?id=ba62/PLOSOne_formatting_sample_title_authors_affiliations.pdf

Author's response: We updated the information

2. Thank you for including your ethics statement: "Ethics approval was obtained from the ethics committees of therespective universities before the study was initiated. Patients provided informed written consent. This work was performed in accordance with the Declaration of Helsinki".

Please amend your current ethics statement to include the full name of the ethics committee/institutional review board(s) that approved your specific study.

Once you have amended this/these statement(s) in the Methods section of the manuscript, please add the same text to the “Ethics Statement” field of the submission form (via “Edit Submission”).

For additional information about PLOS ONE ethical requirements for human subjects research, please refer to http://journals.plos.org/plosone/s/submission-guidelines#loc-human-subjects-research.

Author's response: We updated the information

3. We note that the grant information you provided in the ‘Funding Information’ and ‘Financial Disclosure’ sections do not match.

When you resubmit, please ensure that you provide the correct grant numbers for the awards you received for your study in the ‘Funding Information’ section.

Author's response: We updated the information

4. Thank you for stating the following in the Competing Interests section:
[TJB would like to disclose that he is the owner of Smart Health Heidelberg GmbH (Handschuhsheimer Landstr. 9/1, 69120 Heidelberg, Germany) which develops mobile apps, outside of the submitted work. SHo reports clinical trial
support from Almirall and speaker’s honoraria from Almirall, UCB and AbbVie and has received travel support from the following companies: UCB, Janssen Cilag, Almirall, Novartis, Lilly, LEO Pharma and AbbVie outside the submitted work.].

Please confirm that this does not alter your adherence to all PLOS ONE policies on sharing data and materials, by including the following statement: ""This does not alter our adherence to PLOS ONE policies on sharing data and materials.” (as detailed online in our guide for authors http://journals.plos.org/plosone/s/competing-interests). If there are restrictions on sharing of data and/or materials, please state these. Please note that we cannot proceed with consideration of your article until this information has been declared.

Author's response: We updated the information.

Please include your updated Competing Interests statement in your cover letter; we will change the online submission form on your behalf.

Author's response: We included the statement in our cover letter.

5. In your Data Availability statement, you have not specified where the minimal data set underlying the results described in your manuscript can be found. PLOS defines a study's minimal data set as the underlying data used to reach the conclusions drawn in the manuscript and any additional data required to replicate the reported study findings in their entirety. All PLOS journals require that the minimal data set be made fully available. For more information about our data policy, please see http://journals.plos.org/plosone/s/data-availability.

""Upon re-submitting your revised manuscript, please upload your study’s minimal underlying data set as either Supporting Information files or to a stable, public repository and include the relevant URLs, DOIs, or accession numbers within your revised cover letter. For a list of acceptable repositories, please see http://journals.plos.org/plosone/s/data-availability#loc-recommended-repositories. Any potentially identifying patient information must be fully anonymized.

Important: If there are ethical or legal restrictions to sharing your data publicly, please explain these restrictions in detail. Please see our guidelines for more information on what we consider unacceptable restrictions to publicly sharing data: http://journals.plos.org/plosone/s/data-availability#loc-unacceptable-data-access-restrictions. Note that it is not acceptable for the authors to be the sole named individuals responsible for ensuring data access.

We will update your Data Availability statement to reflect the information you provide in your cover letter.

Author's response: We uploaded additional data within zip files. This data contains thumbnails of all used slides, as well as model prediction scores for each tile within all the data. Since our dataset is not publicly available yet, due to ethical reasons. This additional information (minimal dataset) provides enough information to reproduce all results presented in this work.

6. We note that you have stated that you will provide repository information for your data at acceptance. Should your manuscript be accepted for publication, we will hold it until you provide the relevant accession numbers or DOIs necessary to access your data. If you wish

to make changes to your Data Availability statement, please describe these changes in your cover letter and we will update your Data Availability statement to reflect the information you provide.

Author's response: All necessary data is already provided in zip files in the supplements.

7. We note that you have included the phrase “data not shown” in your manuscript. Unfortunately, this does not meet our data sharing requirements. PLOS does not permit references to inaccessible data. We require that authors provide all relevant data within the paper, Supporting Information files, or in an acceptable, public repository. Please add a citation to support this phrase or upload the data that corresponds with these findings to a stable repository (such as Figshare or Dryad) and provide and URLs, DOIs, or accession numbers that may be used to access these data. Or, if the data are not a core part of the research being presented in your study, we ask that you remove the phrase that refers to these data.

Author's response: We updated the information

8. Your ethics statement should only appear in the Methods section of your manuscript. If your ethics statement is written in any section besides the Methods, please move it to the Methods section and delete it from any other section. Please ensure that your ethics statement is included in your manuscript, as the ethics statement entered into the online submission form will not be published alongside your manuscript.

Author's response: We updated the information

9. We notice that your supplementary figures and tables are included in the manuscript file. Please remove them and upload them with the file type 'Supporting Information'. Please ensure that each Supporting Information file has a legend listed in the manuscript after the references list.

Author's response: We updated the information

10. Please include captions for your Supporting Information files at the end of your manuscript, and update any in-text citations to match accordingly. Please see our Supporting Information guidelines for more information: http://journals.plos.org/plosone/s/supporting-information.

Author's response: We updated the information: File-Names are: FigureS1, FigureS2, FigureS3, TableS1, TableS2, etc.

11. We note that Figure 1 in your submission contain copyrighted images. All PLOS content is published under the Creative Commons Attribution License (CC BY 4.0), which means that the manuscript, images, and Supporting Information files will be freely available online, and any third party is permitted to access, download, copy, distribute, and use these materials in any way, even commercially, with proper attribution. For more information, see our copyright guidelines: http://journals.plos.org/plosone/s/licenses-and-copyright.

We require you to either (1) present written permission from the copyright holder to publish these figures specifically under the CC BY 4.0 license, or (2) remove the figures from your submission:

A. You may seek permission from the original copyright holder of Figure 1 to publish the content specifically under the CC BY 4.0 license.

We recommend that you contact the original copyright holder with the Content Permission Form (http://journals.plos.org/plosone/s/file?id=7c09/content-permission-form.pdf) and the following text: “I request permission for the open-access journal PLOS ONE to publish XXX under the Creative Commons Attribution License (CCAL) CC BY 4.0 (http://creativecommons.org/licenses/by/4.0/). Please be aware that this license allows unrestricted use and distribution, even commercially, by third parties. Please reply and provide explicit written permission to publish XXX under a CC BY license and complete the attached form.”

Please upload the completed Content Permission Form or other proof of granted permissions as an ""Other"" file with your submission. In the figure caption of the copyrighted figure, please include the following text: “Reprinted from [ref] under a CC BY license, with permission from [name of publisher], original copyright [original copyright year].”

B. If you are unable to obtain permission from the original copyright holder to publish these figures under the CC BY 4.0 license or if the copyright holder’s requirements are incompatible with the CC BY 4.0 license, please either i) remove the figure or ii) supply a replacement figure that complies with the CC BY 4.0 license. Please check copyright information on all replacement figures and update the figure caption with source information. If applicable, please specify in the figure caption text when a figure is similar but not identical to the original image and is therefore for illustrative purposes only.

Author's response: In fact, Figure 1 does not contain a copyrighted image. The images were created by us using the "drawio" software and the elements contained in the figure where created by our own. The following statement can be found on the drawio-website "https://www.drawio.com/doc/faq/usage-terms": "There are no restrictions on the usage of any diagrams you generate with draw.io". It is not necessary to obtain permission.

12. Please review your reference list to ensure that it is complete and correct. If you have cited papers that have been retracted, please include the rationale for doing so in the manuscript text, or remove these references and replace them with relevant current references. Any changes to the reference list should be mentioned in the rebuttal letter that accompanies your revised manuscript. If you need to cite a retracted article, indicate the article’s retracted status in the References list and also include a citation and full reference for the retraction notice.

Author's response:We reviewed the reference list and could not find any missing things or incorrect entries.

Additional Editor Comments (if provided):

[Note: HTML markup is below. Please do not edit.]

Reviewers' comments:

Reviewer's Responses to Questions

**Comments to the Author**

1. Is the manuscript technically sound, and do the data support the conclusions?

The manuscript must describe a technically sound piece of scientific research with data that supports the conclusions. Experiments must have been conducted rigorously, with appropriate controls, replication, and sample sizes. The conclusions must be drawn appropriately based on the data presented.
Reviewer #1: Yes
Reviewer #2: Yes

Author's response: We thank the Reviewer.

2. Has the statistical analysis been performed appropriately and rigorously?
Reviewer #1: Yes
Reviewer #2: Yes

Author's response: We thank the Reviewer.

3. Have the authors made all data underlying the findings in their manuscript fully available?

The PLOS Data policy requires authors to make all data underlying the findings described in their manuscript fully available without restriction, with rare exception (please refer to the Data Availability Statement in the manuscript PDF file). The data should be provided as part of the manuscript or its supporting information, or deposited to a public repository. For example, in addition to summary statistics, the data points behind means, medians and variance measures should be available. If there are restrictions on publicly sharing data—e.g. participant privacy or use of data from a third party—those must be specified.

Reviewer #1: Yes
Reviewer #2: No

Author's response: We have provided all data in the form of csv-files with tile-scores and model weights for each slide.

4. Is the manuscript presented in an intelligible fashion and written in standard English?

PLOS ONE does not copyedit accepted manuscripts, so the language in submitted articles must be clear, correct, and unambiguous. Any typographical or grammatical errors should be corrected at revision, so please note any specific errors here.

Reviewer #1: Yes

Reviewer #2: Yes

Author's response: We thank the Reviewer.

5. Review Comments to the Author

Please use the space provided to explain your answers to the questions above. You may also include additional comments for the author, including concerns about dual publication, research ethics, or publication ethics. (Please upload your review as an attachment if it exceeds 20,000 characters)

Reviewer #1: Titus J. Brinker et al.'s work presents an original computational approach to melanocytic lesions, utilizing clear and elegant methods, yielding promising yet not optimal results for clinical translation. Specifically, the study employs Deep-learning models to analyze histological information extracted from both H&E and Melan-A immunostain, aiming to classify lesions into benign nevus or melanoma. The study is well-executed and well-designed, however, as mentioned in the paper, this approach has certain limitations. One of its inherent shortcomings is the inability to provide a comprehensive evaluation of the entire lesion at low magnification, which is usually crucial in the evaluation of melanocytic lesions, overlooking important features such as the symmetry. Another limitation is the lack of a direct link to histological characteristics and the absence of accompanying histological images, which could significantly improve the accessibility and clarity of the study. On the other hand, the study's main strengths include the evaluation of different immunohistochemical clones, a multicentric approach with a well-balanced dataset and training conducted at various magnifications.

Overall, the study could be improved with a few adjustments:

-In the introduction, it is mentioned that pathologists have up to a 25% interobserver discordance rate, but the references cited are relatively dated (from 15 to 27 years ago), a period during which the evaluation of melanocytic lesions has gained new markers and insights. More recent references would be advisable.
-In the same section, from the sentence beginning with 'If melanoma is initially misclassified...' to the end of the paragraph, references are lacking.

Author's response: We have amended the references with two recent publications from 2014 and 2017 that emphasize the information in the paragraph.

-In the Methods section, Table 1 categorizes cases into three classes, including melanoma in situ. While it is implied that melanoma in situ belongs to the melanoma class, the table's presentation may still cause confusion. To enhance clarity, it is advisable to explicitly specify the relationship between melanoma in situ and the melanoma class.

Author's response: We added a sentence which specifies the relationship between melanoma in situ and malignant melanomas.

-In the same section it is stated that a color augmentation approach was used, but it is not established whether this led to improvements in metrics and domain shift. At least some

commentary on this approach, which sparks debate on model cutoff changes, would be recommended.

Author's response: We amended the references with a recent publication that investigated the difference between color augmentations and normalization, which shows that color augmentation leads to improvements and stain normalization is not necessary in addition.

-In the results section, the initial part appears somewhat repetitive, and it would be advisable to condense this portion within the methods to make the entire methodological process more straightforward.
-Still in the results section, the legend in Figure 2 appears to reverse Figures 2A and 2B.

Author's response: We thank the Reviewer for the advice. We condensed the repetitive information in the methods and corrected the legend in Figure 2

-In the discussion, no explanation or hypothesis is provided for these misclassifications, and it is not described from a histological perspective in which cases the model underperformed. It remains unclear whether this underperformance was solely due to variations in technical slide characteristics or if there are specific subsets of lesions where the model struggled more.

Author's response: We have added another paragraph to the discussion which describes the misclassifications in more detail.

Reviewer #2: Since the objective of the study is not simply the development of a DL-based model to diagnose melanoma on WSI, but a comparison of the performance of DL systems using H&E versus MelanA, without lengthening the introduction, I would integrate it by briefly mentioning a few numbers, namely: (1a) the current state-of-the-art performance of published DL models in melanoma diagnosis, (1b) whether there are public datasets to gauge performance, and (2) the reported accuracy boosts by combining H&E with IHC (in other use-cases if there are none in melanoma).

Author's response: (1a) We added two sources within the introduction section that investigated melanoma/nevus classification on H&E stained images. (1b) To our knowledge there exist no public datasets containing histopathological slides of melanoma and nevus, especially not containing H&E and MelanA as well. (2) To our knowledge there exists no other work that investigated the combination of H&E with IHC to boost accuracy. There are only works investigating multi stained slides like Foersch et al., 2023 Nature Medicine.

It would be useful for the reader if the authors provided a few example WSI thumbnails and fields from the three cohorts, for each staining modality, to gauge the entity of staining/scanning variation between cohorts.

Author's response: We have provided some representative WSI thumbnails as examples in a supplementary figure, as well as all WSI thumbnails of all cohorts in the supplement.

Am I correct in understanding that the patches were extracted without stride/overlap? Was the same done for inference? I would state this clearly in the manuscript.

Author's response: All patches used for training, validation and hold-out testing were extracted without stride/overlap. We added the information in the method section.

Some details regarding model architecture are lacking. In addition to the mentioned pooling strategy, where were models cut, and what custom heads were used?

Author's response: Within the methods part, we added the library that initialized our models. Since we reported all information about hyperparameters in the appendix and all other parameters where left as default, there should be no lacking information left.

What hardware was used? What's the inference time per tile and per WSI?

Author's response: Computation was performed on a Nvidia V100 SXM2. The estimated inference time for predicting labels for a single digitized WSI is approximately 30 seconds with a very high variance, depending on magnification and tumor size.

L127. Training the model at tile level using the slide label has some risks. Were there WSIs in which multiple categories coexisted (i.e. melanoma ± in-situ melanoma ± benign nevus)? How were these handled in training? And in inference?

Author's response: The tumor boundaries of all patient samples were manually annotated based on the MelanA expression on the IHC slides. These boundaries were manually transferred to the corresponding H&E slides. In fact, there may be melanomas with adjacent benign nevus tissue, both of which are annotated as melanoma tissue in this case. To remain unbiased by this tile-level noise, we always trained with all annotated WSI slides to avoid losing potential information. Prediction was performed by averaging the tile scores.

The model input is (a batch of) 237×237px patches, but what about its output? Figure 1 seems to show a binary output (melanoma vs benign?) but you also had in-situ melanoma in the slide categories. What exactly was the output of the model?

Author's response: Melanoma in situ and malignant melanoma differ only in the integrity of the basement membrane. This information is not visible on 237×237px patches. However, the features at the cellular level do not differ between the two classes. The group of melanomas in situ was therefore assigned to the group of malignant melanomas, as both differ from benign nevi at the cellular level. Therefore, the output of the model was binary (melanoma vs benign)

L127 "All tiles of the slide were predicted and the slide score was calculated by averaging all tile scores (see Figure 1)". Can you provide more detail? The answer to this question depends on the previous one. Was the model a binary melanoma/benign model, and was your final label "benign" if benign tiles outnumbered malignant tiles? Was your model a multicategory one (melanoma - melanoma in situ - benign nevus) and did you predict the most common category as the slide label?

Author's response: The model was in fact not a multicategory model, but a binary melanoma/benign model. The slide score was calculated by averaging the tile scores. We

just report for the data description the amount of in-situ melanoma, since these border cases are harder to classify in general.

How did you handle tiles which were not melanoma, melanoma in-situ or nevus? In the training set you annotated and extracted only tiles from the lesion, but what about the test set? Did you run inference on the whole tissue, including epidermis, dermis and subcutis uninvolved by melanocytic tumor? Did you use a tissue detector to filter out empty tiles?

Author's response: All tiles used for the training, validation and hold-out test sets were processed exactly the same. Only MelanA expressing tissue areas were annotated and subsequently empty tiles were sorted out using a reversed red filter. This ensured that tissue from the epidermis, dermis and subcutis predominantly belonged to the melanocytic tumor.

The reported performances of the H&E-only and MelanA-only models make me think. The pathologists among the authors of this work are invited to discuss their interpretation of the fact that the MelanA+H&E classifier does not seem to work better than the simple H&E classifier, at least on the Dresden cohort. What does the pathologist's thought process look like when evaluating MelanA in addition to H&E, and was the combined model designed coherently?

Author's response: We have added another paragraph to the discussion which describes the misclassifications in more detail.

L288 while I agree that each magnification contains partly different information, I don't think you can infer that from the observation that the combination of all 4 magnifications brings a slight overall improvement. Unless you prove that it is not due to ensembling.

Author's response: We thank the Reviewer for the observation, this effect is probably due to ensembling, we have highlighted this in the manuscript.
Wir halten das für ein Pseudo ensembling.

6. PLOS authors have the option to publish the peer review history of their article (what does this mean?). If published, this will include your full peer review and any attached files.

If you choose “no”, your identity will remain anonymous but your review may still be made public.

**Do you want your identity to be public for this peer review?** For information about this choice, including consent withdrawal, please see our Privacy Policy.

Reviewer #1: Yes: Giorgio Cazzaniga
Reviewer #2: Yes: Alessandro Caputo

[NOTE: If reviewer comments were submitted as an attachment file, they will be attached to this email and accessible via the submission site. Please log into your account, locate the manuscript record, and check for the action link "View Attachments". If this link does not appear, there are no attachment files.]

While revising your submission, please upload your figure files to the Preflight Analysis and Conversion Engine (PACE) digital diagnostic tool, https://pacev2.apexcovantage.com/. PACE helps ensure that figures meet PLOS requirements. To use PACE, you must first register as a user. Registration is free. Then, login and navigate to the UPLOAD tab, where you will find detailed instructions on how to use the tool. If you encounter any issues or have any questions when using PACE, please email PLOS at figures@plos.org. Please note that Supporting Information files do not need this step.
